# C9orf72 protein quality control by UBR5‐mediated heterotypic ubiquitin chains

**DOI:** 10.15252/embr.202255895

**Published:** 2023-06-15

**Authors:** Julia Jülg, Dieter Edbauer, Christian Behrends

**Affiliations:** ^1^ Munich Cluster for Systems Neurology, Medical Faculty Ludwig‐Maximilians‐Universität München Munich Germany; ^2^ German Center for Neurodegenerative Diseases Munich Munich Germany

**Keywords:** BAG6 complex, C9orf72, heterotypic ubiquitin chains, K11/K48‐linked ubiquitin, UBR5, Post-translational Modifications & Proteolysis, Translation & Protein Quality

## Abstract

Hexanucleotide repeat expansions within *C9orf72* are a frequent cause of amyotrophic lateral sclerosis and frontotemporal dementia. Haploinsufficiency leading to reduced C9orf72 protein contributes to disease pathogenesis. C9orf72 binds SMCR8 to form a robust complex that regulates small GTPases, lysosomal integrity, and autophagy. In contrast to this functional understanding, we know far less about the assembly and turnover of the C9orf72–SMCR8 complex. Loss of either subunit causes the concurrent ablation of the respective partner. However, the molecular mechanism underlying this interdependence remains elusive. Here, we identify C9orf72 as a substrate of branched ubiquitin chain‐dependent protein quality control. We find that SMCR8 prevents C9orf72 from rapid degradation by the proteasome. Mass spectrometry and biochemical analyses reveal the E3 ligase UBR5 and the BAG6 chaperone complex as C9orf72‐interacting proteins, which are components of the machinery that modifies proteins with K11/K48‐linked heterotypic ubiquitin chains. Depletion of UBR5 results in reduced K11/K48 ubiquitination and increased C9orf72 when SMCR8 is absent. Our data provide novel insights into C9orf72 regulation with potential implication for strategies to antagonize C9orf72 loss during disease progression.

## Graphical abstract

This study identified a quality control mechanism for C9orf72 which is induced by the absence of its complex partner SMCR8, involves K11/K48‐ubiquitination of C9orf72 by the ubiquitin ligase UBR5 and leads to rapid proteasomal degradation of C9orf72.

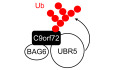

## Introduction

Accurate composition of multiprotein complexes requires constant quality control to assure complex functionality and to maintain proteostasis. C9orf72 forms a robust complex with Smith‐Magenis syndrome chromosome region 8 (SMCR8) to function in endomembrane trafficking processes and lysosomal homeostasis (Amick *et al*, 2016; Sellier *et al*, 2016; Sullivan *et al*, 2016; Yang *et al*, 2016; Jung *et al*, 2017). Together with WD repeat‐containing protein 41 (WDR41), C9orf72 and SMCR8 act as regulators of autophagy, an intracellular degradation pathway for protein aggregates, damaged organelles, and pathogens (Sellier *et al*, 2016; Sullivan *et al*, 2016; Yang *et al*, 2016). Here, the C9orf72‐SMCR8–WDR41 complex associates with the ULK1 complex thereby modulating its kinase activity and thus the formation of autophagosomes by which cytosolic content is engulfed and delivered for turnover to the lysosome (Sullivan *et al*, 2016; Yang *et al*, 2016; Jung *et al*, 2017). Furthermore, cellular localization studies demonstrated the recruitment of the C9orf72–SMCR8–WDR41 complex to the cytosolic surface of lysosomes depending on amino acid availability (Amick *et al*, 2016, 2020). There, the presence of the C9orf72–SMCR8–WDR41 complex is crucial for maintaining lysosomal function (O'Rourke *et al*, 2016; Corrionero & Horvitz, 2018; Amick *et al*, 2020). While C9orf72 and SMCR8 sequences show low similarity, both proteins share a common structural feature as they harbor a predicted DENN protein domain at the C‐terminus (Zhang *et al*, 2012; Levine *et al*, 2013). Members of the DENN domain‐containing proteins act as regulators of small GTPases, which cycle between GTP and GDP nucleotide‐bound states, and in this way drive and regulate diverse endomembrane‐based processes (Fukuda, 2008). Structural elucidation and biochemical approaches suggest that the C9orf72–SMCR8 complex exhibits GDP exchange factor (GEF) and GTPase activating protein (GAP) function, thereby regulating different Rab and ARF GTPases (Farg *et al*, 2014; Sellier *et al*, 2016; Webster *et al*, 2016; Corbier & Sellier, 2017; Tang *et al*, 2020; Su *et al*, 2021).

The importance of C9orf72 is highlighted by its pathological context where expanded GGGGCC nucleotide repeats within the noncoding region of *C9orf72* are one of the leading causes for amyotrophic lateral sclerosis (ALS) and frontotemporal dementia (FTD) (DeJesus‐Hernandez *et al*, 2011; Renton *et al*, 2011). Three molecular mechanisms have been proposed in which *C9orf72* hexanucleotide repeats lead to neurodegeneration: (i) the generation of expended GGGGCC sense and antisense transcripts, which then accumulate in nuclear RNA foci (DeJesus‐Hernandez *et al*, 2011; Gendron *et al*, 2013), (ii) the unconventional translation of these RNA transcripts into distinct aggregation‐prone dipeptide repeat proteins (DPRs) (Ash *et al*, 2013; Mori *et al*, 2013; Zu *et al*, 2013) and (iii) the downregulation of *C9orf72* expression and consequently decrease in C9orf72 protein levels (Gijselinck *et al*, 2012; Frick *et al*, 2018; Saberi *et al*, 2018). While the former two mechanisms imply toxic gain‐of‐functions, which has been established in several studies (Almeida *et al*, 2013; Donnelly *et al*, 2013; Haeusler *et al*, 2014; Mizielinska *et al*, 2014; Zhang *et al*, 2016; Conlon *et al*, 2018; Khosravi *et al*, 2020), the molecular mechanism of how C9orf72 loss‐of‐function contributes to ALS/FTD pathology remains unclear. Nonetheless, it is well established that depletion of C9orf72 affects cellular processes involved in the progression of neurodegenerative diseases. In neuronal cells, decreased C9orf72 expression causes reduced autophagic activity, which is paralleled with impaired clearance of toxic DPRs and increased cell death (Sellier *et al*, 2016; Boivin *et al*, 2020). In addition, the downregulation of C9orf72 in macrophages and microglia results in abnormal endosomal trafficking and altered immune response as evident from the accumulation of defective lysosomes and increased production of cytokines, respectively (O'Rourke *et al*, 2016).

Maintaining C9orf72 protein levels is ensured by the interaction with its complex partner SMCR8. The downregulation of either C9orf72 or SMCR8 causes complex instability and the elimination of the respective binding partner (Sellier *et al*, 2016; Sullivan *et al*, 2016; Zhang *et al*, 2018). The molecular mechanism underlying this mutual dependence between C9orf72 and SMCR8 protein levels is yet unknown. Given the substantial contribution of C9orf72 to cell function and viability but also to neuropathology it is of great relevance to understand this aspect of C9orf72 quality control in order to therapeutically overcome protein loss during disease progression. For this purpose, we generated CRISPR/Cas9 knockout (KO) cell lines for C9orf72 and SMCR8 to examine C9orf72–SMCR8 complex stability at the endogenous level. We show that C9orf72 is degraded by the ubiquitin‐proteasome‐system (UPS) and that the presence of SMCR8 protects C9orf72 from fast protein turnover. We performed immunoprecipitation coupled with mass spectrometry to identify quality control units that target unassembled C9orf72 for proteasomal degradation and found the ubiquitin E3‐ligase UBR5 and the BAG6 chaperone complex to associate with C9orf72. Both proteins were recently described to participate in a specific protein quality control pathway in which K11/K48‐heterotypic ubiquitin chains are assembled on newly synthesized proteins (Yau *et al*, 2017). Consistently, we detected the modification of unassembled C9orf72 with K11/K48‐branched ubiquitin chains. Together, our findings provide evidences for a heterotypic ubiquitin chain‐dependent protein quality control of C9orf72.

## Results

### Loss of SMCR8 causes proteasomal degradation of C9orf72

**Figure 1:**
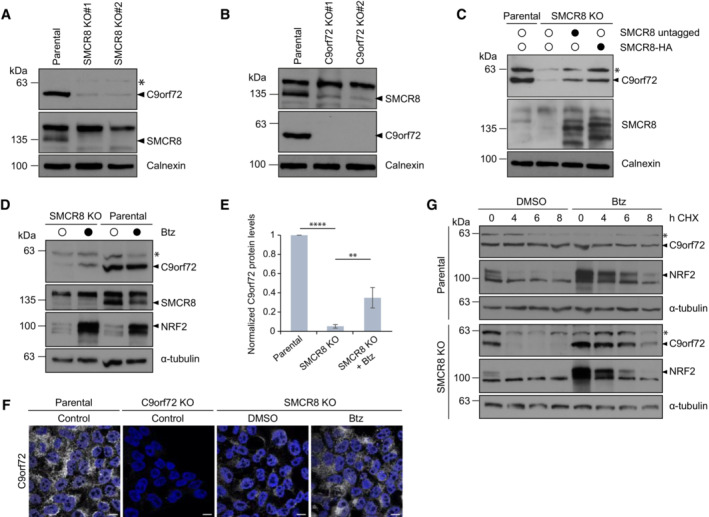
Loss of SMCR8 causes proteasomal degradation of C9orf72 A, BLysates from parental, SMCR8 KO (A) or C9orf72 KO (B) 293T cells were analyzed by immunoblotting.CRescue of C9orf72 protein levels in SMCR8 KO cells after transient overexpression of untagged‐ or C‐terminal tagged SMCR8 analyzed by immunoblotting.DParental and SMCR8 KO cells were treated with DMSO or Bortezomib (Btz) followed by lysis and immunoblotting.EWestern blot quantification of C9orf72 protein levels in parental, SMCR8 KO and SMCR8 KO cells treated with Btz. *n* = 3 biological replicates. Data represent mean ± SD. Statistical analysis of C9orf72/α‐tubulin ratio was performed using one‐tailed, unpaired Student's *t*‐test. **P < 0.01, ****P < 0.0001.FSMCR8 KO cells were treated with DMSO or Btz prior to fixation and immunostaining with an anti‐C9orf72 antibody. Untreated parental and C9orf72 KO cells served as controls. Scale bar, 10 μm.GParental and SMCR8 KO cells were subjected to a cycloheximide (CHX) chase in the absence or presence of DMSO or Btz. Data information: * indicates unspecific protein band detected by C9orf72 antibody. Calnexin or α‐tubulin served as loading control. NRF2 confirmed proteasomal inhibition by Btz. Source data are available online for this figure.

**Figure EV1:**
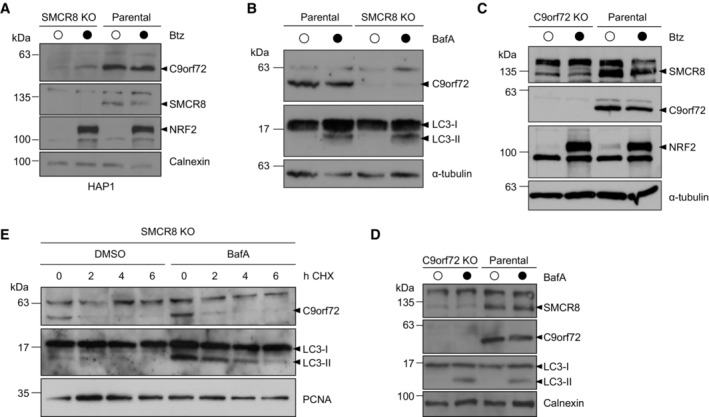
Differential regulation of C9orf72 and SMCR8 protein levels AHAP1 parental and SMCR8 KO cells were treated with DMSO or Btz and analyzed by immunoblotting with indicated antibodies.B293T parental and SMCR8 KO cells were treated with DMSO or Bafilomycin A (BafA) and analyzed by Western blot.C293T parental and C9orf72 KO cells were treated with DMSO or Btz followed by lysis and immunoblotting.D293T parental and C9orf72 KO cells were treated with DMSO or BafA followed by Western blot analysis.E293T SMCR8 KO cells were subjected to a cycloheximide (CHX) chase in the absence or presence of DMSO or BafA and analyzed by immunoblotting. Data information: NFR2 confirmed proteasomal inhibition by Btz. LC3 confirmed the inhibition of autophagosome‐lysosome fusion by BafA. Calnexin, PCNA, and α‐tubulin served as loading controls. Source data are available online for this figure.

Given the previously reported mutual dependence of C9orf72 and SMCR8 protein levels (Amick *et al*, 2016; Sellier *et al*, 2016; Sullivan *et al*, 2016; Ugolino *et al*, 2016; Zhang *et al*, 2018), we characterized the protein abundance of both complex subunits in CRISPR/Cas9 engineered 293T cells, which lack SMCR8 or C9orf72. Deletion of either protein caused a strong reduction in the respective complex partner (Fig 1A and B), confirming the reciprocal regulation of C9orf72 and SMCR8 protein levels. Furthermore, reconstitution of SMCR8 knockout (KO) cells by transient re‐expression of SMCR8 partially rescued the abundance decrease of C9orf72 (Fig 1C), assigning SMCR8 an important role in C9orf72 stabilization. To dissect the molecular events underlying C9orf72 turnover, we treated SMCR8 KO cells with the proteasomal inhibitor Bortezomib (Btz). Compared with DMSO control, C9orf72 partially recovered upon proteasome inhibition as revealed by complementary immunoblotting and immunofluorescence experiments (Figs 1D–F and EV1A). Blocking the lysosomal pathway with Bafilomycin A1 (BafA1), however, did not increase C9orf72 in SMCR8 KO cells (Fig EV1B). This suggests that C9orf72 abundance is controlled post‐translationally by the proteasome. Besides, inhibition of proteasomal or autophagosomal degradation in C9orf72 KO cells could not restore SMCR8 levels (Fig EV1C and D), implying differential regulation of the C9orf72–SMCR8 complex subunits. To corroborate these findings, we performed cycloheximide (CHX) chase assays and monitored C9orf72 protein stability in the presence and absence of SMCR8. Cycloheximide inhibits protein translation, which allows to follow protein degradation over time. When protein translation was blocked in parental cells, C9orf72 levels remained stable for up to 8 h (Fig 1G). By contrast, in the absence of SMCR8 C9orf72 was completely turned over within 4 h. Blocking the proteasome with Btz, in turn, restored C9orf72 levels while BafA1 treatment had no effect (Figs 1G and EV1E). These results suggest that C9orf72 protein turnover is facilitated by the proteasome and is initially triggered by the loss of its complex partner SMCR8.

**Figure 2:**
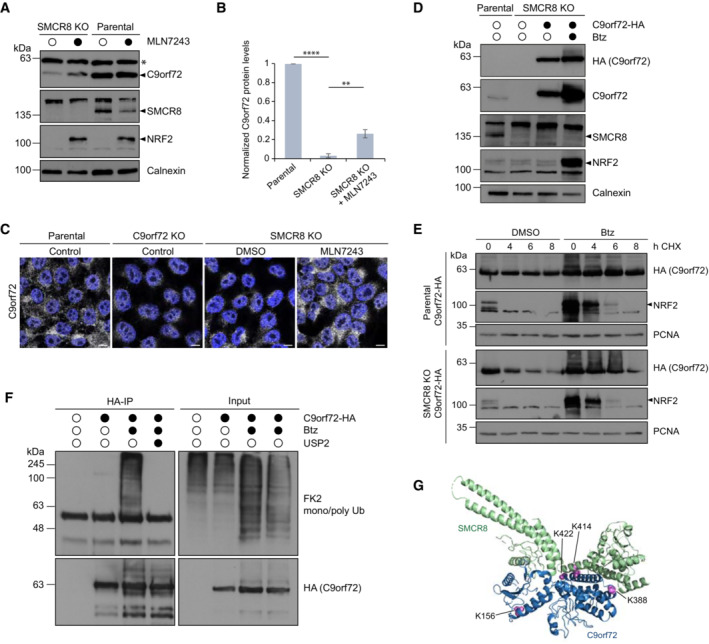
C9orf72 abundance is regulated by ubiquitination A Parental and SMCR8 KO 293T cells were treated with DMSO or MLN7243 followed by lysis and immunoblotting.BWestern blot quantification of C9orf72 protein levels in parental, SMCR8 KO and SMCR8KO cells treated with MLN7243. *n* = 3 biological replicates. Data represent mean ± SD. Statistical analysis of C9orf72/α‐tubulin ratio was performed using one‐tailed, unpaired Student's *t*‐test. **P < 0.01, ****P < 0.0001.CSMCR8 KO cells were treated with DMSO or MLN7243 prior to fixation and immunostaining with an anti‐C9orf72 antibody. Untreated parental and C9orf72 KO cells served as controls. Scale bar, 10 μm.DEmpty or stably C9orf72‐HA expressing SMCR8 KO cells were treated with Btz or DMSO followed by lysis and immunoblotting. Untreated parental cells served as control.EParental and SMCR8 KO cells stably expressing C9orf72‐HA were subjected to CHX chase in absence and presence of DMSO or Btz.FLysates from empty or stably C9orf72‐HA expressing SMCR8 KO cells treated with DMSO or Btz were subjected to HA‐IP under denaturing conditions in the absence and presence of the catalytic domain of the deubiquitinase USP2.GStructural model of C9orf72 (blue) in complex with SMCR8 (green) adapted from PDB 6V4U (Su *et al*, 2021) with potential ubiquitination sites (magenta) in C9orf72 identified by mass spectrometry following denaturing HA‐IP from C9orf72‐HA expressing 293T cells treated with Btz. Data information: Calnexin and PCNA served as loading controls. NRF2 confirmed UAE inhibition by MLN7243 or proteasomal inhibition by Btz. * indicates unspecific protein band detected by C9orf72 antibody. Source data are available online for this figure.

### C9orf72 abundance is regulated by ubiquitination

**Figure EV2:**
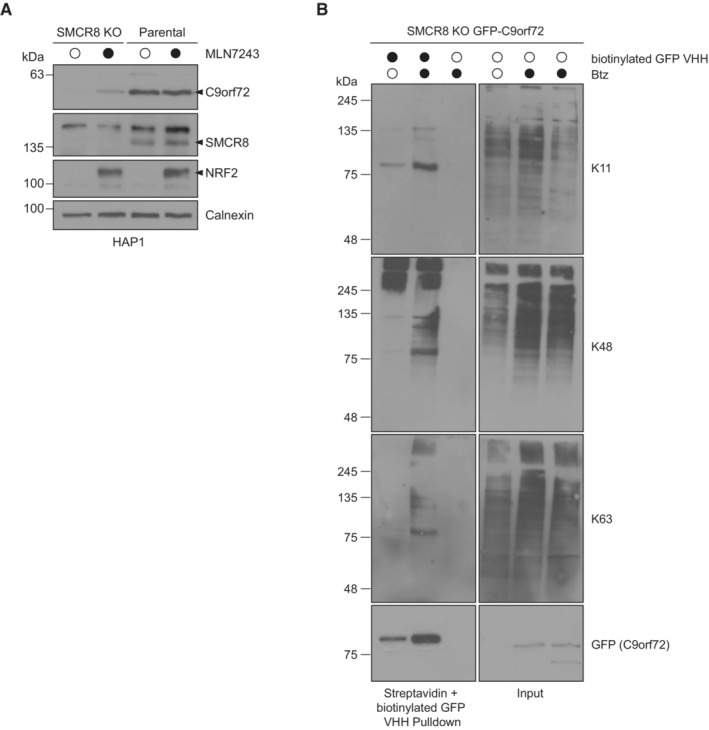
C9orf72 levels upon inhibition of the ubiquitination machinery AHAP1 parental or SMCR8 KO cells were treated with DMSO or MLN7243 and analyzed by Western blot.BSMCR8 KO cells stably expressing GFP‐C9orf72 were treated with DMSO or Btz followed by lysis under denaturing conditions. Lysates were incubated with biotinylated GFP VHH nanobodies coupled with Streptavidin agarose and analyzed by immunoblotting. Source data are available online for this figure.

Since ubiquitination is the signal for proteasomal degradation, we tested whether C9orf72 is a target of the ubiquitin conjugation machinery. Thereto, we employed the small‐molecule inhibitor MLN7243, which blocks the ubiquitin‐activating enzyme (UAE) with the consequence that substrates can no longer be conjugated with ubiquitin. Acute inhibition of ubiquitination in SMCR8 KO cells partially restored C9orf72 protein levels as observed by immunoblotting and immunofluorescence (Figs 2A–C and EV2A). To examine whether C9orf72 is indeed ubiquitinated for its targeting to the proteasome, we generated a cell line stably expressing C‐terminal HA‐tagged C9orf72 in the SMCR8 KO background (Fig 2D). Validation of this cell line showed an increase in C9orf72‐HA upon proteasomal inhibition and unstable C9orf72‐HA levels when SMCR8 was absent and protein translation was blocked (Fig 2D and E), which mimicked the behavior of endogenous C9orf72 in SMCR8 KO cells (Fig 1G). Next, we immunoprecipitated C9orf72‐HA from SMCR8 KO cells under denaturing conditions and probed for ubiquitin conjugates. As expected, C9orf72‐HA was found to be highly ubiquitinated upon proteasome inhibition and this ubiquitination was completely reversed when immunoprecipitated C9orf72‐HA was treated with the deubiquitinating enzyme USP2 (Fig 2F). Mass spectrometry analysis of C9orf72‐HA from denaturing immunoprecipitations unveiled four potential Btz‐sensitive ubiquitination sites, which are located in the N‐terminal (K156) and the C‐terminal (K388, K414, and K422) SMCR8‐binding interface of C9orf72 (Tang *et al*, 2020; Norpel *et al*, 2021; Fig 2G, Table 1). Besides, we detected signature peptides for K48‐, K11‐, and K63‐linked ubiquitin (Table 1). The presence of these three ubiquitin linkage types was further confirmed by Streptavidin pulldowns with biotinylated GFP nanobodies by which eGFP‐C9orf72 was immunoprecipitated under denaturing conditions (Fig EV2B). Overall, these findings indicate an ubiquitin‐dependent regulation of C9orf72.

**Table 1:** Ubiquitin‐remnant diGly profiling of C9orf72‐HA upon Btz treatment.

Intensity Btz_n1	Intensity Btz_n2	Intensity DMSO_n1	Intensity DMSO_n2	Intensity MOCK_n1	Intensity MOCK_n2	Gene names	Positions within proteins	Stringent GlyGly (K) Probabilities
0	0	0	0	8593300	0	CTTNBP2NL	264;264	TLK(1)EEMESLK
2E+09	1,9E+09	3,1E+07	3,2E+07	6540400	8843700	UBB;RPS27A;UBC;UBA52	48;23;30	LIFAGK(1)QLEDGR
2,1E+07	0	0	0	0	0	UBB;RPS27A;UBC;UBA52	63;38;45	TLSDYNIQK(1)ESTLHLVLR
5,4E+07	5,2E+07	0	0	0	0	UBB;RPS27A;UBC;UBA52	11	TLTGK(1)TITLEVEPSDTIENVK
2,4E+08	0	0	0	0	0	BCAP29	85;93;179;179;136;179	K(1)LVEDQEK(1)LK
2,4E+08	0	0	0	0	0	BCAP29	92;100;186;186;143;186	K(1)LVEDQEK(1)LK
1,4E+07	2,3E+07	0	0	0	1,3E+07	LDHA	155	ISGFPK(1)N
0	0	0	0	0	1,4E+07	KIAA1586	633;660	TLFHLCK(1)ILK(1)YEVDLNDFR
0	0	0	0	0	1,4E+07	KIAA1586	636;663	TLFHLCK(1)ILK(1)YEVDLNDFR
0	0	0	0	1,6E+07	1,8E+07	RBMX	22;22;24;22;22	LFIGGLNTETNEK(1)ALEAVFGK
0	0	1,2E+07	0	0	0	R3HDM1	108;125	DEAEK(1)EK(1)ASDK
0	0	1,2E+07	0	0	0	R3HDM1	110;127	DEAEK(1)EK(1)ASDK
1,8E+08	5,2E+08	3,4E+08	5,1E+08	2,4E+08	4,5E+08	KLK11	88;120;261	K(1)PGVYTK
0	0	0	1,9E+07	0	0	RGN	98	K(1)NNRFNDGK
8811300	7175700	0	0	0	0	C9orf72	388	AFLDQVFQLK(1)PGLSLR
0	4856000	0	0	0	0	C9orf72	414	ALTLIK(1)YIEDDTQK
2,6E+08	1,4E+08	0	0	0	0	C9orf72	422	YIEDDTQK(1)GK
9,4E+07	8,5E+07	0	0	0	0	C9orf72	156	QENVQK(1)IILEGTER
0	1E+07	0	0	0	0	TBK1	231	NK(0.5)EVMYK(0.5)IITGK
0	1E+07	0	0	0	0	TBK1	236	NK(0.5)EVMYK(0.5)IITGK

C9orf72‐HA expressing SMCR8 KO cells were treated with DMSO or Btz, subjected to denaturing HA‐IP and processed by in‐gel tryptic digest and MS analysis. Empty SMCR8 KO cells (MOCK) served as control. Data derive from two technical replicates.

### C9orf72 associates with the E3 ligase UBR5 and BAG6 chaperone complex

**Figure 3:**
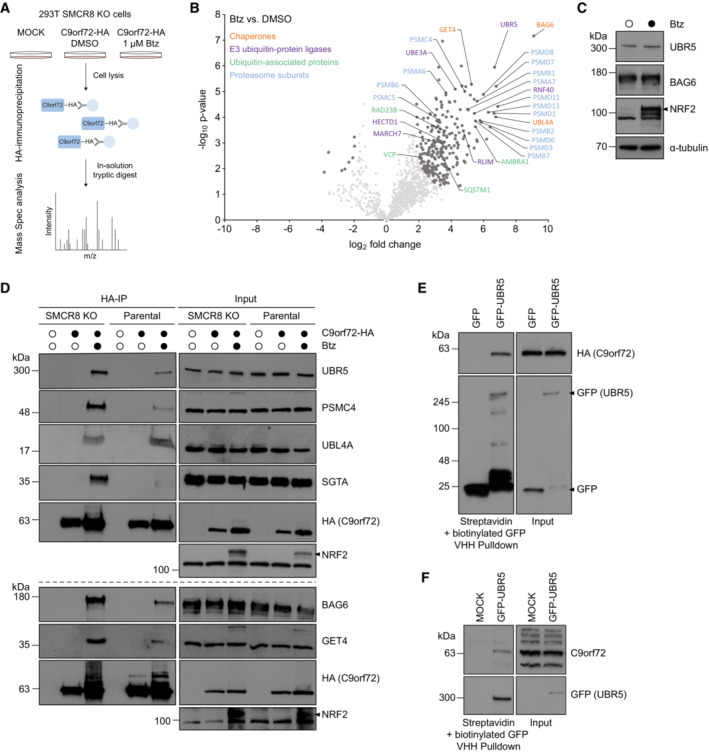
C9orf72 associates with the E3 ligase UBR5 and BAG6 chaperone complex AExperimental workflow to identify C9orf72 quality control factors.BVolcano plot of C9orf72 candidate interacting proteins upon proteasomal inhibition. Lysates from C9orf72‐HA expressing SMCR8 KO cells treated with DMSO or Btz were subjected to HA‐IP and C9orf72 immune complexes were analyzed by mass spectrometry. *n* = 4 biological replicates with each including *n* = 2 technical replicates. Pearson's correlation, DMSO ≥ 0.747, Btz ≥ 0.873. Interacting candidates reaching FDR < 0.01, s0 = 1 (Student's *t*‐test) are highlighted in dark gray.CC9orf72‐HA expressing SMCR8 KO cells were treated with DMSO or Btz and analyzed by immunoblotting. α‐tubulin served as loading control.DLysates from C9orf72‐HA expressing parental and SMCR8 KO cells treated with DMSO or Btz were subjected to HA‐IP followed by immunoblot analysis. Empty parental and SMCR8 KO served as controls.ESMCR8 KO cells expressing C9orf72‐HA were transiently transfected with GFP‐UBR5 or GFP and treated with DMSO or Btz. Lysates were incubated with Streptavidin agarose coupled with biotinylated GFP VHH nanobodies and bound proteins were analyzed by immunoblotting.FParental 293T cells were transiently transfected with GFP‐UBR5 or MOCK followed by pulldown and immunoblot analysis. Data information: NRF2 confirmed proteasomal inhibition by Btz. MOCK refers to control transfection without plasmid. Source data are available online for this figure.

**Figure 4:**
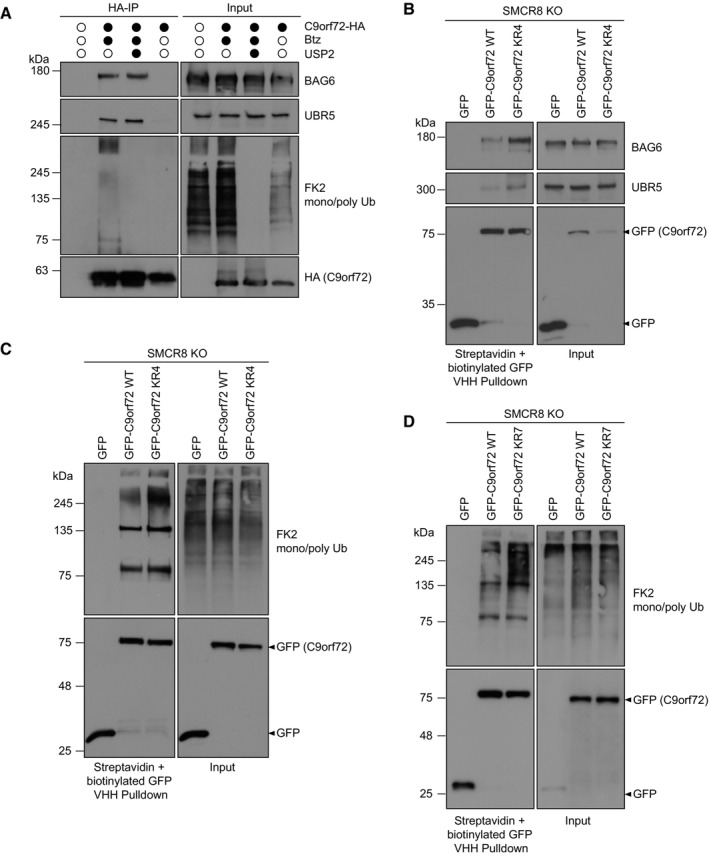
C9orf72‐binding properties of UBR5 and BAG6 ASMCR8 KO cells stably overexpressing C9orf72‐HA were treated with Btz and lysates were subjected to HA‐IP under mild conditions in the absence and presence of the catalytic domain of the deubiquitinase USP2. FK2 confirmed deubiquitination by USP2.BSMCR8 KO cells stably overexpressing GFP, GFP‐C9orf72 wild‐type (WT) or GFP‐C9orf72 mutant K156R, K388R, K414R, K422R (KR4) were treated with Btz and subjected to streptavidin agarose coupled with biotinylated GFP VHH nanobodies.CSMCR8 KO cells stably overexpressing GFP, GFP‐C9orf72 WT or GFP‐C9orf72 KR4 were treated with Btz followed by streptavidin pulldown with biotinylated GFP VHH nanobodies under denaturing conditions.DSMCR8 KO cells transiently overexpressing GFP, GFP‐C9orf72 WT or GFP‐C9orf72 mutant K156R, K388R, K414R, K422R, K424R, K425R, K428R (KR7) were treated with Btz followed by streptavidin pulldown with biotinylated GFP VHH nanobodies under denaturing conditions. Source data are available online for this figure.

**Figure EV3:**
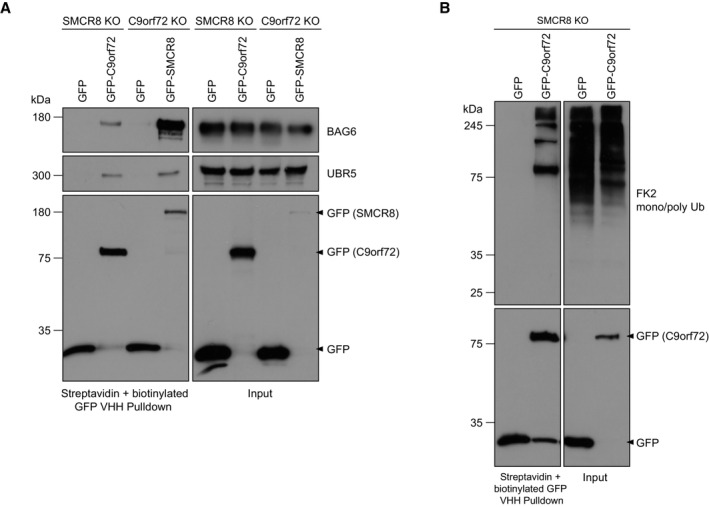
Interaction of BAG6 and UBR5 with uncomplexed C9orf72 and SMCR8 A293T SMCR8 KO and C9orf72 KO cells were transfected with GFP‐C9orf72 and GFP‐SMCR8, respectively. GFP served as control. Cells were lysed under mild conditions, lysates were incubated with biotinylated GFP VHH coupled with Streptavidin agarose and analyzed by Western blot.BSMCR8 KO cells stably expressing GFP or GFP‐C9orf72 were treated with Btz and subjected to streptavidin pulldown using biotinylated GFP VHH nanobodies under denaturing conditions. Source data are available online for this figure.

To identify cellular quality control units that recognize and process C9orf72 for proteasomal degradation in the absence of SMCR8, we performed another round of HA‐immunoprecipitations (IPs) from mildly lysed C9orf72‐HA expressing SMCR8 KO cells and analyzed the C9orf72 interactome by mass spectrometry using label‐free quantification (Fig 3A). Comparing Btz treatment to DMSO control revealed an entire quality control machinery significantly enriched by C9orf72 (Fig 3B, Dataset EV1). Among them, we detected several ubiquitin E3 ligases (UBR5, RNF40, UBE3A, HECTD1, MARCH7, RLIM), ubiquitin‐associated proteins including VCP, RAD23B, AMBRA1, and SQSTM1; the BAG6 chaperone complex; and multiple proteasomal subunits. Intriguingly, the BAG6 complex and UBR5 scored as the most prominent C9orf72 candidate interactors. To exclude that the enrichment of C9orf72‐binding partners was due to an overall increase in abundance of candidate interacting proteins following Btz treatment, we monitored the protein levels of BAG6 and UBR5 and did not observe any overt changes after blocking the proteasome (Fig 3C). Next, we sought to validate the interaction of C9orf72 with these quality control components. Using HA‐IP and Western blotting, BAG6 and its complex members GET4, UBL4A, and SGTA as well as the E3 ligase UBR5, and the proteasomal subunit PSMC4 were all confirmed to bind C9orf72 (Fig 3D). With the exception of UBL4A, these interactions tended to be increased in the absence of SMCR8 when comparing IPs from parental and SMCR8 KO cells. Further, we co‐expressed GFP‐UBR5 or GFP and C9orf72‐HA in SMCR8 KO cells and performed Streptavidin pulldowns with biotinylated GFP nanobodies. In this setting, C9orf72 was exclusively pulled down with GFP‐UBR5 (Fig 3E). In addition, we detected endogenous C9orf72 bound to GFP‐UBR5 when the latter was expressed in parental cells (Fig 3F). To exclude the possibility that UBR5 and BAG6 only bind to ubiquitinated C9orf72, we differentially treated immunoprecipitated C9orf72‐HA from SMCR8 KO cells in mild conditions with the deubiquitinase USP2. As expected, UBR5 and BAG6 bound C9orf72 independent of its ubiquitination status (Fig 4A). Next, we asked whether mutation of the Btz‐sensitive ubiquitination sites (K156, K388, K414, and K422) in C9orf72 would affect its binding to UBR5 and BAG6. Intriguingly, the binding of both proteins to a variant in which these sites were mutated to arginine (C9orf72 KR4) was markedly increased compared with wild‐type C9orf72 (Fig 4B). Consistent with this increased UBR5 binding, we also observed elevated ubiquitination of C9orf72 KR4 (Fig 4C), which was sustained when adjacent lysine residues (K424, K425, K428) were additionally mutated to arginine in the C9orf72 KR7 variant (Fig 4D). Together, these results highlight UBR5 and BAG6 as potential effectors of C9orf72 in the absence of SMCR8. Intriguingly, we also observed the binding of UBR5 and BAG6 to SMCR8 when C9orf72 was absent (Fig EV3A). While this finding suggests that these quality control components might be dedicated to survey complex assembly in general, this remains speculative and requires further analysis.

### Depletion of UBR5 reduces K11/48 ubiquitination of C9orf72 and increases C9orf72 abundance

**Figure 5:**
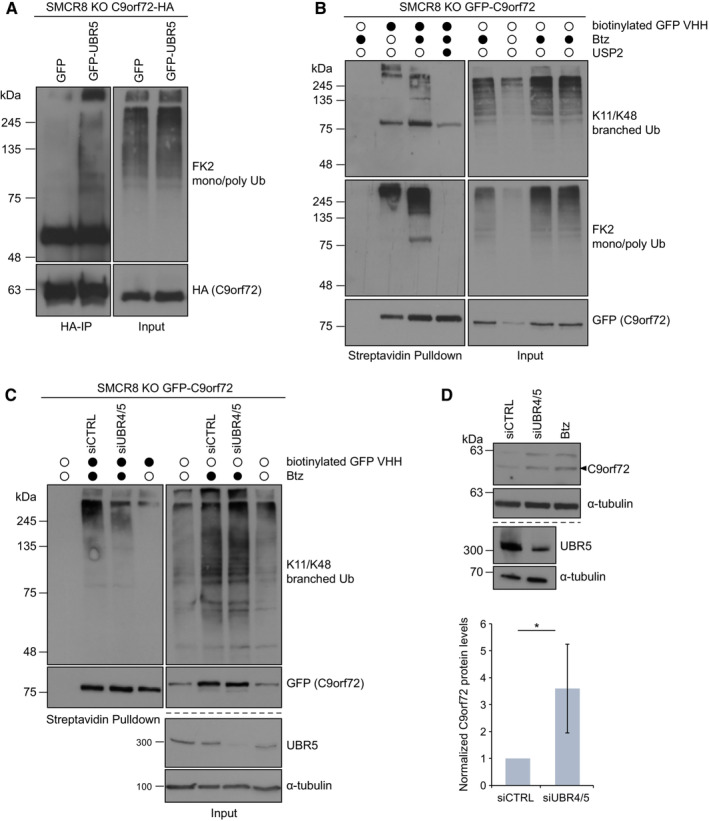
Depletion of UBR5 reduces K11/48 ubiquitination of C9orf72 and increases C9orf72 abundance ASMCR8 KO cells stably expressing C9orf72‐HA were transiently transfected with GFP or GFP‐UBR5 and treated with Btz followed by HA‐IP under denaturing conditions.BSMCR8 KO cells stably expressing GFP‐C9orf72 were treated with DMSO or Btz followed by denaturing lysis and differential USP2 treatment. Lysates were incubated with biotinylated GFP VHH nanobodies coupled with Streptavidin agarose and enriched proteins were analyzed by immunoblotting.CGFP‐C9orf72 expressing SMCR8 KO cells were transiently transfected with siRNAs targeting UBR4 and UBR5 (siUBR4/5) or nontargeting siRNA control (siCTRL) and treated with DMSO or Btz. Denatured lysates were incubated with biotinylated GFP VHH nanobodies coupled with Streptavidin agarose followed by immunoblot analysis.DSMCR8 KO cells were transiently transfected with siUBR4/5, siCTRL or left untreated but grown in the presence of Btz. Lysates were subjected to immunoblotting. Quantification of C9orf72 levels in SMCR8 KO cells treated with siUBR4/5 or siCTRL. *n* = 3 biological replicates. Data represent mean ± SD. Statistical analysis of C9orf72/α‐tubulin ratio was performed using one‐tailed, unpaired Student's *t*‐test. *P < 0.05. Representative Western blot is shown beside. Data information: α‐tubulin served as loading control. Source data are available online for this figure.

The E3 ligase UBR5 and the chaperone BAG6 were recently discovered to mediate a linkage‐specific quality control pathway involving K11/K48‐heterotypic ubiquitin chains, which target proteins for proteasomal degradation (Yau *et al*, 2017). The strong association of C9orf72 with the BAG6 complex and UBR5 raised the question of whether C9orf72 is a substrate of this pathway. To test this hypothesis, we firstly co‐expressed GFP‐UBR5 or GFP with C9orf72‐HA in SMCR8 KO cells and performed denaturing immunoprecipitations. As expected, only UBR5 overexpression led to a prominent ubiquitination of C9orf72 (Fig 5A). Secondly, we analyzed the ubiquitin chain linkage type of GFP‐C9orf72 precipitated under denaturing conditions from SMCR8 KO cells treated with Btz. Using a K11/K48‐specific antibody, we found that C9orf72 was indeed modified with K11/K48‐heterotypic ubiquitin chains upon proteasome inhibition (Figs 5B and EV3B). USP2 deubiquitinates ubiquitin assemblies independent from their linkage type and is therefore able to also cleave K11/K48‐heterotypic chains (Boughton *et al*, 2020). When incubating immunoprecipitated GFP‐C9orf72 from SMCR8 KO cells with USP2, K11/K48‐linked conjugates were completely removed from GFP‐C9orf72 (Fig 5B). Since UBR5 teams up with another E3 ligase, UBR4, for the synthesis of K11/K48‐linked ubiquitin chains, it requires the depletion of both enzymes to overall decrease this heterotypic ubiquitination (Yau *et al*, 2017). Therefore, we co‐depleted UBR5 and UBR4 from SMCR8 KO cells expressing GFP‐C9orf72 and examined the levels of K11/K48 ubiquitin conjugates on GFP‐C9orf72. Compared with nontargeting control, double knockdown of UBR4 and UBR5 decreased K11/K48 ubiquitination of GFP‐C9orf72 (Fig 5C). Consistently, co‐depletion of UBR5 and UBR4 also increased C9orf72 protein levels in SMCR8 KO cells (Fig 5D). In summary, these findings show that C9orf72 is a substrate of the UBR5/UBR4‐driven K11/K48‐ubiquitin‐specific quality control pathway.

## Discussion

**Figure 6:**
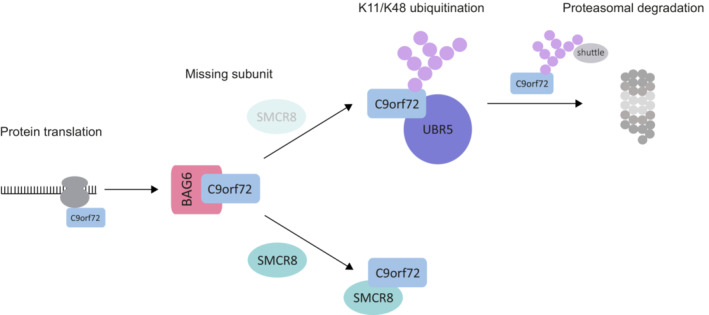
Model of C9orf72 quality control mediated by BAG6 and UBR5 Following protein synthesis, the BAG6 chaperone complex associates with C9orf72 and assists in the assembly of the C9orf72–SMCR8 complex. If SMCR8 is missing, orphaned C9orf72 is targeted by the E3 ligase UBR5 for K11/K48 ubiquitination and send for proteasomal degradation.

C9orf72 function in complex with SMCR8 is crucial for membrane trafficking and organelle integrity, whereas the loss of C9orf72 is strongly associated with neurodegeneration. While the mutual regulation of C9orf72 and SMCR8 abundance (Amick *et al*, 2016; Sellier *et al*, 2016; Sullivan *et al*, 2016; Ugolino *et al*, 2016; Zhang *et al*, 2018) and the stabilization of C9orf72 by SMCR8 (Ugolino *et al*, 2016; Leskela *et al*, 2019) has been established, the underlying molecular pathways remained elusive. Here, we identified a protein quality control mechanism for uncomplexed C9orf72, which involves its modification with specific K11/K48‐branched ubiquitin chains through the ubiquitin E3 ligase UBR5, ultimately leading to rapid proteasomal degradation of C9orf72 (Fig 6). Notably, this does not exclude that other mechanisms including autophagy contribute to the control of C9orf72 abundance when it is in complex with SMCR8.

Monitoring C9orf72 protein levels in the presence and absence of SMCR8 led us to the conclusion that the loss of SMCR8 is the initial event designating C9orf72 for degradation. We made use of this context to investigate the components of the ubiquitin‐proteasome system facilitating C9orf72 elimination and found that C9orf72 is highly ubiquitinated at multiple sites including lysine residues K157, K388, K414, and K422. Interestingly, C9orf72 harbors two SMCR8‐binding interfaces from which the first interface spans amino acids (aa) 13–198 and the second aa 392–418 (Norpel *et al*, 2021). The fact that the identified ubiquitin acceptor sites are located within these two interfaces indicates that uncomplexed C9orf72 is specifically recognized and earmarked for degradation since these lysine residues are most likely buried in the fully assembled SMCR8–C9orf72 complex. Conversely, quality control units might not be able to modify C9orf72 in a complex with SMCR8, thereby providing a possible explanation for the high stability of C9orf72 when SMCR8 is present. However, an important question remains how uncomplexed C9orf72 is recognized by the cellular quality control system.

Our search for effectors and regulators of C9orf72 proteasomal degradation revealed BAG6 and UBR5 as promising candidates. UBR5 belongs to the HECT E3 ligase family and is essential for proteostasis (Callaghan *et al*, 1998; Koyuncu *et al*, 2018). BAG6, together with UBL4A and GET4, forms the BAG6 chaperone complex, which is involved in several quality control pathways for example in the sorting of membrane proteins to either the endoplasmic reticulum or the proteasome (Hessa *et al*, 2011; Shao *et al*, 2017). Consistently, we also detected UBL4A and GET4 enriched by uncommitted C9orf72. Interaction of the majority of the BAG6‐UBR5 machinery with C9orf72 in the absence of SMCR8 supports our hypothesis that orphan C9orf72 is a preferred target for degradation. The observation that UBL4A binds C9orf72 equally well regardless of SMCR8 absence or presence indicates that the BAG6 complex or at least parts thereof might be involved in priming C9orf72 for complex formation with SMCR8 following protein synthesis.

Intriguingly, BAG6 and UBR5 were recently identified as critical components of a specific quality control pathway that earmarks its substrates for proteasomal degradation with K11/K48‐heterotypic ubiquitin chains (Yau *et al*, 2017). In this pathway, VCP and p62, which were also enriched with uncommitted C9orf72, function as proteasomal shuttles that recognize and guide K11/K48‐ubiquitinated substrates to the proteasome (Yau *et al*, 2017). The fact that all components of this quality control machinery (including VCP and p62) were significantly enriched in our C9orf72 interaction proteomics raised the question of whether C9orf72 is a target of K11/K48 ubiquitination. Indeed, the depletion of UBR4 and UBR5 reduced K11/K48 ubiquitination of C9orf72 and partially recovered C9orf72 abundance. Importantly, UBR4 and UBR5 are considered essential E3 ligases to synthesize K11/K48‐ubiquitin chains as it requires only the depletion of these two to reduce the cellular K11/K48 ubiquitination signal (Yau *et al*, 2017). In what way UBR4 and UBR5 collaborate in this pathway, is yet unclear. UBR5 seems to act alone on C9orf72 since we did not detect any specific UBR4‐derived peptides in C9orf72 immune complexes. Based on these findings, we therefore propose a model in which the BAG6 chaperone complex binds to C9orf72 following protein synthesis and assures the formation of a functional complex with SMCR8. When SMCR8 is missing, C9orf72 is modified by the E3 ligase UBR5 with K11/K48‐branched ubiquitin chains, which encodes the signal for rapid turnover by the proteasome (Fig 6). Notably, it remains to be examined whether other ubiquitin ligases present in our C9orf72 proteomics (e.g., RNF40, UBE3A, HECTD1, MARCH7, RLIM) contribute to ubiquitination and proteasomal targeting of uncomplexed C9orf72. The fact that we also detect signature peptides for K63‐linked ubiquitin in C9orf72 IPs indicates that ubiquitination of C9orf72 likely extends beyond K11/K48‐branched ubiquitin chains.

Protein quality control ensures that newly synthesized proteins acquire their correct folding and location within the cell but also assists in their proper assembly into larger protein complexes (Juszkiewicz & Hegde, 2018). Failures in these steps threaten the proteome to be imbalanced and require the instant elimination of misfolded, mislocalized, or orphaned proteins from the cell (Harper & Bennett, 2016). It is therefore conceivable that without its complex partner SMCR8, C9orf72 might lose its functional relevance or might even be harmful to the cell. Consistent with this notion, the majority of reported functions for C9orf72 are assigned to its role within the C9orf72–SMCR8 complex (Amick *et al*, 2016; Sellier *et al*, 2016; Sullivan *et al*, 2016; Webster *et al*, 2016; Yang *et al*, 2016; Jung *et al*, 2017; Tang *et al*, 2020; Su *et al*, 2021). However, since the protein abundance of both subunits is co‐regulated, the individual roles of both proteins remain largely elusive. K11/K48 ubiquitination might be in particular suited to facilitate rapid degradation of orphan C9orf72 to avoid aberrant interaction since the modification with branched ubiquitin chains is considered to massively increase substrate recognition by the proteasome (Meyer & Rape, 2014).

For the future, it will be important to examine whether C9orf72 complex formation with SMCR8 is compromised when neuronal cells accumulate aggregation‐prone DPRs and whether this in turn then triggers UBR5‐mediated degradation of C9orf72. A central aspect of C9orf72 GGGGCC repeat expansion‐driven ALS/FTD pathogenesis is that C9orf72 loss‐of‐function affects essential processes including autophagy, lysosome integrity, and signaling, as well as membrane organization. The findings provided here could possibly be exploited to module C9orf72 protein abundance when inaccurate C9orf72–SMCR8 complex assembly leads to increased degradation of C9orf72. In summary, our work identified C9orf72 as a substrate of UBR5‐mediated K11/K48 ubiquitination providing novel insights into the quality control of C9orf72.

## Materials and Methods

### Plasmids and cloning

C9orf72 ORF (Horizon) was amplified with attB recombination sites by PCR and cloned into the entry vector pDONR223, pHAGE‐CMV‐C‐Flag‐HA, and pHAGE‐N‐eGFP using Gateway^®^Technology. GFP‐UBR5 plasmid (#52050) was acquired from Addgene.

### Cell culture and stable cell line generation

293T cells (ATCC, CRL‐3216, tested negative for mycoplasma) were cultured in Dulbecco's Modified Eagle's Medium (DMEM, Gibco) complemented with 10% fetal bovine serum (FBS) and 1% sodium pyruvate (Gibco) and HAP1 cells were cultured in Iscove's Modified Dulbecco's Medium (IMDM, Gibco) supplemented with 10% FBS and 1% L‐Glutamine (Gibco) at 37°C and 5% CO_2_. For stable cell lines, the medium was supplemented with 2 μg/ml puromycin dihydrochloride (Sigma) or 6 μg/ml blasticidin (InvivoGen). Stable cell lines expressing C9orf72‐HA or eGFP‐C9orf72 WT and KR mutants were generated by lentiviral transduction. For virus production, destination vector and lentiviral packaging plasmids pMD2.G and psPAX2 were co‐transfected into 293T cells using Lipofectamine 2000 (Invitrogen) according to the manufacturer's instructions. Recipient 293T cells were incubated with lentiviral particles and 8 μg/ml polybrene (Merck Millipore) followed by antibiotic selection. C9orf72 knockout 293T cells were generated by CRISPR/Cas genome editing. sgRNAs were designed using the GPP sgRNA Designer tool (Broad Institute) and inserted into the pLentiCRISPR‐HF1‐Puro (Addgene) vector. Lentivirus production and transduction of recipient cells were performed as described above. Single‐cell clones were obtained by FACS sorting and analyzed by immunoblotting.

### Treatments and transfections

Reagents were used with the following concentrations and incubation times: 100 nM Bafilomycin A (Biomol) for 4 h, 1 μM Bortezomib (LC Labs) for 8 h, 100 μg/ml Cycloheximide (Calbiochem) up to 8 h, 1 μM UAE inhibitor MLN7243 (Takeda) for 4 h and 500 nM USP2 enzyme (BostonBiochem) for 1 h. For transient transfection of 293T cells, plasmid DNA and polyethylenimine (PEI, Polyscience Europe) were mixed in optiMEM (Gibco) for 20 min at room temperature (RT) and cells were incubated with this mix for 48 h. For siRNA knockdowns, 293T cells were reversely transfected using Lipofectamine RNAiMAX (Invitrogen) and ON‐target individual siRNAs (Dharmacon Horizon) targeting UBR4 (J‐014021‐10) and UBR5 (J‐007189‐07) or nontargeting siRNA control (D‐001810‐10). Cells were harvested 30 h after transfection.

### Antibodies

Primary antibodies BAG6 (Proteintech, 26417‐1‐AP), Calnexin (Abcam, ab22595), C9orf72 (GeneTex, GTX632041), C9orf72 (Proteintech, 25757‐1‐AP), GET4 (Proteintech, 27768‐1‐AP), GFP (Roche, 11814460001), HA (Cell Signaling, 3724S), NRF2 (Abcam, ab62352), PSMC4 (Bethyl Laboratories, A303‐850A), SGTA (Proteintech, 11019‐2‐AP), SMCR8 (Abcam, ab202283), α‐tubulin (Abcam, ab7291), Ubiquitin clone FK2 (Enzo Life Sciences, BML‐PW8810), Ubiquitin K11 clone 2A3/2E6 (Genentech), Ubiquitin K48 clone Apu2 (Millipore, 05‐1307), Ubiquitin K63 clone Apu3‐A8 (Genentech), Ubiquitin K11/K48‐bispecific (Genentech), UBL4A (Proteintech, 14253‐1‐AP) and UBR5 (Cell Signaling, 65344) were used for immunoblotting at a dilution of 1:1,000 in 5% milk in Tris‐buffered saline with Tween20 (TBS‐T). C9orf72 (Proteintech, 22637‐1‐AP) antibody was used for immunofluorescence staining at a dilution of 1:50 in 0.1% BSA in PBS. For secondary antibodies in immunoblotting anti‐mouse‐HRP (Promega, W402B), anti‐rabbit‐HRP (Promega, W401B), and anti‐human‐HRP (Promega, W403B) were applied at a dilution of 1:10,000. For immunofluorescence, the secondary antibody goat anti‐rabbit AlexaFluor™594 (Life Technologies, A11012) was used at a dilution of 1:1,000.

### Immunofluorescence

293T cells were seeded on poly‐L‐lysine (Sigma) coated coverslips. After washing 3× with Dulbecco's Balanced Salt Solution (DPBS, Gibco), cells were fixed with 4% paraformaldehyde (PFA, ChemCruz) for 10 min and then permeabilized with 0.5% Triton X‐100 for 10 min. Cells were blocked with 1% bovine serum albumin (BSA) for 1 h and stained with primary and then secondary antibody for each 1 h at RT. Coverslips were mounted using Prolong DAPI (Invitrogen). Images were recorded with a Zeiss LSM800 confocal microscope and processed with ZEN 2011 (Blue edition) software.

### Immunoblotting

Cell pellets were lysed in RIPA buffer (50 mM Tris–HCl pH 7.4, 150 mM NaCl, 0.1% SDS, 0.5% sodium deoxycholate, 1% NP‐40) supplemented with protease and phosphatase inhibitors (Roche), incubated for 30 min on ice and centrifuged at 20,000 *g*, 4°C for 10 min. Protein concentration was adjusted to 2 μg/μl by BCA assay and samples were boiled in sample buffer (200 mM Tris–HCl pH 6.8, 6% SDS, 20% glycerol, 0.1 g/ml DTT, 0.01 mg bromophenol blue) for 5 min at 95°C. 20 μg protein was subjected to custom‐made polyacrylamide gels or 4–20% Mini‐PROTEAN TGX gels (Bio‐Rad) and proteins were transferred to a 0.45 μm nitrocellulose (Cytiva Amersham™) or polyvinylidene difluoride (PVDF, Merck Millipore) membrane. Membranes were blocked in 5% milk in TBS‐T and incubated with primary antibodies overnight at 4°C or for 1 h at RT. Following washing in TBS‐T, membranes were incubated with secondary antibodies for 1 h at RT and washed again. Enhanced chemiluminescence (ECL, Perkin Elmer) was applied on membranes, and protein bands were visualized on X‐ray films (Fujifilm). Quantification of immunoblots was determined in ImageJ by applying the “Gel Analysis” method. Data represent mean ± SD from three biological replicates. Statistical significance was calculated using a one‐tailed, unpaired Student's *t*‐test. *P*‐value < 0.05 was considered significant (*P < 0.05, **P < 0.01, ***P < 0.001, ****P < 0.0001). All samples as well as loading controls originating from a single replicate were processed on the same blot.

### Immunoprecipitations and pulldowns

For mass spectrometry (MS), 4 × 15‐cm dishes of 293T SMCR8 KO C9orf72‐HA cells were treated with Btz (1 μM) or DMSO for 8 h, and cell pellets were lysed in MCLB buffer (50 mM Tris–HCl pH 7.4, 150 mM NaCl, 0.5% NP‐40) supplemented with protease and phosphatase inhibitors for 30 min on ice. Following centrifugation at 20,000 *g*, 4°C for 10 min, supernatants were filtered through a 0.45 μm PVDF membrane (Merck Millipore), and protein concentration was adjusted by BCA assay. Prepared lysates were incubated with equilibrated anti‐HA beads (Sigma) overnight while rotating at 4°C. After washing 5× in MCLB buffer and 5× in PBS, elution was achieved by incubating beads with HA‐peptide for 30 min at RT and samples were further processed in‐solution for MS (see below). For immunoblotting, 2 × 10 cm dishes of 293T SMCR8 KO or parental C9orf72‐HA cells were used as input. Immunoprecipitations were performed as described above with the exception that after binding, anti‐HA beads were washed 5× in MCLB buffer and elution was obtained by boiling samples in 3× sample buffer for 5 min at 95°C. For Streptavidin pulldowns, Streptavidin agarose (Sigma) was loaded with GFP VHH biotinylated nanobodies (Chromotek) in MCLB buffer for 1 h at 4°C while rotating. After washing 3× in dilution buffer, beads were incubated with lysates prepared in MCLB buffer overnight at 4°C and then washed 5× in MCLB buffer. For USP2 treatment, beads were washed 3× in MCLB buffer, 1× in reaction buffer (50 mM Tris–HCl pH 8, 10 mM NaCl, 0.01% NP‐40, 0.5 mM DTT), then treated with recombinant USP2 (500 nM) in reaction buffer for 1 h at 30°C, and washed again 3× with MCLB buffer. All samples were boiled in 3× sample buffer for 5 min at 95°C.

### Denaturing immunoprecipitations

Cell pellets from 2 × 10 cm dishes of 239T SMCR8 KO C9orf72‐HA or eGFP‐C9orf72 were lysed in denaturation buffer (50 mM Tris–HCl pH 8, 150 mM NaCl, 0.5 mM DTT, 0.5 mM PMSF, 10 mM NEM, 0.5% NP‐40, 1% SDS, Benzonase) supplemented with protease and phosphatase inhibitors and incubated for 10 min on ice. Lysates were diluted 6x with dilution buffer (50 mM Tris–HCl pH 8, 300 mM NaCl, 10 mM NEM, 0.5% NP‐40) supplemented with protease and phosphatase inhibitors and sonicated at an amplitude of 50% for 10 s (1 s sonication and 1 s break in turns). Following centrifugation at 20,000 *g*, 4°C for 10 min, protein concentration was adjusted by BCA assay. Lysates were loaded on HA‐agarose or Streptavidin agarose coupled with biotinylated GFP VHH nanobodies overnight at 4°C. For deubiquitination by USP2, beads from the Btz‐treated sample were divided into two fractions after overnight incubation. Beads were washed 3× in dilution buffer, 1× in reaction buffer (50 mM Tris–HCl pH 8, 10 mM NaCl, 0.01% NP‐40, 0.5 mM DTT), and then treated with recombinant USP2 (500 nM) in reaction buffer for 1 h at 30°C. Samples without USP2 were washed 4× in dilution buffer and incubated in dilution buffer for 1 h at 30°C. Elution was attained by adding 3× sample buffer and boiling at 95°C for 5 min. For analysis of C9orf72 ubiquitination status by MS, 293T SMCR8 KO C9orf72‐HA cells were treated with Btz (1 μM) or DMSO for 8 h and subjected to denaturing immunoprecipitation as described above. Following elution in 3× sample buffer, eluates were separated by SDS–PAGE prior to in‐gel tryptic digestion (see below).

### Mass spectrometry sample preparation

Following HA‐immunoprecipitation, proteins were precipitated with 20% TCA and incubated for 20 min on ice. After centrifugation at 20,000 *g*, 4°C for 30 min, the supernatant was discarded, 10% cold TCA was added to pellets, and centrifuged again. Pellets were washed 3× in cold acetone, centrifuged, and then dried in a speed vacuum concentrator. For in‐solution tryptic digestion, pellets were resolved in 50 mM ammonium bicarbonate (ABC) pH 8.0 with 10% acetonitrile (ACN). 0.5 μg trypsin was added and proteins were digested for 4 h at 37°C. Reaction was stopped by adding 5% formic acid in 5% ACN for 10 min at RT. Peptides were dried and reconstituted in 1% trifluoroacetic acid (TFA) in 5% ACN. For in‐gel tryptic digestion, eluted proteins were separated on a polyacrylamide gel and the gel was cut into small gel pieces (1 × 1 mm). Gel pieces were washed 3× in 50 mM ABC with 50% ethanol for each 15 min at RT, dehydrated by incubating 2× in absolute ethanol, and dried using the speed vacuum concentrator. Proteins were reduced by incubating gel pieces in 10 mM DTT in 50 mM ABC for 45 min at 55°C and then alkylated by adding 55 mM iodoacetamide in 50 mM ABC for 30 min at RT. Gel pieces were washed in 50 mM ABC for 15 min at RT and dehydrated in absolute ethanol for 15 min at RT twice in turns. Following the last step of dehydration with ethanol, gel pieces were dried completely. 0.5 μg trypsin in 50 mM ABC was incubated on gel pieces overnight at 37°C. Peptides were extracted by incubating gel pieces with 3% TFA in 30% ACN, 2× 70% ACN and 2× 100% ACN. All elution steps were conducted for 20 min at RT and eluates were collected after every step. Peptide eluates were evaporated to approximately 1/5 of the original volume and mixed 1:1 with 1% TFA in 5% ACN. Desalting of peptides was accomplished on custom‐made C18 stage tips and peptides were reconstituted in 0.1% formic acid for MS analysis.

### Mass spectrometry data collection

Following reconstitution in 0.1% formic acid, samples were loaded on a 75 μm × 15 cm fused silica capillary (custom‐made) packed with C18Q resin (Reprosil‐PUR 120, 1.9 μm, Dr. Maisch) and separated using EASY nLC 1,200 liquid chromatography (Thermo Scientific). For the C9orf72 interactome, peptides were separated using a 35 min ACN gradient in 0.1% formic acid at a flowrate of 400 nl/min (10–38% ACN for 23 min, 38–60% ACN for 2 min and 60–100% ACN for 5 min). For the C9orf72 ubiquitination status, peptide separation was performed with a 60 min ACN gradient in 0.1% formic acid at a flowrate of 400 nl/min (10–38% ACN for 35 min, 38–60% ACN for 5 min and 60–100% ACN for 10 min). Separated peptides were detected using Q Exactive HF mass spectrometer (Thermo Scientific).

### Mass spectrometry data analysis

MS data output was processed in MaxQuant (version 1.6.0.1; Cox & Mann, 2008) with the human Uniprot‐FASTA reference proteome (UP000005640) in a reversed decoy mode and a false discovery rate (FDR) of 0.01. For the C9orf72 interactome, four biological replicates with each including two technical replicates for every condition were analyzed using label‐free quantification (LFQ) and re‐quantification. MaxQuant protein groups file was further processed in Perseus (version 1.6.5.0; Tyanova *et al*, 2016). For basic filtering, reverse and site‐specific identifications as well as common contaminants were eliminated. Protein hits reaching a peptide count >1 in 3 out of 4 biological replicates were used for subsequent statistics. Statistical significance of proteins in Btz vs. DMSO condition was determined from log_2_ transformed LFQ intensities by the Student's *t*‐test setting FDR < 0.01 and minimal fold change s0 = 1. Pearson's correlations resulted from multi‐scatter plots after the log_2_ transformation of LFQ intensities. For the identification of the C9orf72 ubiquitination status, two technical replicates for each condition (MOCK, DMSO, Btz) were analyzed in MaxQuant using diGly as variable modifications. Lysine residues within C9orf72 were illustrated in PyMOL (version 2.5) using Protein Data Bank (PDB) identifier 6V4U (Su *et al*, 2021).

## Author contributions

**Julia Jülg:** Conceptualization; data curation; formal analysis; validation; investigation; visualization; methodology; writing – original draft; writing – review and editing. **Dieter Edbauer:** Resources; writing – review and editing. **Christian Behrends:** Conceptualization; resources; supervision; funding acquisition; writing – original draft; project administration; writing – review and editing.

## Disclosure and competing interests statement

The authors declare that they have no conflict of interest.

## Supporting information



Expanded View Figures PDF

Dataset EV1

Source Data for Expanded View

PDF+

Source Data for Figure 1

Source Data for Figure 2

Source Data for Figure 3

Source Data for Figure 4

Source Data for Figure 5

## Data Availability

The mass spectrometry proteomics data have been deposited to the ProteomeXchange Consortium (http://proteomecentral.proteomexchange.org) via the PRIDE partner repository with the dataset identifier PXD039887 (https://proteomecentral.proteomexchange.org/cgi/GetDataset?ID=PXD039887).
