# Supplementary material for: C9orf72 protein quality control by UBR5‐mediated heterotypic ubiquitin chains
Source: EMBO Rep. 2023 Jun 15;24(8):e55895. doi: 10.15252/embr.202255895 (PMC10398660; doi:10.15252/embr.202255895)
Supplement: Supplementary file 1 — Expanded View Figures PDF [file EMBR-24-e55895-s006.pdf]

# Expanded View Figures

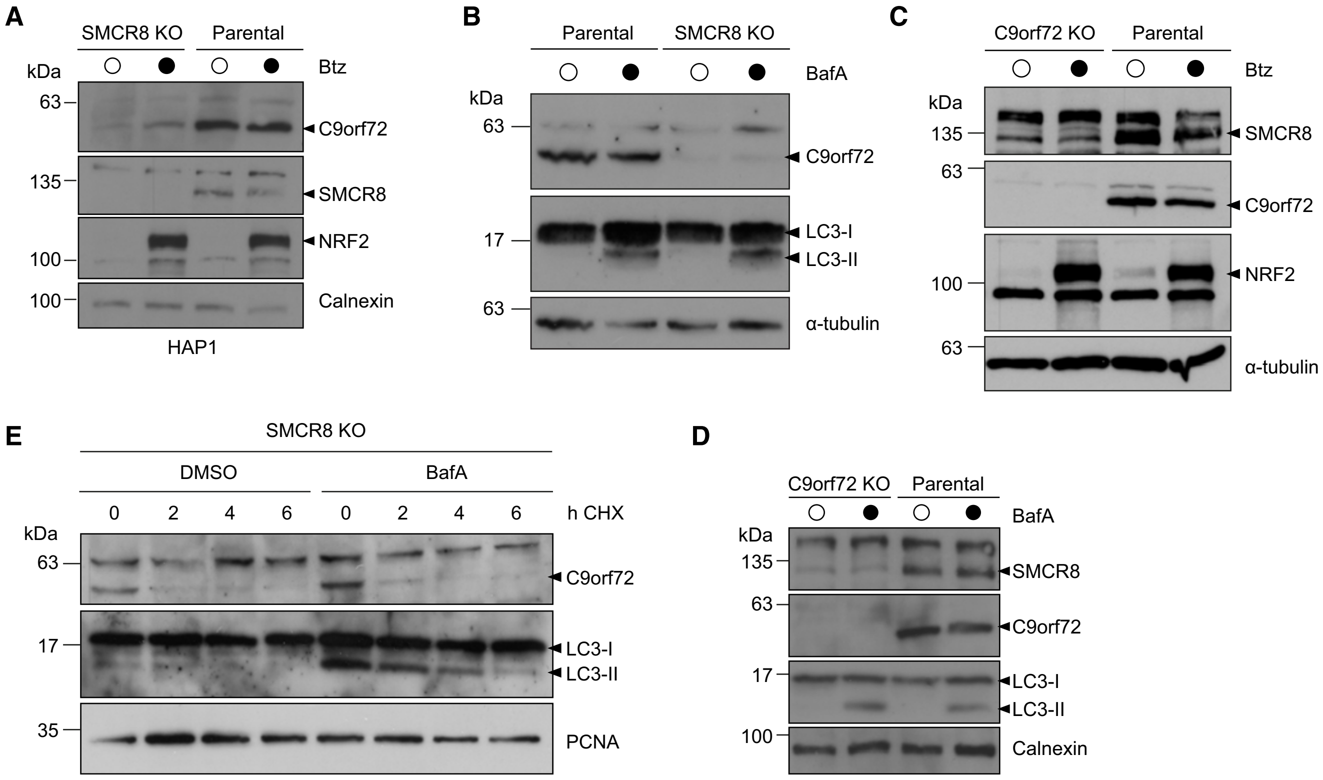


**Figure EV1. Differential regulation of C9orf72 and SMCR8 protein levels.**

A HAP1 parental and SMCR8 KO cells were treated with DMSO or Btz and analyzed by immunoblotting with indicated antibodies.
B 293T parental and SMCR8 KO cells were treated with DMSO or Bafilomycin A (BafA) and analyzed by Western blot.
C 293T parental and C9orf72 KO cells were treated with DMSO or Btz followed by lysis and immunoblotting.
D 293T parental and C9orf72 KO cells were treated with DMSO or BafA followed by Western blot analysis.
E 293T SMCR8 KO cells were subjected to a cycloheximide (CHX) chase in the absence or presence of DMSO or BafA and analyzed by immunoblotting.

Data information: NFR2 confirmed proteasomal inhibition by Btz. LC3 confirmed the inhibition of autophagosome-lysosome fusion by BafA. Calnexin, PCNA, and α-tubulin served as loading controls.
Source data are available online for this figure.

© 2023 The Authors

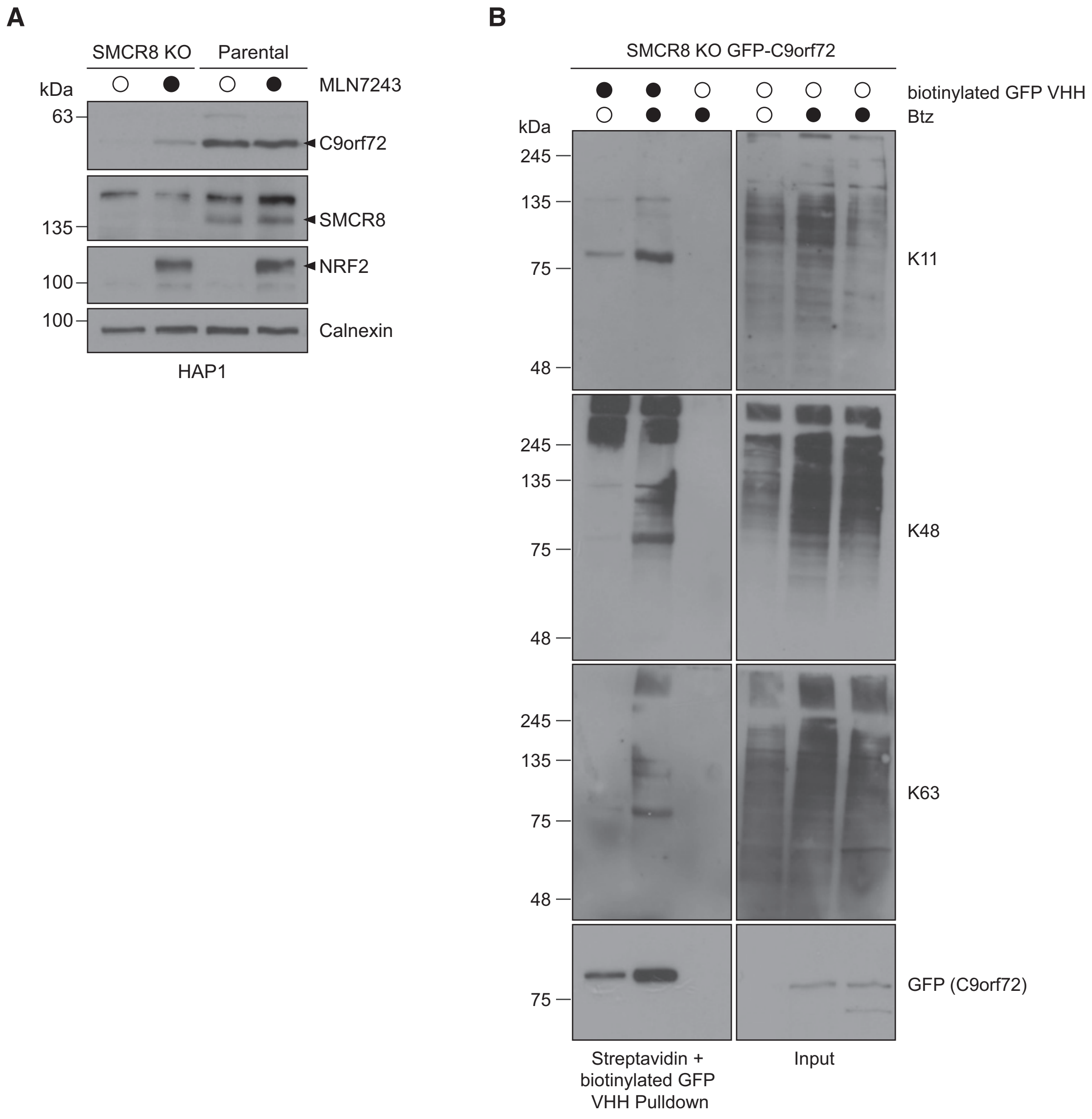


**Figure EV2. C9orf72 levels upon inhibition of the ubiquitination machinery.**

A HAP1 parental or SMCR8 KO cells were treated with DMSO or MLN7243 and analyzed by Western blot.

B SMCR8 KO cells stably expressing GFP-C9orf72 were treated with DMSO or Btz followed by lysis under denaturing conditions. Lysates were incubated with biotinylated GFP VHH nanobodies coupled with Streptavidin agarose and analyzed by immunoblotting.

Source data are available online for this figure.


© 2023 The Authors

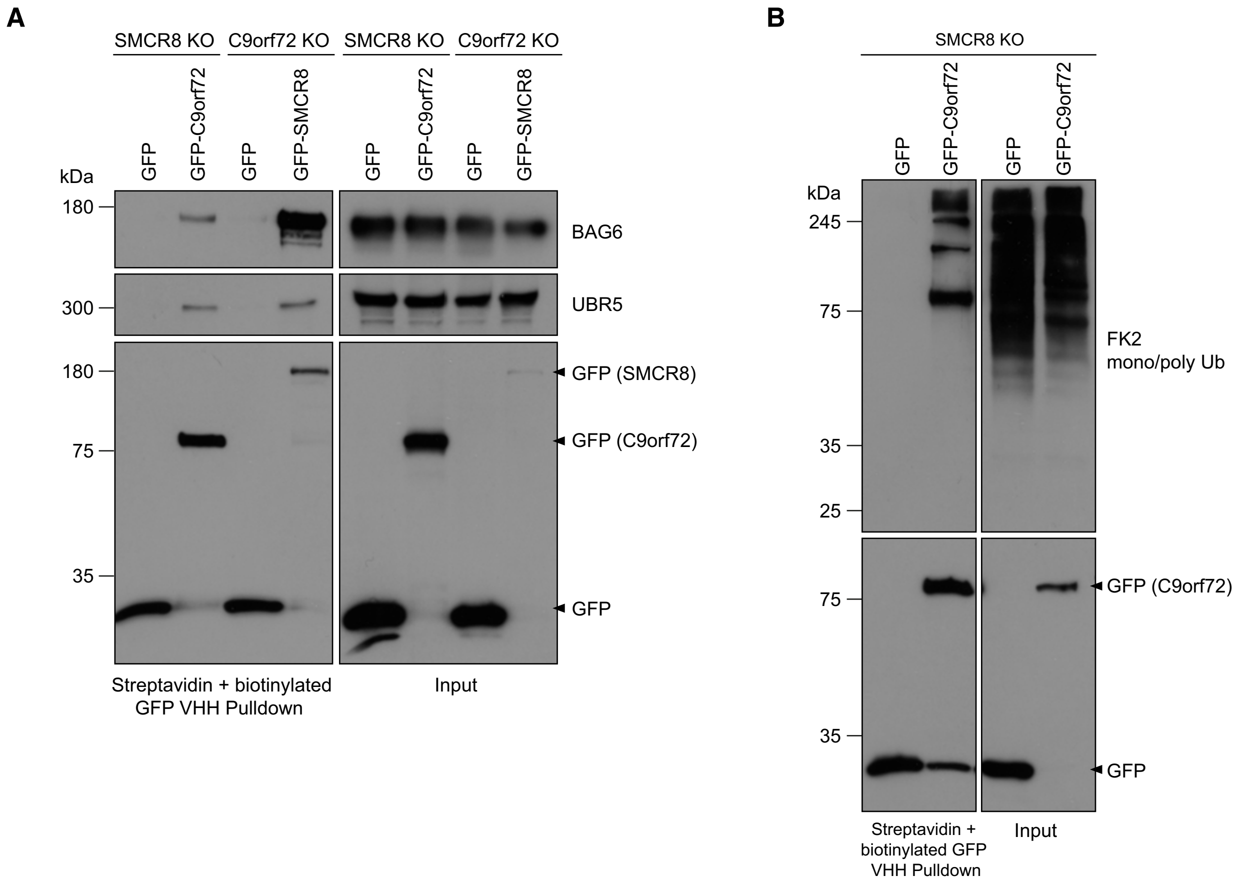


**Figure EV3. Interaction of BAG6 and UBR5 with uncomplexed C9orf72 and SMCR8.**

A 293T SMCR8 KO and C9orf72 KO cells were transfected with GFP-C9orf72 and GFP-SMCR8, respectively. GFP served as control. Cells were lysed under mild conditions, lysates were incubated with biotinylated GFP VHH coupled with Streptavidin agarose and analyzed by Western blot.

B SMCR8 KO cells stably expressing GFP or GFP-C9orf72 were treated with Btz and subjected to streptavidin pulldown using biotinylated GFP VHH nanobodies under denaturing conditions.

Source data are available online for this figure.

© 2023 The Authors
