# Supplementary figures and images for: C9orf72 protein quality control by UBR5‐mediated heterotypic ubiquitin chains (part 1 of 2)
Source: EMBO Rep. 2023 Jun 15;24(8):e55895. doi: 10.15252/embr.202255895 (PMC10398660; doi:10.15252/embr.202255895)

Figure 1A

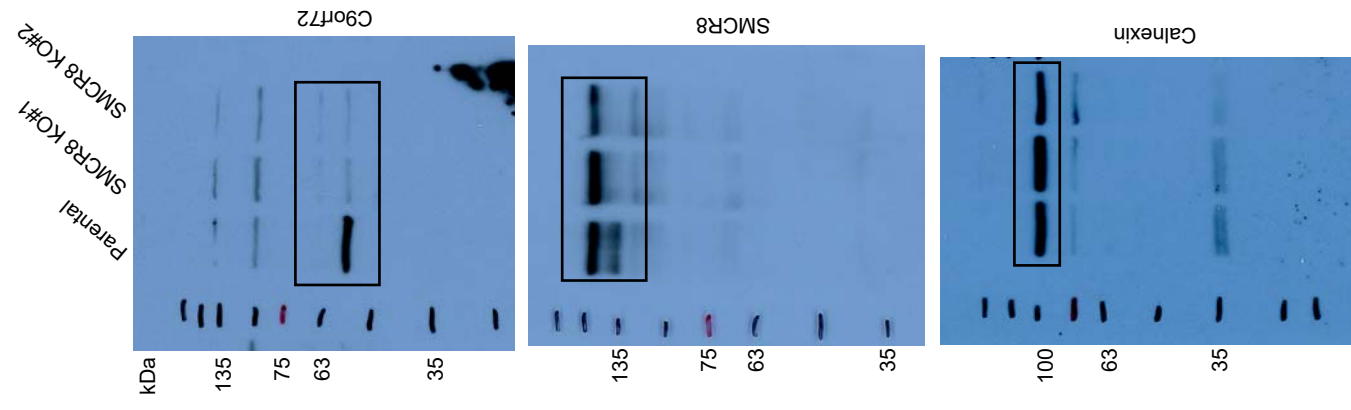

Figure 1B

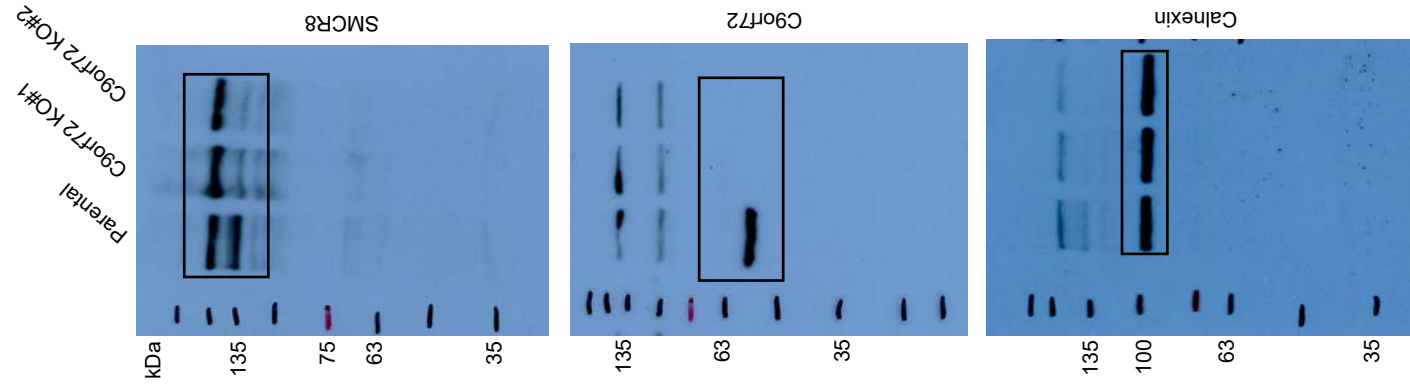

Figure 1C

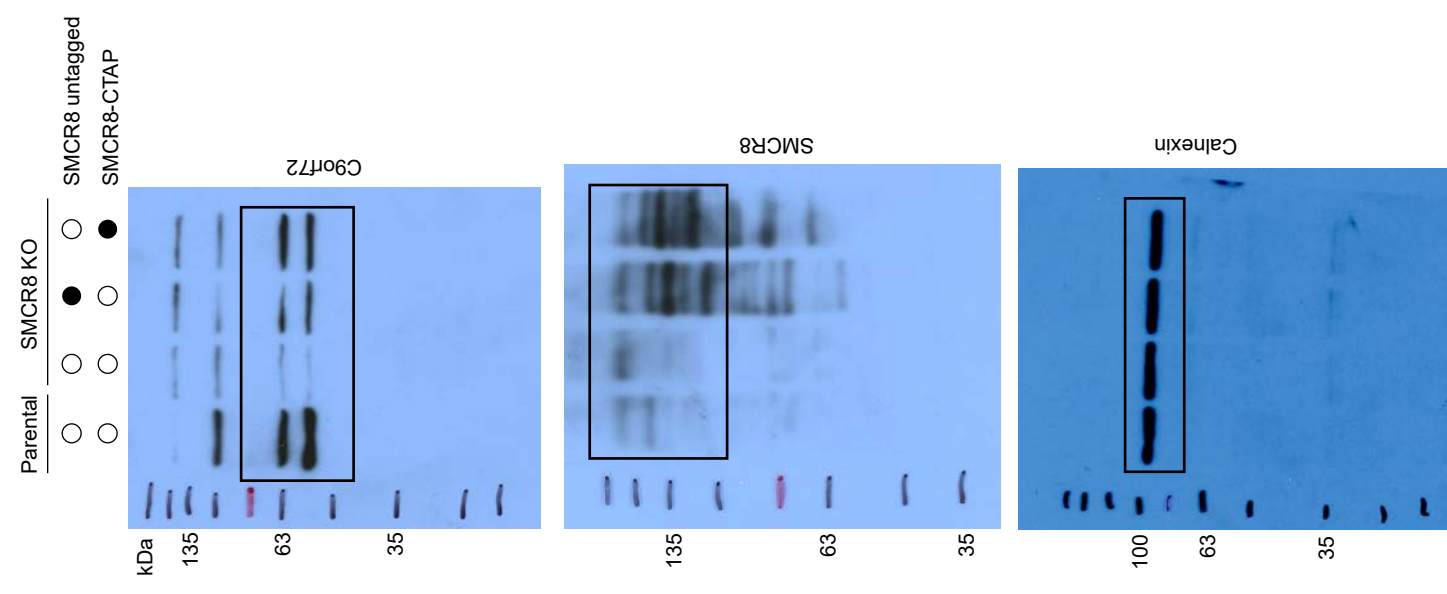

Figure 1G

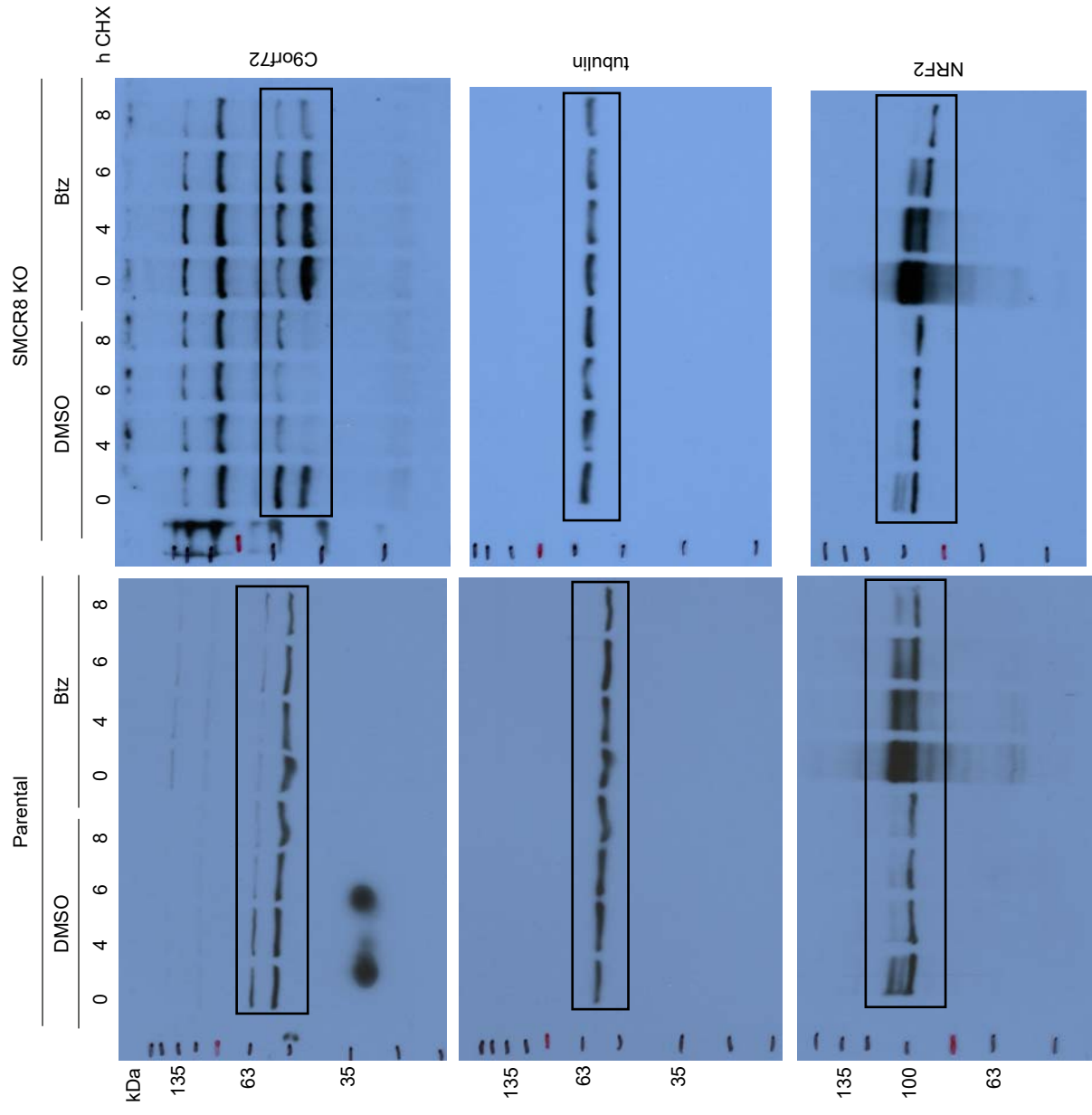

Figure 1D

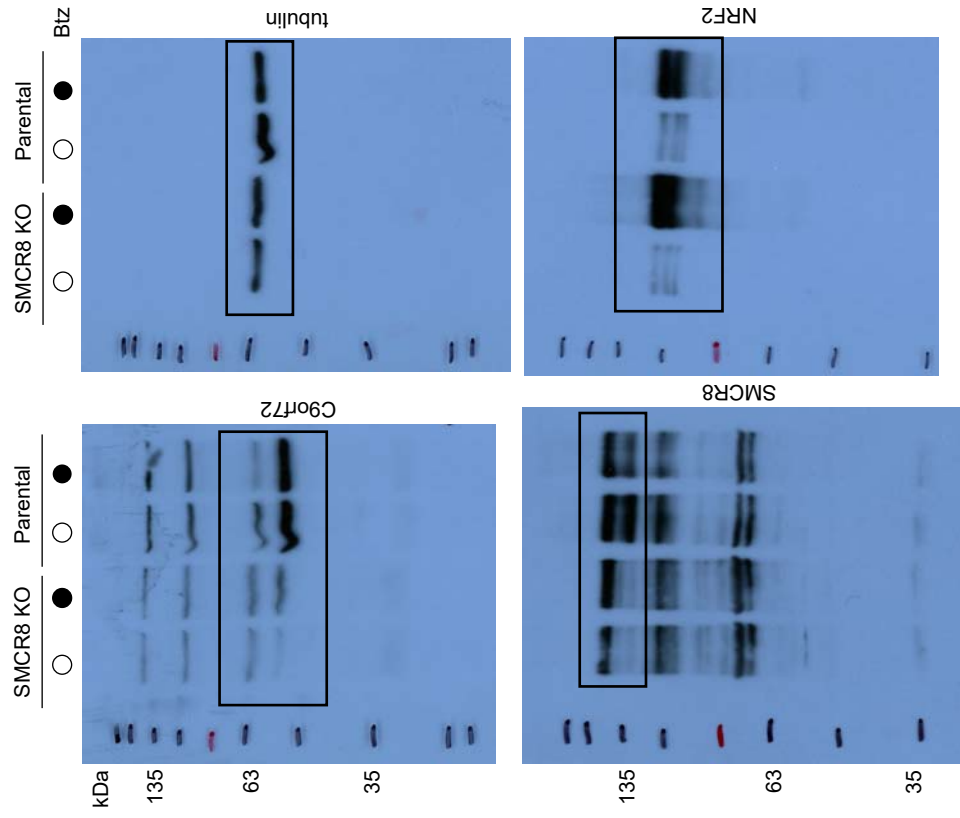

Supplement: Supplementary file 5 — Source Data for Figure 1 [file EMBR-24-e55895-s007.zip › Figure 1/Figure 1.pdf]

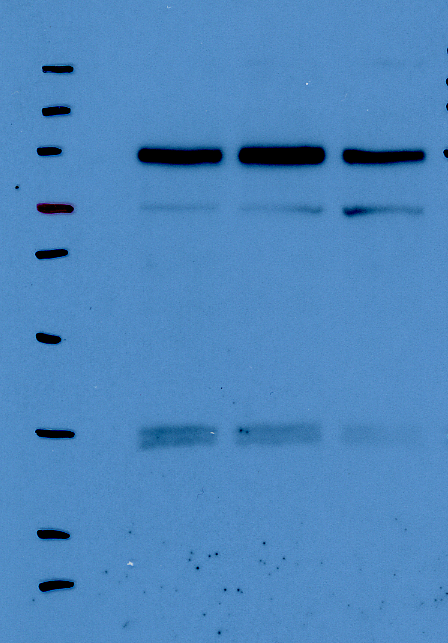

Supplement: Supplementary file 5 — Source Data for Figure 1 [file EMBR-24-e55895-s007.zip › Figure 1/1a/1a_Calnexin.tif]

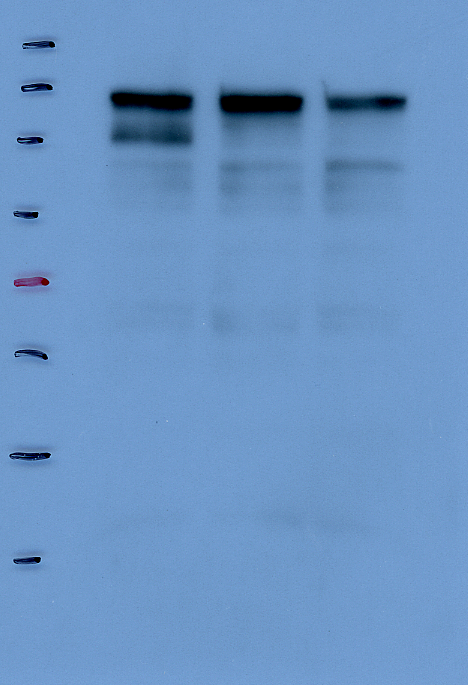

Supplement: Supplementary file 5 — Source Data for Figure 1 [file EMBR-24-e55895-s007.zip › Figure 1/1a/1a_SMCR8.tif]

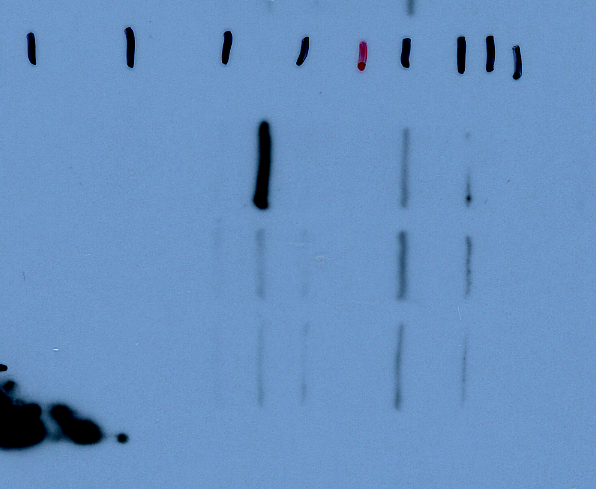

Supplement: Supplementary file 5 — Source Data for Figure 1 [file EMBR-24-e55895-s007.zip › Figure 1/1a/1a_C9orf72.tif]

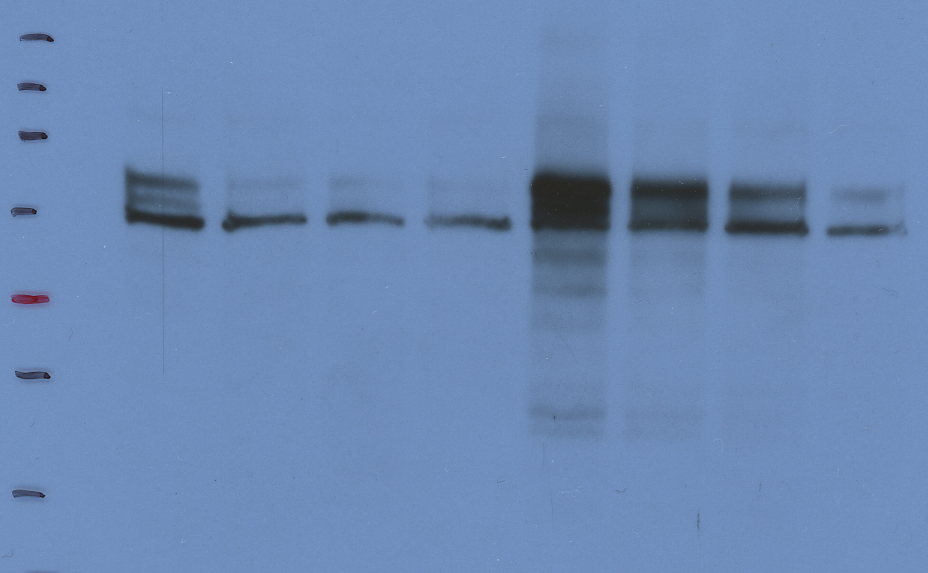

Supplement: Supplementary file 5 — Source Data for Figure 1 [file EMBR-24-e55895-s007.zip › Figure 1/1g/1g_NRF2_parental.tif]

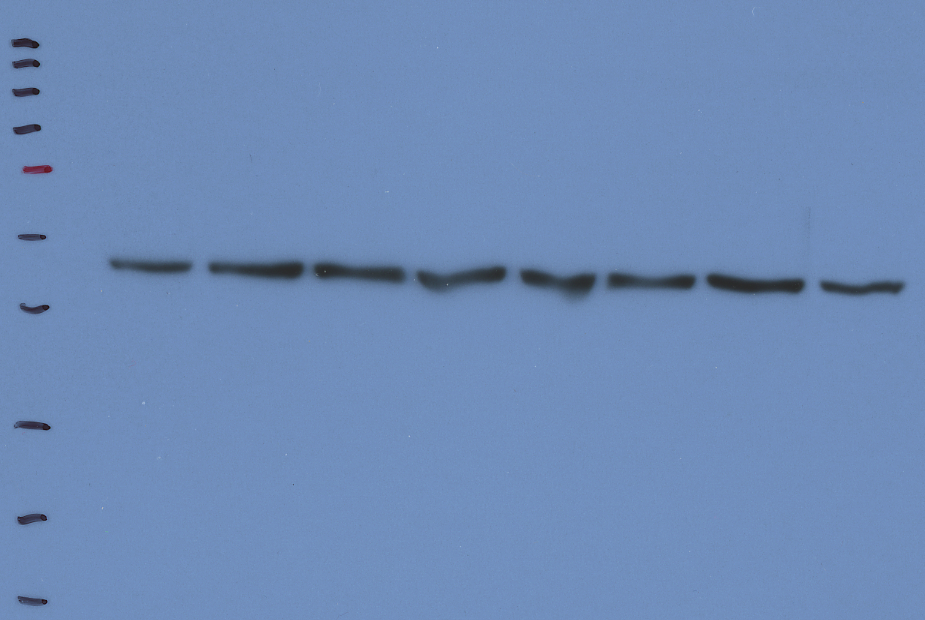

Supplement: Supplementary file 5 — Source Data for Figure 1 [file EMBR-24-e55895-s007.zip › Figure 1/1g/1g_tubulin_parental.tif]

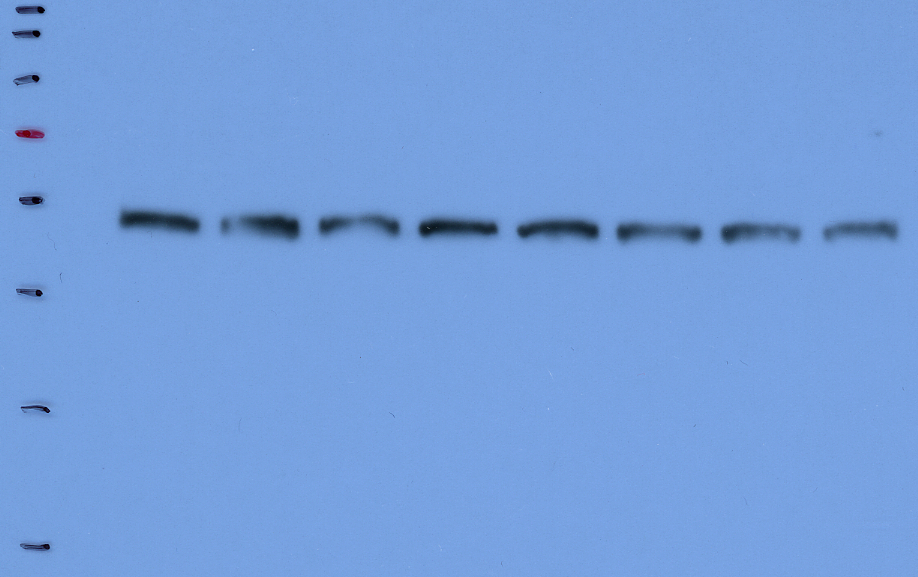

Supplement: Supplementary file 5 — Source Data for Figure 1 [file EMBR-24-e55895-s007.zip › Figure 1/1g/1g_tubulin_SMCR8KO.tif]

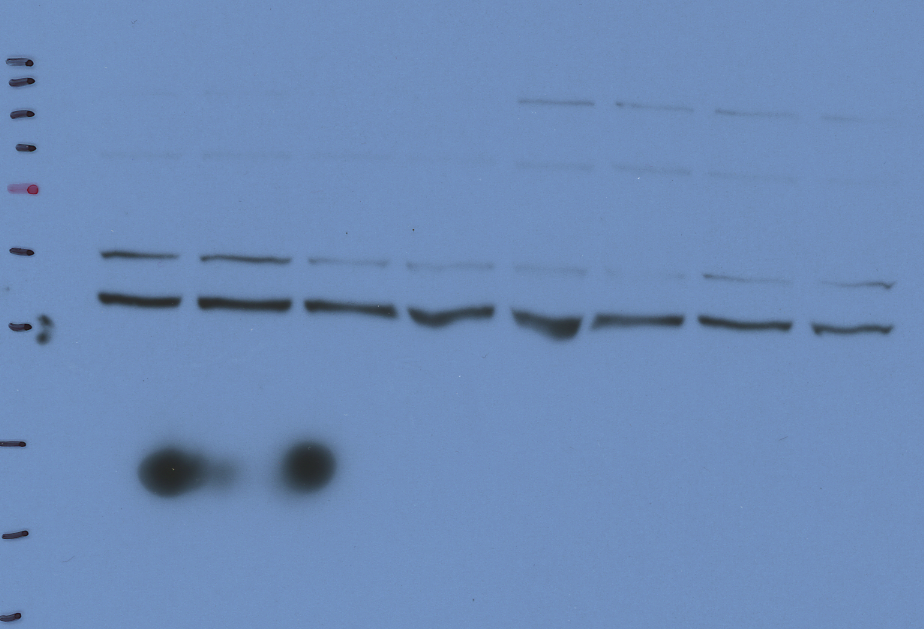

Supplement: Supplementary file 5 — Source Data for Figure 1 [file EMBR-24-e55895-s007.zip › Figure 1/1g/1g_C9orf72_parental.tif]

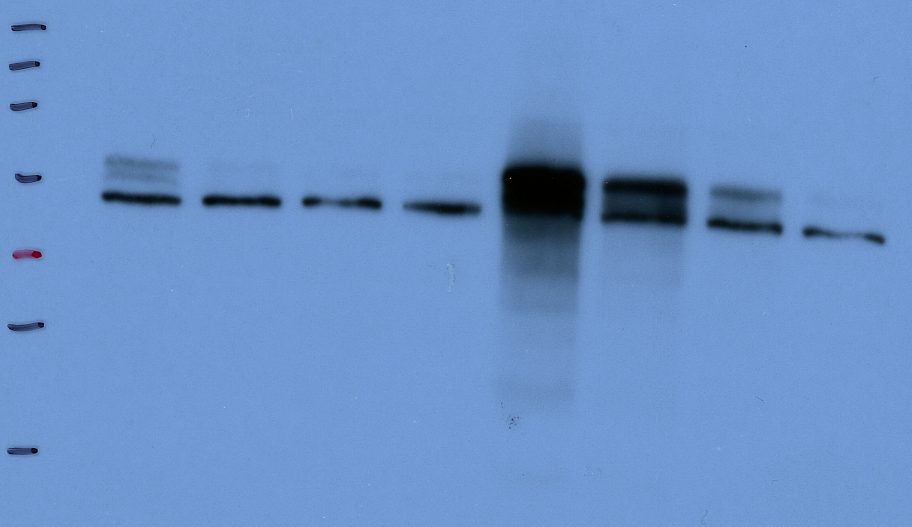

Supplement: Supplementary file 5 — Source Data for Figure 1 [file EMBR-24-e55895-s007.zip › Figure 1/1g/1g_NRF2_SMCR8KO.tif]

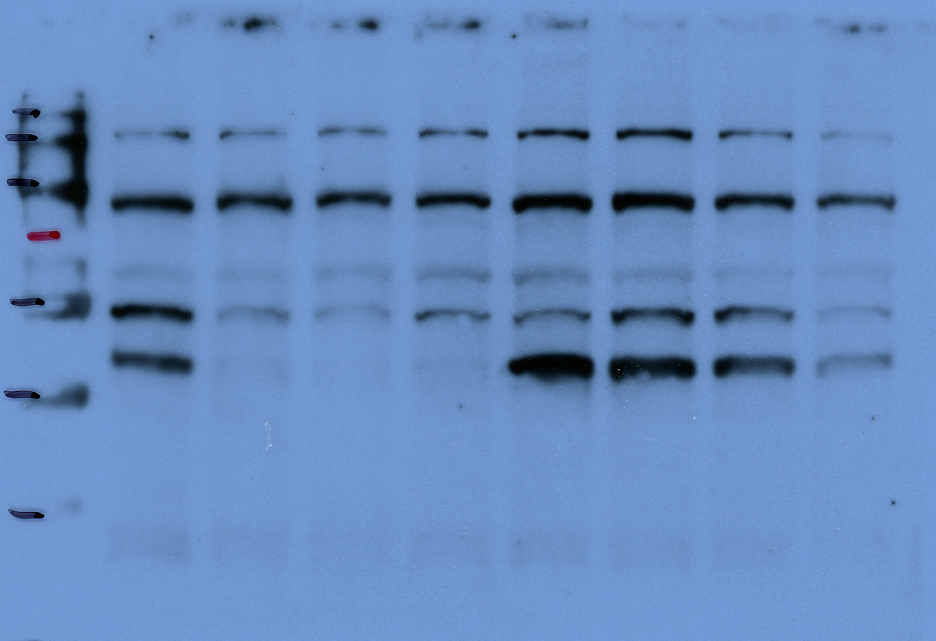

Supplement: Supplementary file 5 — Source Data for Figure 1 [file EMBR-24-e55895-s007.zip › Figure 1/1g/1g_C9orf72_SMCR8KO.tif]

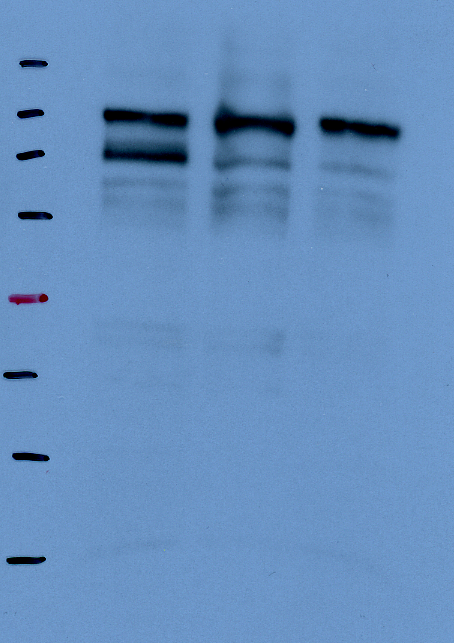

Supplement: Supplementary file 5 — Source Data for Figure 1 [file EMBR-24-e55895-s007.zip › Figure 1/1b/1b_SMCR8.tif]

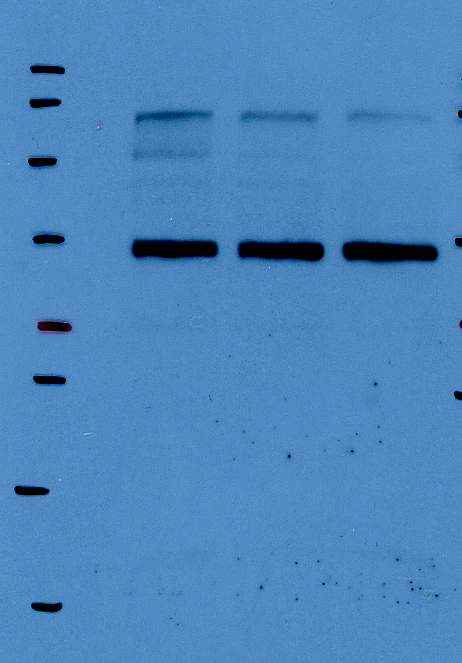

Supplement: Supplementary file 5 — Source Data for Figure 1 [file EMBR-24-e55895-s007.zip › Figure 1/1b/1b_Calnexin.tif]

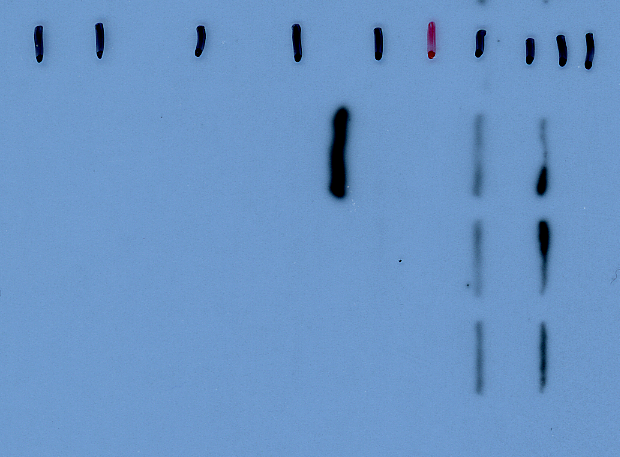

Supplement: Supplementary file 5 — Source Data for Figure 1 [file EMBR-24-e55895-s007.zip › Figure 1/1b/1b_C9orf72.tif]

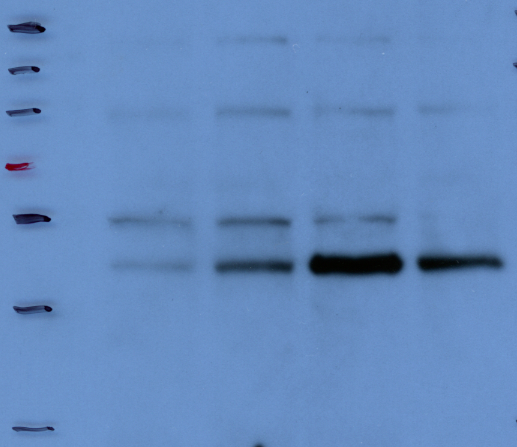

Supplement: Supplementary file 5 — Source Data for Figure 1 [file EMBR-24-e55895-s007.zip › Figure 1/1e/1e_C9orf72_n2.tif]

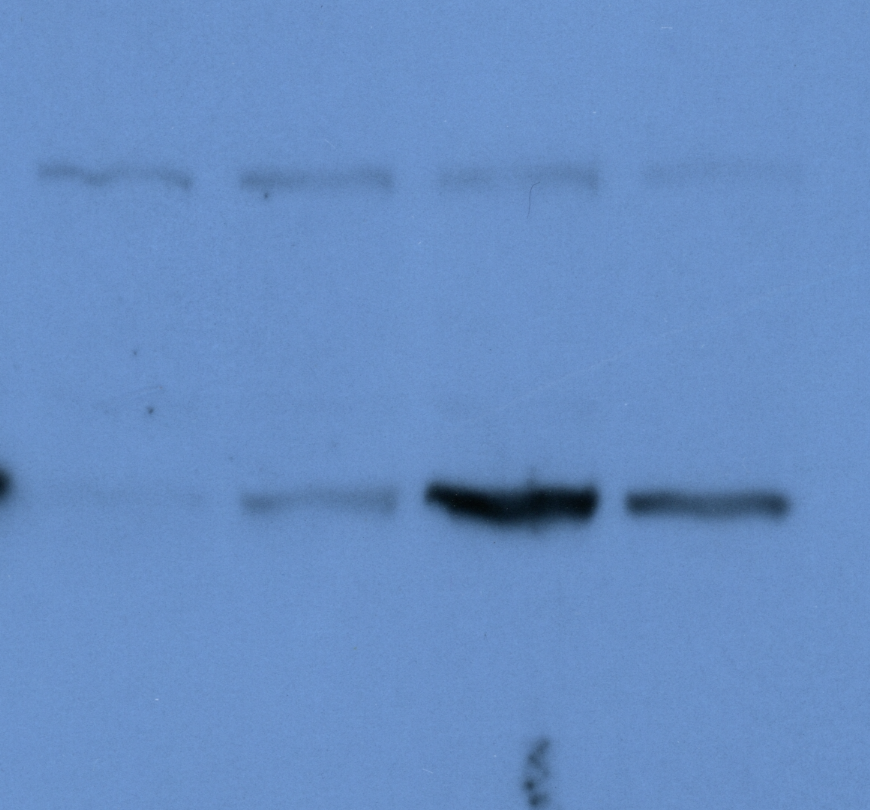

Supplement: Supplementary file 5 — Source Data for Figure 1 [file EMBR-24-e55895-s007.zip › Figure 1/1e/1e_C9orf72_n3.tif]

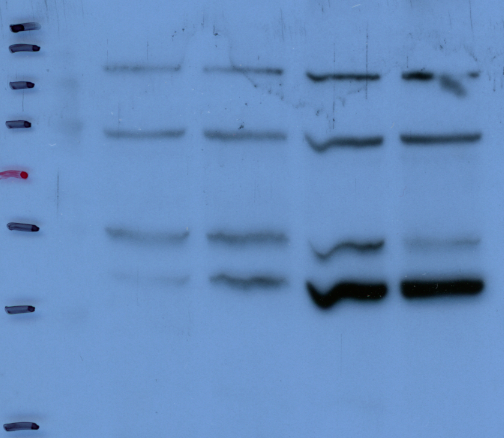

Supplement: Supplementary file 5 — Source Data for Figure 1 [file EMBR-24-e55895-s007.zip › Figure 1/1e/1e_C9orf72_n1.tif]

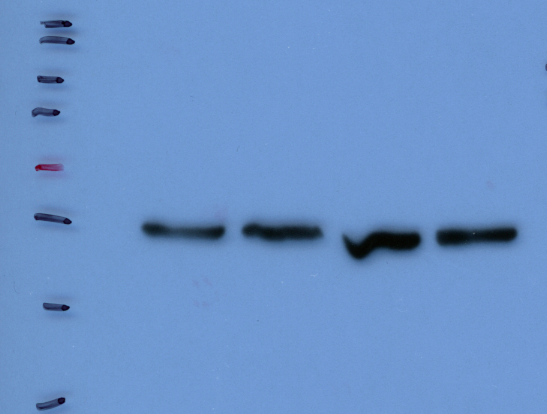

Supplement: Supplementary file 5 — Source Data for Figure 1 [file EMBR-24-e55895-s007.zip › Figure 1/1e/1e_tubulin_n1.tif]

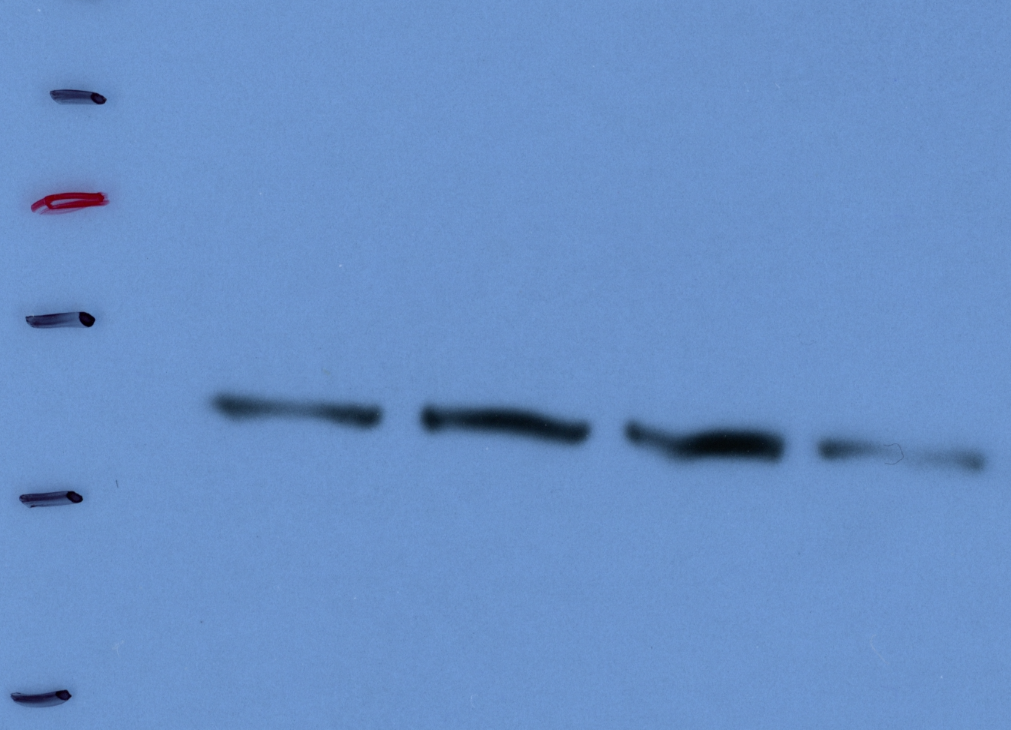

Supplement: Supplementary file 5 — Source Data for Figure 1 [file EMBR-24-e55895-s007.zip › Figure 1/1e/1e_tubulin_n3.tif]

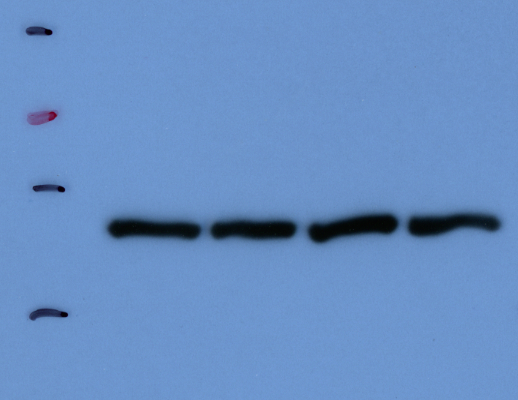

Supplement: Supplementary file 5 — Source Data for Figure 1 [file EMBR-24-e55895-s007.zip › Figure 1/1e/1e_tubulin_n2.tif]

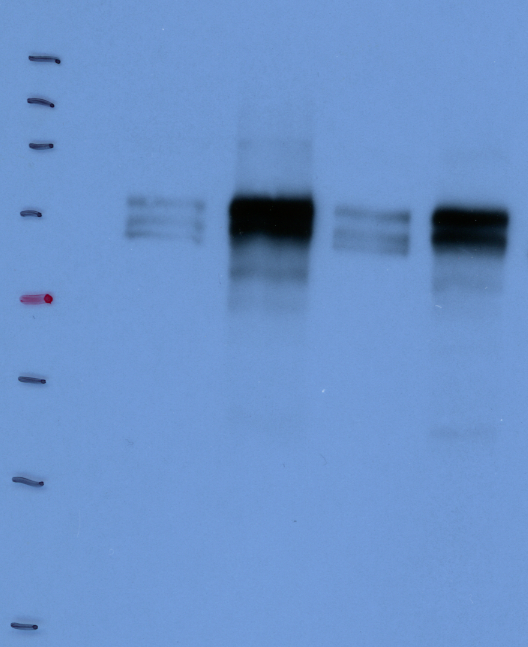

Supplement: Supplementary file 5 — Source Data for Figure 1 [file EMBR-24-e55895-s007.zip › Figure 1/1d/1d_NRF2.tif]

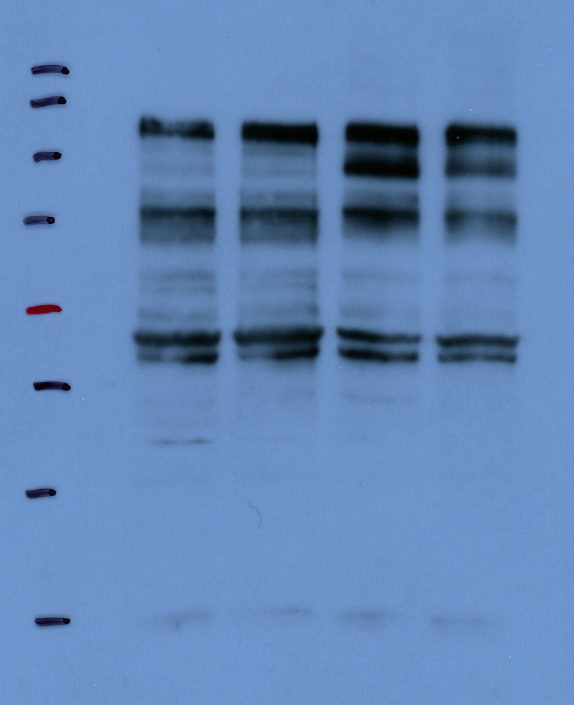

Supplement: Supplementary file 5 — Source Data for Figure 1 [file EMBR-24-e55895-s007.zip › Figure 1/1d/1d_SMCR8.tif]

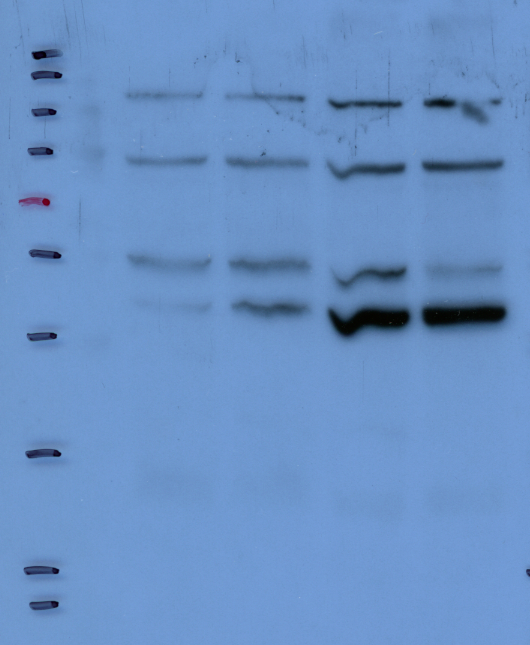

Supplement: Supplementary file 5 — Source Data for Figure 1 [file EMBR-24-e55895-s007.zip › Figure 1/1d/1d_C9orf72.tif]

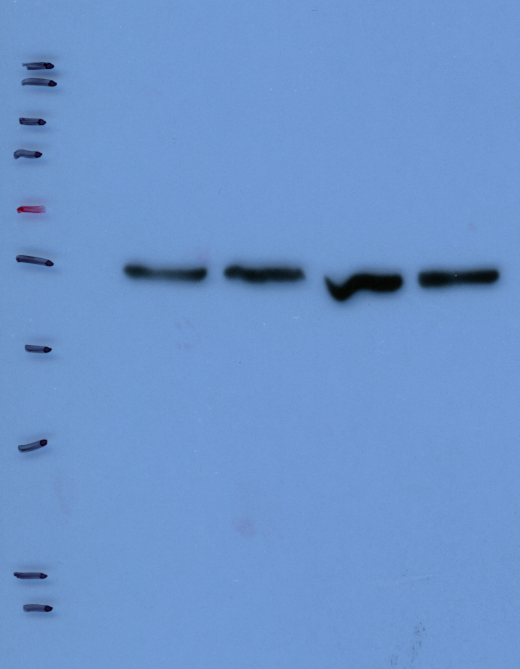

Supplement: Supplementary file 5 — Source Data for Figure 1 [file EMBR-24-e55895-s007.zip › Figure 1/1d/1d_tubulin.tif]

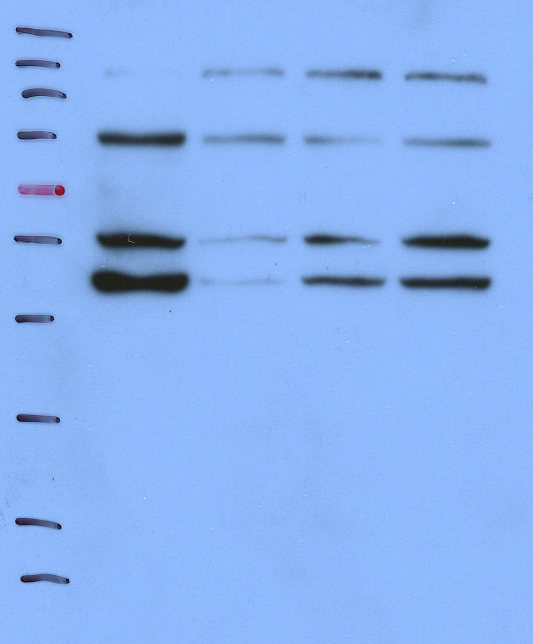

Supplement: Supplementary file 5 — Source Data for Figure 1 [file EMBR-24-e55895-s007.zip › Figure 1/1c/1c_C9orf72.tif]

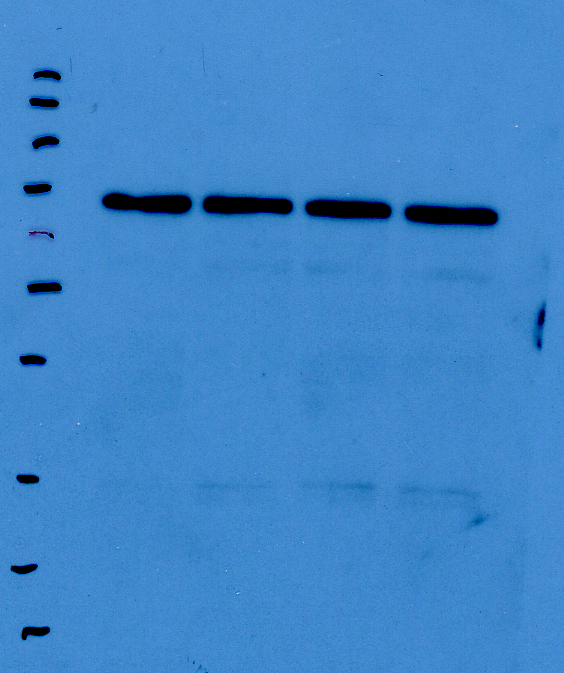

Supplement: Supplementary file 5 — Source Data for Figure 1 [file EMBR-24-e55895-s007.zip › Figure 1/1c/1c_Calnexin.tif]

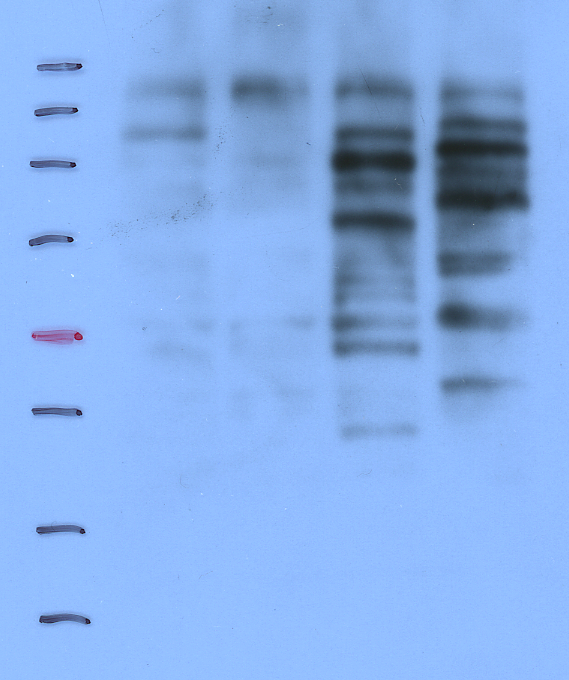

Supplement: Supplementary file 5 — Source Data for Figure 1 [file EMBR-24-e55895-s007.zip › Figure 1/1c/1c_SMCR8.tif]

Figure 2A

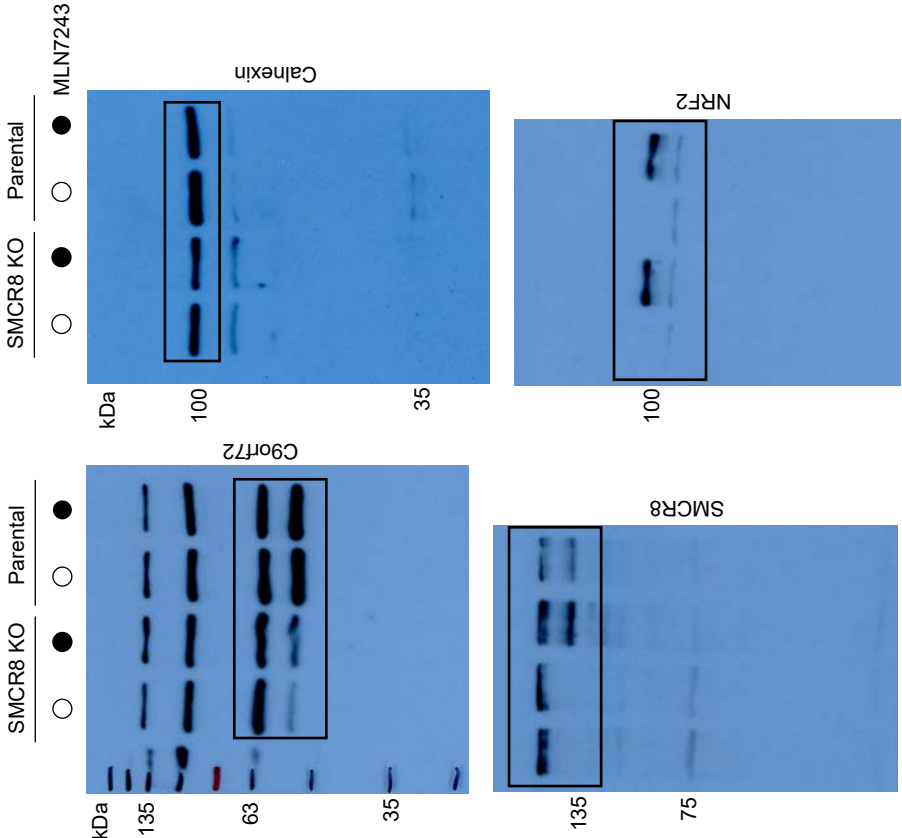

Figure 2D

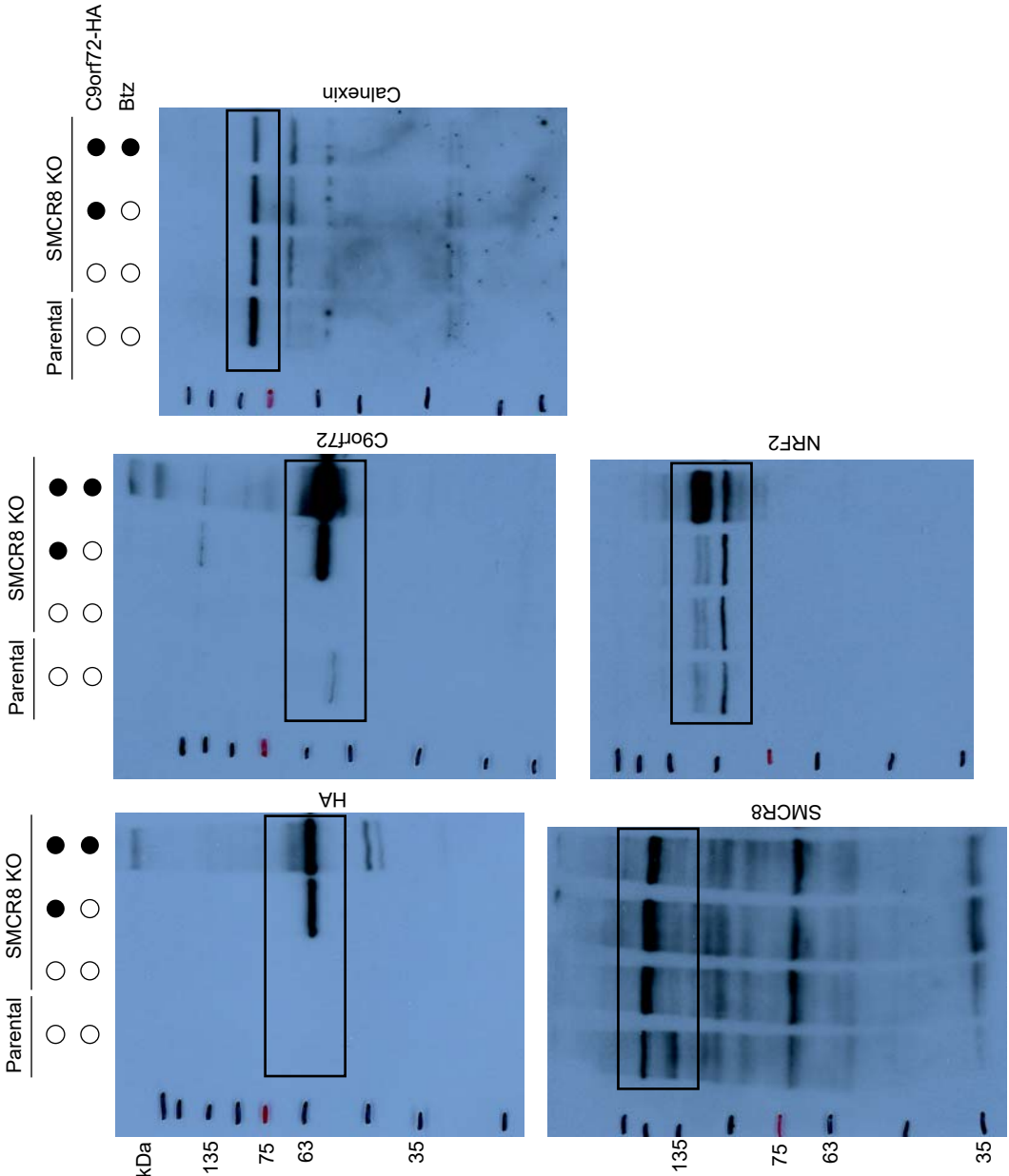

Figure 2E

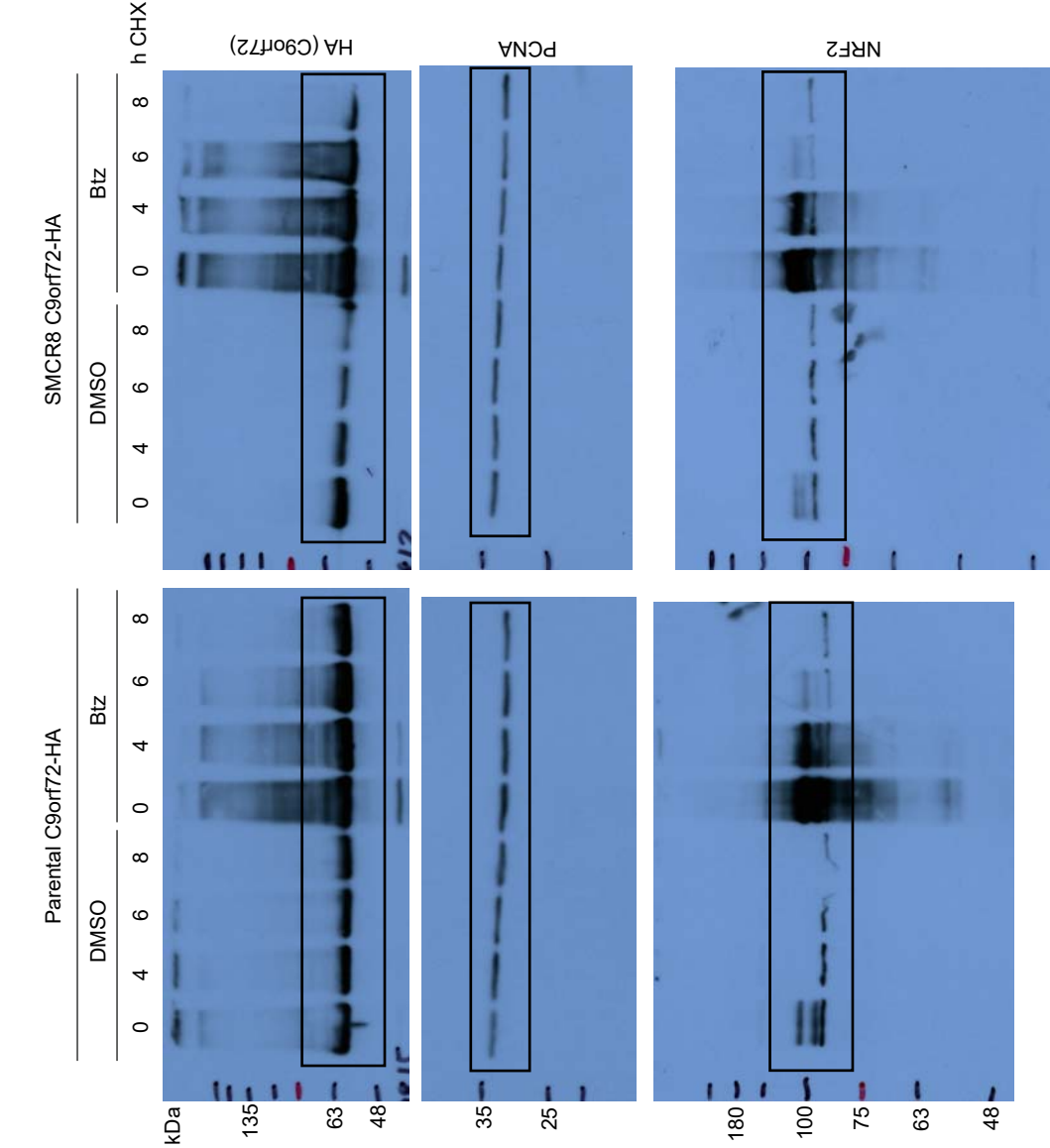

Figure 2F

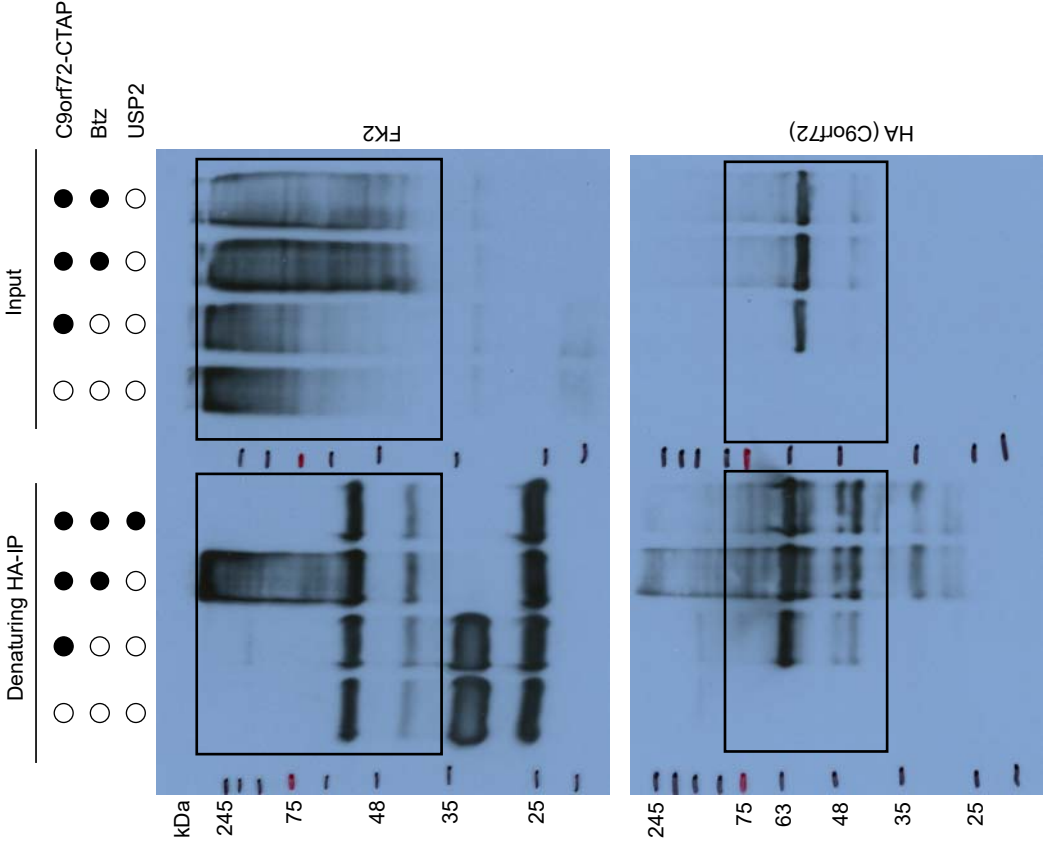

Supplement: Supplementary file 6 — Source Data for Figure 2 [file EMBR-24-e55895-s003.zip › Figure 2/Figure 2.pdf]

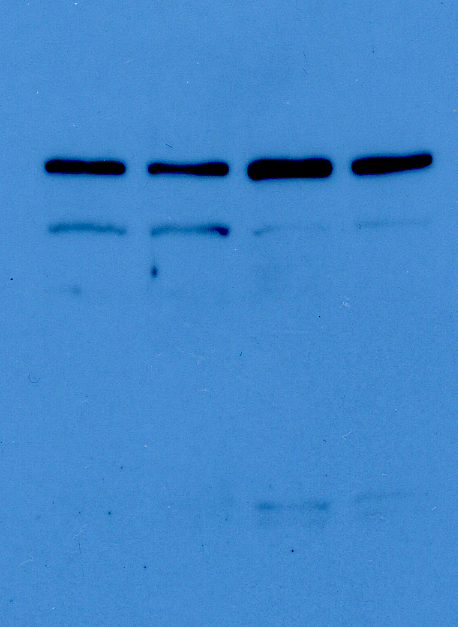

Supplement: Supplementary file 6 — Source Data for Figure 2 [file EMBR-24-e55895-s003.zip › Figure 2/2a/2a_Calnexin.tif]

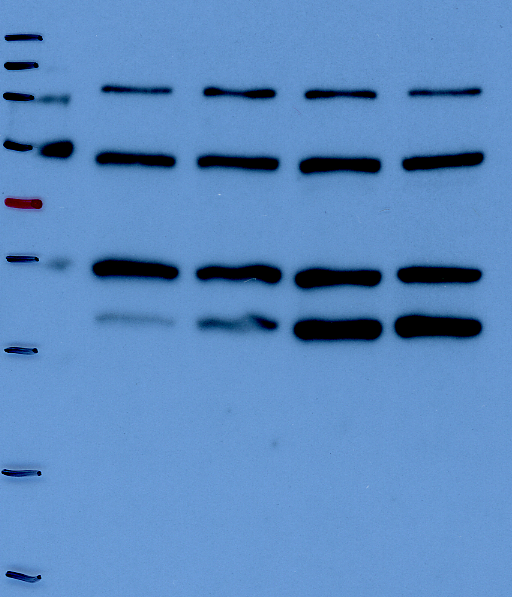

Supplement: Supplementary file 6 — Source Data for Figure 2 [file EMBR-24-e55895-s003.zip › Figure 2/2a/2a_C9orf72.tif]

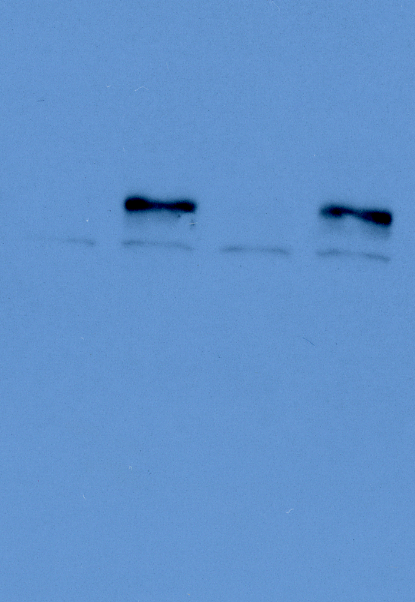

Supplement: Supplementary file 6 — Source Data for Figure 2 [file EMBR-24-e55895-s003.zip › Figure 2/2a/2a_NRF2.tif]

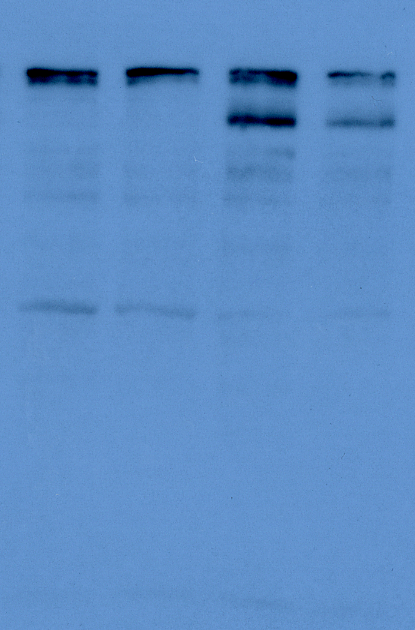

Supplement: Supplementary file 6 — Source Data for Figure 2 [file EMBR-24-e55895-s003.zip › Figure 2/2a/2a_SMCR8.tif]

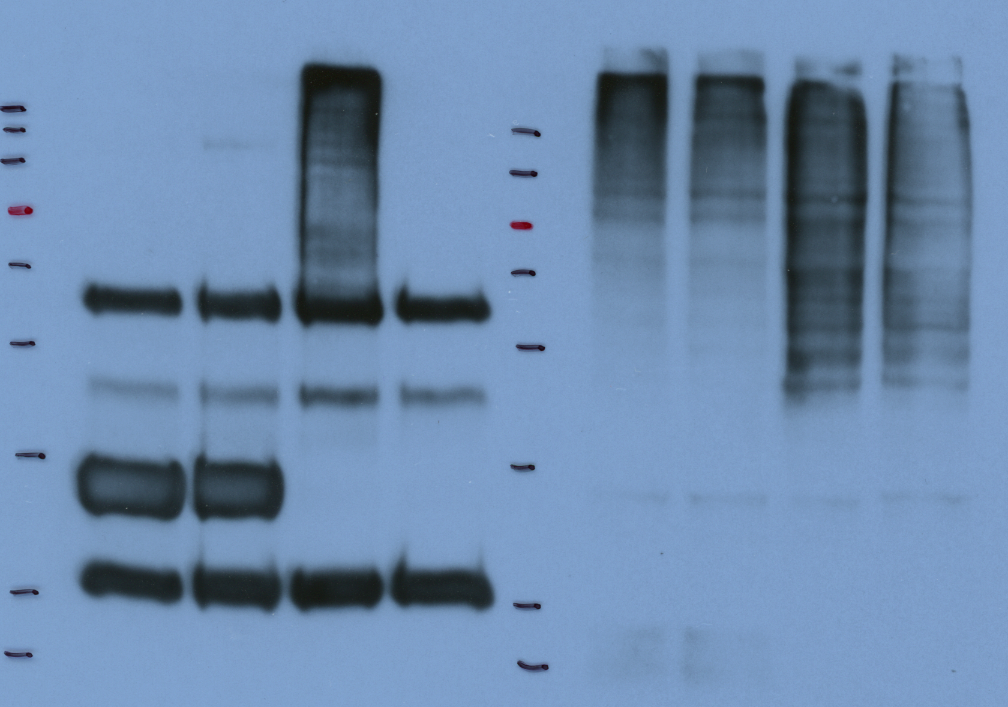

Supplement: Supplementary file 6 — Source Data for Figure 2 [file EMBR-24-e55895-s003.zip › Figure 2/2f/2f_FK2.tif]

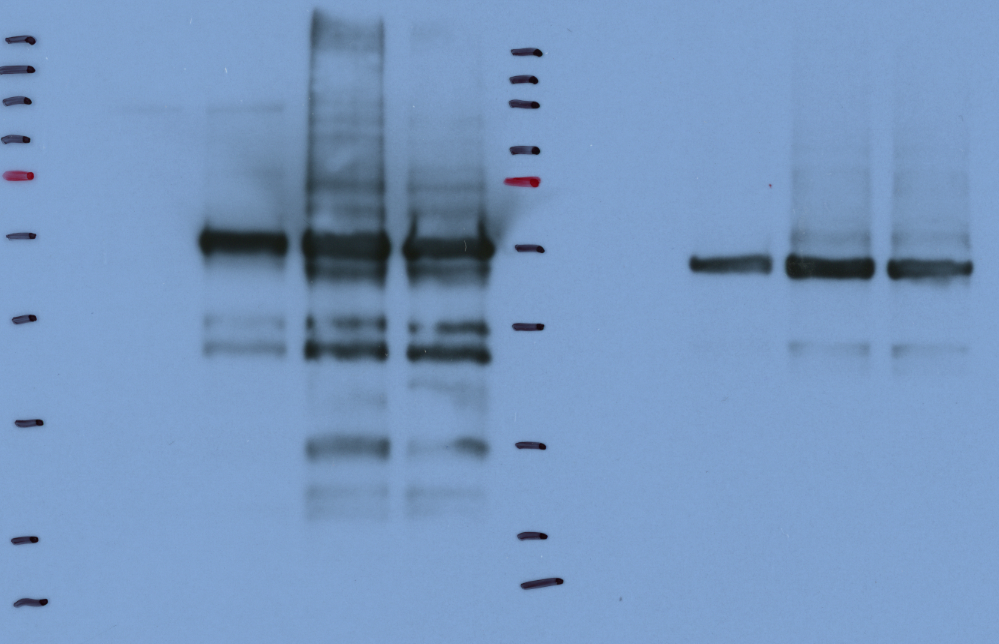

Supplement: Supplementary file 6 — Source Data for Figure 2 [file EMBR-24-e55895-s003.zip › Figure 2/2f/2f_HA.tif]

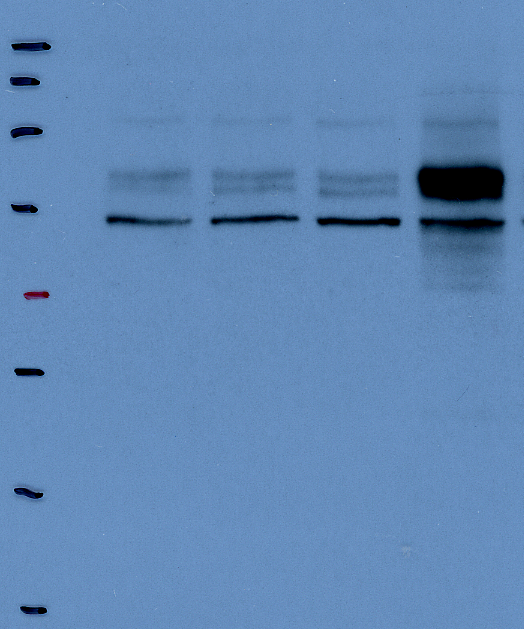

Supplement: Supplementary file 6 — Source Data for Figure 2 [file EMBR-24-e55895-s003.zip › Figure 2/2d/2d_NRF2.tif]

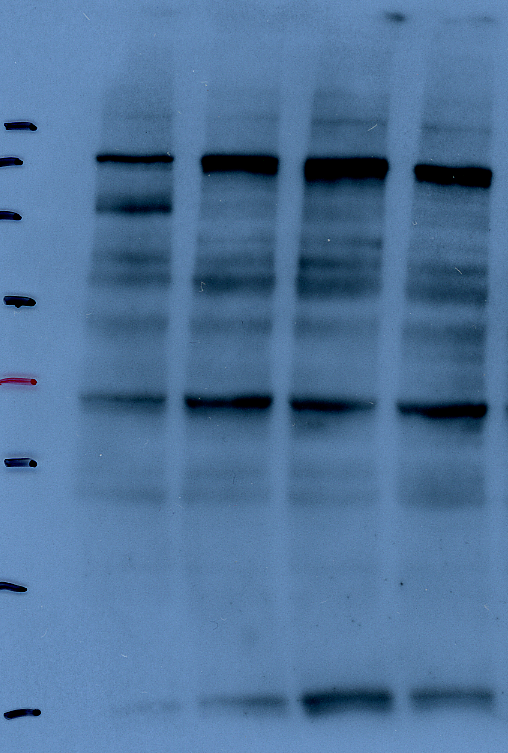

Supplement: Supplementary file 6 — Source Data for Figure 2 [file EMBR-24-e55895-s003.zip › Figure 2/2d/2d_SMCR8.tif]

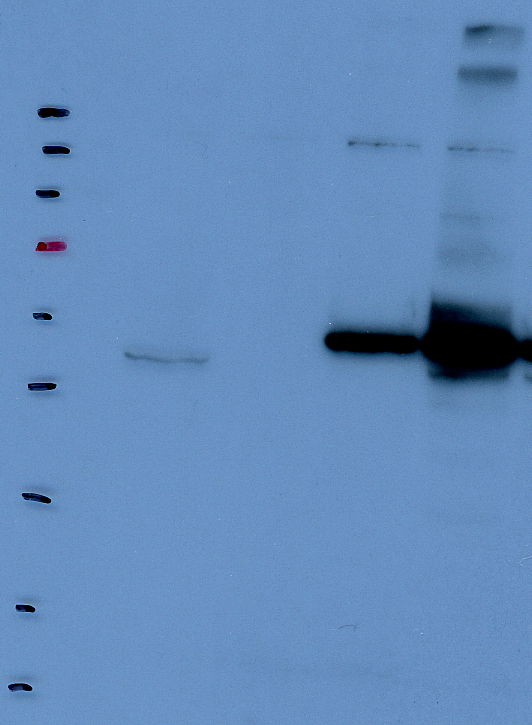

Supplement: Supplementary file 6 — Source Data for Figure 2 [file EMBR-24-e55895-s003.zip › Figure 2/2d/2d_C9orf72.tif]

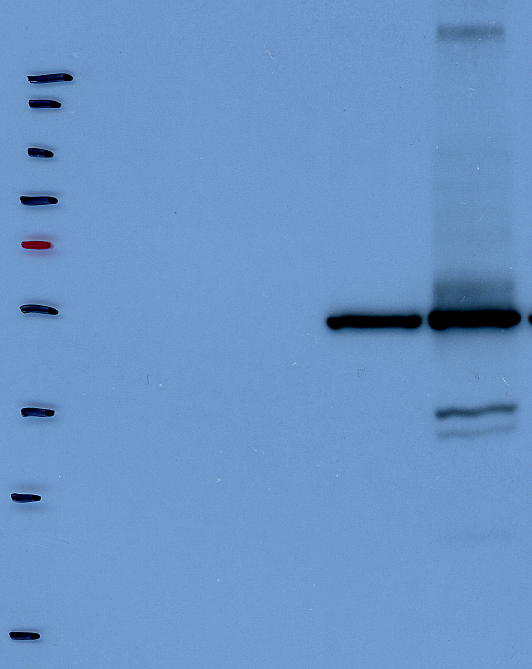

Supplement: Supplementary file 6 — Source Data for Figure 2 [file EMBR-24-e55895-s003.zip › Figure 2/2d/2d_HA.tif]

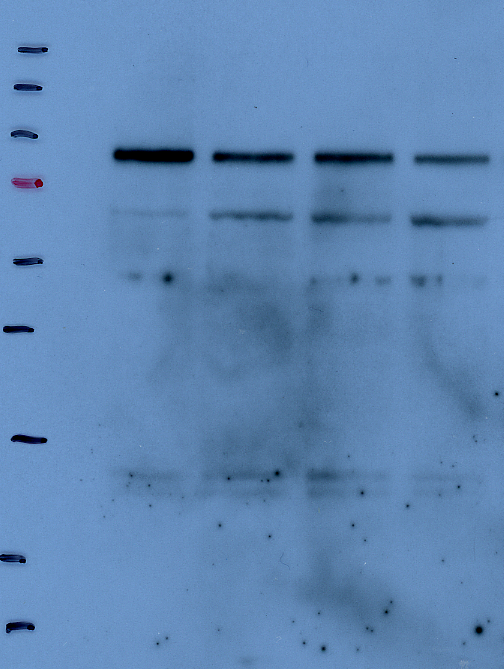

Supplement: Supplementary file 6 — Source Data for Figure 2 [file EMBR-24-e55895-s003.zip › Figure 2/2d/2d_Calnexin.tif]

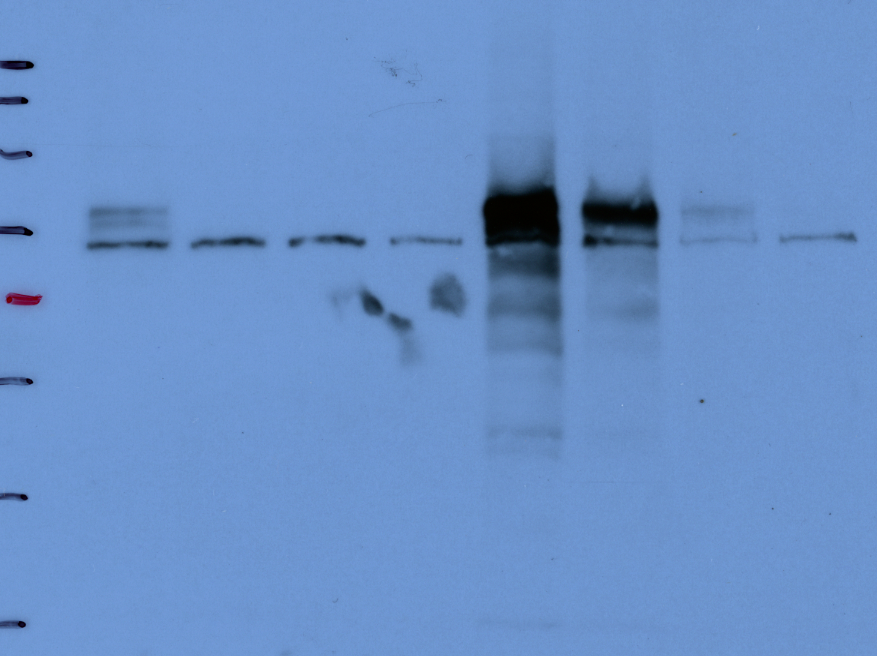

Supplement: Supplementary file 6 — Source Data for Figure 2 [file EMBR-24-e55895-s003.zip › Figure 2/2e/2e_NRF2_SMCR8KO.tif]

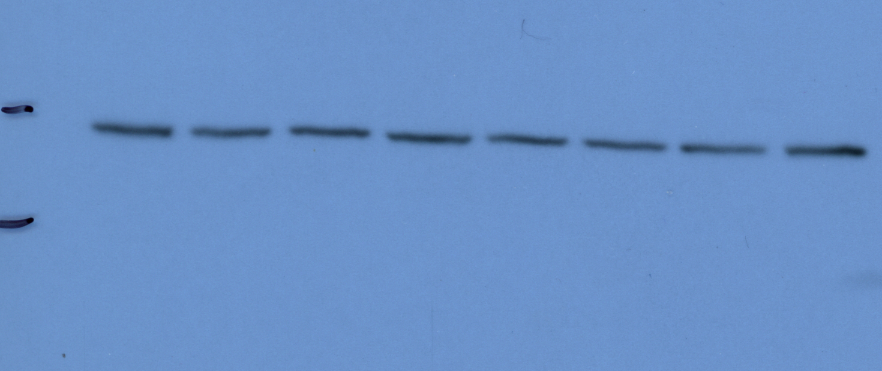

Supplement: Supplementary file 6 — Source Data for Figure 2 [file EMBR-24-e55895-s003.zip › Figure 2/2e/2e_PCNA_SMCR8.tif]

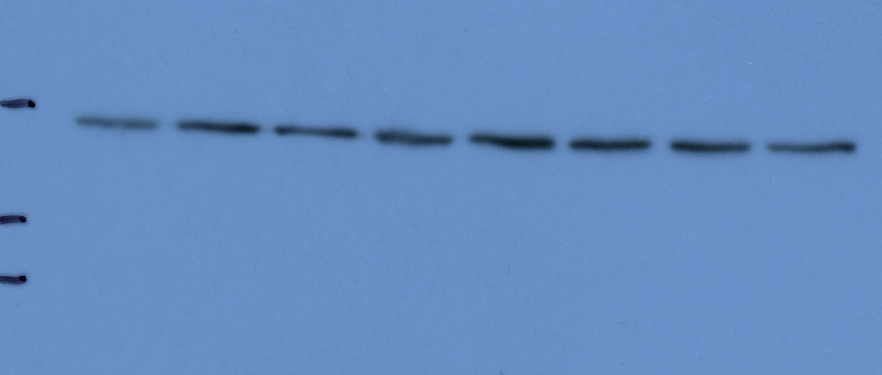

Supplement: Supplementary file 6 — Source Data for Figure 2 [file EMBR-24-e55895-s003.zip › Figure 2/2e/2e_PCNA_parental.tif]

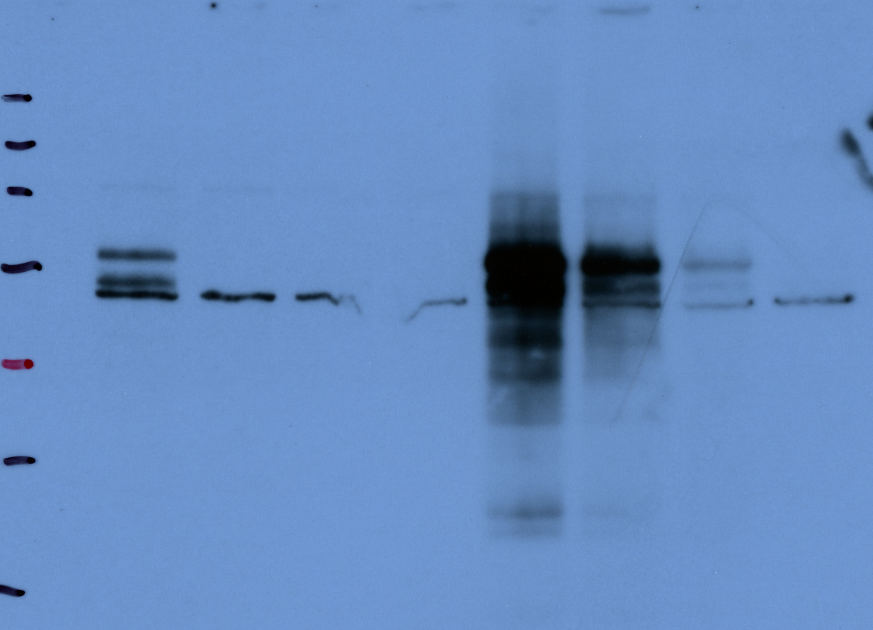

Supplement: Supplementary file 6 — Source Data for Figure 2 [file EMBR-24-e55895-s003.zip › Figure 2/2e/2e_NRF2_parental.tif]

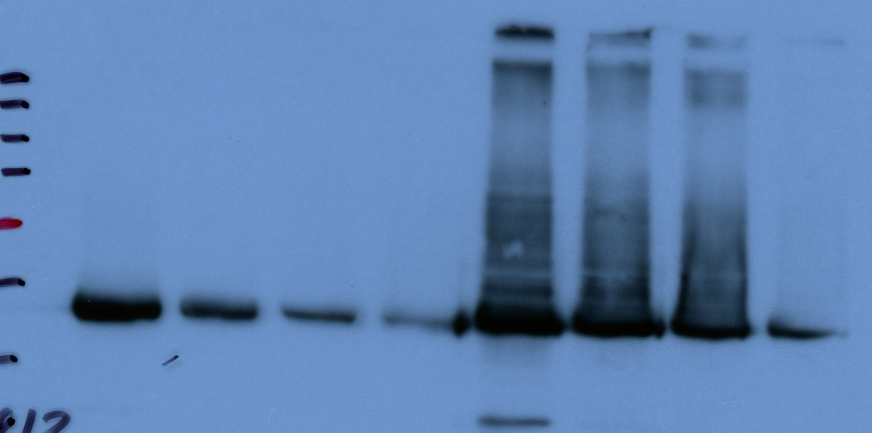

Supplement: Supplementary file 6 — Source Data for Figure 2 [file EMBR-24-e55895-s003.zip › Figure 2/2e/2e_HA_SMCR8.tif]

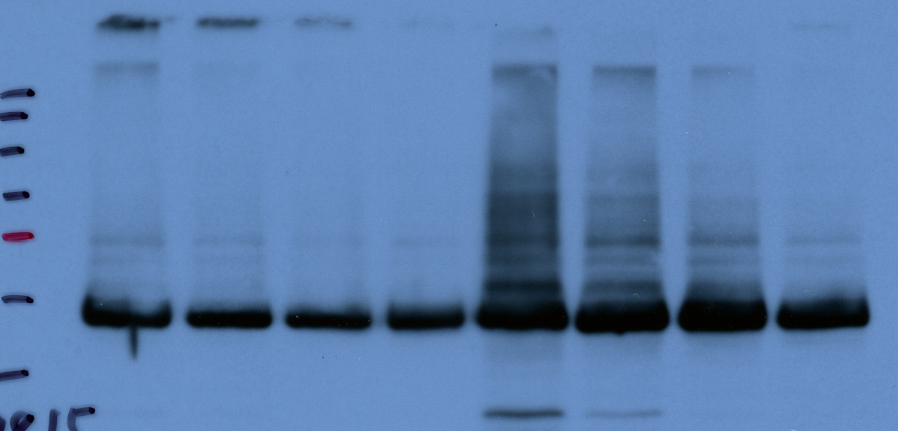

Supplement: Supplementary file 6 — Source Data for Figure 2 [file EMBR-24-e55895-s003.zip › Figure 2/2e/2e_HA_parental.tif]

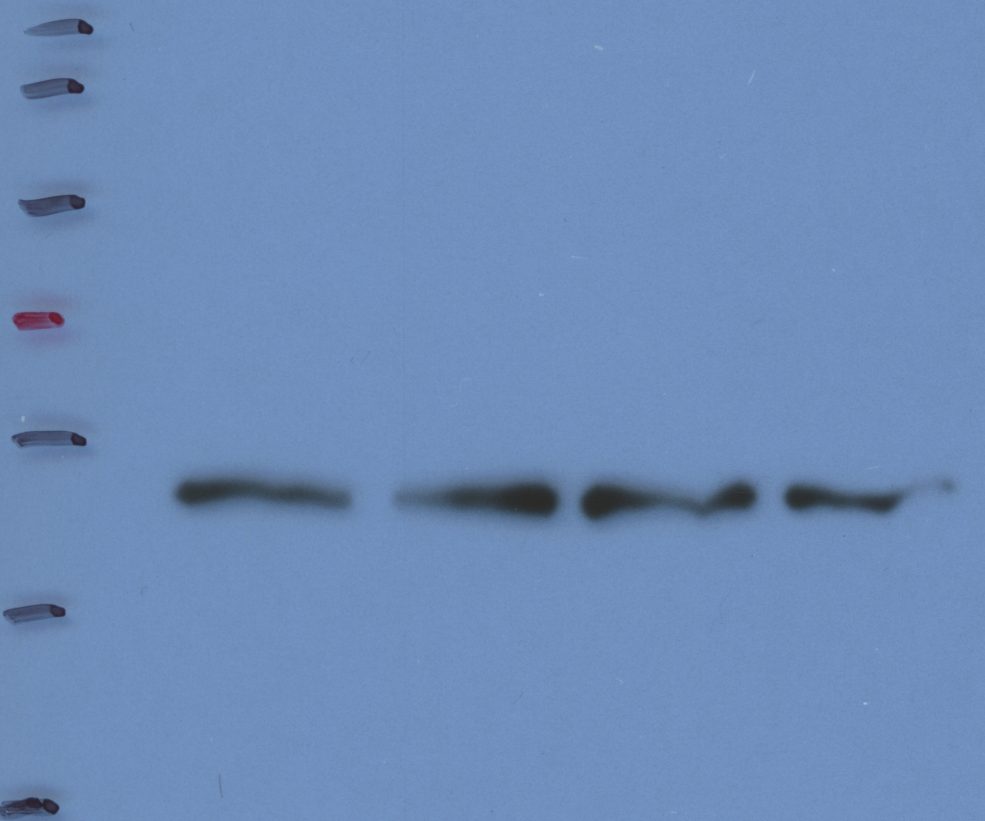

Supplement: Supplementary file 6 — Source Data for Figure 2 [file EMBR-24-e55895-s003.zip › Figure 2/2b/2b_tubulin_n2.tif]

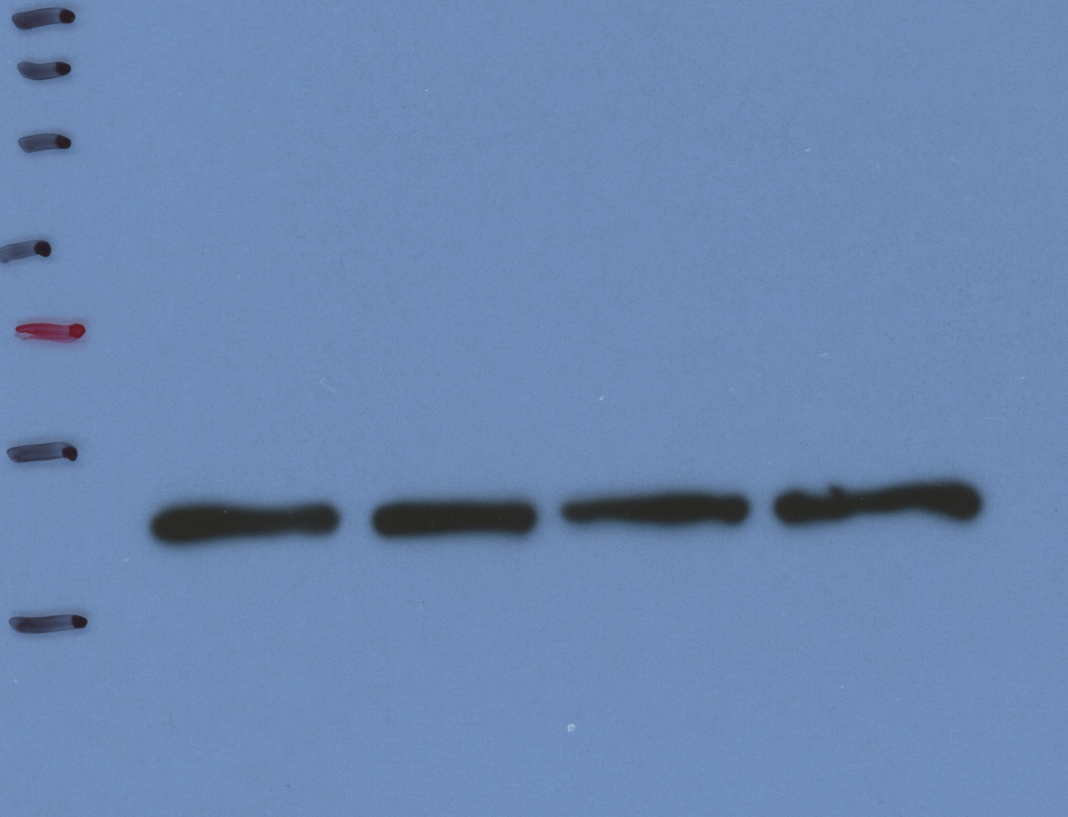

Supplement: Supplementary file 6 — Source Data for Figure 2 [file EMBR-24-e55895-s003.zip › Figure 2/2b/2b_tubulin_n3.tif]

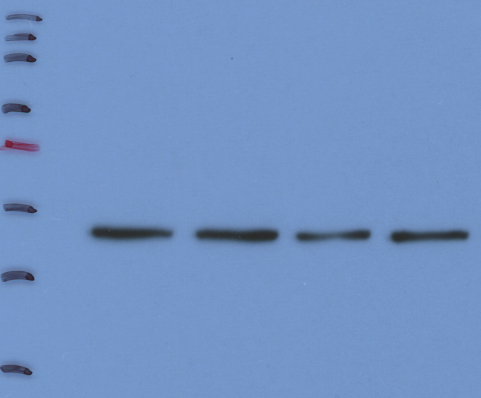

Supplement: Supplementary file 6 — Source Data for Figure 2 [file EMBR-24-e55895-s003.zip › Figure 2/2b/2b_tubulin_n1.tif]

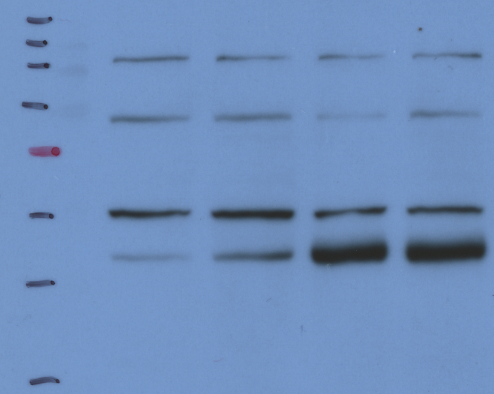

Supplement: Supplementary file 6 — Source Data for Figure 2 [file EMBR-24-e55895-s003.zip › Figure 2/2b/2b_C9orf72_n1.tif]

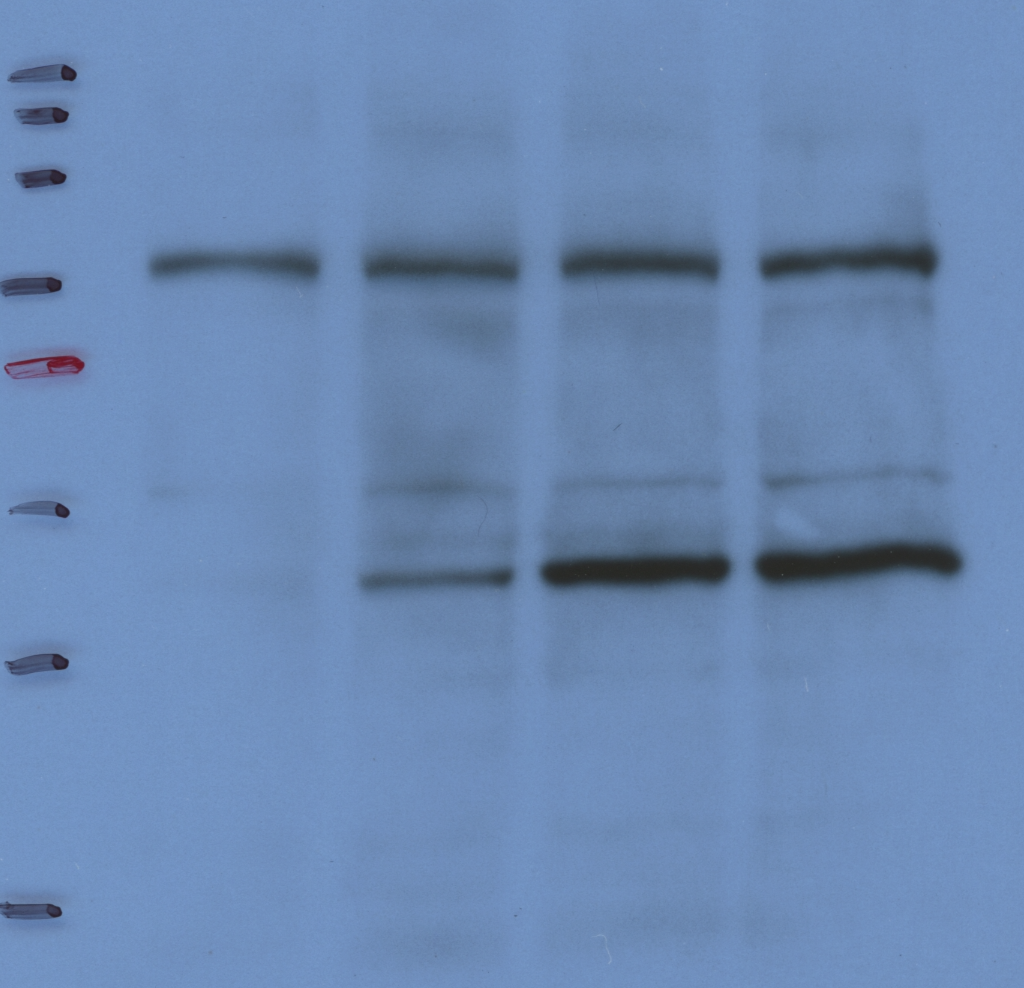

Supplement: Supplementary file 6 — Source Data for Figure 2 [file EMBR-24-e55895-s003.zip › Figure 2/2b/2b_C9orf72_n3.tif]

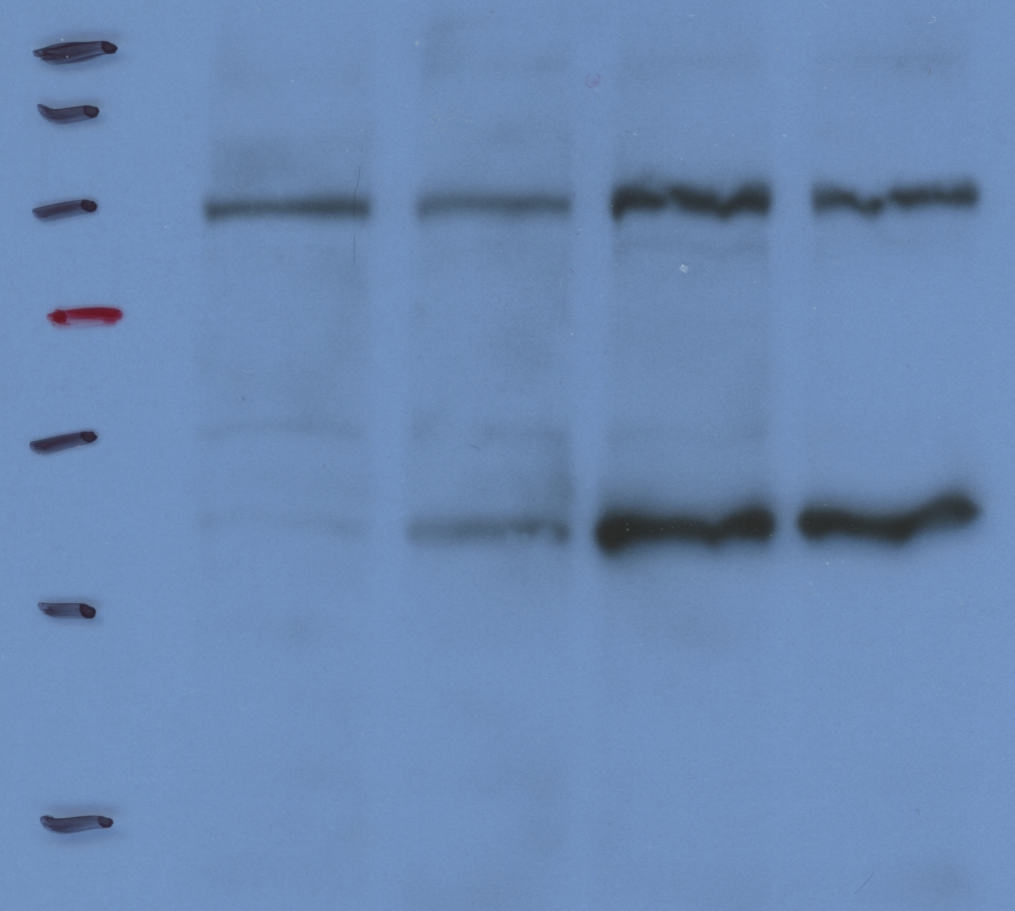

Supplement: Supplementary file 6 — Source Data for Figure 2 [file EMBR-24-e55895-s003.zip › Figure 2/2b/2b_C9orf72_n2.tif]

Figure 3C

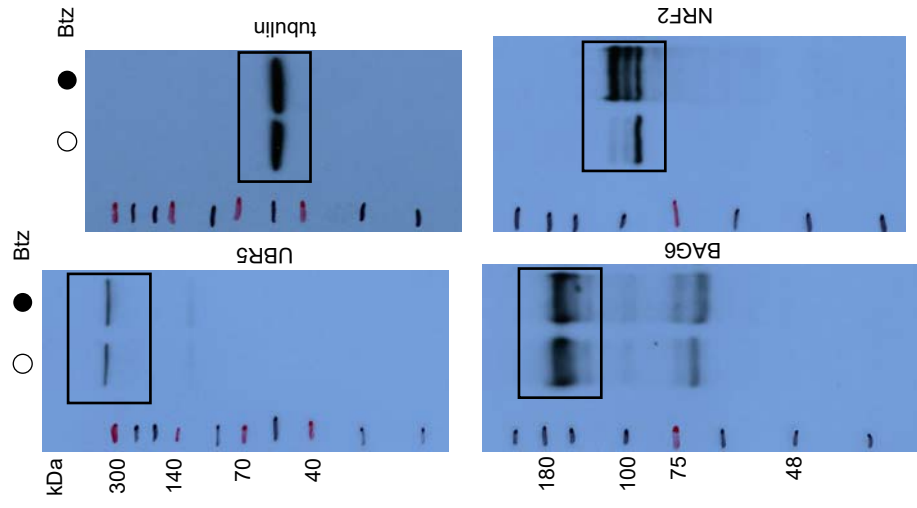

Figure 3E

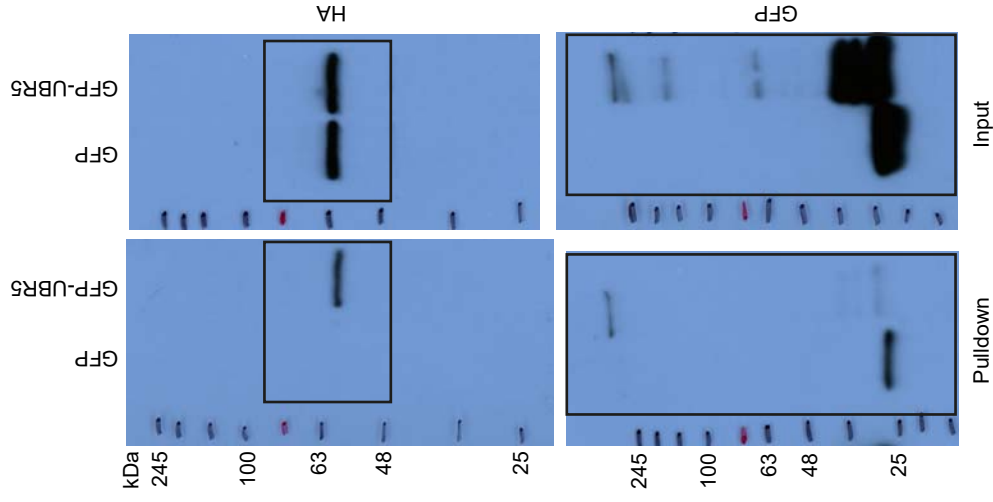

Figure 3F

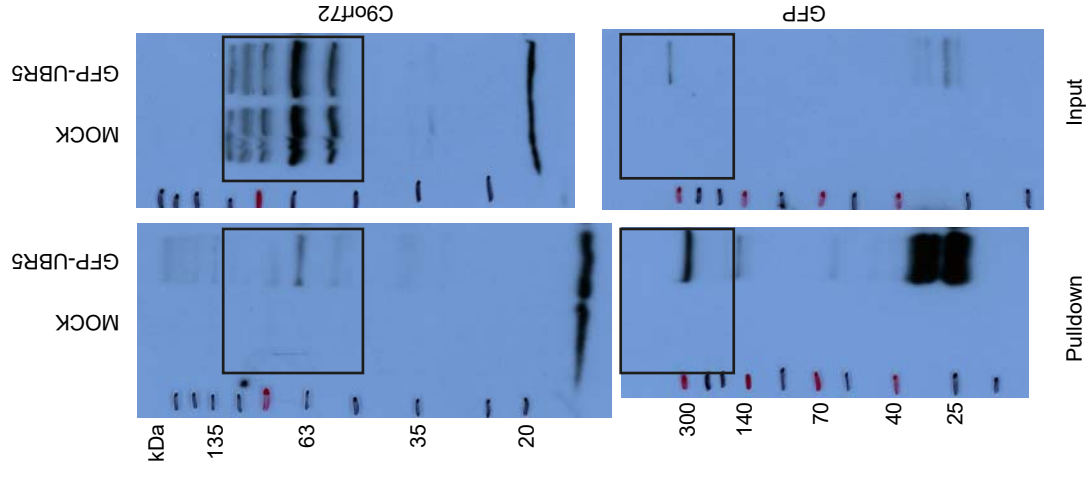

Figure 3D

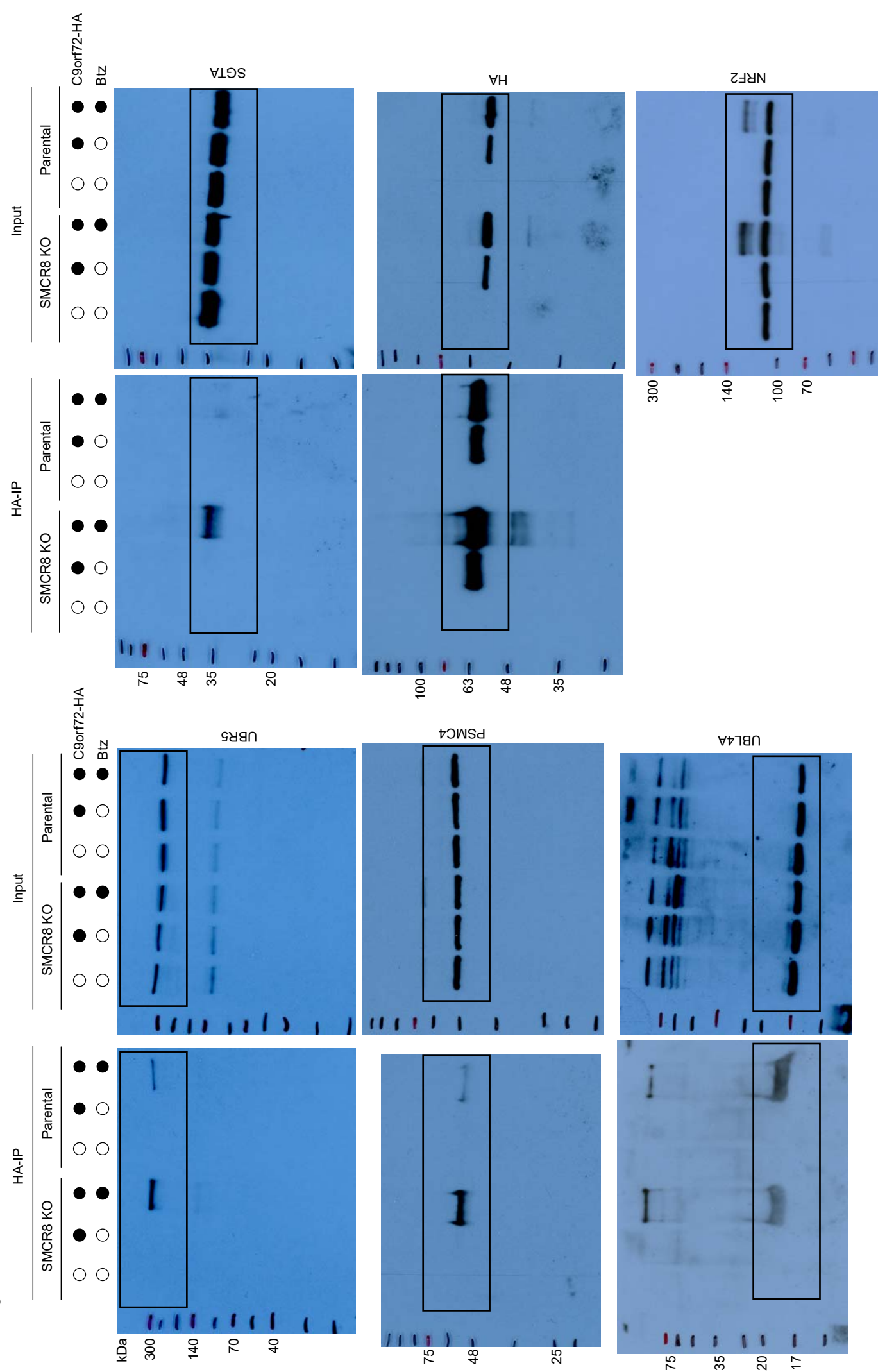

Figure 3D

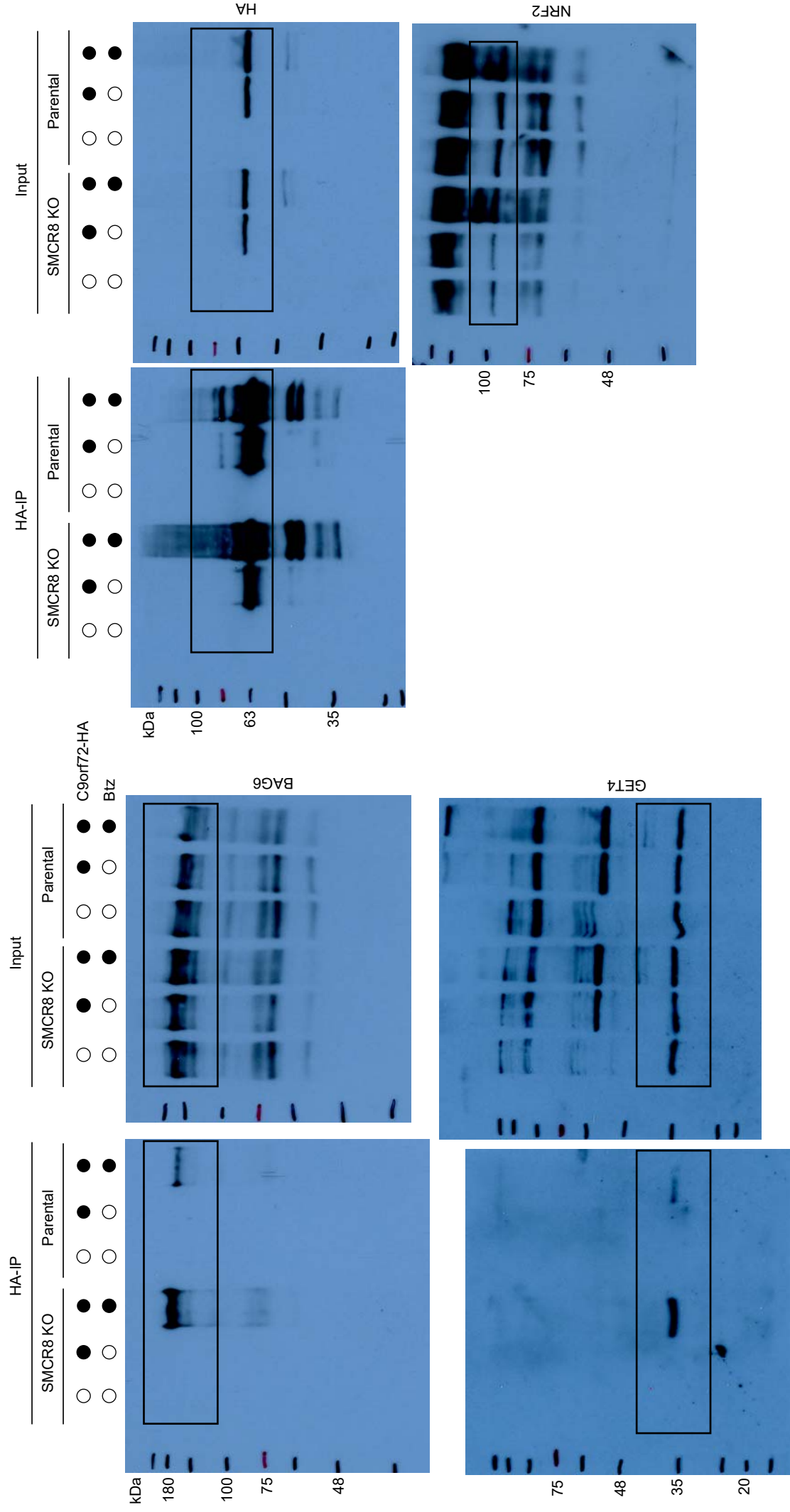

Supplement: Supplementary file 7 — Source Data for Figure 3 [file EMBR-24-e55895-s001.zip › Figure 3/Figure 3.pdf]

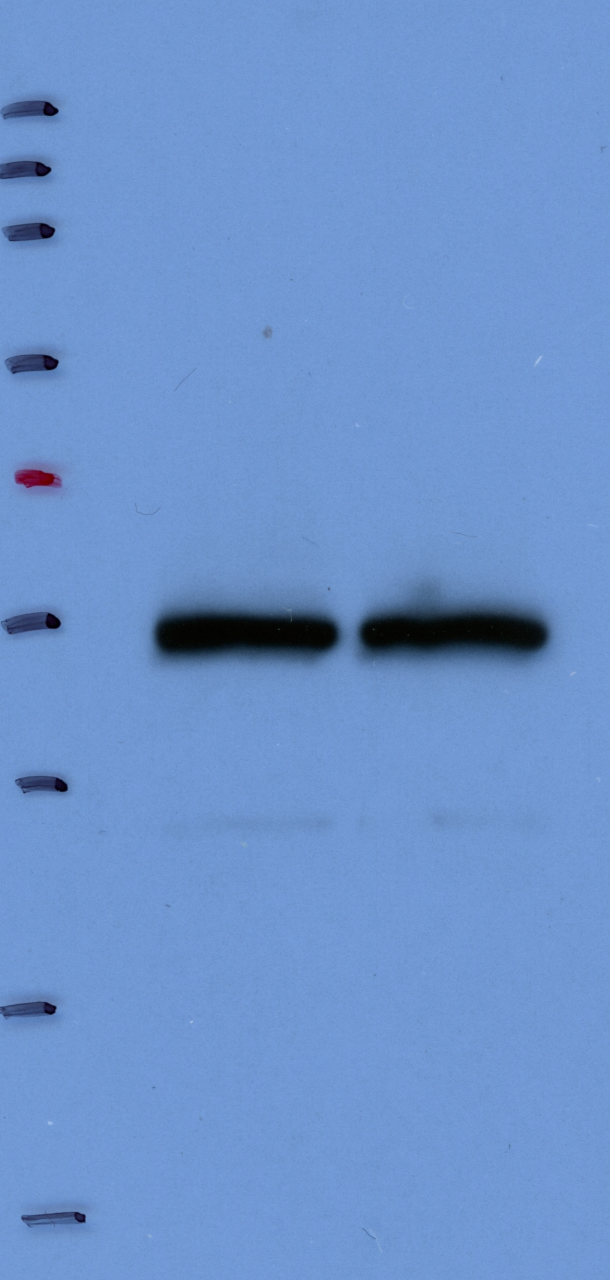

Supplement: Supplementary file 7 — Source Data for Figure 3 [file EMBR-24-e55895-s001.zip › Figure 3/3e/3e_HA_input.tif]

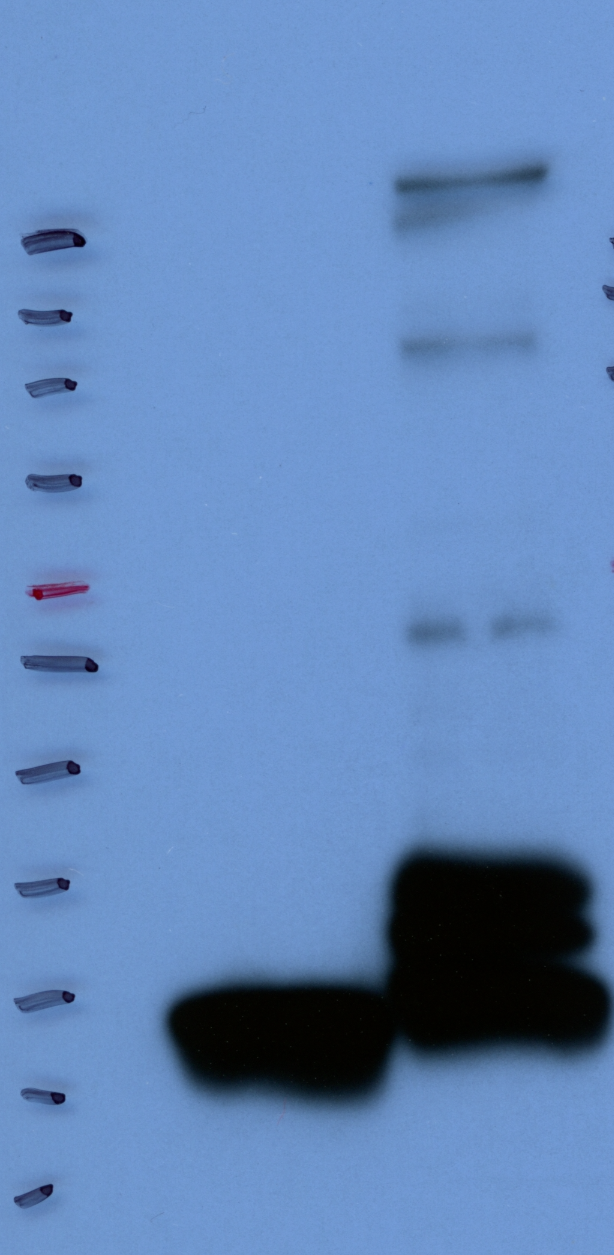

Supplement: Supplementary file 7 — Source Data for Figure 3 [file EMBR-24-e55895-s001.zip › Figure 3/3e/3e_GFP_IP.tif]

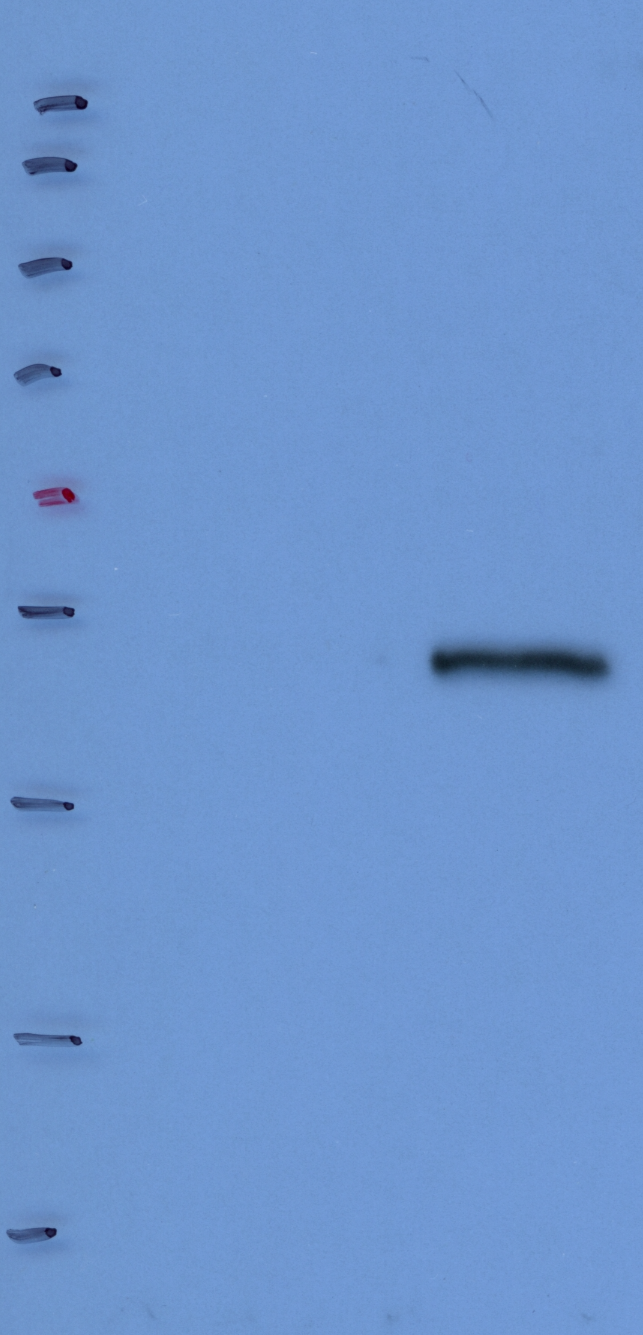

Supplement: Supplementary file 7 — Source Data for Figure 3 [file EMBR-24-e55895-s001.zip › Figure 3/3e/3e_HA_IP.tif]

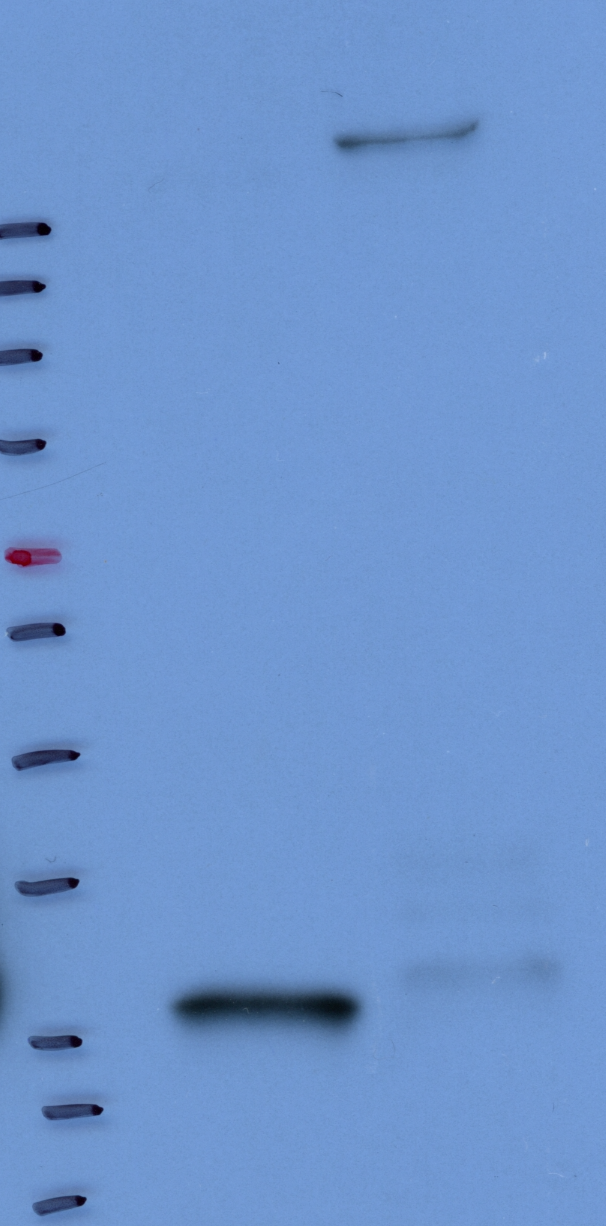

Supplement: Supplementary file 7 — Source Data for Figure 3 [file EMBR-24-e55895-s001.zip › Figure 3/3e/3e_GFP_input.tif]

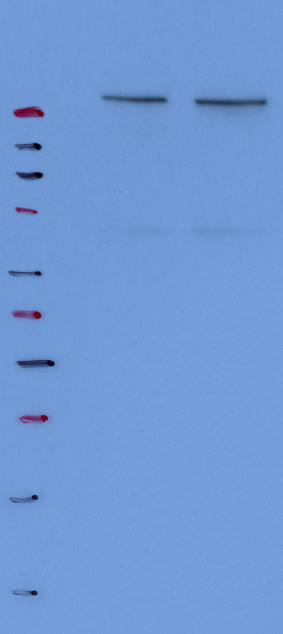

Supplement: Supplementary file 7 — Source Data for Figure 3 [file EMBR-24-e55895-s001.zip › Figure 3/3c/3c_UBR5.tif]

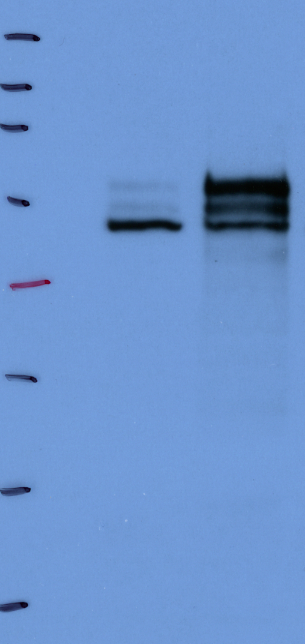

Supplement: Supplementary file 7 — Source Data for Figure 3 [file EMBR-24-e55895-s001.zip › Figure 3/3c/3c_NRF2.tif]

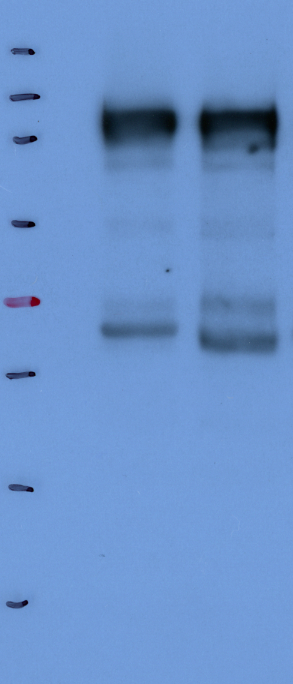

Supplement: Supplementary file 7 — Source Data for Figure 3 [file EMBR-24-e55895-s001.zip › Figure 3/3c/3c_BAG6.tif]

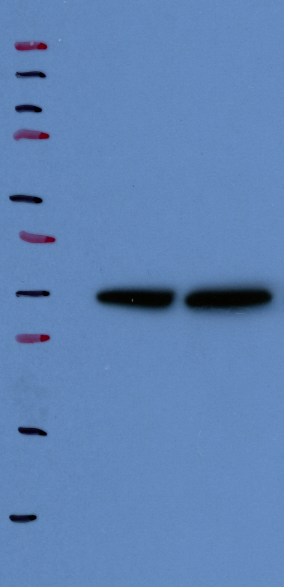

Supplement: Supplementary file 7 — Source Data for Figure 3 [file EMBR-24-e55895-s001.zip › Figure 3/3c/3c_tubulin.tif]

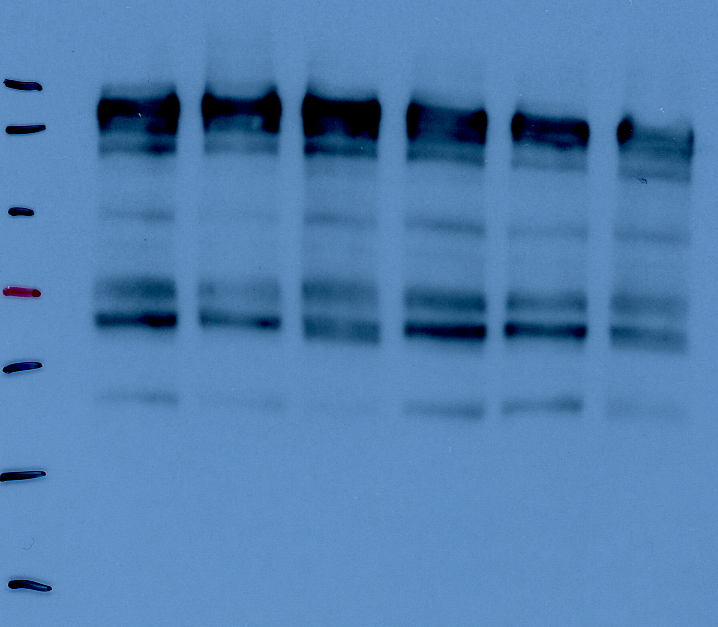

Supplement: Supplementary file 7 — Source Data for Figure 3 [file EMBR-24-e55895-s001.zip › Figure 3/3d/3d_BAG6 input.tif]

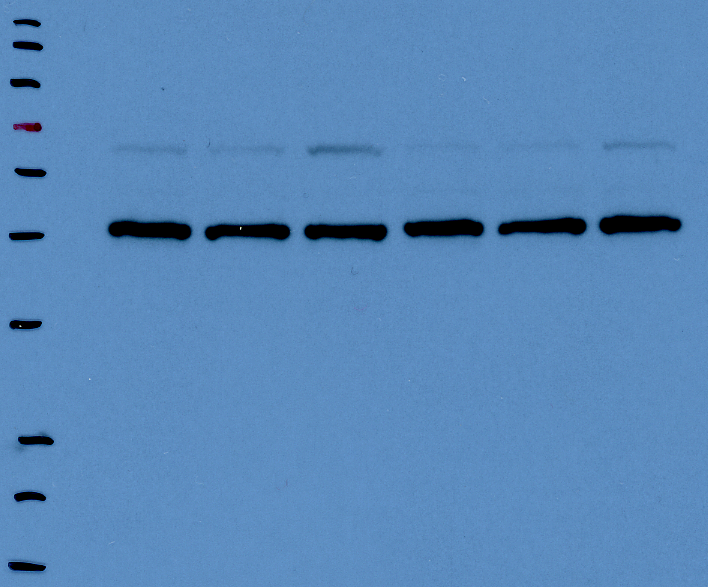

Supplement: Supplementary file 7 — Source Data for Figure 3 [file EMBR-24-e55895-s001.zip › Figure 3/3d/3d_PSMC4 input.tif]

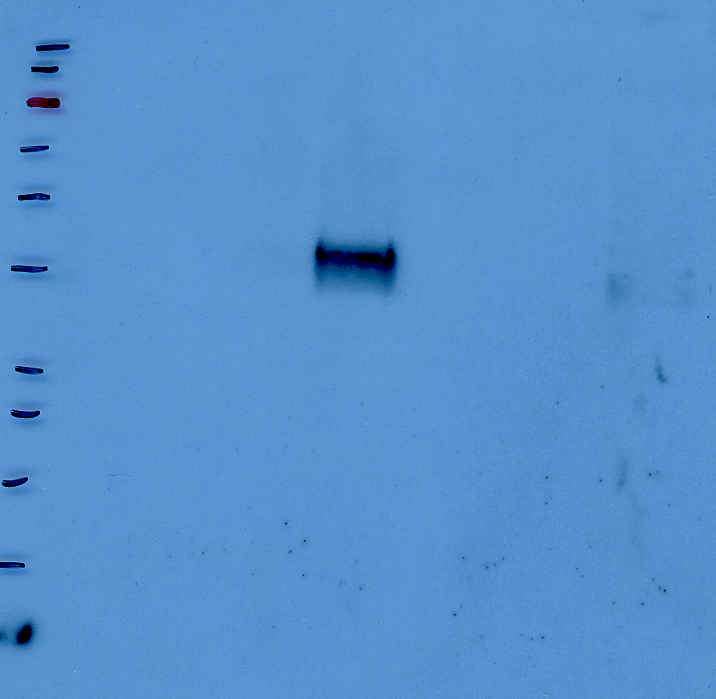

Supplement: Supplementary file 7 — Source Data for Figure 3 [file EMBR-24-e55895-s001.zip › Figure 3/3d/3d_SGTA IP.tif]

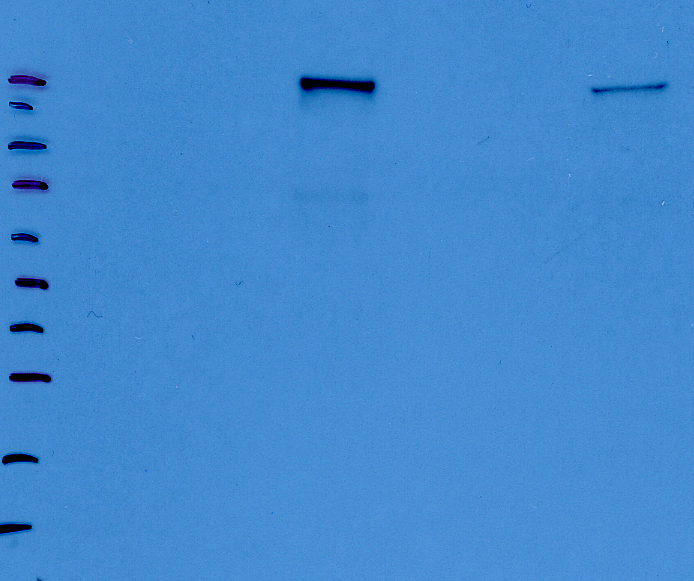

Supplement: Supplementary file 7 — Source Data for Figure 3 [file EMBR-24-e55895-s001.zip › Figure 3/3d/3d_UBR5 IP.tif]

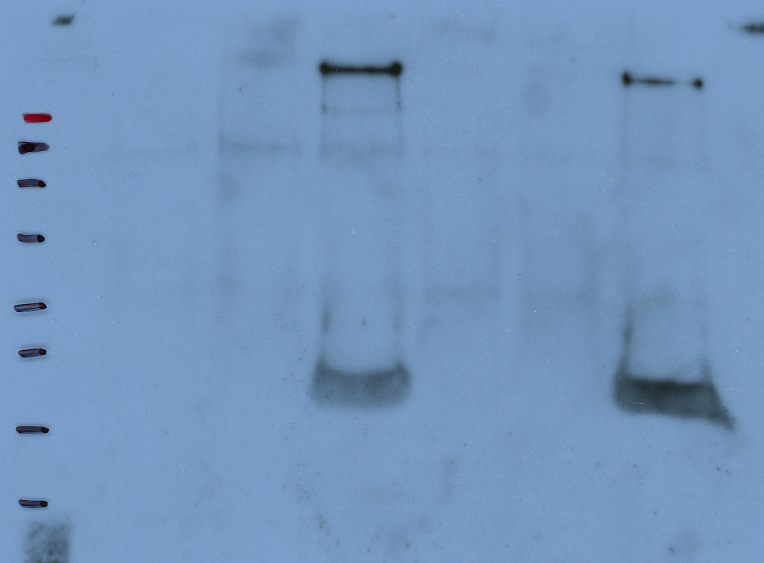

Supplement: Supplementary file 7 — Source Data for Figure 3 [file EMBR-24-e55895-s001.zip › Figure 3/3d/3d_UBL4A IP.tif]

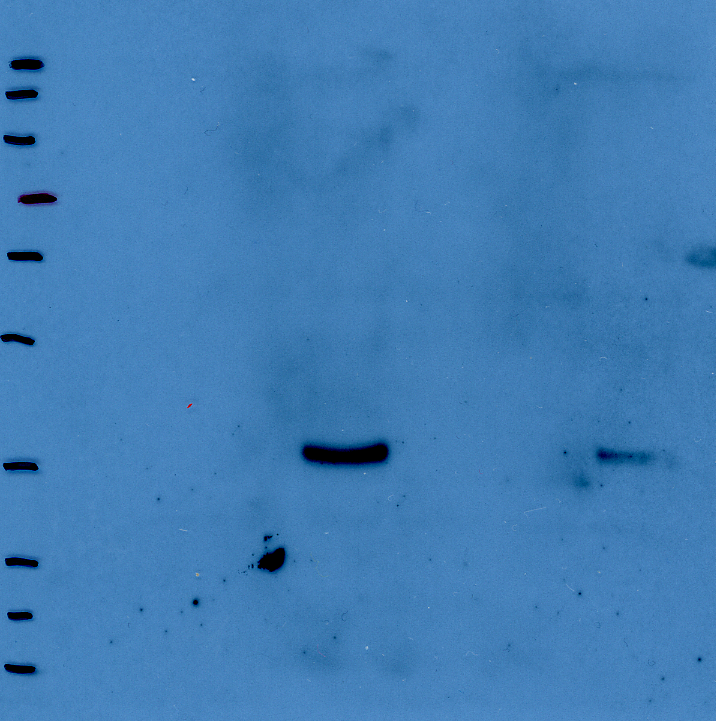

Supplement: Supplementary file 7 — Source Data for Figure 3 [file EMBR-24-e55895-s001.zip › Figure 3/3d/3d_GET4 IP.tif]

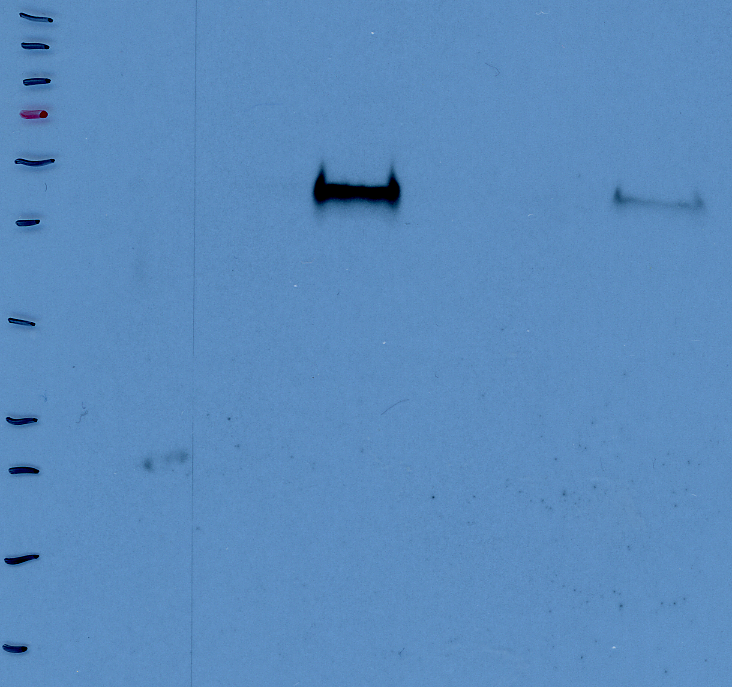

Supplement: Supplementary file 7 — Source Data for Figure 3 [file EMBR-24-e55895-s001.zip › Figure 3/3d/3d_PSMC4 IP.tif]

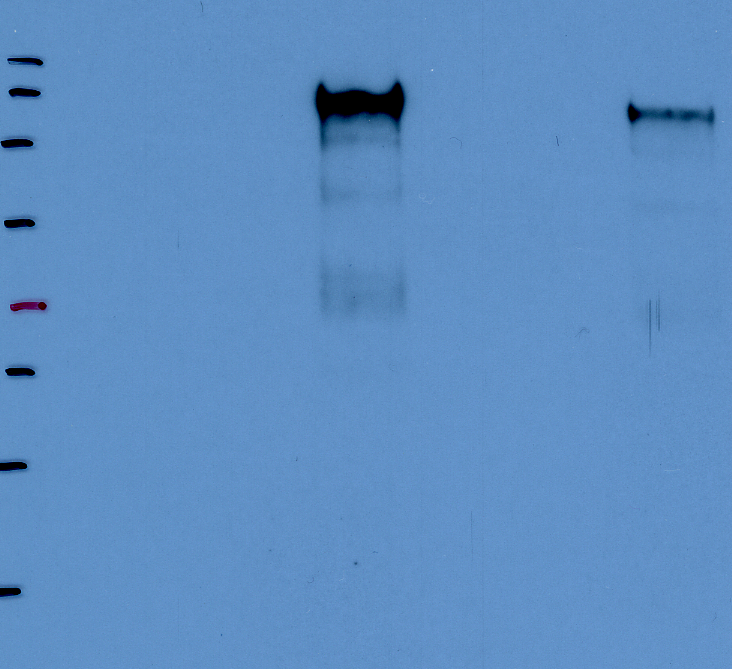

Supplement: Supplementary file 7 — Source Data for Figure 3 [file EMBR-24-e55895-s001.zip › Figure 3/3d/3d_BAG6 IP.tif]

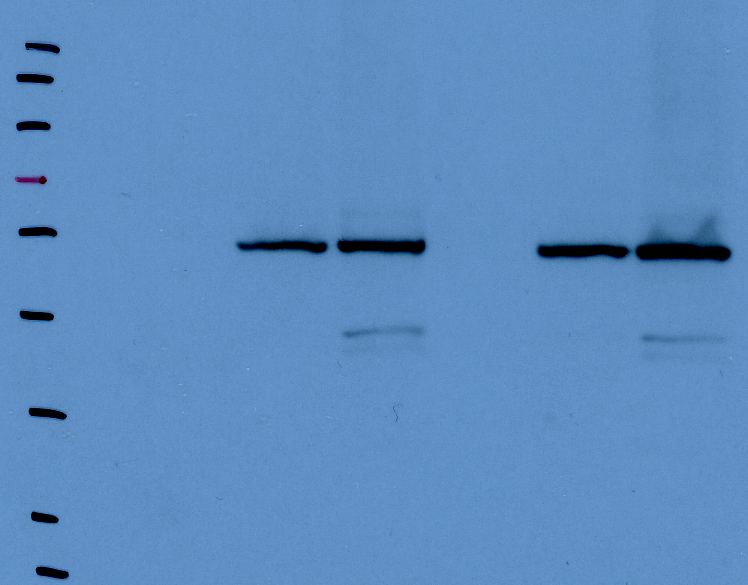

Supplement: Supplementary file 7 — Source Data for Figure 3 [file EMBR-24-e55895-s001.zip › Figure 3/3d/3d_HA input_2.tif]

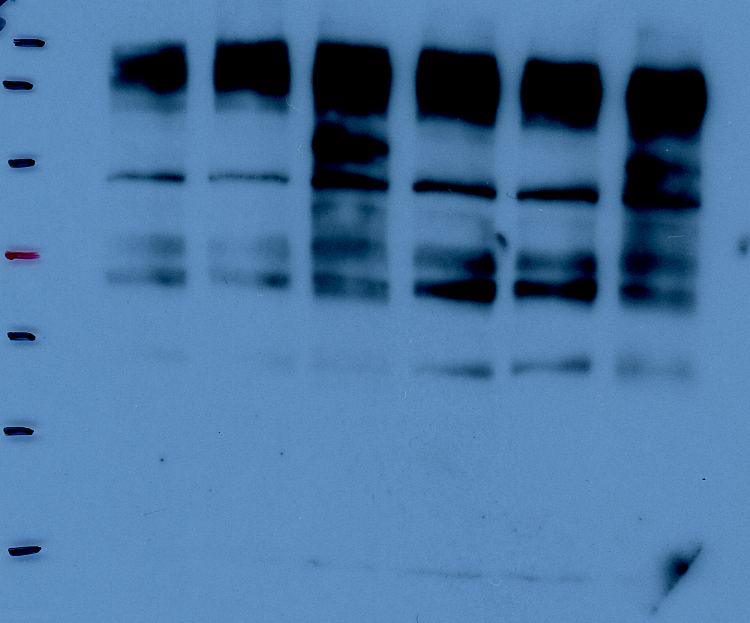

Supplement: Supplementary file 7 — Source Data for Figure 3 [file EMBR-24-e55895-s001.zip › Figure 3/3d/3d_NRF2_2.tif]

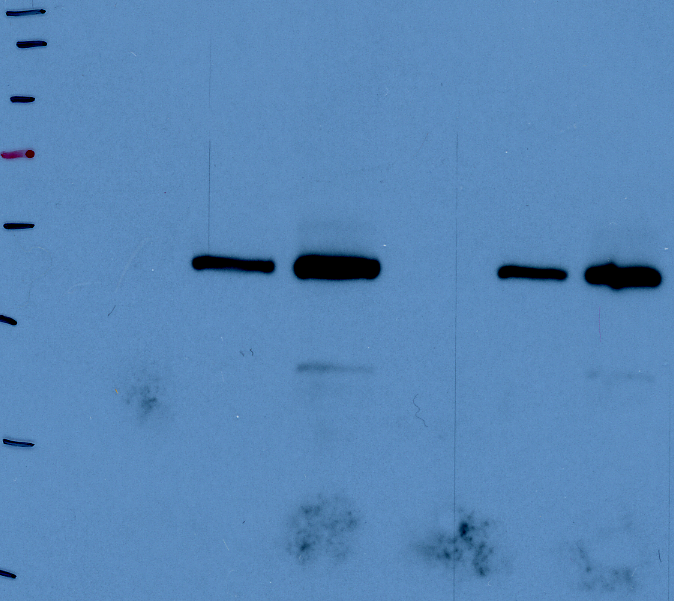

Supplement: Supplementary file 7 — Source Data for Figure 3 [file EMBR-24-e55895-s001.zip › Figure 3/3d/3d_HA input_1.tif]

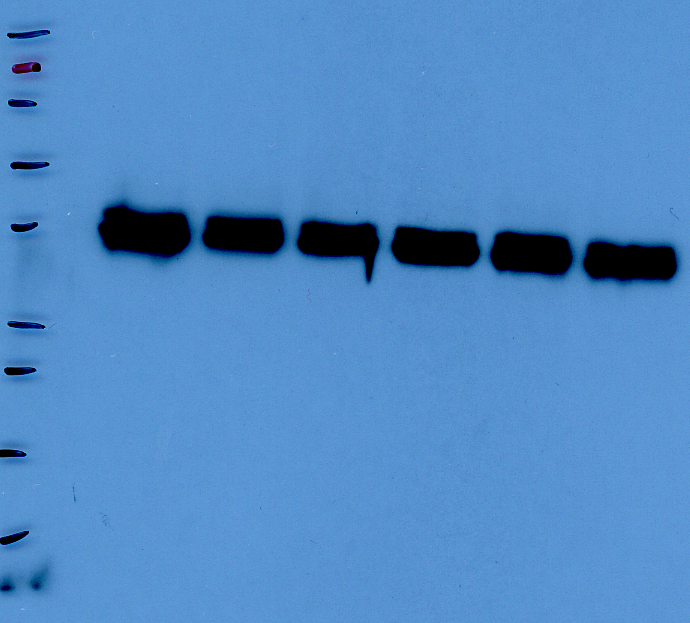

Supplement: Supplementary file 7 — Source Data for Figure 3 [file EMBR-24-e55895-s001.zip › Figure 3/3d/3d_SGTA input.tif]

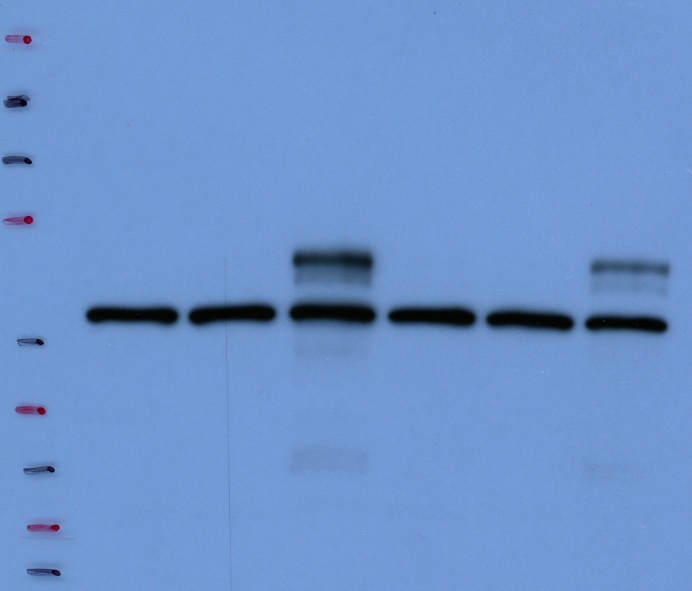

Supplement: Supplementary file 7 — Source Data for Figure 3 [file EMBR-24-e55895-s001.zip › Figure 3/3d/3d_NRF2_1.tif]

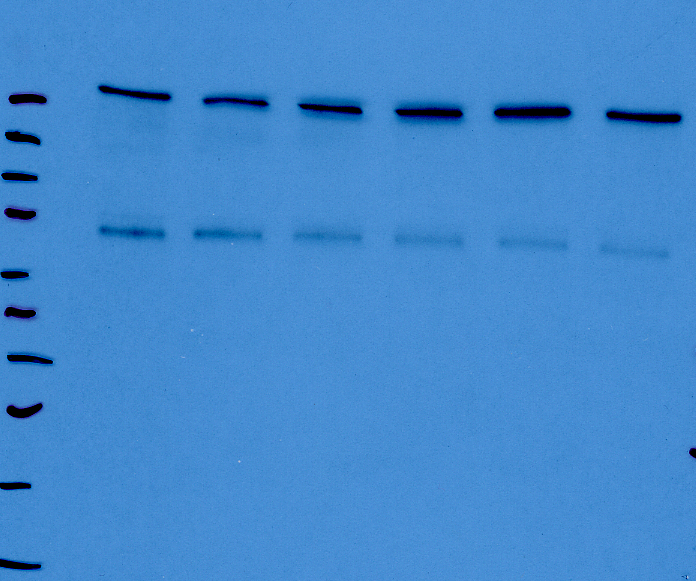

Supplement: Supplementary file 7 — Source Data for Figure 3 [file EMBR-24-e55895-s001.zip › Figure 3/3d/3d_UBR5 input.tif]

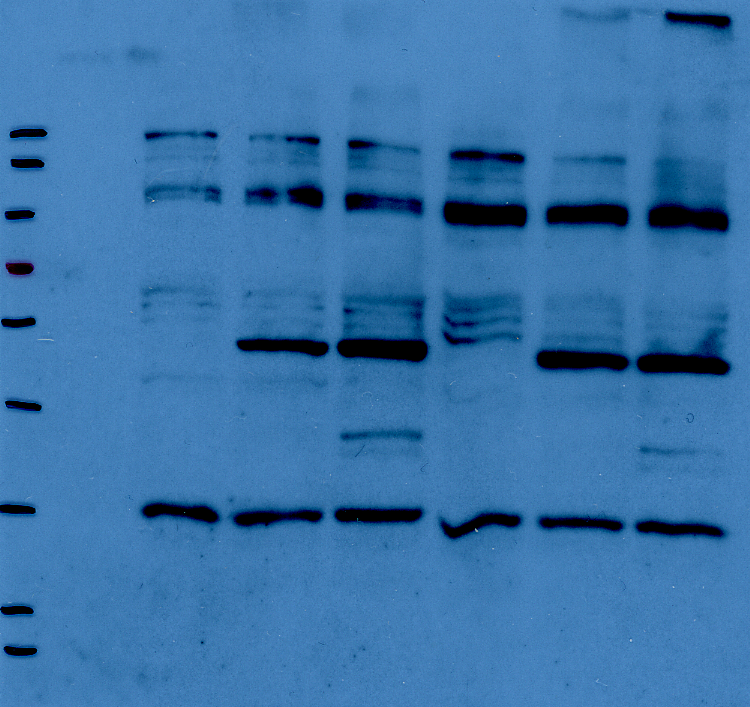

Supplement: Supplementary file 7 — Source Data for Figure 3 [file EMBR-24-e55895-s001.zip › Figure 3/3d/3d_GET4 input.tif]

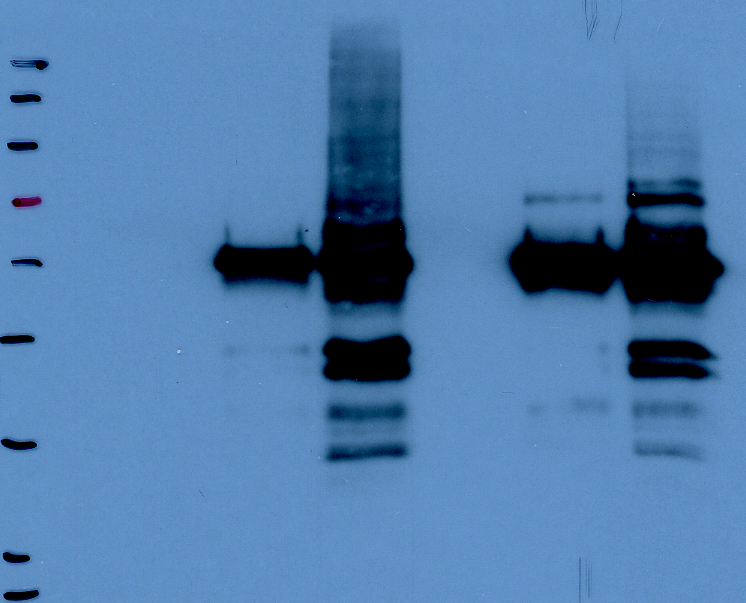

Supplement: Supplementary file 7 — Source Data for Figure 3 [file EMBR-24-e55895-s001.zip › Figure 3/3d/3d_HA IP_2.tif]

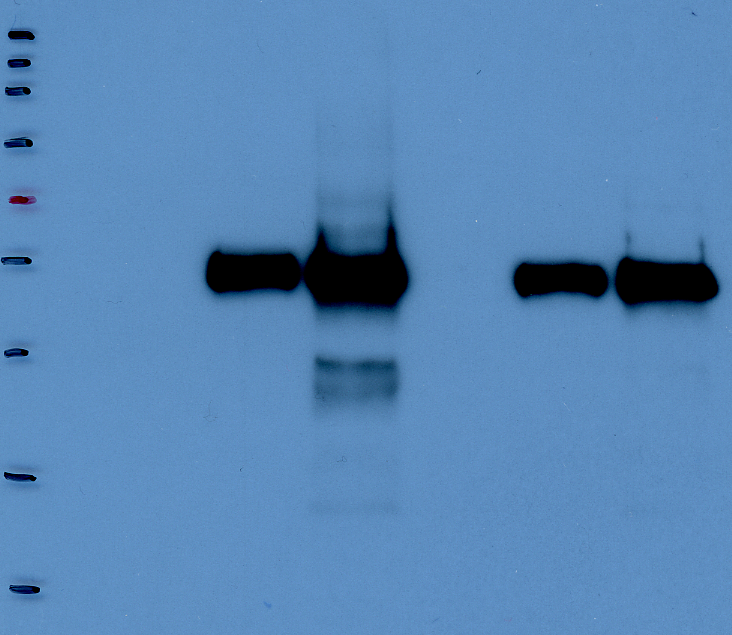

Supplement: Supplementary file 7 — Source Data for Figure 3 [file EMBR-24-e55895-s001.zip › Figure 3/3d/3d_HA IP_1.tif]

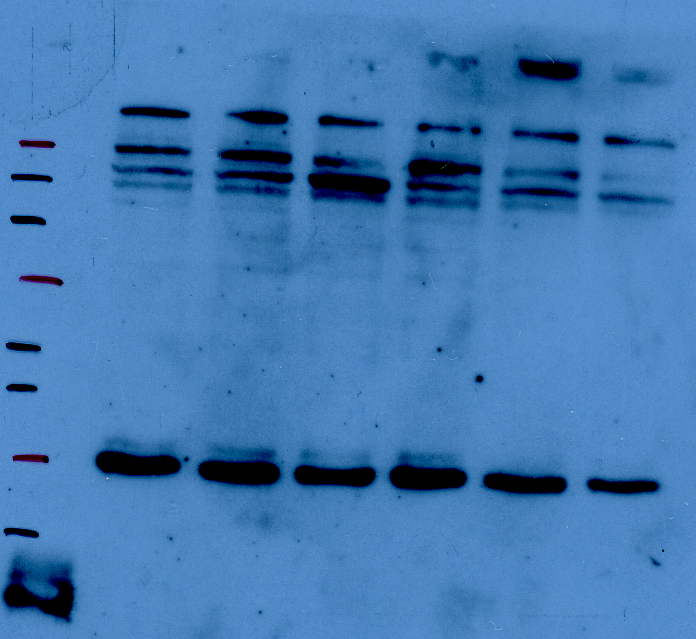

Supplement: Supplementary file 7 — Source Data for Figure 3 [file EMBR-24-e55895-s001.zip › Figure 3/3d/3d_UBL4A input.tif]

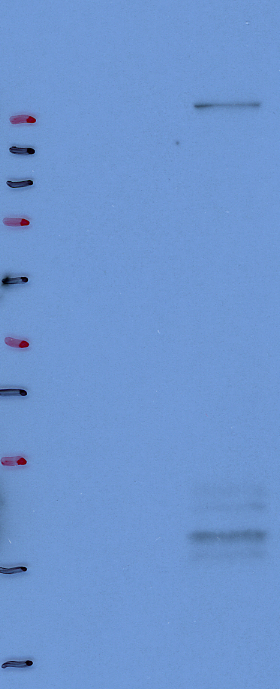

Supplement: Supplementary file 7 — Source Data for Figure 3 [file EMBR-24-e55895-s001.zip › Figure 3/3f/3f_GFP_input.tif]

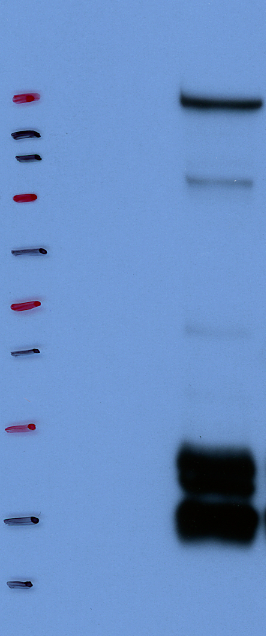

Supplement: Supplementary file 7 — Source Data for Figure 3 [file EMBR-24-e55895-s001.zip › Figure 3/3f/3f_GFP_IP.tif]

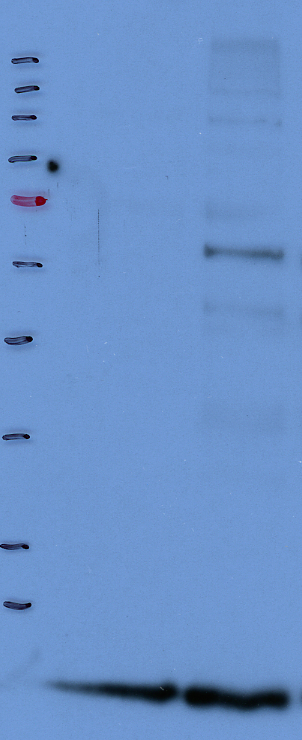

Supplement: Supplementary file 7 — Source Data for Figure 3 [file EMBR-24-e55895-s001.zip › Figure 3/3f/3f_C9orf72_IP.tif]

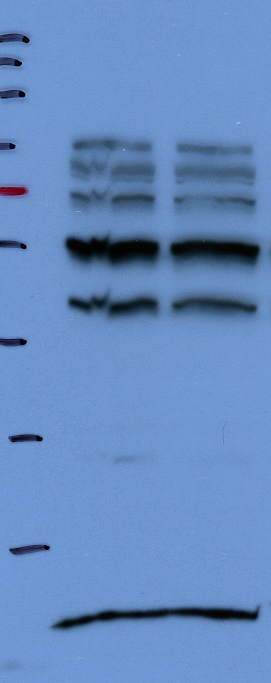

Supplement: Supplementary file 7 — Source Data for Figure 3 [file EMBR-24-e55895-s001.zip › Figure 3/3f/3f_C9orf72_input.tif]

Figure 4A

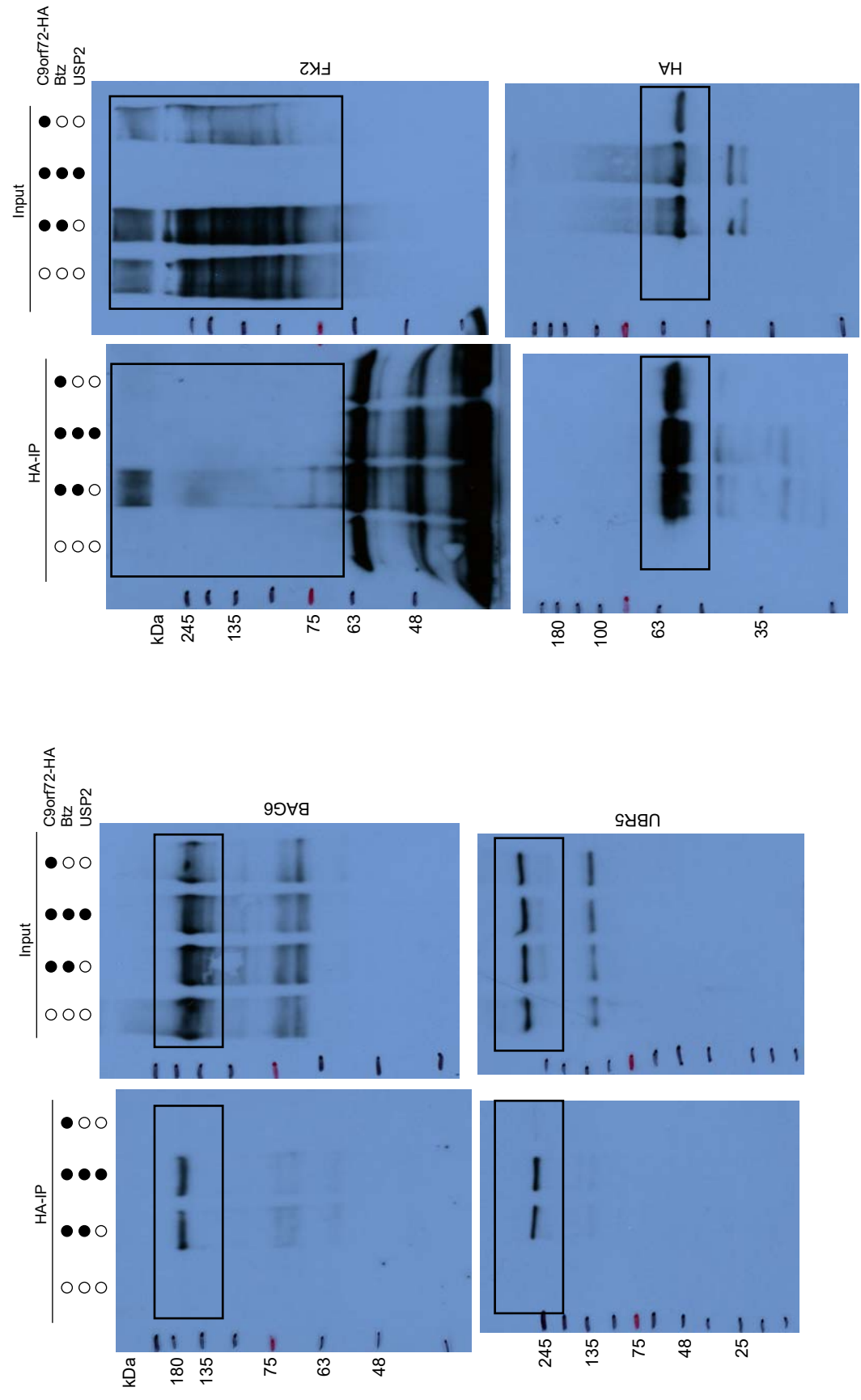

Figure 4B

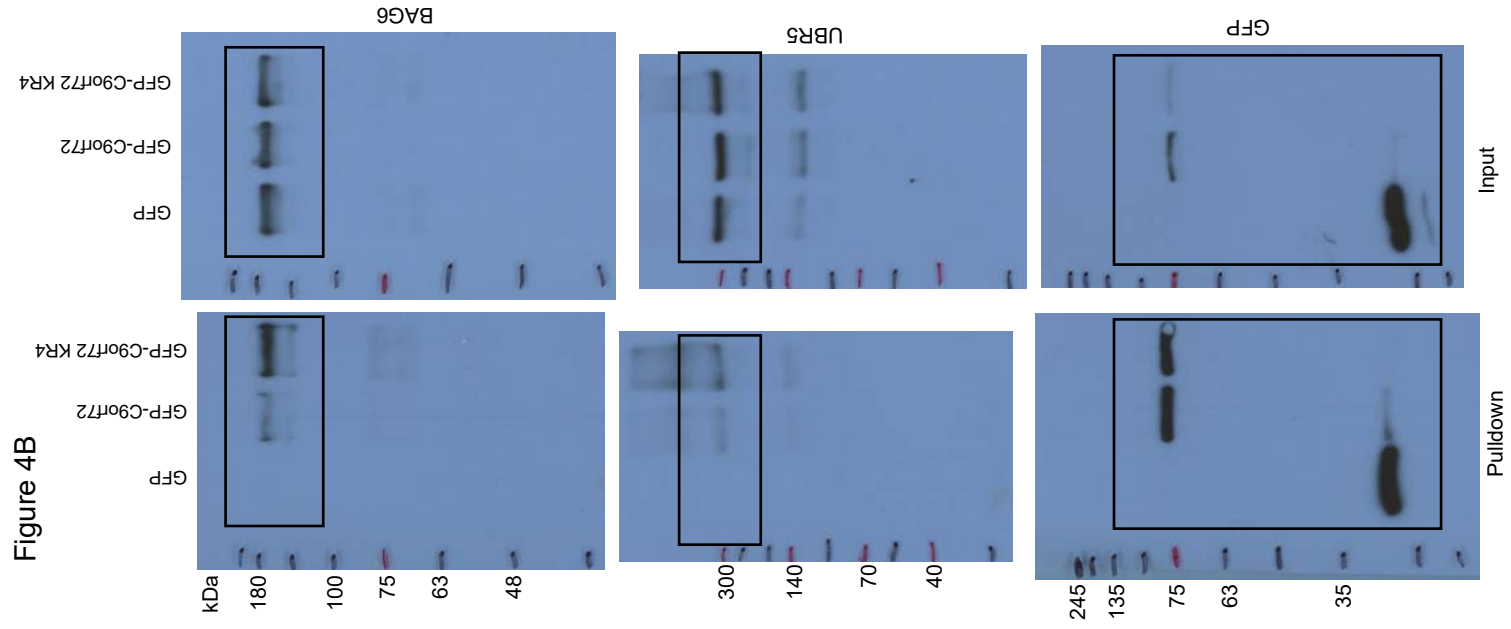

Figure 4C

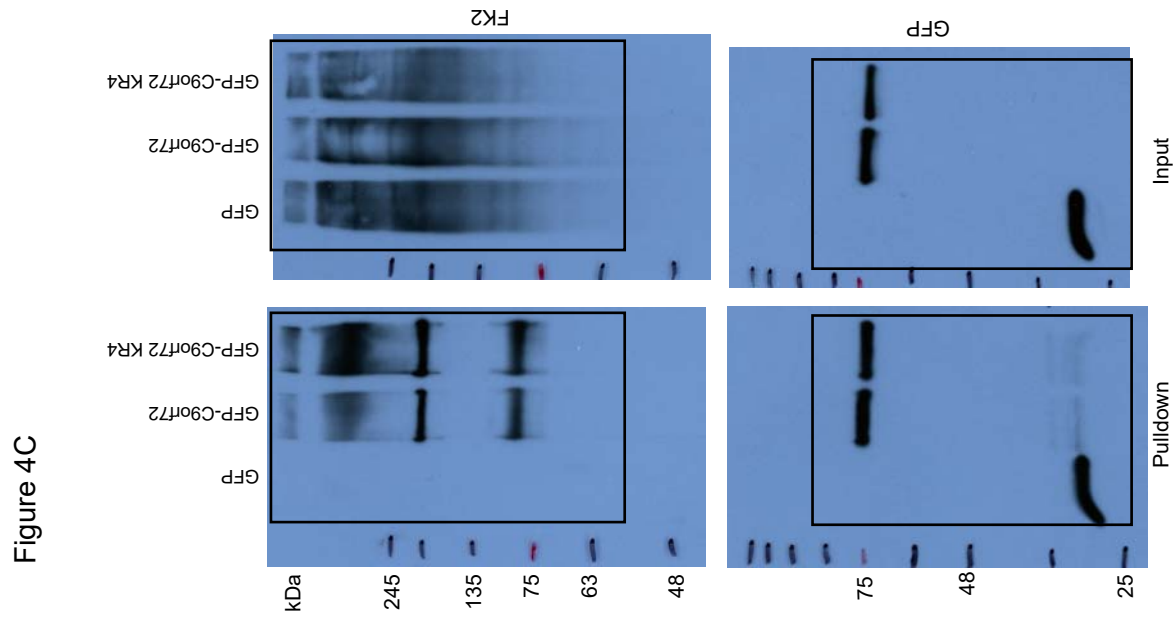

Figure 4D

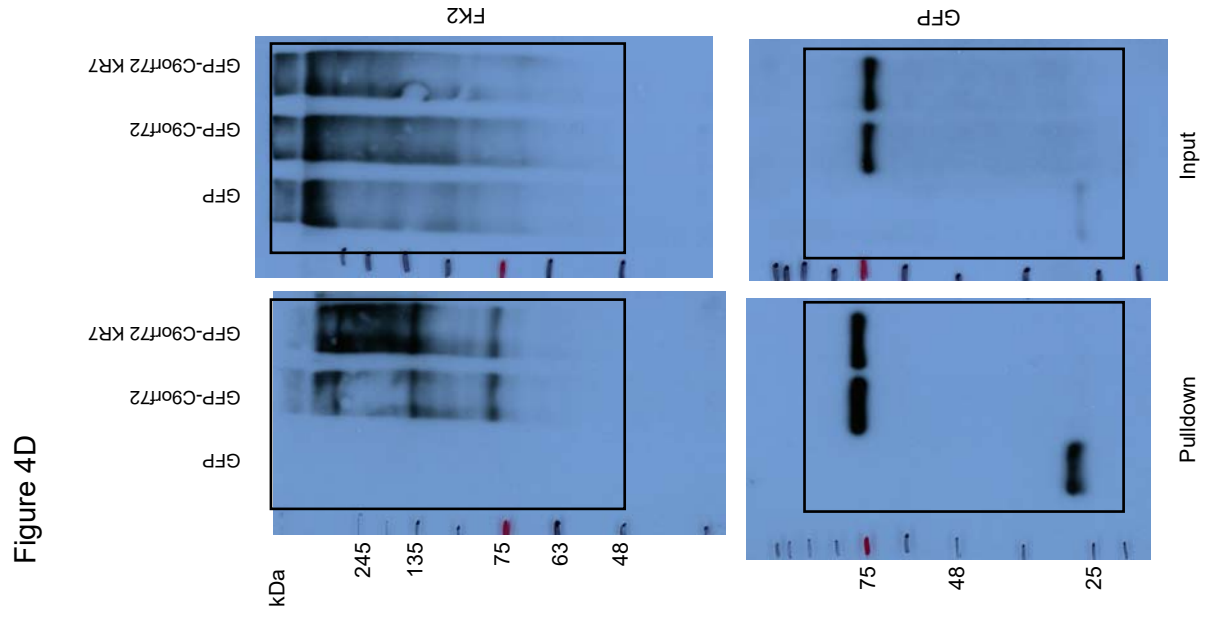

Supplement: Supplementary file 8 — Source Data for Figure 4 [file EMBR-24-e55895-s009.zip › Figure 4/Figure 4.pdf]

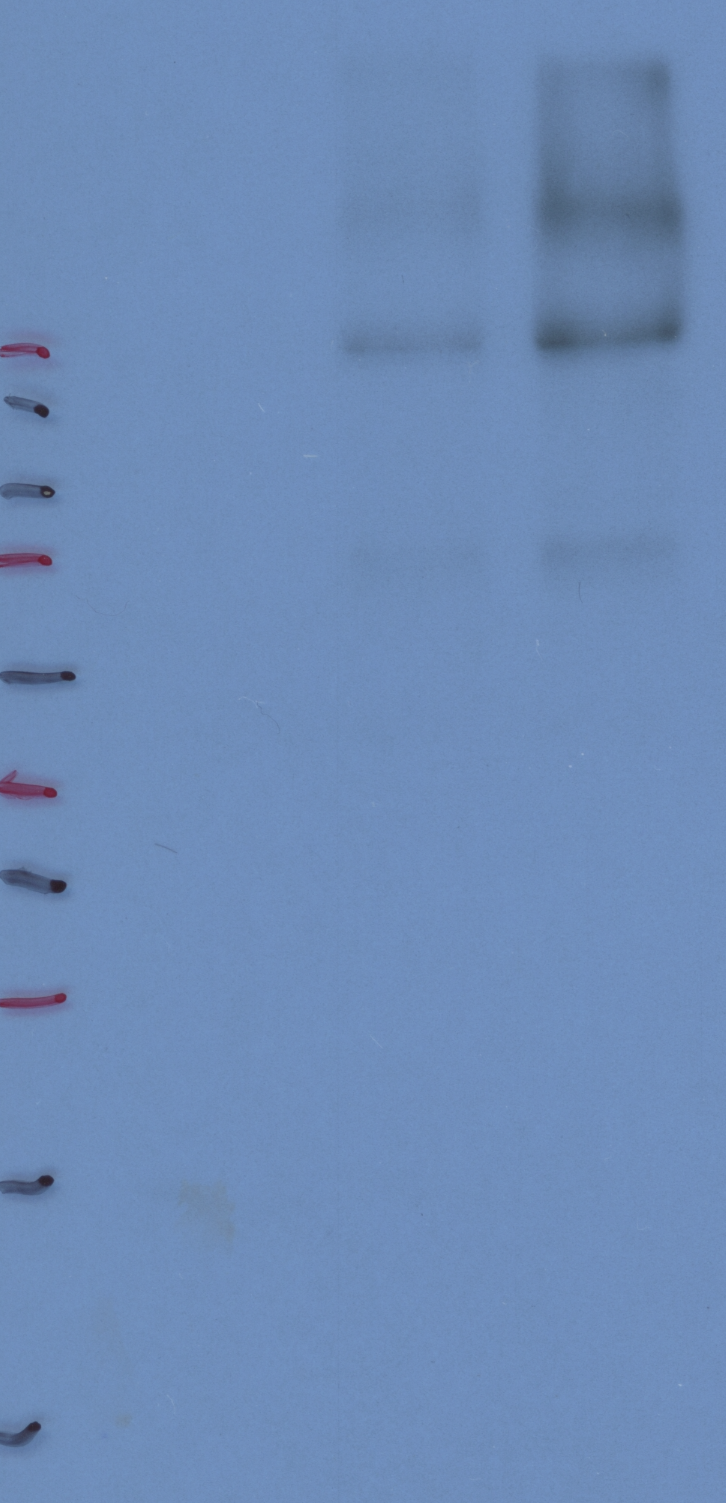

Supplement: Supplementary file 8 — Source Data for Figure 4 [file EMBR-24-e55895-s009.zip › Figure 4/4b/4b_UBR5_IP.tif]

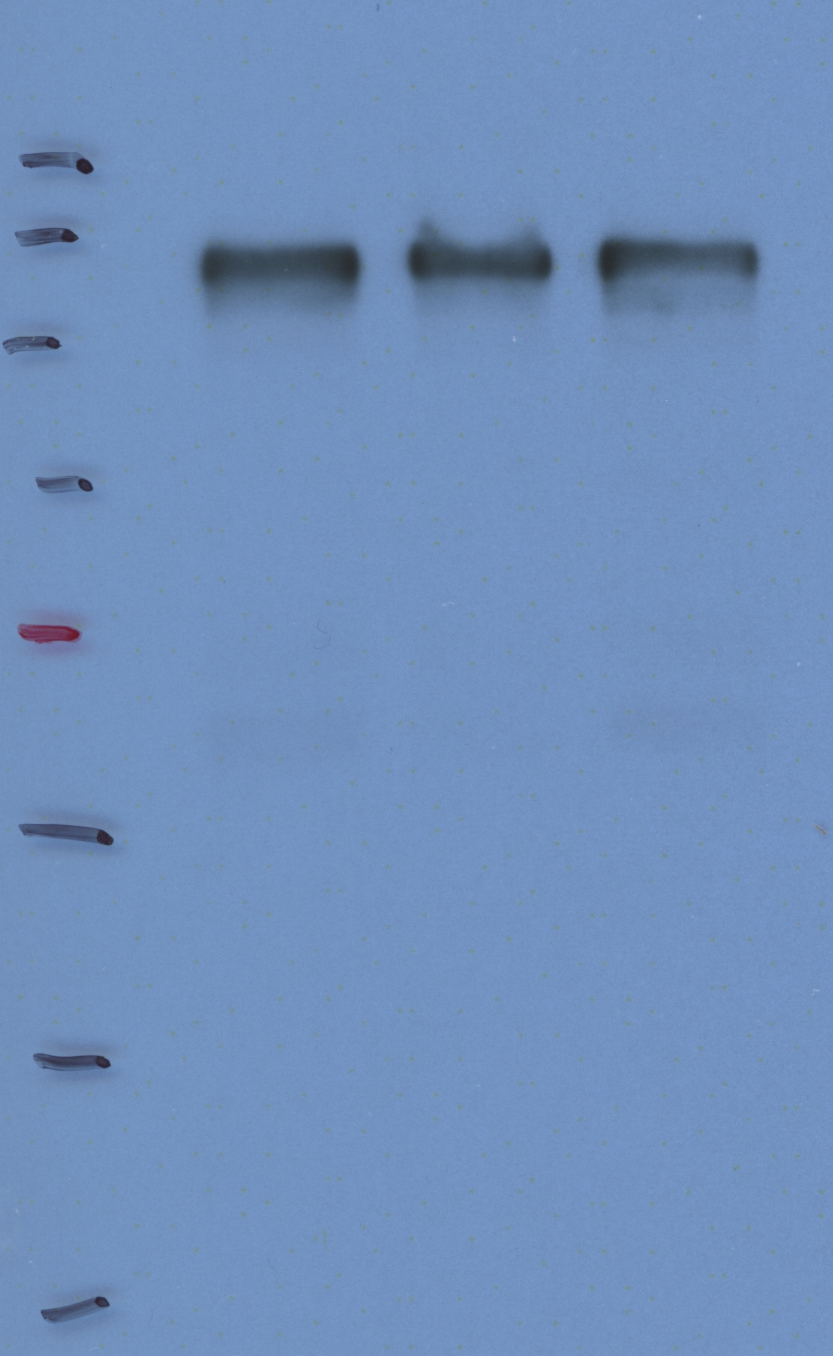

Supplement: Supplementary file 8 — Source Data for Figure 4 [file EMBR-24-e55895-s009.zip › Figure 4/4b/4b_Bag6_input.tif]

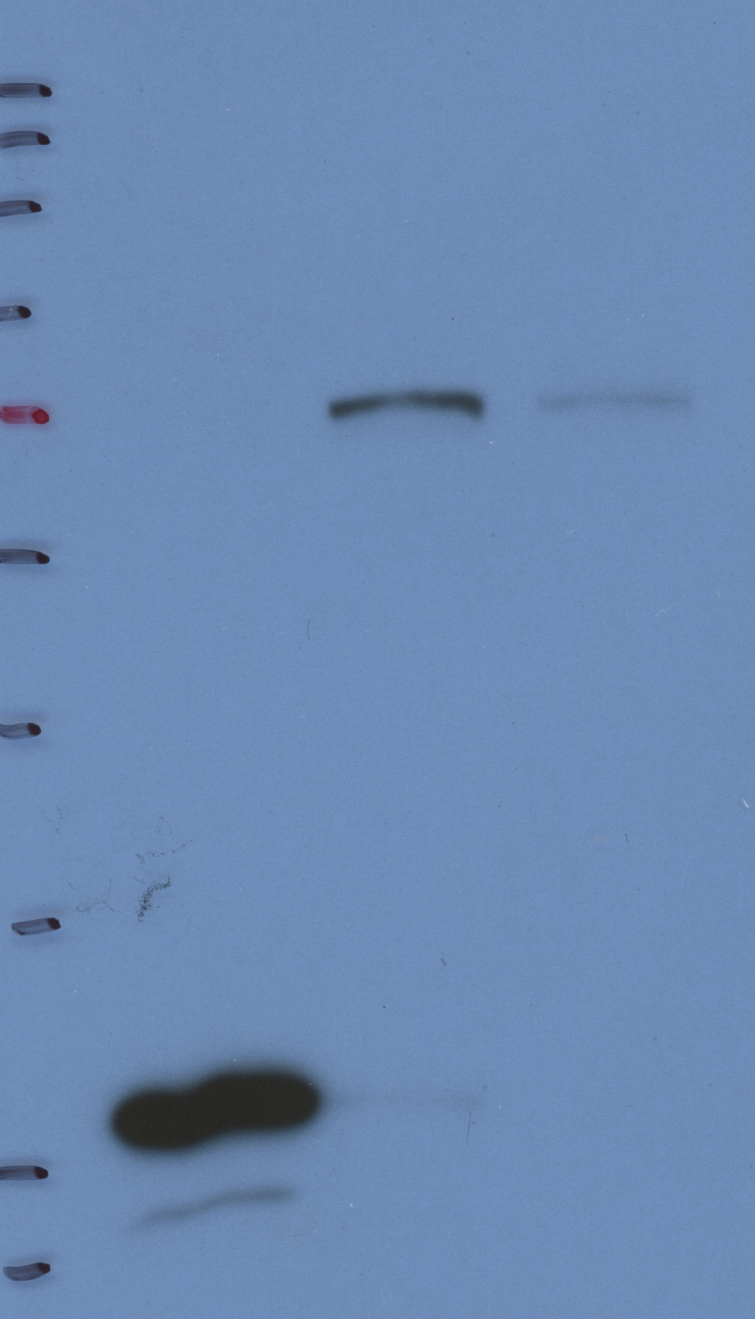

Supplement: Supplementary file 8 — Source Data for Figure 4 [file EMBR-24-e55895-s009.zip › Figure 4/4b/4b_GFP_input.tif]

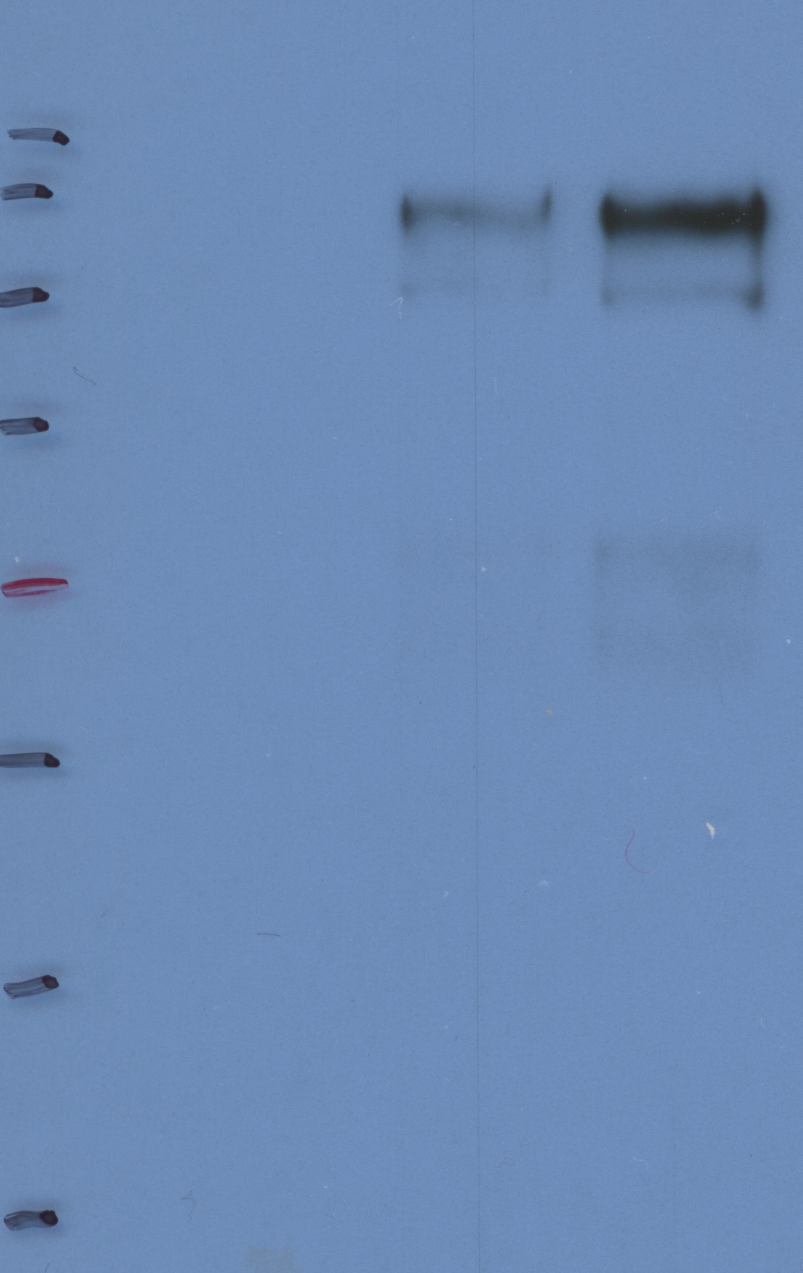

Supplement: Supplementary file 8 — Source Data for Figure 4 [file EMBR-24-e55895-s009.zip › Figure 4/4b/4b_Bag6_IP.tif]

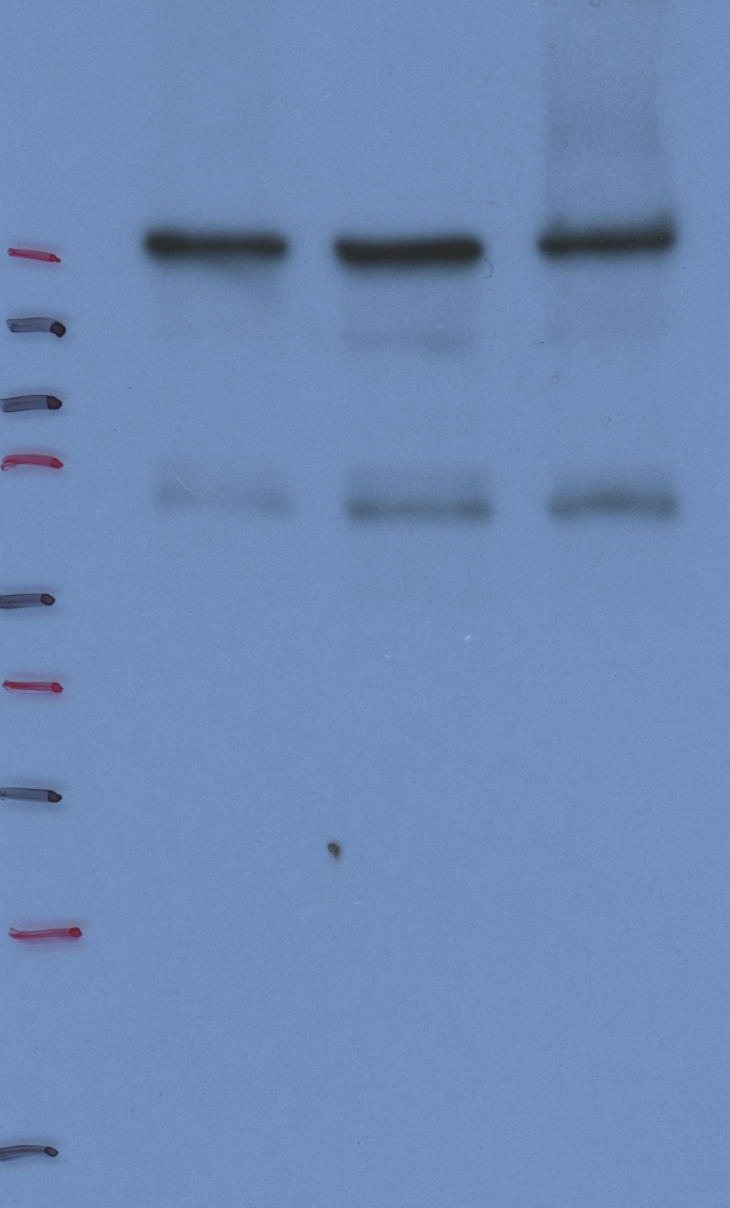

Supplement: Supplementary file 8 — Source Data for Figure 4 [file EMBR-24-e55895-s009.zip › Figure 4/4b/4b_UBR5_input.tif]

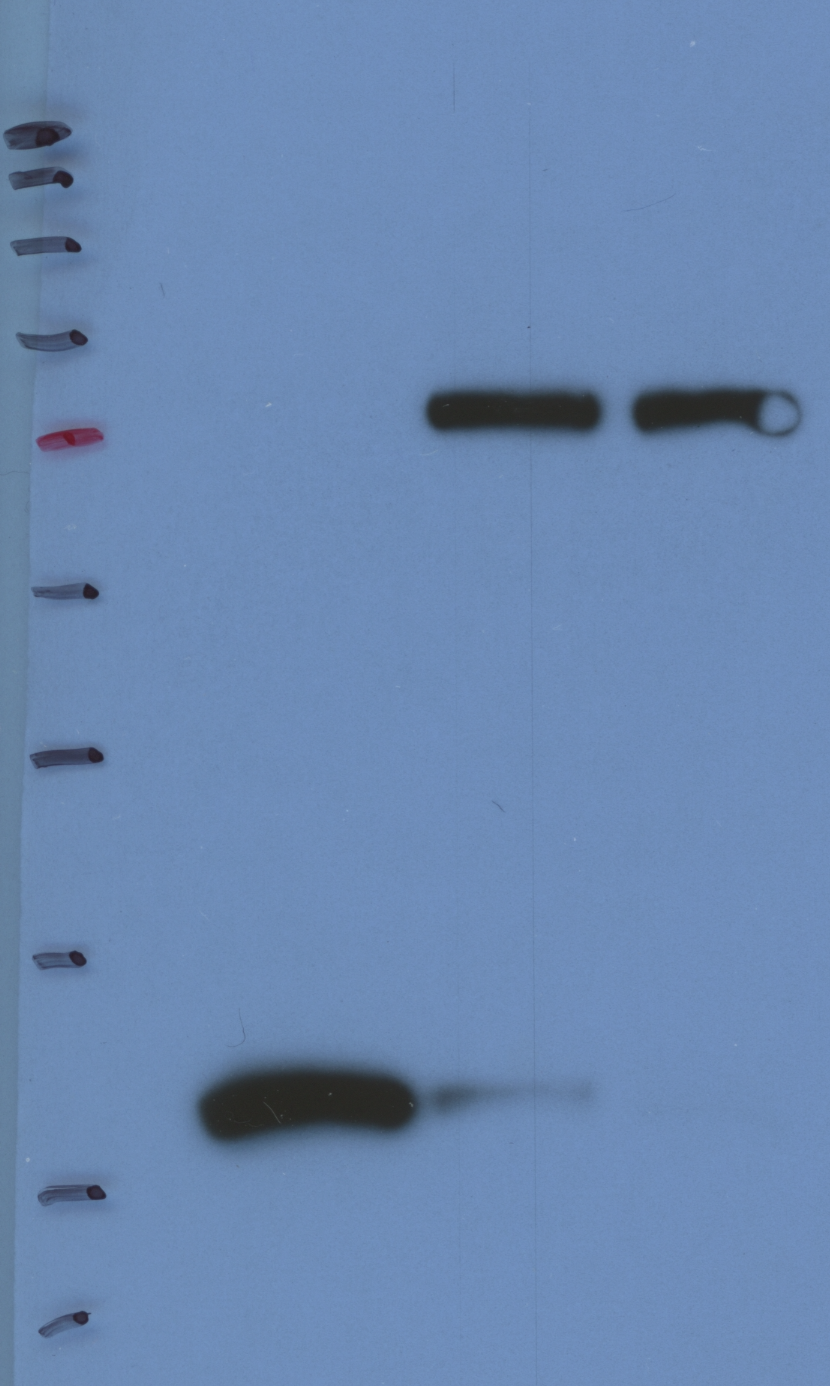

Supplement: Supplementary file 8 — Source Data for Figure 4 [file EMBR-24-e55895-s009.zip › Figure 4/4b/4b_GFP_IP.tif]

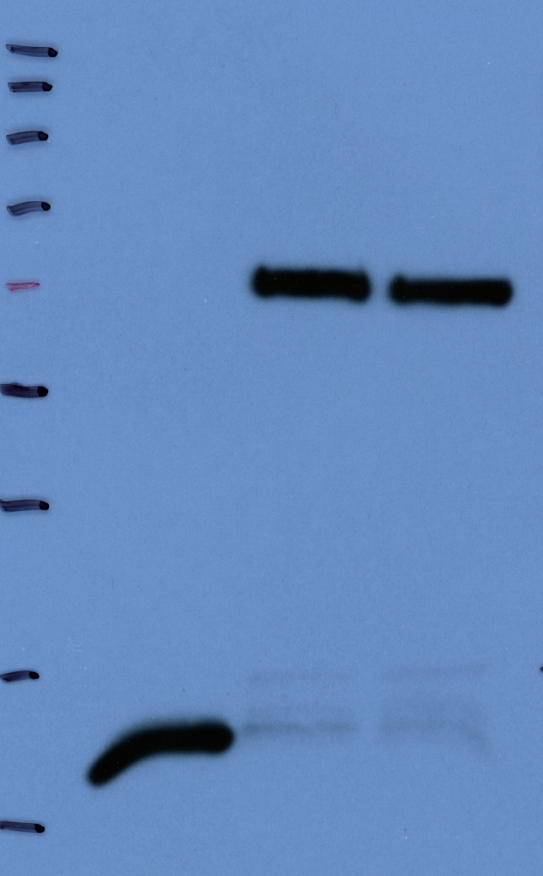

Supplement: Supplementary file 8 — Source Data for Figure 4 [file EMBR-24-e55895-s009.zip › Figure 4/4c/4c_GFP_IP.tif]

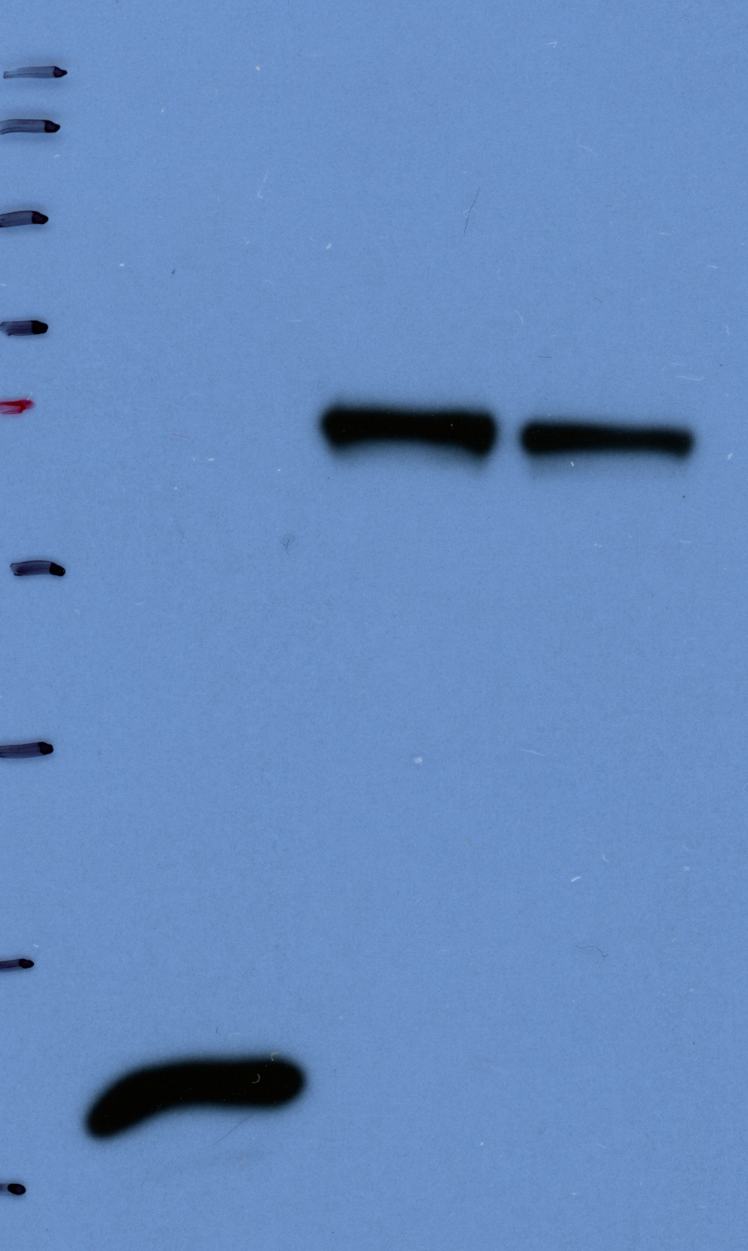

Supplement: Supplementary file 8 — Source Data for Figure 4 [file EMBR-24-e55895-s009.zip › Figure 4/4c/4c_GFP_input.tif]

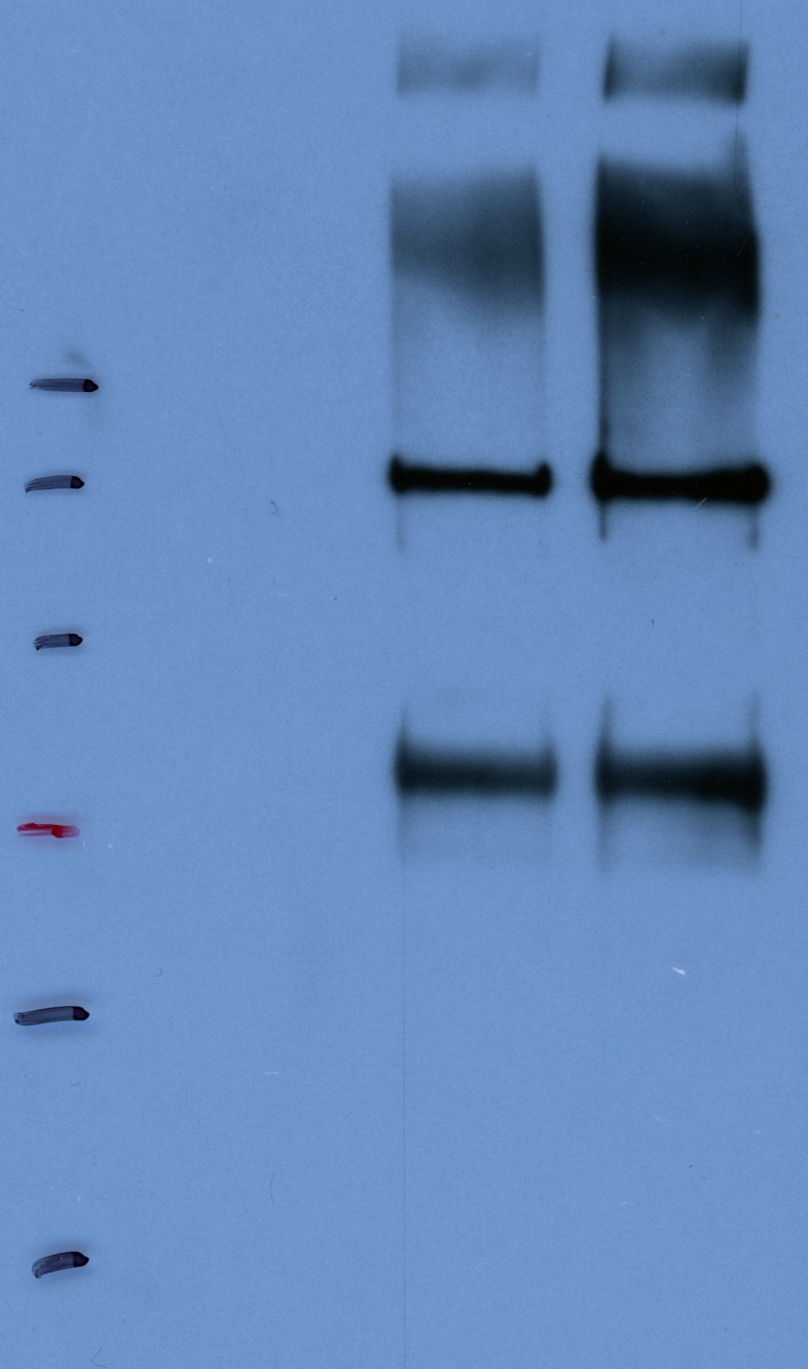

Supplement: Supplementary file 8 — Source Data for Figure 4 [file EMBR-24-e55895-s009.zip › Figure 4/4c/4c_FK2_IP.tif]

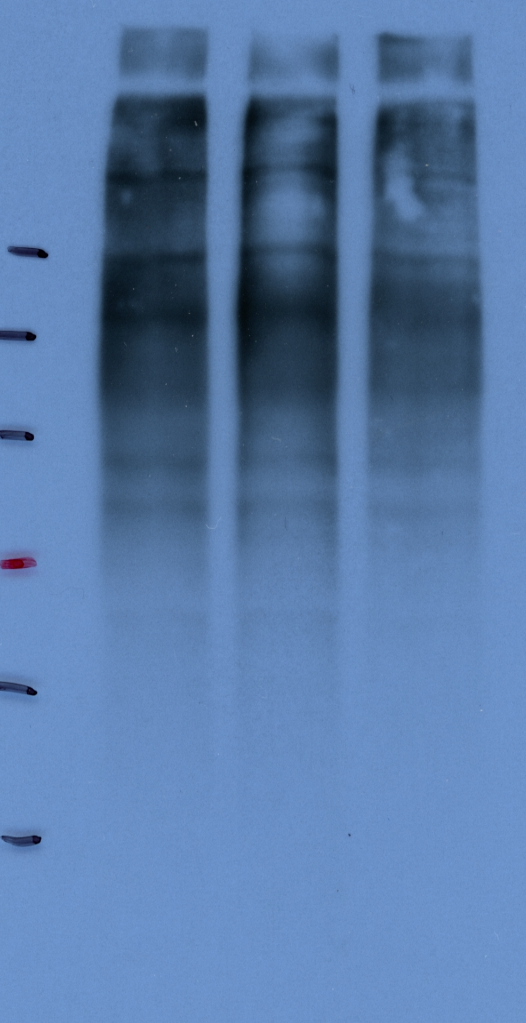

Supplement: Supplementary file 8 — Source Data for Figure 4 [file EMBR-24-e55895-s009.zip › Figure 4/4c/4c_FK2_input.tif]

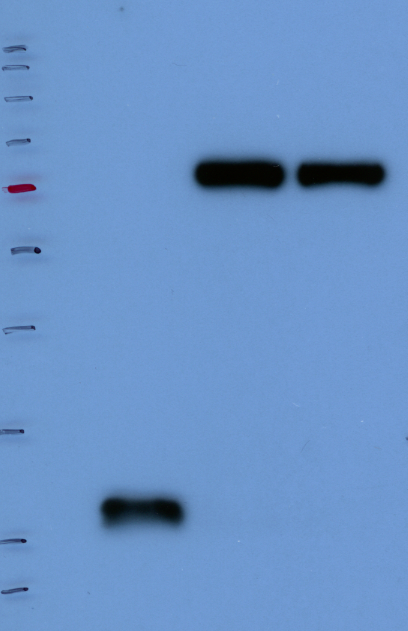

Supplement: Supplementary file 8 — Source Data for Figure 4 [file EMBR-24-e55895-s009.zip › Figure 4/4d/4d_GFP_IP.tif]

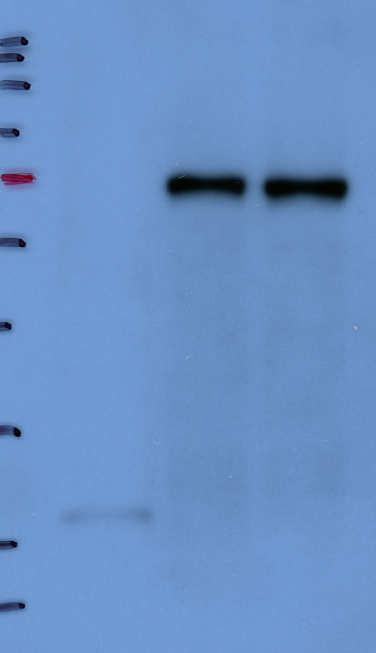

Supplement: Supplementary file 8 — Source Data for Figure 4 [file EMBR-24-e55895-s009.zip › Figure 4/4d/4d_GFP_input.tif]

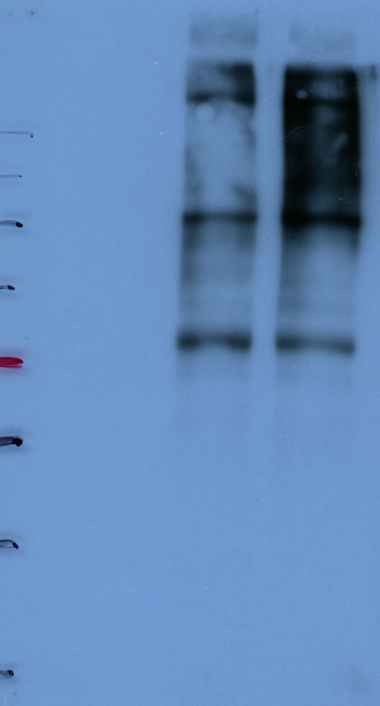

Supplement: Supplementary file 8 — Source Data for Figure 4 [file EMBR-24-e55895-s009.zip › Figure 4/4d/4d_FK2_IP.tif]

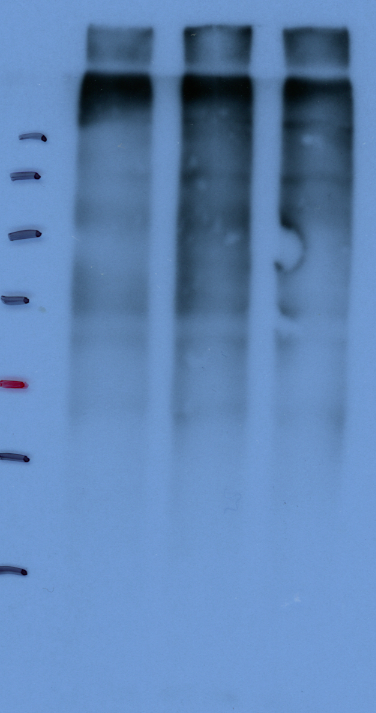

Supplement: Supplementary file 8 — Source Data for Figure 4 [file EMBR-24-e55895-s009.zip › Figure 4/4d/4d_FK2_input.tif]

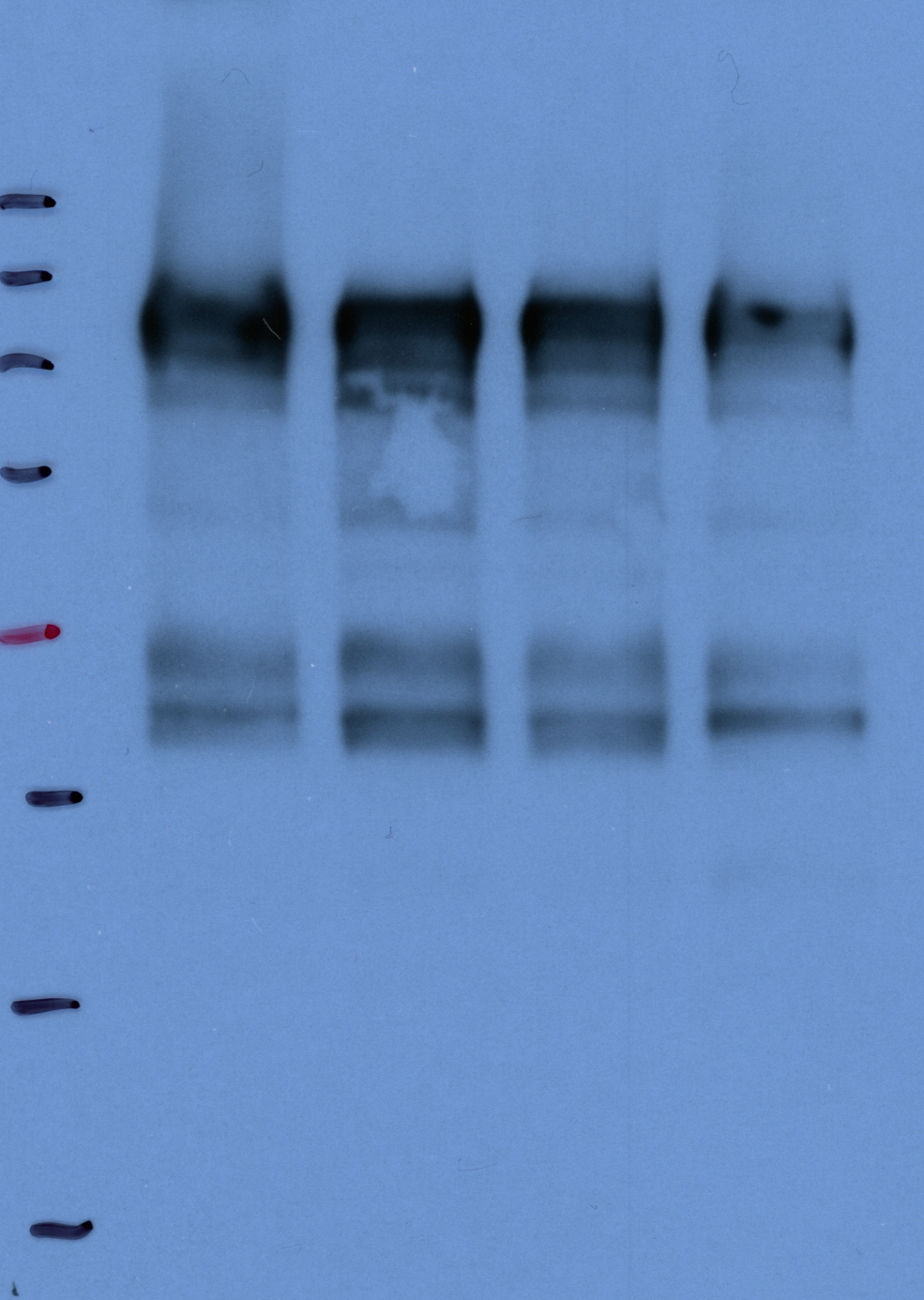

Supplement: Supplementary file 8 — Source Data for Figure 4 [file EMBR-24-e55895-s009.zip › Figure 4/4a/4a_Bag6_input.tif]

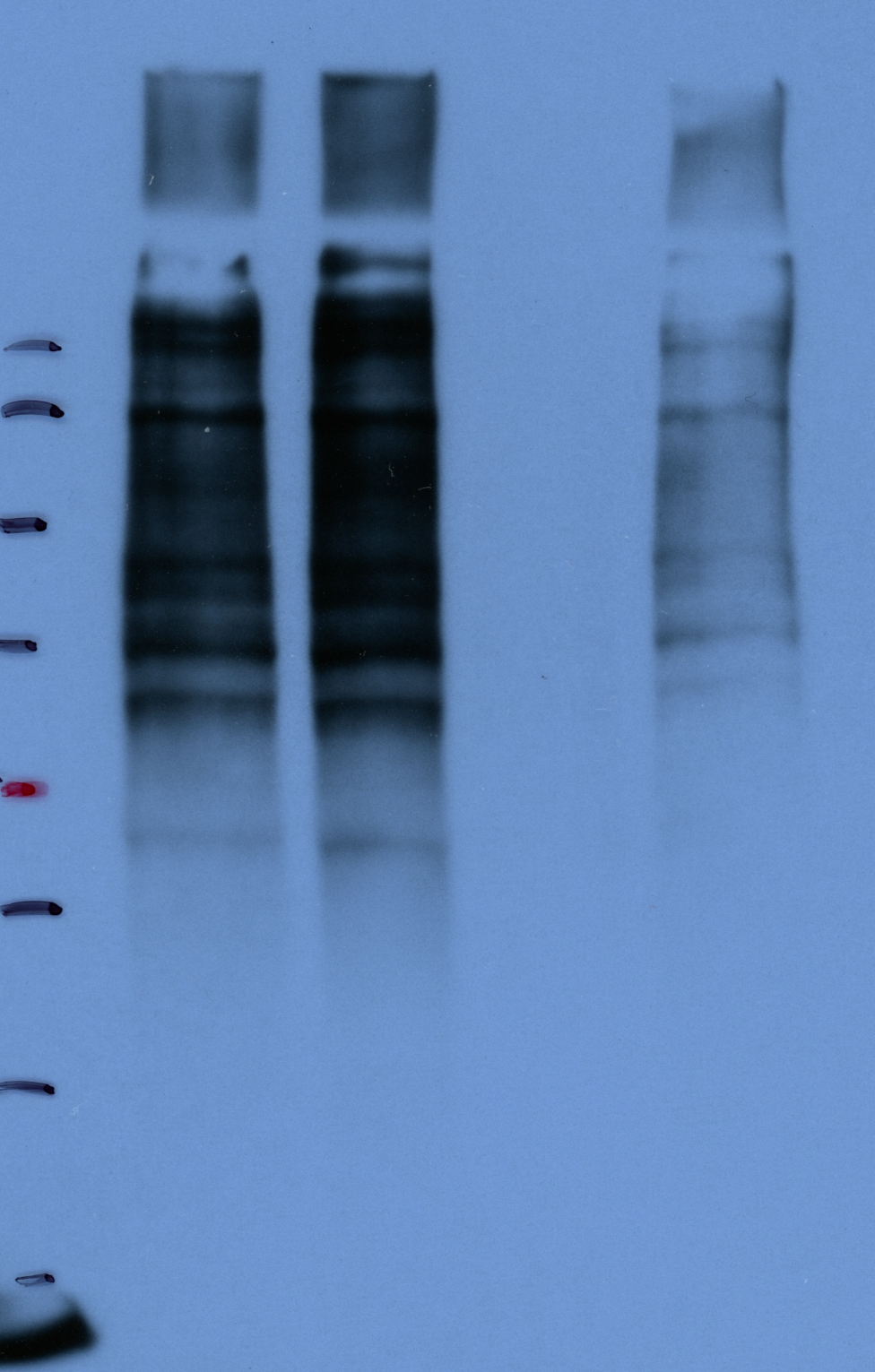

Supplement: Supplementary file 8 — Source Data for Figure 4 [file EMBR-24-e55895-s009.zip › Figure 4/4a/4a_FK2_input.tif]

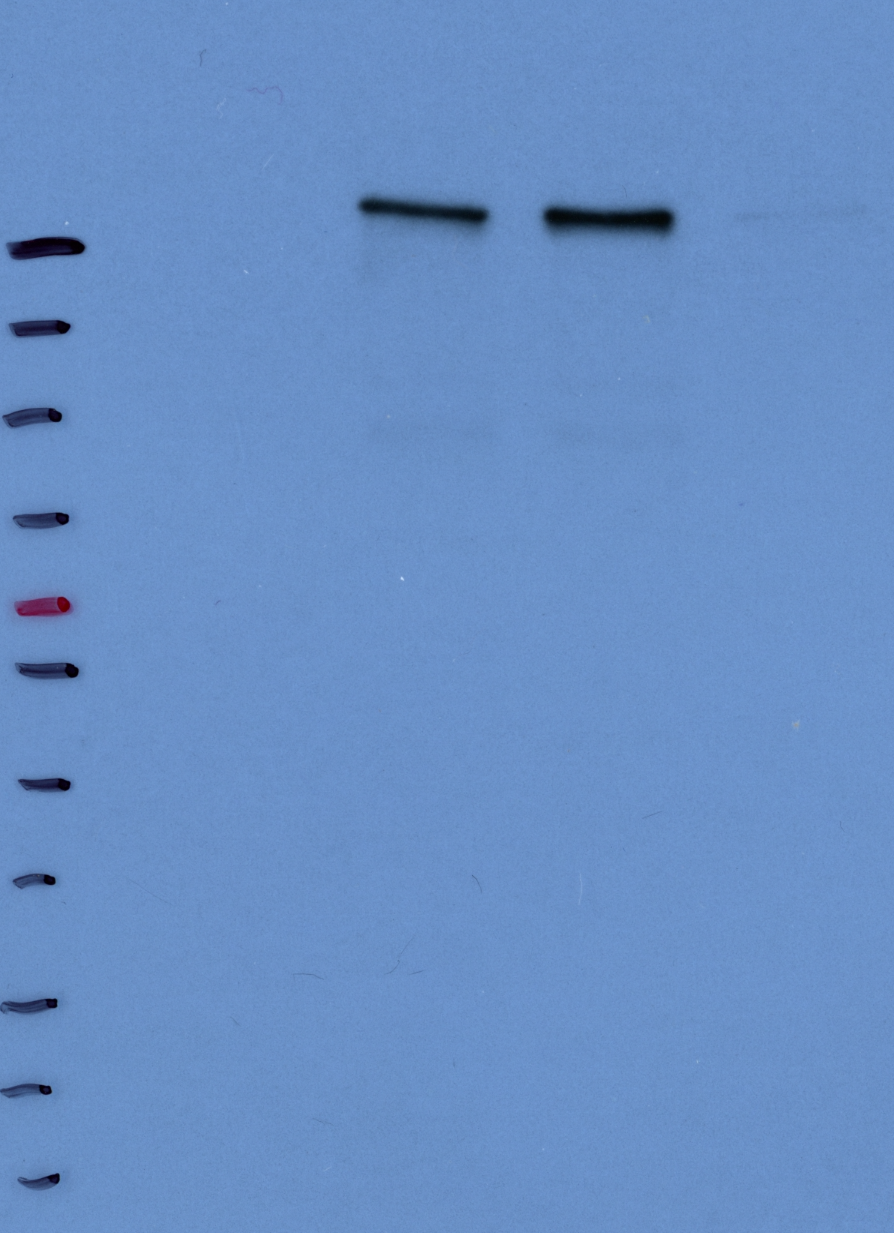

Supplement: Supplementary file 8 — Source Data for Figure 4 [file EMBR-24-e55895-s009.zip › Figure 4/4a/4a_UBR5_IP.tif]

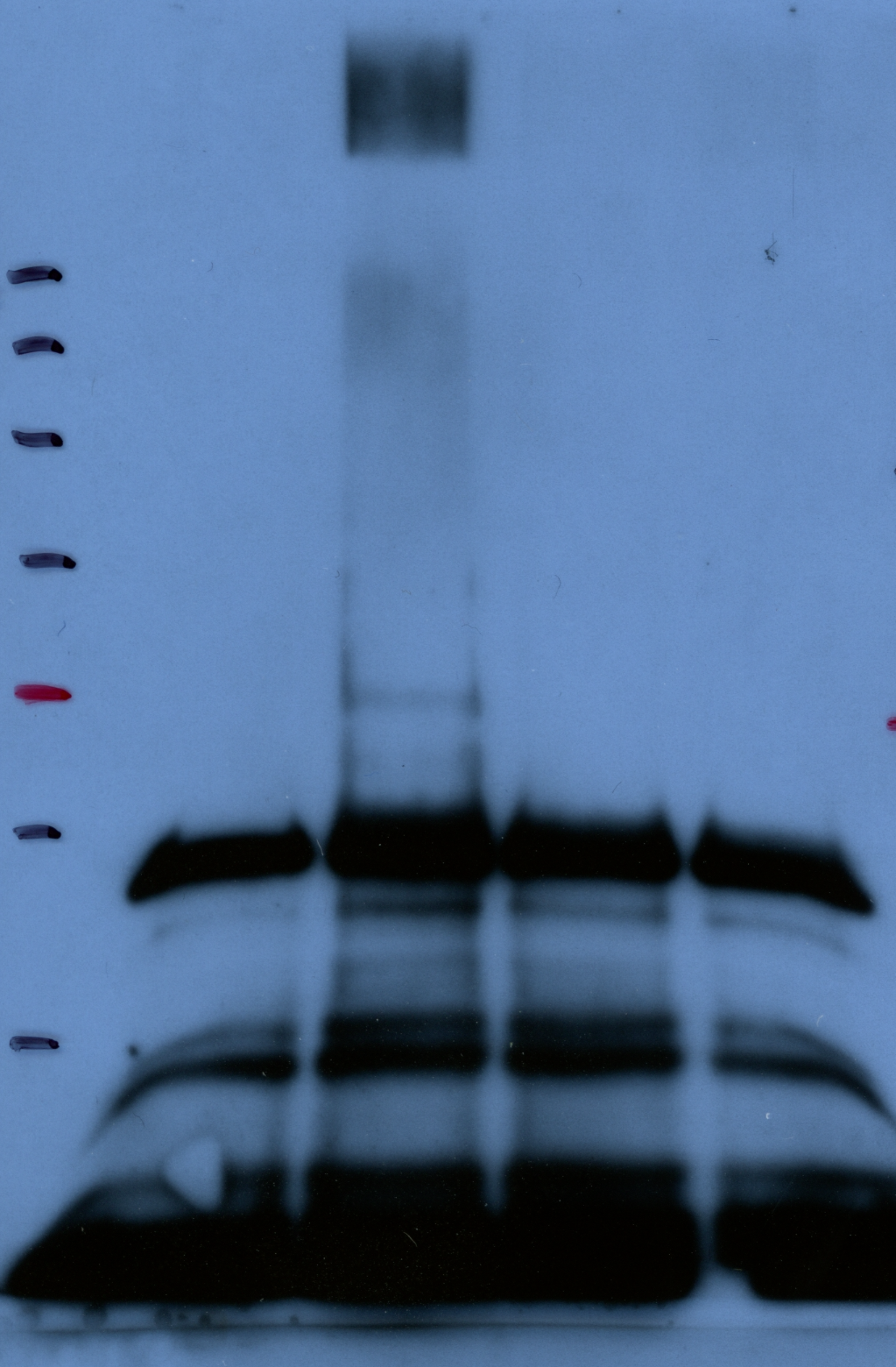

Supplement: Supplementary file 8 — Source Data for Figure 4 [file EMBR-24-e55895-s009.zip › Figure 4/4a/4a_FK2_IP.tif]
