# Supplementary figures and images for: C9orf72 protein quality control by UBR5‐mediated heterotypic ubiquitin chains (part 2 of 2)
Source: EMBO Rep. 2023 Jun 15;24(8):e55895. doi: 10.15252/embr.202255895 (PMC10398660; doi:10.15252/embr.202255895)

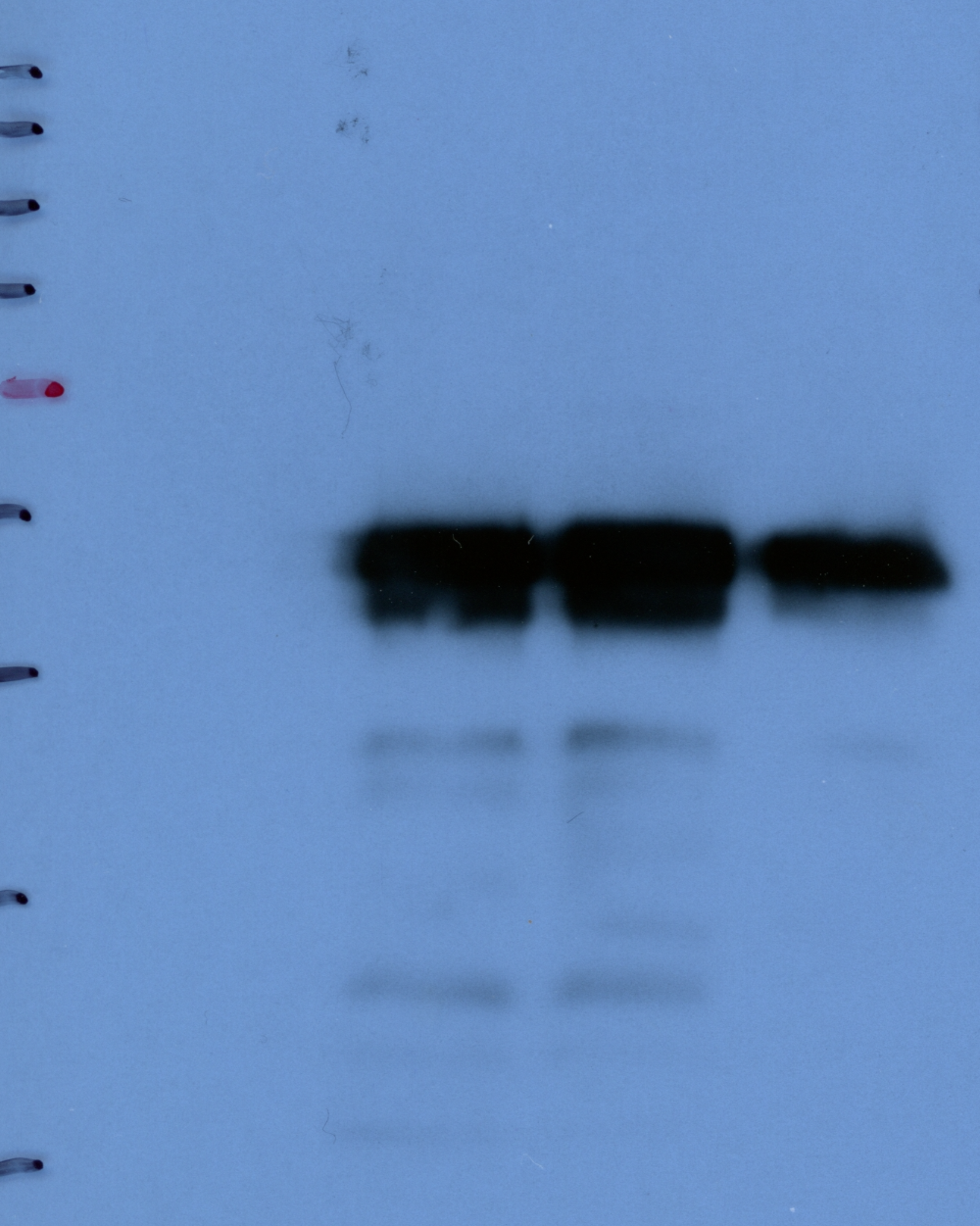

Supplement: Supplementary file 8 — Source Data for Figure 4 [file EMBR-24-e55895-s009.zip › Figure 4/4a/4a_HA_IP.tif]

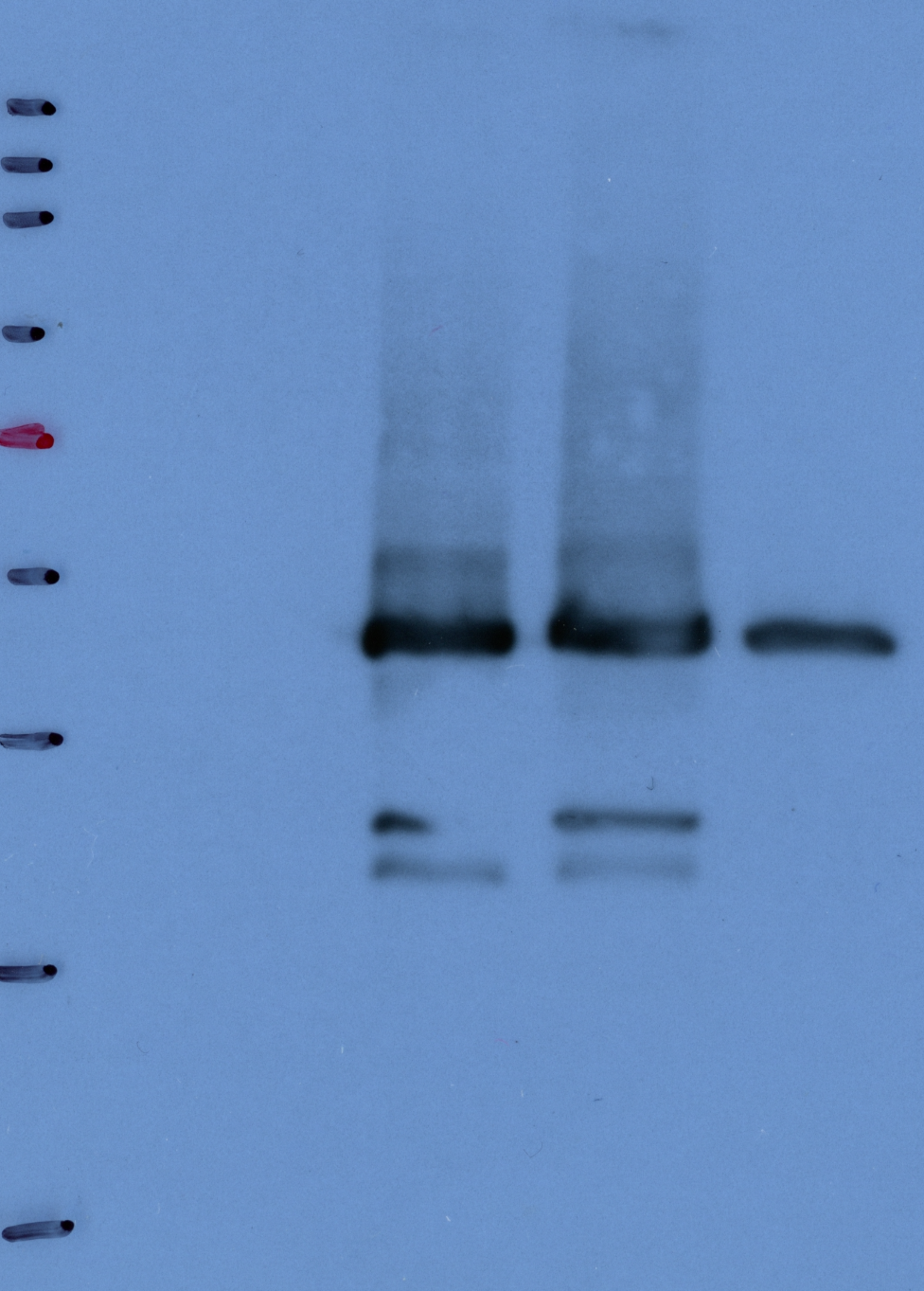

Supplement: Supplementary file 8 — Source Data for Figure 4 [file EMBR-24-e55895-s009.zip › Figure 4/4a/4a_HA_Input.tif]

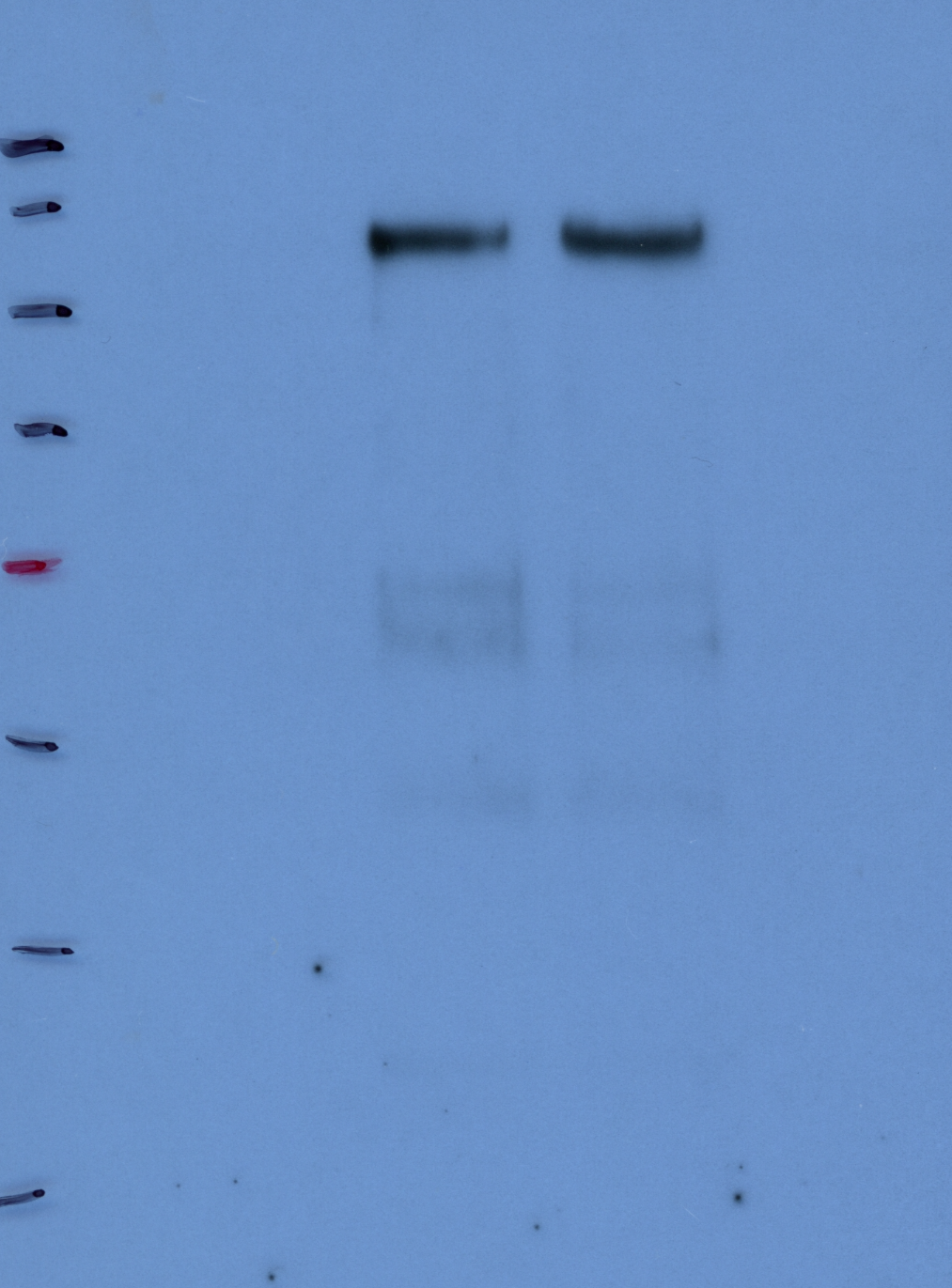

Supplement: Supplementary file 8 — Source Data for Figure 4 [file EMBR-24-e55895-s009.zip › Figure 4/4a/4a_Bag6_IP.tif]

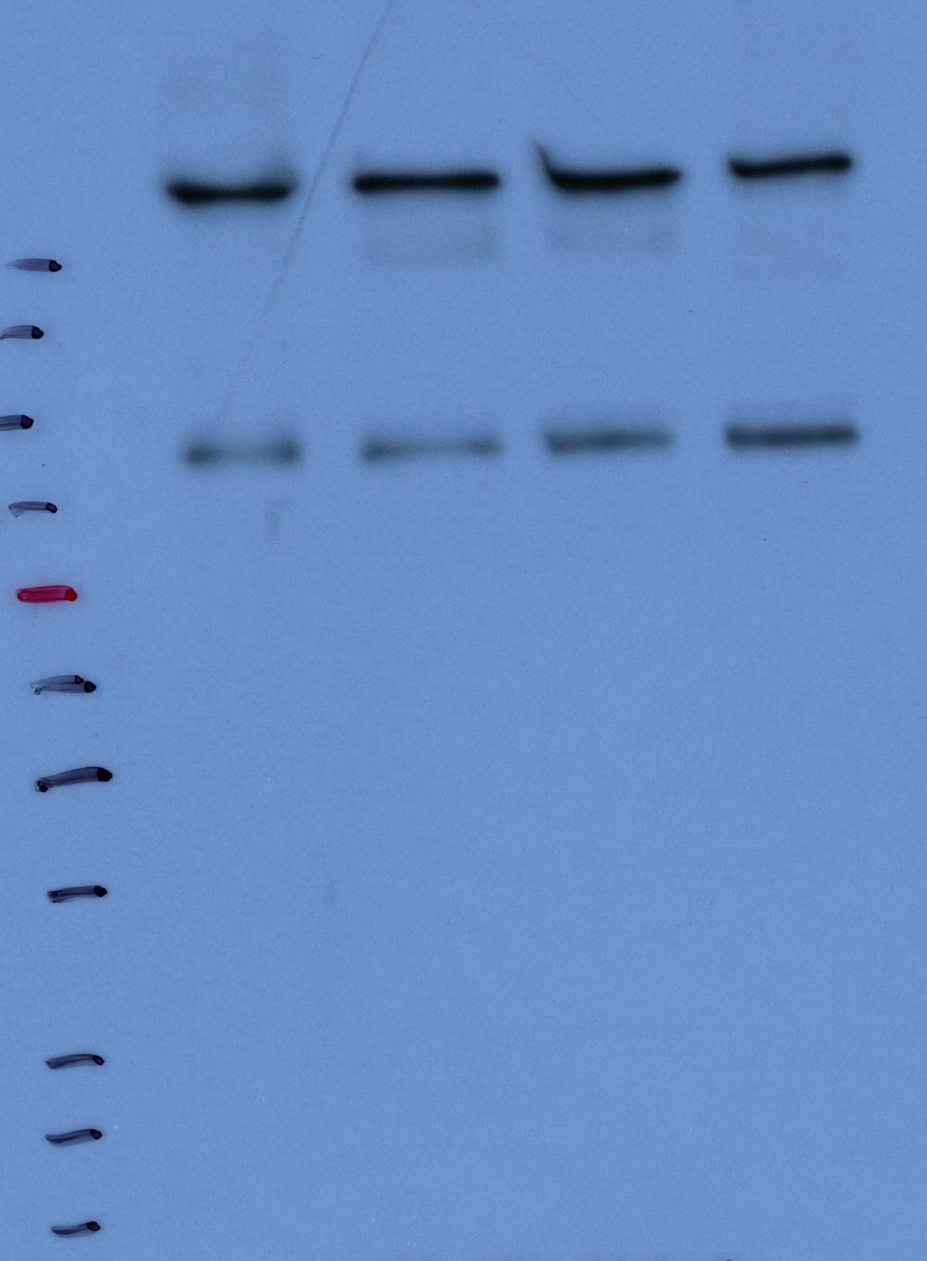

Supplement: Supplementary file 8 — Source Data for Figure 4 [file EMBR-24-e55895-s009.zip › Figure 4/4a/4a_UBR5_input.tif]

Figure 5A

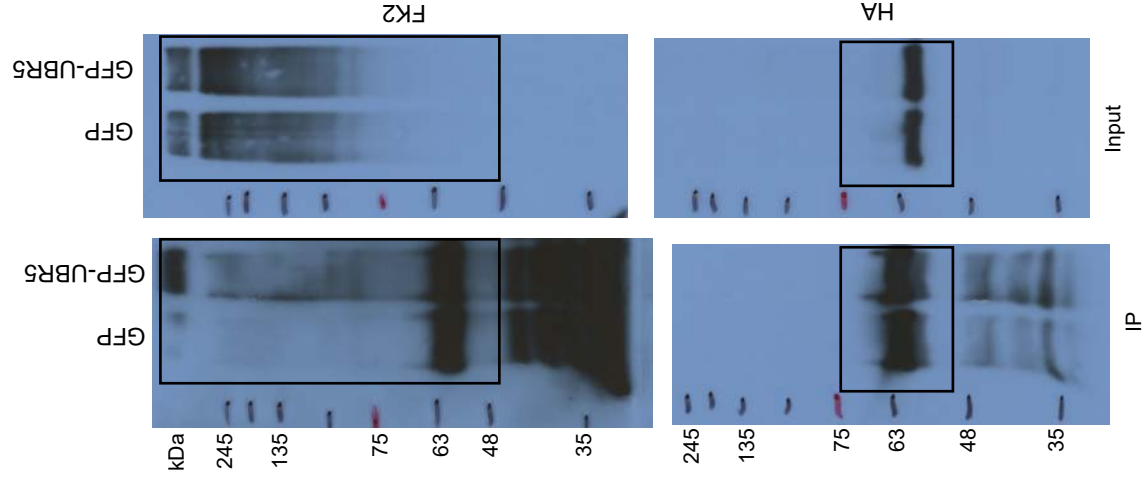

Figure 5B

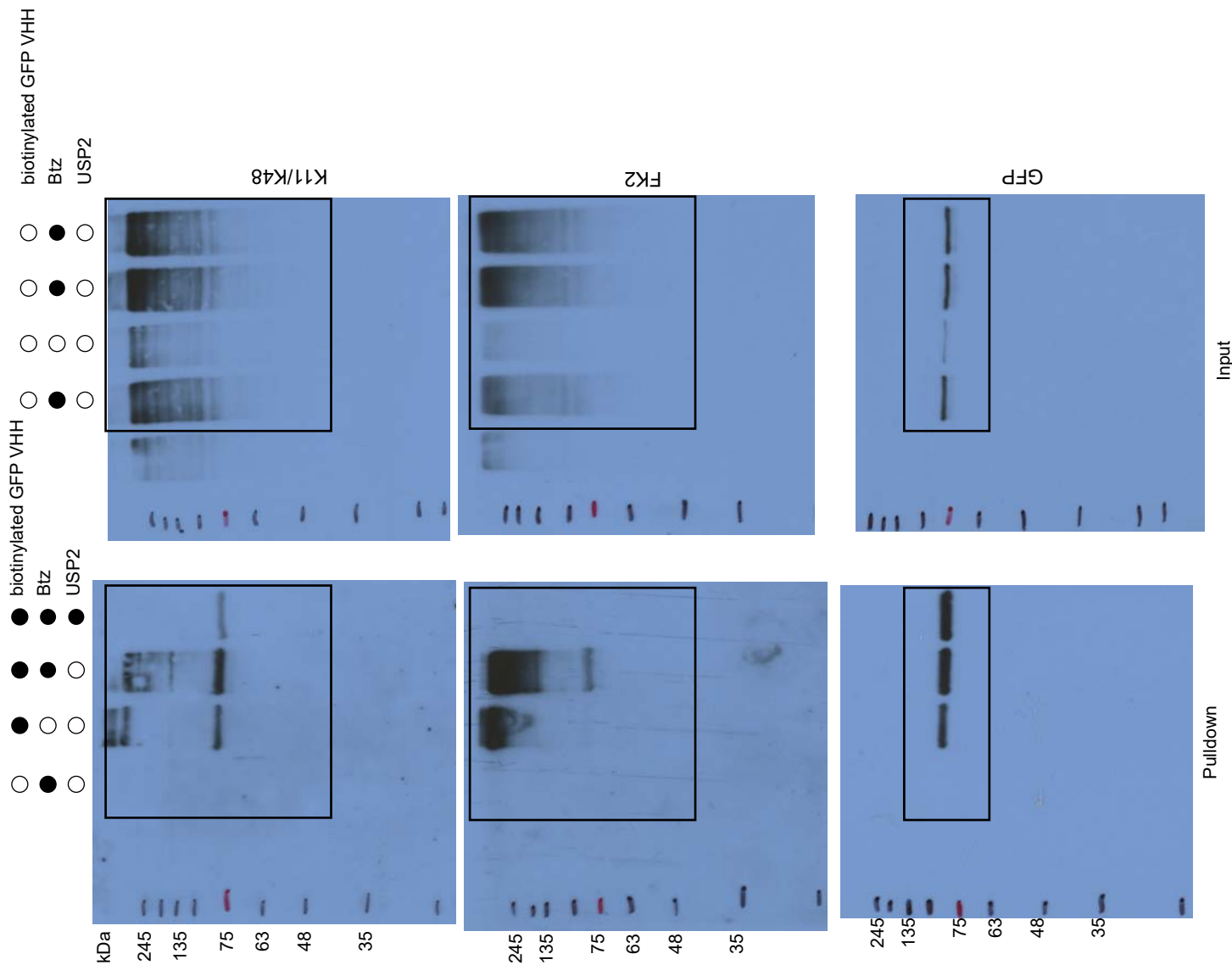

293T SMCR8 KO eGFP-C9orf72

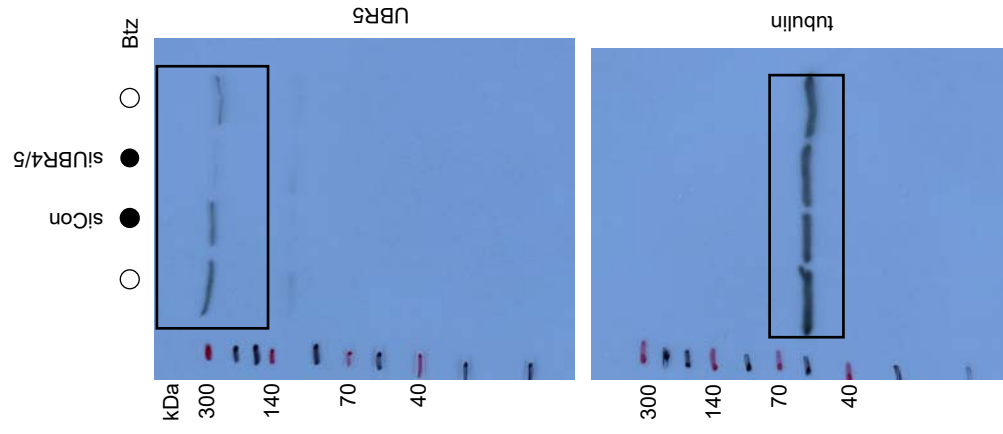

Supplement: Supplementary file 9 — Source Data for Figure 5 [file EMBR-24-e55895-s005.zip › Figure 5/Figure 5.pdf]

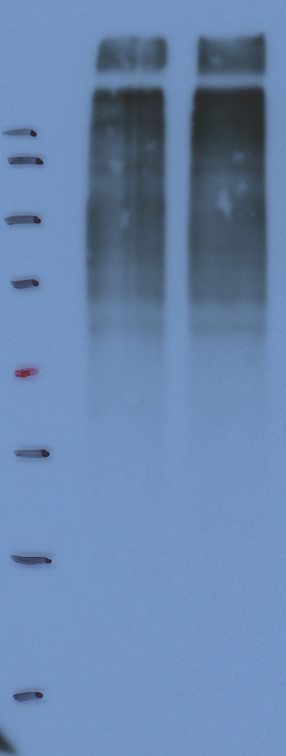

Supplement: Supplementary file 9 — Source Data for Figure 5 [file EMBR-24-e55895-s005.zip › Figure 5/5a/5a_FK2_input.tif]

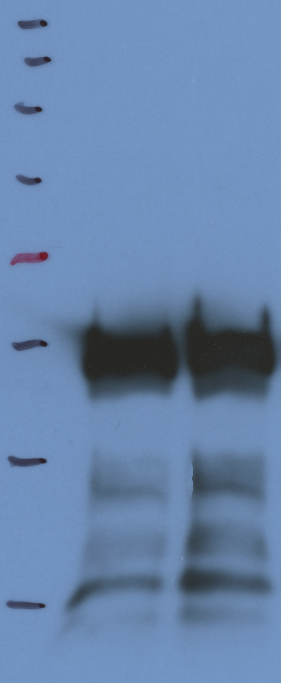

Supplement: Supplementary file 9 — Source Data for Figure 5 [file EMBR-24-e55895-s005.zip › Figure 5/5a/5a_HA_IP.tif]

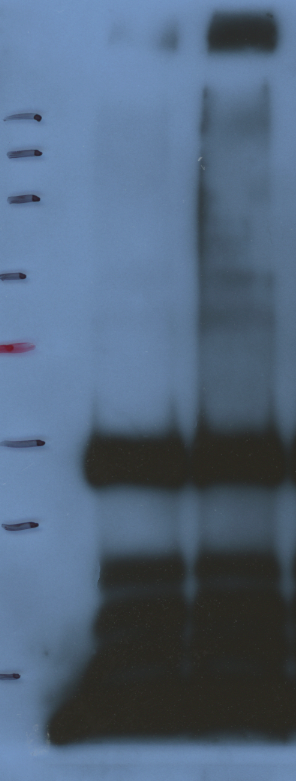

Supplement: Supplementary file 9 — Source Data for Figure 5 [file EMBR-24-e55895-s005.zip › Figure 5/5a/5a_FK2_IP.tif]

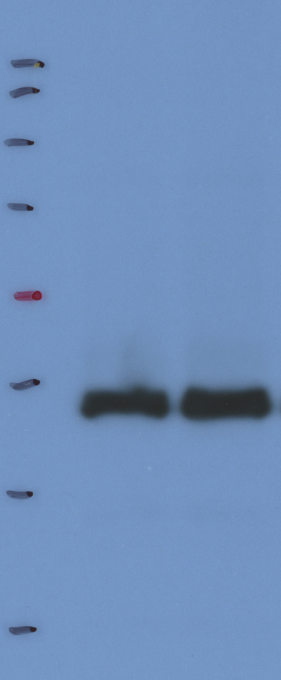

Supplement: Supplementary file 9 — Source Data for Figure 5 [file EMBR-24-e55895-s005.zip › Figure 5/5a/5a_HA_input.tif]

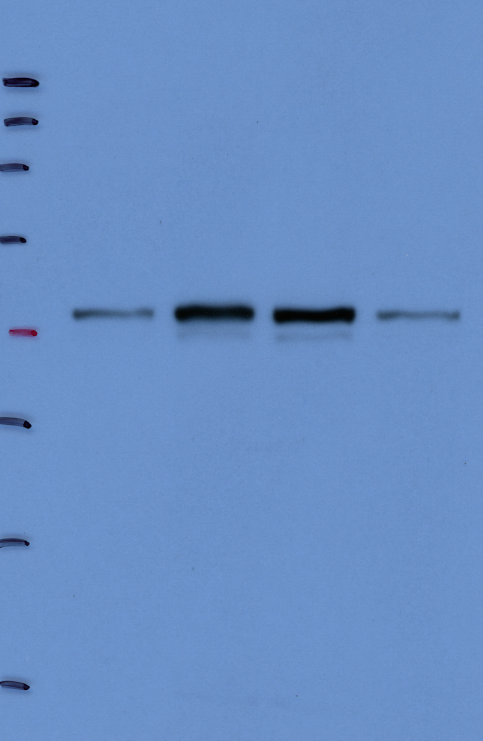

Supplement: Supplementary file 9 — Source Data for Figure 5 [file EMBR-24-e55895-s005.zip › Figure 5/5c/5c_GFP_input.tif]

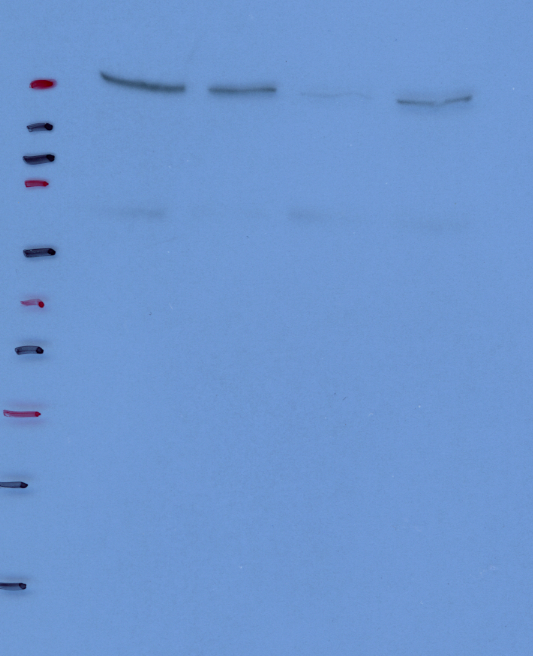

Supplement: Supplementary file 9 — Source Data for Figure 5 [file EMBR-24-e55895-s005.zip › Figure 5/5c/5c_UBR5.tif]

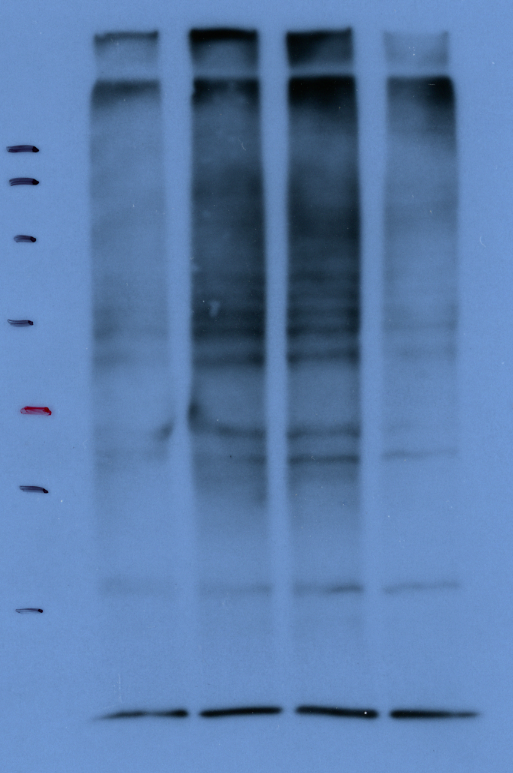

Supplement: Supplementary file 9 — Source Data for Figure 5 [file EMBR-24-e55895-s005.zip › Figure 5/5c/5c_K11K48_input.tif]

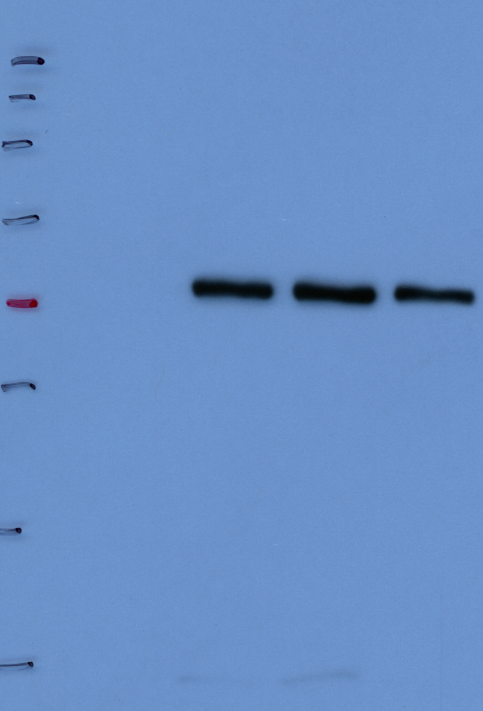

Supplement: Supplementary file 9 — Source Data for Figure 5 [file EMBR-24-e55895-s005.zip › Figure 5/5c/5c_GFP_IP.tif]

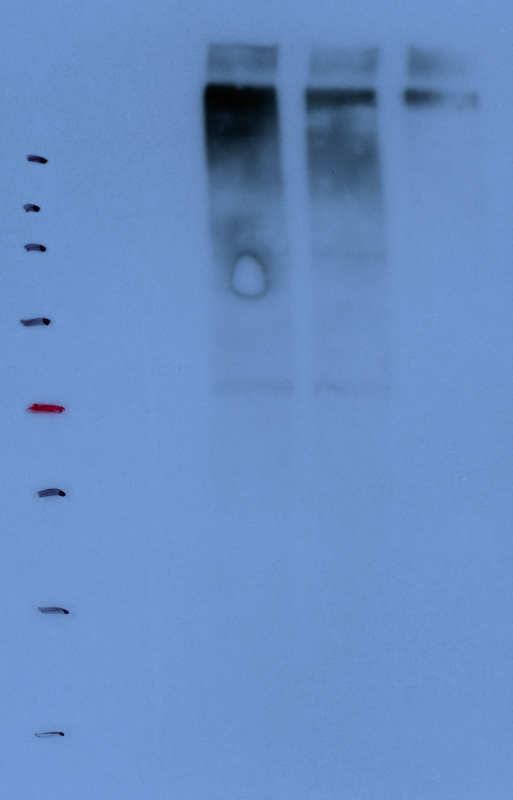

Supplement: Supplementary file 9 — Source Data for Figure 5 [file EMBR-24-e55895-s005.zip › Figure 5/5c/5c_K11K48_IP.tif]

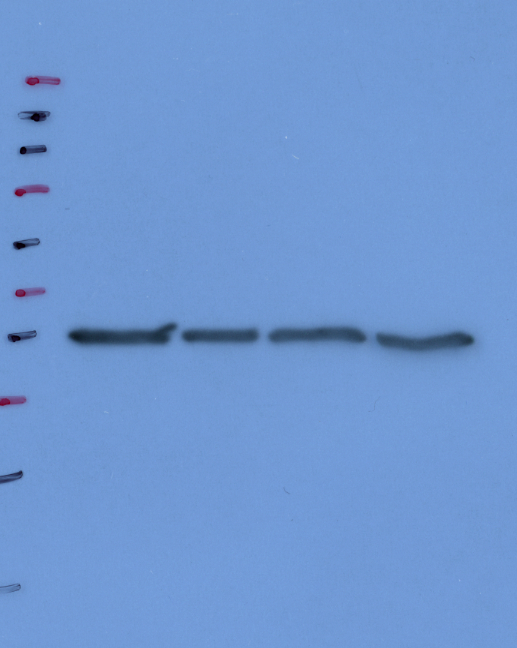

Supplement: Supplementary file 9 — Source Data for Figure 5 [file EMBR-24-e55895-s005.zip › Figure 5/5c/5c_tubulin.tif]

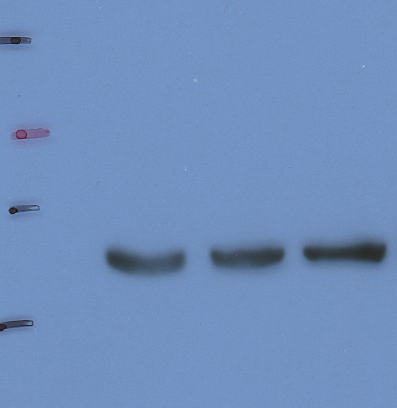

Supplement: Supplementary file 9 — Source Data for Figure 5 [file EMBR-24-e55895-s005.zip › Figure 5/5d/5d_tubulin_n1.tif]

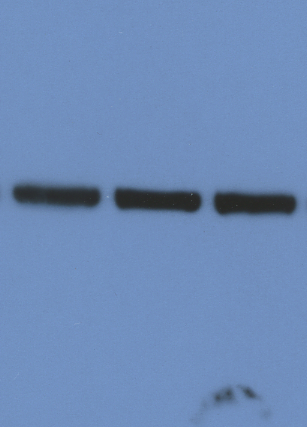

Supplement: Supplementary file 9 — Source Data for Figure 5 [file EMBR-24-e55895-s005.zip › Figure 5/5d/5d_tubulin_n2.tif]

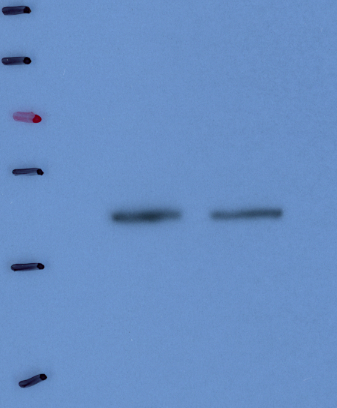

Supplement: Supplementary file 9 — Source Data for Figure 5 [file EMBR-24-e55895-s005.zip › Figure 5/5d/5d_tubulin_n3.tif]

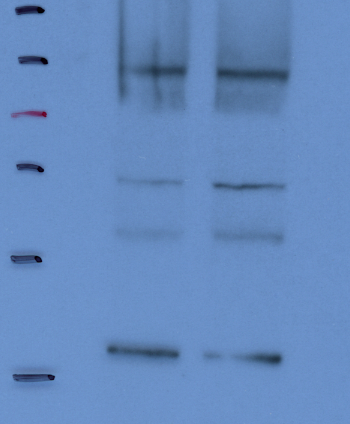

Supplement: Supplementary file 9 — Source Data for Figure 5 [file EMBR-24-e55895-s005.zip › Figure 5/5d/5d_C9orf72_n3.tif]

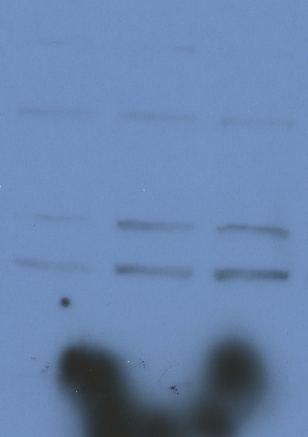

Supplement: Supplementary file 9 — Source Data for Figure 5 [file EMBR-24-e55895-s005.zip › Figure 5/5d/5d_C9orf72_n2.tif]

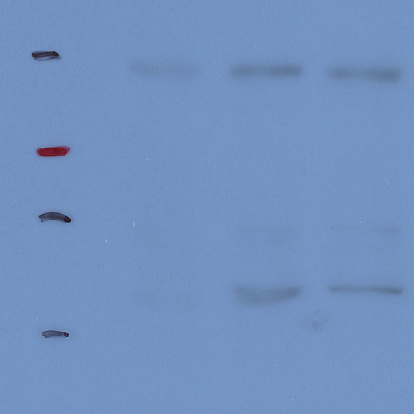

Supplement: Supplementary file 9 — Source Data for Figure 5 [file EMBR-24-e55895-s005.zip › Figure 5/5d/5d_C9orf72_n1.tif]

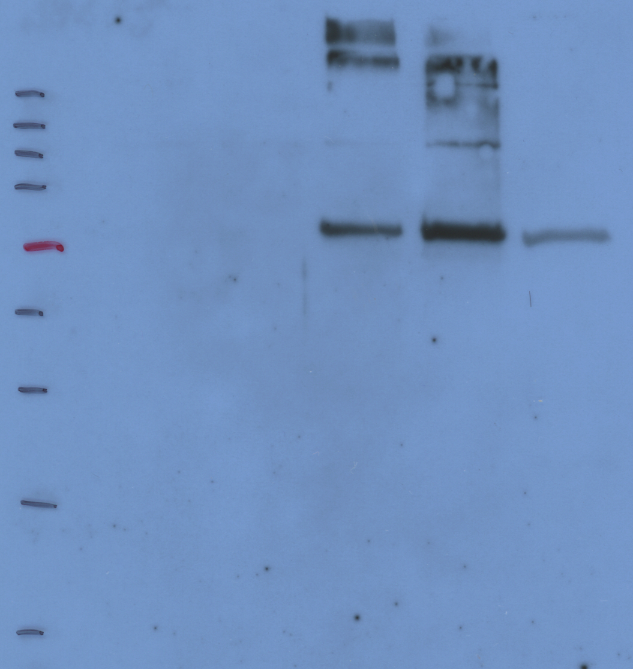

Supplement: Supplementary file 9 — Source Data for Figure 5 [file EMBR-24-e55895-s005.zip › Figure 5/5b/5b_K11K48_IP.tif]

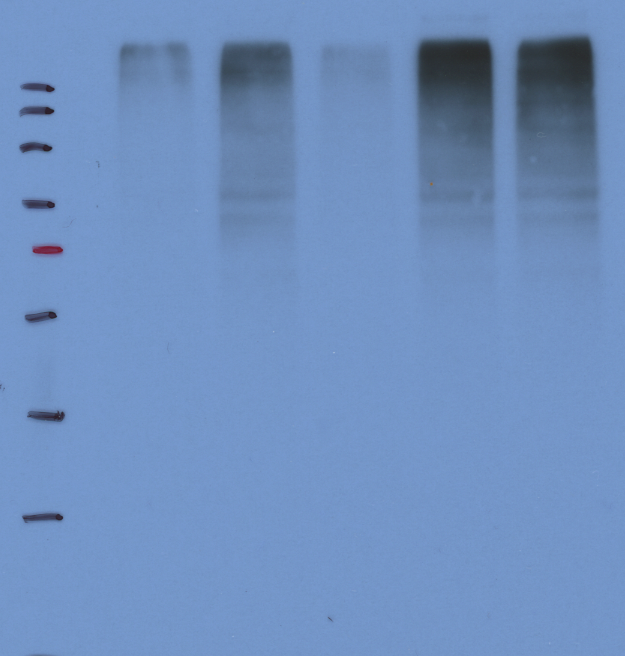

Supplement: Supplementary file 9 — Source Data for Figure 5 [file EMBR-24-e55895-s005.zip › Figure 5/5b/5b_FK2_input.tif]

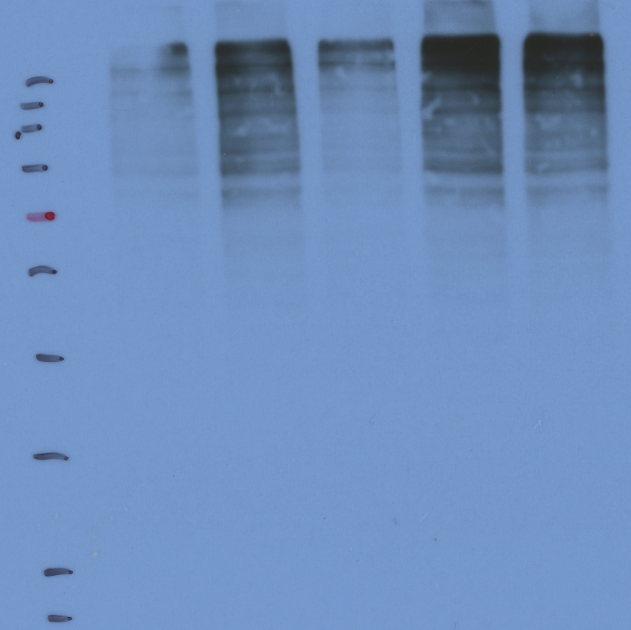

Supplement: Supplementary file 9 — Source Data for Figure 5 [file EMBR-24-e55895-s005.zip › Figure 5/5b/5b_K11K48_input.tif]

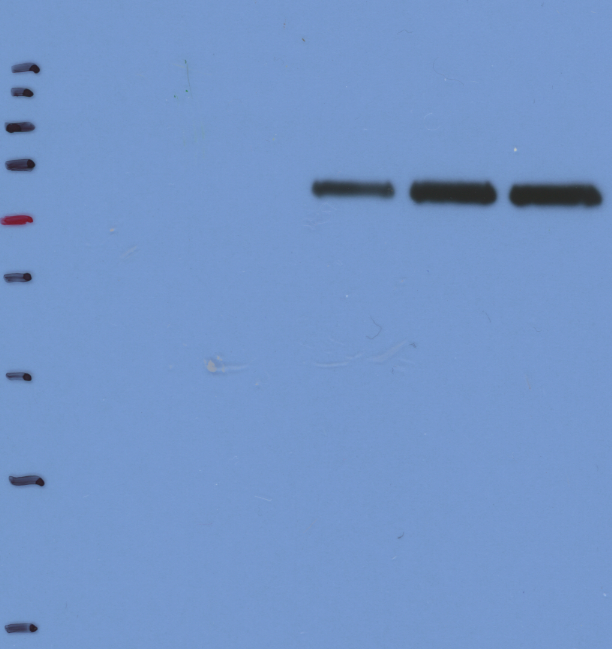

Supplement: Supplementary file 9 — Source Data for Figure 5 [file EMBR-24-e55895-s005.zip › Figure 5/5b/5b_GFP_IP.tif]

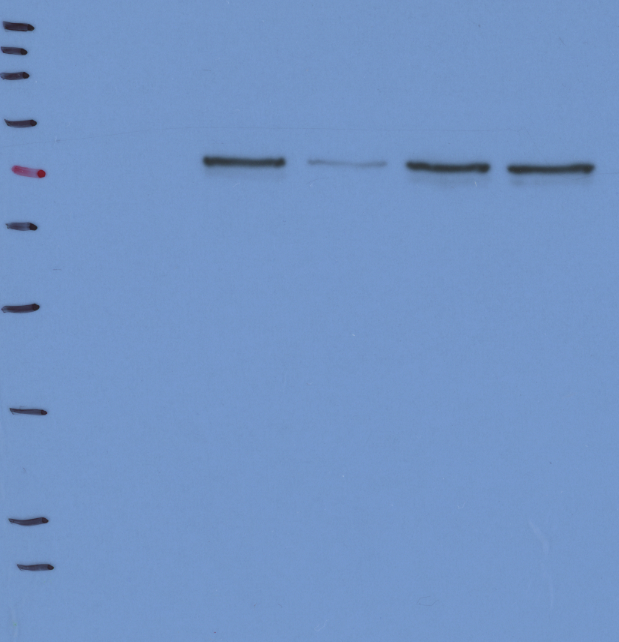

Supplement: Supplementary file 9 — Source Data for Figure 5 [file EMBR-24-e55895-s005.zip › Figure 5/5b/5b_GFP_input.tif]

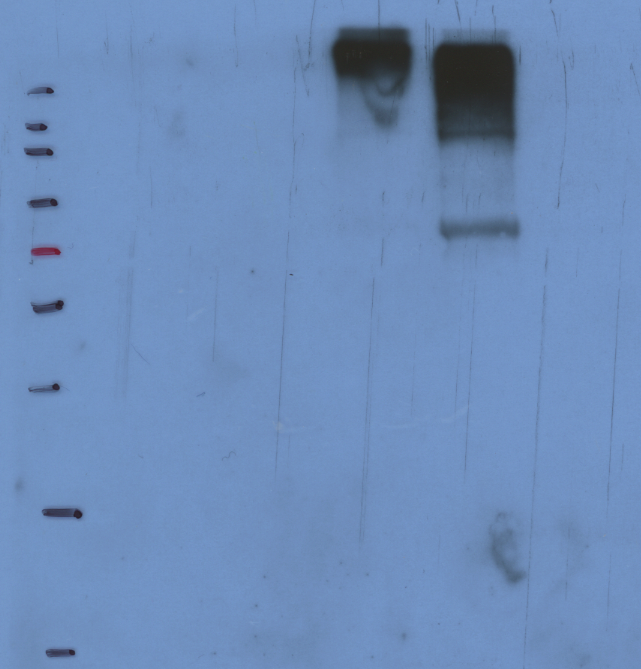

Supplement: Supplementary file 9 — Source Data for Figure 5 [file EMBR-24-e55895-s005.zip › Figure 5/5b/5b_FK2_IP.tif]
