# Supplementary material for: C9orf72 protein quality control by UBR5‐mediated heterotypic ubiquitin chains
Source: EMBO Rep. 2023 Jun 15;24(8):e55895. doi: 10.15252/embr.202255895 (PMC10398660; doi:10.15252/embr.202255895)
Supplement: Supplementary file 10 [file EMBR-24-e55895-s008.pdf]

EMBO reports

C9orf72 protein quality control by UBR5-mediated heterotypic ubiquitin chains

Julia Jülg, Dieter Edbauer, and Christian Behrends
DOI: 10.15252/embr.202255895

Corresponding author(s): Christian Behrends (Christian.Behrends@mail03.med.uni-muenchen.de)

Review Timeline:

Submission Date:	3rd Aug 22
Editorial Decision:	29th Sep 22
Revision Received:	8th Feb 23
Editorial Decision:	3rd May 23
Revision Received:	10th May 23
Accepted:	26th May 23

Editor: Martina Rembold

Transaction Report:

(Note: With the exception of the correction of typographical or spelling errors that could be a source of ambiguity, letters and reports are not edited. Depending on transfer agreements, referee reports obtained elsewhere may or may not be included in this compilation. Referee reports are anonymous unless the Referee chooses to sign their reports.)

1st Editorial Decision 29th Sep 2022

Dear Prof. Behrends

Thank you once more for the submission of your manuscript to EMBO reports and thank you also for providing feedback on the concerns raised by referee 2.

I had informed you that the referee's opinions on your manuscript were not in agreement and that referee 2 raised important concerns challenging the main conclusions of your study. As I indicated, I am open to consider your manuscript without an in-depth investigation of the mechanism (How is C9orf72 with repeat-expansions controlled? Does Ubr5 depletion affect signaling by the C9orf72/SMCR8 complex? How is SMCR8 degraded?) but note that the data reporting on the degradation of uncomplexed C9orf72 by Ubr5 need to be solid and convincing. You have now outlined that and how you aim to address the referee concerns and based on your point-by-point response, I have decided to invite you to revise your manuscript - along the lines outlined in your revision plan regarding the points raised by referee 2 - for potential publication in EMBO reports.

Please address all referee concerns in a complete point-by-point response. Acceptance of the manuscript will depend on a positive outcome of a second round of review. It is EMBO reports policy to allow a single round of revision only and acceptance or rejection of the manuscript will therefore depend on the completeness of your responses included in the next, final version of the manuscript.

We realize that it is difficult to revise to a specific deadline. In the interest of protecting the conceptual advance provided by the work, we recommend a revision within 3 months (December 29th). Please discuss the revision progress ahead of this time with me if you require more time to complete the revisions.

You can either publish the study as a short report or as a full article. For short reports, the revised manuscript should not exceed 27,000 characters (including spaces but excluding materials & methods and references) and 5 main plus 5 expanded view figures. The results and discussion sections must further be combined, which will help to shorten the manuscript text by eliminating some redundancy that is inevitable when discussing the same experiments twice. For a normal article there are no length limitations, but it should have more than 5 main figures and the results and discussion sections must be separate. In both cases, the entire materials and methods must be included in the main manuscript file.

I list below all information that is important for the resubmission. Let me add here that Table S2 should be called Dataset EV2 and the legend should be added to a separate tab within the same file.

*****IMPORTANT NOTE:
We perform an initial quality control of all revised manuscripts before re-review. Your manuscript will FAIL this control and the handling will be DELAYED if the following APPLIES:

1) A data availability section providing access to data deposited in public databases is missing. If you have not deposited any data, please add a sentence to the data availability section that explains that.

2) Your manuscript contains statistics and error bars based on n=2. Please use scatter blots in these cases. No statistics should be calculated if n=2.

When submitting your revised manuscript, please carefully review the instructions that follow below. Failure to include requested items will delay the evaluation of your revision.*****

When submitting your revised manuscript, we will require:

1) a .docx formatted version of the manuscript text (including legends for main figures, EV figures and tables). Please make sure that the changes are highlighted to be clearly visible.

2) individual production quality figure files as .eps, .tif, .jpg (one file per figure).
Please download our Figure Preparation Guidelines (figure preparation pdf) from our Author Guidelines pages https://www.embopress.org/page/journal/14693178/authorguide for more info on how to prepare your figures.

3) a .docx formatted letter INCLUDING the reviewers' reports and your detailed point-by-point responses to their comments. As part of the EMBO Press transparent editorial process, the point-by-point response is part of the Review Process File (RPF), which will be published alongside your paper.

4) a complete author checklist, which you can download from our author guidelines (). Please insert information in the checklist that is also reflected in the manuscript. The completed author checklist will also be part of the RPF.

5) Please note that all corresponding authors are required to supply an ORCID ID for their name upon submission of a revised manuscript (). Please find instructions on how to link your ORCID ID to your account in our manuscript tracking system in our Author guidelines
()

6) We replaced Supplementary Information with Expanded View (EV) Figures and Tables that are collapsible/expandable online. A maximum of 5 EV Figures can be typeset. EV Figures should be cited as 'Figure EV1, Figure EV2" etc... in the text and their respective legends should be included in the main text after the legends of regular figures.

- For the figures that you do NOT wish to display as Expanded View figures, they should be bundled together with their legends in a single PDF file called *Appendix*, which should start with a short Table of Content. Appendix figures should be referred to in the main text as: "Appendix Figure S1, Appendix Figure S2" etc. See detailed instructions regarding expanded view here:

- Additional Tables/Datasets should be labeled and referred to as Table EV1, Dataset EV1, etc. Legends have to be provided in a separate tab in case of .xls files. Alternatively, the legend can be supplied as a separate text file (README) and zipped together with the Table/Dataset file.

7) Before submitting your revision, primary datasets (and computer code, where appropriate) produced in this study need to be deposited in an appropriate public database (see http://msb.embopress.org/authorguide#dataavailability).

Specifically, we would kindly ask you to provide public access to the mass spectrometry datasets.

Please remember to provide a reviewer password if the datasets are not yet public.

The accession numbers and database should be listed in a formal "Data Availability " section (placed after Materials & Method) that follows the model below (see also http://msb.embopress.org/authorguide#dataavailability). Please note that the Data Availability Section is restricted to new primary data that are part of this study.

Data availability

The datasets (and computer code) produced in this study are available in the following databases:

- RNA-Seq data: Gene Expression Omnibus GSE46843 (https://www.ncbi.nlm.nih.gov/geo/query/acc.cgi?acc=GSE46843)
- [data type]: [name of the resource] [accession number/identifier/doi] ([URL or identifiers.org/DATABASE:ACCESSION])

*** Note - All links should resolve to a page where the data can be accessed. ***

8) The journal requires a statement specifying whether or not authors have competing interests (defined as all potential or actual interests that could be perceived to influence the presentation or interpretation of an article). In case of competing interests, this must be specified in your disclosure statement. Further information: https://www.embopress.org/competing-interests

9) Figure legends and data quantification:
The following points must be specified in each figure legend:

- the name of the statistical test used to generate error bars and P values,
- the number (n) of independent experiments (please specify technical or biological replicates) underlying each data point,
- the nature of the bars and error bars (s.d., s.e.m.)
- If the data are obtained from n {less than or equal to} 2, use scatter blots showing the individual data points.

Discussion of statistical methodology can be reported in the materials and methods section, but figure legends should contain a basic description of n, P and the test applied.

See also the guidelines for figure legend preparation:
https://www.embopress.org/page/journal/14693178/authorguide#figureformat

- Please also include scale bars in all microscopy images.

10) We would also encourage you to include the source data for figure panels that show essential data. Numerical data should be provided as individual .xls or .csv files (including a tab describing the data). For blots or microscopy, uncropped images should be submitted (using a zip archive if multiple images need to be supplied for one panel). Additional information on source data and instruction on how to label the files are available .

11) Our journal encourages inclusion of *data citations in the reference list* to directly cite datasets that were re-used and obtained from public databases. Data citations in the article text are distinct from normal bibliographical citations and should directly link to the database records from which the data can be accessed. In the main text, data citations are formatted as follows: "Data ref: Smith et al, 2001" or "Data ref: NCBI Sequence Read Archive PRJNA342805, 2017". In the Reference list, data citations must be labeled with "[DATASET]". A data reference must provide the database name, accession number/identifiers and a resolvable link to the landing page from which the data can be accessed at the end of the reference. Further instructions are available at .

12) As part of the EMBO publication's Transparent Editorial Process, EMBO reports publishes online a Review Process File to accompany accepted manuscripts. This File will be published in conjunction with your paper and will include the referee reports, your point-by-point response and all pertinent correspondence relating to the manuscript.

You are able to opt out of this by letting the editorial office know (emboreports@embo.org). If you do opt out, the Review Process File link will point to the following statement: "No Review Process File is available with this article, as the authors have chosen not to make the review process public in this case."

We would also welcome the submission of cover suggestions, or motifs to be used by our Graphics Illustrator in designing a cover.

I look forward to seeing a revised form of your manuscript when it is ready and please let me know if you have questions or comments regarding the revision.

You can use this link to submit your revision: https://embor.msubmit.net/cgi-bin/main.plex

Kind regards,

Martina

Martina Rembold, PhD
Senior Editor
EMBO reports

****

Referee #1:

Expansion of the hexanucleotide repeat GGGGCC in C9orf72 is the most common genetic defect causing ALS. Both gain-of-function and reduced levels of C9orf72 protein have been implicated as pathomechanisms. In this manuscript, Jülg and Behrends uncover a mechanism that normally controls the levels of C9orf72. The authors follow up on reported observations by others indicating that C9orf72 and SMCR8 bind to each other and work together in regulating autophagy, and that they are mutually dependent for expression. The authors now go on to show that the absence of SMCR8 leads to degradation of C9orf72 in a way that depends on the E1 ubiquitin-activating enzyme and the proteasome, and that, accordingly, loss of SMCR8 leads to C9orf72 ubiquitination. The authors next utilize mass spectrometry to identify proteins associated with C9orf72 in the absence of SMCR8 and identify, among other hits, the E3 ligase Ubr5. Finally, they show that the interaction of C9orf72 and Ubr5 is stimulated in the absence of SMCR8, and that depletion of Ubr5 reduces K11/48 ubiquitination of C9orf72 and increases its abundance. Together, the data uncover a critical mechanism regulating C9orf72 levels, whereby excess protein that is not assembled in the SMCR8 complex is recycled via Ubr5-mediated ubiquitination and degradation.

The data is of very good quality and the conclusions are convincing. Although many important questions are left unanswered - e.g., whether Ubr5 depletion also rescues expression of mutant C9orf72, and what are the functional consequences of Ubr5 depletion on signaling by the C9orf72/SMCR8 complex- the findings are timely and of broad enough interest to merit publication. Below I suggest only a few simple experiments that would make the story more complete.

1. The authors have also followed up on the observation that SMCR8 levels are reciprocally reduced in the absence of C9orf72. Surprisingly, SMCR8 levels could not be increased by inhibiting the proteasome. It would be good to know: (a) Is SMCR8 degraded in an autophagy-dependent manner? (b) What is the effect of C9orf72 knockout on SMCR8 mRNA levels and vice-versa? (c) Does SMCR8 co-IP with Ubr5 in C9orf72 knockout cells?

2. On p.8, the statement "The increased C9orf72 binding of quality control components in the absence of SMCR8 suggests that uncomplexed C9orf72 exposes an eat-me signal that is likely buried in the fully assembled SMCR8-C9orf72 complex" opens a can of worms. First, how do the authors define "eat-me signal"? This is term is often utilized for autophagy; do they mean a degron? And do they refer to the E3 binding site or the ubiquitination site on C9orf72 that is buried by SMCR8? If the authors

wish to keep the statement, I would like to know the effect of mutations of the predicted (buried) Lys ubiquitination sites.

Other points:

3. Fig.2B is mislabeled: it should be MLN instead of Btz

4. Fig.2D is mislabeled: lane #8 should indicate USP2 treatment

Referee #2:

This manuscript addresses the potential mechanism of the turnover of subunits of the C9orf72- SMCR8 complex involved in several human diseases. The authors claimed to have identified C9orf72 as a substrate of branched ubiquitin chain-dependent protein quality control by the E3 ligase UBR5 and the BAG6 chaperone. While the hypothesis is of potential interest to the relevant field, the conclusions are not well supported by the presented data, which suffer from poor quality, absence of controls, and artificial experimental settings. Solid experiments are needed to support the main conclusions that the degradation of C9orf72 depends on the K11/K48 ubiquitination mediated by UBR5/BAG6.

Major scientific concerns:

1. Data quality is very weak. Most of the data rely on artificial systems of overexpressed proteins. Protein-protein interactions are claimed based on co-immunoprecipitation experiments without proper controls. There are many cases of inconsistent or self-conflicting data. To address these issues, most of the immunoblots need to be quantified. Appropriate controls must be added, and proteins of endogenous levels need to be analyzed.

2. The central claims that C9orf72 is degraded by the UPS system and that URB5-dependnet K11/K48 ubiquitin chains mediate the degradation of C9orf72 are insufficiently supported. Given the poor quality of the data, it is unclear whether the observed changes are the results of a minor/indirect effect or simply artifacts. Direct ubiquitination assays are essential for supporting the conclusions, such as the employment of in vitro ubiquitination assays and in vivo assays using Ub lysine mutant (K11, K48, and K63) and C9orf72 lysine mutants (the three ubiquitin modification sites).

Specific points:

3. Fig 1C, the unspecific bands marked by the asterisk have highly variable intensities that correlate well with the detected C9orf72 levels. The data is contradictory with its interpretations.

4. Fig 1D, the recovery of C9orf72 upon proteasome inhibition was very weak, <10% of the parental level at most. The positive control NRF2 was recovered to its 100% parental level under the same treatment. This result indicates that the vast majority of C9orf72 is not degraded by the proteasome. The authors need to quantify the result and accurately describe it in the manuscript.

5. Fig EV1A, the blot shows that blocking the lysosomal pathway with BafA1 did not increase C9orf72 in SMCR8 KO cells. However, a limited literature search reveals previous studies suggesting opposite results. Both Ugolino et al. 2016 (PMID 278755531) and Leskelä et al., 2019 (PMID 31658762) reported that C9orf72 undergoes autophagosomal degradation in BafA1 treatment experiments. In the study by Chitiprolu et al., 2018 (PMID 30022074), C9ORF72 forms a complex with p62, a receptor for autophagy degradation, suggesting C9ORF72 could be an autophagy substrate. The authors of the current manuscript need to quantify the data carefully and discuss the discrepancies with previous reports.

6. Fig EV1B, it is surprising that inhibition of proteasomal degradation in C9orf72 KO cells did not change SMCR8 levels at all. Although this is not a major point of the manuscript, it may still require some work to validate this result. Does SMCR8 respond to BafA1 treatment?

7. Fig 1D and 2A, the SMCR8 protein level was significantly decreased in parental cells treated with Bzt or MLN7243, while the C9orf72 protein level did not show any change. The author should explain these results.

8. Fig 1F and 1G, the CHX chase results must be quantified. The data seem to be problematic because NRF2 does not show much turnover under the DMSO condition and its half-life seems be even longer with DMSO than with Btz.

9. Fig 2A and 2B have the same issues as Fig 1.

10. Fig 2C, the expression level of HA-C9orf72 appears to be orders of magnitude higher than that of endogenous C9orf72. Studies of the degradation of such overexpressed proteins are unlikely to reflect the natural turnover process of the native protein. In addition, the overexpression of C9orf72 did not cause any restoration of SMCR8 protein levels, contradicting the author's claim that C9orf72 and SMCR8 are mutually dependent.

11. Fig 2D, the apparent ubiquitination of overexpressed HA-C9orf72 in the HA-IP blot is not surprising, as it can be expected for most overexpressed proteins. Negative controls, such as HA-SMCR8 or K-null mutant of C9orf72 should be included to show the specificity of C9orf72.

12. Along the same line, the MS and following analysis are based on the heavily over-expressed HA-C9orf72 that does not mimic the behavior of endogenous C9orf72. The results need to be validated with the endogenous C9orf72. Importantly, the condition of the MS analysis does not exclude the possibility that the detected E3 ligases and ubiquitin-associated proteins are simply binding to ubiquitin or other unknown proteins but have no interaction with C9orf72. More rigorous analysis such as in vitro ubiquitination assays are necessary to support the authors' claims.

13. The claim that "these interactions were increased in the absence of SMCR8 when comparing immunoprecipitations from parental and SMCR8 KO cells" is very weak. There is no quantitation for any of the data. In the top panels, the level of HA-C9orf72 in the HA-IP precipitates from the SMCR KO sample is higher than from the parental sample, so the blots are consistent with the higher level of bait (HA-C9orf72) rather than any increased interaction as claimed. The data are self-conflicting as the bottom panel shows lower level of HA-C9orf72 in the IP samples. Finally, the depletion of SMCR does not show any impact on the level of HA-C9orf72, which completely contradicts with the main conclusion that SMCR8 controls C9orf72 degradation.

14. Fig 3E shows that C9orf72 was pulled down with GFP-URB5 exclusively after proteasomal inhibition. This suggests that URB5 may simply bind to ubiquitin but not C9orf72 at all. Similarly, in Fig 3D, all the putative interactors of HA-C9orf72 was pulled down exclusive after proteasomal inhibition, suggesting that they may simply bind to ubiquitin but not C9orf72.

15. Fig 3F attempts to show a co-IP between GFP-URB5 and endogenous C9orf72. The co-IP experiment lacks an appropriate control for the over-expressed GFP-URB5, and the result could be a non-specific effect.

16. Fig 4A, the experiment to show positive signals of K11/K48 immunoreactivity has two major issues: 1) over-expression of eGFP-C9orf72, 2) the large moiety of GFP could have ubiquitination of its own. The experiment needs to be done with endogenous C9orf72 or HA-C9orf72 expressed at the endogenous level.

17. Fig 4B has the same issues as Fig 4A. In addition, the depletion of UBR4/5 has no impacts on the level of GFP-C9orf72, which contradicts with the main claim that UBR4/5 promotes C9orf72 degradation.

18. Fig EV2A, the detection of K11, K48, and K63 could be due to non-specific effects. Negative controls, such as SMCR8, which authors claim is not ubiquitinated, should be added. The data also raises an important question whether the degradation of C9orf72 is mediated by traditional poly-Ub chains or K11/K48 hybrid chains. There is no good evidence that the latter plays an important role.

19. All the experiments seem to be done in 293T cells. Since it has been reported that the degradation of C9orf72 is cell type-dependent (PMID 31658762), key experiments supporting the conclusions need to be validated in other types of cells such as neurons.

20. The model includes BAG6, but there is hardly any data except for one IP experiment. More evidence is needed. Similarly, the competition between SMCR8 and ubiquitination is mostly speculative and lacks any convincing data.

Referee #3:

The manuscript by Juelg and Behrends is short and sweet. The authors elucidate the mechanism of c9orf72 degradation when it is destabilised in absence of its binding partner, SMCR8. They build a system that establishes degradation via the ubiquitin proteasome system, and which enables interrogation of the degradation machinery by mass-spectrometry. They reveal a host of quality control enzymes and proteins that are further probed for interaction studies (mostly in overexpression settings). The most prominent hits are the BAG6 complex and the E3 ligase UBR5; the latter enables functional verification, as it has been associated with assembly of K11/K48 branched chains. Using the antibody previously developed and verified (Yau et al, Cell 2017), the authors show that c9orf72 is K11/K48 modified, which I agree is highly suggestive that this QC pathway is involved.

The paper adds to the understanding of this complex, which is pathophysiologically relevant in a number of neurodegenerative conditions including ALS. The next steps, ie understanding how repeat expansion variants affect complex formation and degradation behaviour, seem the logical progression from this work.

The experiments are logically presented, the data is clear and the text is crisp. I only have minor comments and would support publication of the manuscript.

Comments:
- In Figure 1, a band is marked on the C9orf72 western blots as non-specific band, however its behaviour sometimes closely matches that of C9orf72 (1C, 1G) but sometimes it does not (1D, 1F, 2A). Please identify this band.

- Please further annotate/explain the NRF2 control blots which also show 2 bands.
- Please explain source of the K11/K48 antibody in methods.
- Please add reference to the pdb code in Fig 2E legend

In the discussion (p9 bottom) the authors further discuss the idea that when in isolation, c9orf72 displays Lys residues not available when in complex with SMCR8, and I agree that the complex is stable as the Lys residues are not available. However, this does not address the more pertinent question, which is how the protein is recognised when in isolation. The authors refer to this elusive 'eat-me' signal in the main text, but maybe there is an opportunity to discuss this in more detail in the Discussion too.

We would like to thank all reviewers for their time, effort and their valuable comments. We greatly appreciate the reviewers' feedback and are confident that the changes we made improved our paper substantially.

##

Referee #1

The data is of very good quality and the conclusions are convincing. Although many important questions are left unanswered -e.g., whether Ubr5 depletion also rescues expression of mutant C9orf72, and what are the functional consequences of Ubr5 depletion on signaling by the C9orf72/SMCR8 complex- the findings are timely and of broad enough interest to merit publication. Below I suggest only a few simple experiments that would make the story more complete.

1. The authors have also followed up on the observation that SMCR8 levels are reciprocally reduced in the absence of C9orf72. Surprisingly, SMCR8 levels could not be increased by inhibiting the proteasome. It would be good to know:

(a) Is SMCR8 degraded in an autophagy-dependent manner?

>This seems not be the case. We treated parental and C9orf72 KO cells with BafA and observed that SMCR8 was not restored under these conditions. This finding is shown in Fig EV1D.

(b) What is the effect of C9orf72 knockout on SMCR8 mRNA levels and vice-versa?

>We thank the reviewer for this question and agree that it is important to consider the regulation of SMCR8 and C9orf72 at the transcriptional level. Ugolino *et al.* 2016 have already addressed this issue and studied SMCR8 and C9orf72 mRNA levels in respective knockout mice and 293T cells using qPCR. In these settings, SMCR8 mRNA levels were not reduced upon depletion of C9orf72. In contrast, C9orf72 transcripts were even increased in SMCR8 KO cells. The findings of Ugolino *et al*. indicate that mutual regulation of SMCR8 and C9orf72 levels might occur at the protein level.

(c) Does SMCR8 co-IP with Ubr5 in C9orf72 knockout cells?

>We addressed this question in our Figure for the Reviewers represented below where we overexpressed GFP-SMCR8 and GFP-C9orf72 (or GFP alone) in the respective KO cell line followed by IPs. Surprisingly, we found that uncomplexed SMCR8 indeed

interacts with both UBR5 and BAG6. Compared to uncomplexed C9orf72, SMCR8's interaction with BAG6 seemed even more prominent. This increased interaction of BAG6 with SMCR8 could imply that BAG6 chaperones uncomplexed SMCR8 for a longer time to allow SMCR8 to exert functions outside of the SMCR8-C9orf72-WDR41 complex such as e.g. the regulation of ULK1 kinase activity and gene expression (Jung et al. 2017 PMID: 28195531). Since this exciting hypothesis requires rigorous testing and in-depth mechanistic analysis, we would like to report on this in a separated study. At this point, we feel that this finding is too preliminary for the current manuscript. If, however, the reviewer sees this differently, we would be happy to included it as e.g. a supplementary figure.

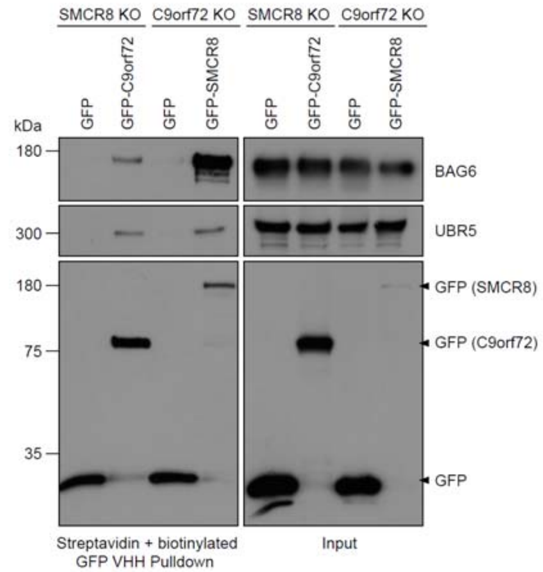

Figure for the reviewers – Interaction of BAG6 and UBR5 with uncomplexed C9orf72 and SMCR8.

SMCR8 KO and C9orf72 KO cells were transiently transfected with GFP-C9orf72 and GFP-SMCR8, respectively. Cells were treated with Btz and lysates were subjected to streptavidin agarose coupled with biotinylated GFP VHH nanobodies. Transfection of GFP served as control.

2. On p.8, the statement "The increased C9orf72 binding of quality control components in the absence of SMCR8 suggests that uncomplexed C9orf72 exposes an eat-me signal that is likely buried in the fully assembled SMCR8-C9orf72 complex" opens a can of worms. First, how do the authors define "eat-me signal"? This is term is often utilized for autophagy; do they mean a degron? And do they refer to the E3 binding site or the ubiquitination site on C9orf72 that is buried by SMCR8? If the authors wish to keep the statement, I would like to know the effect of mutations of the predicted (buried) Lys ubiquitination sites.

>We are grateful for this critical comment and apologize for being too vague in our statement. When writing "eat-me signal" we actually meant "degron" and referred to the ubiquitination sites in C9orf72 which are buried in the complex with SMCR8. As suggested, we generated a C9orf72 variant in which all four candidate ubiquitination sites were mutated to arginine and performed IPs under mild and denaturing conditions. Intriguingly, this mutant bound more BAG6 and UBR5 and carried more ubiquitin than wild-type C9orf72. The latter trend become even more prominent when we additionally mutated three adjacent lysines in the C9orf72 KR7 variant. These findings are now included as new Fig 4B-D. Nevertheless, since the nature of the degron is currently not clear, we dropped our degron/eat-me signal statement. Nevertheless, in our discussion we highlighted that this is an important question to address (page 10 top).

Other points:

3. Fig.2B is mislabeled: it should be MLN instead of Btz

>We thank the reviewer for pointing out that we mislabeled the MLN7243 treatment. We corrected this mistake in our fluorescence data which is now represented in Fig 2C in the revised manuscript.

4. Fig.2D is mislabeled: lane #8 should indicate USP2 treatment

>We thank the reviewer for noticing that the USP2 treatment in Fig 2D, which is now Fig 2F, was not labeled. Experiments in Fig 2F and Fig 5B were performed under denaturing conditions which are not suitable for USP2 reactions. Therefore, we performed USP2 treatments directly on the beads after binding. In Fig 4A, the HA-IP was performed using mild conditions. Here, we also treated with USP2 directly on the beads and in addition, added USP2 to the input to visualize the effect of USP2 on ubiquitin.

##

Referee #2

This manuscript addresses the potential mechanism of the turnover of subunits of the C9orf72- SMCR8 complex involved in several human diseases. The authors claimed to have identified C9orf72 as a substrate of branched ubiquitin chain-dependent protein quality control by the E3 ligase UBR5 and the BAG6 chaperone. While the hypothesis is of potential

interest to the relevant field, the conclusions are not well supported by the presented data, which suffer from poor quality, absence of controls, and artificial experimental settings. Solid experiments are needed to support the main conclusions that the degradation of C9orf72 depends on the K11/K48 ubiquitination mediated by UBR5/BAG6.

Major scientific concerns:

1. Data quality is very weak. Most of the data rely on artificial systems of overexpressed proteins. Protein-protein interactions are claimed based on co-immunoprecipitation experiments without proper controls. There are many cases of inconsistent or self-conflicting data. To address these issues, most of the immunoblots need to be quantified. Appropriate controls must be added, and proteins of endogenous levels need to be analyzed.

>We agree that the robustness of our findings could be made more prominent by quantifying key results. We have done this now for Fig 1D and Fig 2A as these represent analyses of endogenous C9orf72. The quantifications clearly support our claim that C9orf72 abundance is sensitive to inhibition of the ubiquitin-proteasome system in the absence of SMCR8. Moreover, we added additional controls where this clearly helps to substantiate our findings (e.g., GFP in IPs under mild and denaturing conditions).

2. The central claims that C9orf72 is degraded by the UPS system and that URB5-dependnet K11/K48 ubiquitin chains mediate the degradation of C9orf72 are insufficiently supported. Given the poor quality of the data, it is unclear whether the observed changes are the results of a minor/indirect effect or simply artifacts. Direct ubiquitination assays are essential for supporting the conclusions, such as the employment of in vitro ubiquitination assays and in vivo assays using Ub lysine mutant (K11, K48, and K63) and C9orf72 lysine mutants (the three ubiquitin modification sites).

>We are very thankful for the suggestion of using C9orf72 K-R mutants in our study. We have generated a variant in which all four candidate ubiquitination sites have been mutated to arginine (C9orf72 KR4) and used it in IPs under mild and denaturing conditions (please see #11 for details). In addition, we used UBR5 overexpression as an additional system and observed increase ubiquitination of C9orf72 (please see #11 for details). However, given the size of UBR5 and the lack of literature on UBR5 purification, we did not attempt to perform an in vitro ubiquitination assay with UBR5 and C9orf72. The specificity of such an in vitro reaction (and hence its usefulness) is

also at least debatable. Similarly, the use of ubiquitin lysine mutants is highly controversial in the field. To circumvent these issues, we deliberately took advantage of the highly specific hybrid chain antibody to detect K11/K48 polyubiquitin.

Specific points:

3. Fig 1C, the unspecific bands marked by the asterisk have highly variable intensities that correlate well with the detected C9orf72 levels. The data is contradictory with its interpretations.

>We perceive the concern of the referee about the asterisk marked unspecific band in our C9orf72 Western blots and refer to the manufacturer's information of GeneTex (https://www.genetex.com/Product/Detail/C9orf72-antibody-GT779/GTX632041#datasheet) where this band is also detected in other tissues types such as mouse and rat brain extracts when immunoblotting with antibody GTX632041. Here, the unspecific band varies in intensities for mouse and rat even though C9orf72 levels are the same in both tissue extracts. We also refer to the C9orf72 antibody characterization study by Laflamme et al. 2019 (PMID: 31612854, Supplement Fig 1A and 1B) showing this unspecific protein band in HEK293 cells and mouse brain lysates when immunoblotting with GTX632041 for C9orf72. Here, the unspecific band remains constant independent from C9orf72 levels. In addition, Lui et al. 2021 (PMID: 33558503, Source Data Fig 5F) used the GTX632041 antibody for Western blot quantification also detecting a protein band slightly above the C9orf72 signal which appears to be inconsistent throughout different replicates of the same experiment setup. When comparing C9orf72 levels in parental 293T (Fig 1A, 1B, 1C, 1D, 1G, 2A, EV1A, EV1B, EV1C and EV1E) we also observe that the unspecific band seem to appear in a randomized manner without showing any direct correlation to the presence of C9orf72. Based on the above-mentioned arguments, we consider the asterisk protein band as non-specific.

4. Fig 1D, the recovery of C9orf72 upon proteasome inhibition was very weak, <10% of the parental level at most. The positive control NRF2 was recovered to its 100% parental level under the same treatment. This result indicates that the vast majority of C9orf72 is not degraded by the proteasome. The authors need to quantify the result and accurately describe it in the manuscript.

>The reviewer's observation that C9orf72 levels are not restored to the full extend upon proteasomal inhibition in Fig 1D is also stated in our manuscript by referring to "partially recovered" C9orf72 levels. In addition, we now quantified C9orf72 protein levels represented in the new Fig 1E showing that treatment with Btz can recover C9orf72 up to ~ 40%.

5. Fig EV1A, the blot shows that blocking the lysosomal pathway with BafA1 did not increase C9orf72 in SMCR8 KO cells. However, a limited literature search reveals previous studies suggesting opposite results. Both Ugolino et al. 2016 (PMID 278755531) and Leskelä et al., 2019 (PMID 31658762) reported that C9orf72 undergoes autophagosomal degradation in BafA1 treatment experiments. In the study by Chitiprolu et al., 2018 (PMID 30022074), C9ORF72 forms a complex with p62, a receptor for autophagy degradation, suggesting C9ORF72 could be an autophagy substrate. The authors of the current manuscript need to quantify the data carefully and discuss the discrepancies with previous reports.

>We thank the referee for bringing this up. We want to point out that both Ugolino et al. 2016 and Leskelä et al. 2019 addressed C9orf72 degradation in presence of SMCR8. Although not specifically mentioned, Chitiprolu et al. 2018 also studied C9orf72 when SMCR8 was present in the cell. It is reasonable to assume and we do not exclude that C9orf72 as a member of the functional C9orf72-SMCR8 complex undergoes autophagic degradation as part of a feedback loop as shown for other key regulator such as ULK1.
In our manuscript, however, we monitored uncomplexed C9orf72 which, without SMCR8, most likely loses its functional relevance in the cell and therefore requires rapid turnover by the proteasome. In addition, Ugolino et al. 2016 (Fig S7E) showed that inhibition of the lysosomal pathway but also the inhibition of the proteasome causes increased levels of overexpressed C9orf72 with MG132 having an even stronger effect than BafA1. Therefore, we do not find our results in discrepancy with the literature in the field. Instead, the complex mechanism of C9orf72 degradation is intriguing and seems tightly controlled. Nevertheless, we added a sentence regarding other degradation pathways for complexed C9orf72 in our discussion.

6. Fig EV1B, it is surprising that inhibition of proteasomal degradation in C9orf72 KO cells did not change SMCR8 levels at all. Although this is not a major point of the manuscript, it may still require some work to validate this result. Does SMCR8 respond to BafA1 treatment?

>We agree that the implied differential regulation of SMCR8 is an interesting aspect. It

also emphasizes that the quality control of C9orf72-SMCR8 is a very complex mechanism that is in need of extensive investigation. To further contribute to the understanding of C9orf72-SMCR8 complex regulation, we monitored SMCR8 protein abundance upon C9orf72 KO in the absence and presence of BafA1 which is shown as new Fig EV1D. Here, we could not detect restoration of SMCR8 protein levels upon inhibition of autophagosomal degradation.

7. Fig 1D and 2A, the SMCR8 protein level was significantly decreased in parental cells treated with Bzt or MLN7243, while the C9orf72 protein level did not show any change. The author should explain these results.

>We thank the referee for noticing the decrease of SMCR8 levels after proteasome inhibition. Since the quality control of SMCR8 remains largely elusive, we can only speculate on how SMCR8 is degraded in the cell. In the presence of C9orf72, SMCR8 might undergo slow proteasomal degradation while blocking this pathway could redirect SMCR8 to faster turnover via the autophagic pathway leading to a net decrease in protein levels. This would be in line with our observation that blocking the proteasome does not restore SMCR8 protein levels in C9orf72 KO cells as shown in Fig. EV1C.

8. Fig 1F and 1G, the CHX chase results must be quantified. The data seem to be problematic because NRF2 does not show much turnover under the DMSO condition and its half-life seems be even longer with DMSO than with Btz.

>We apologize for the confusion – the lower band in the NRF2 blot is an unspecific band detected by the NRF2 antibody. We indicated the height of the specific NRF2 band with an arrow on all relevant immunoblots (revised Fig 1D and 1G, new Fig 2E, revised Fig 3C and 3D). Considering the right protein band, NRF2 is rapidly turned over in untreated conditions whereas proteasomal inhibition leads to a strong stabilization of NRF2 levels (revised Fig 1G), confirming the effect of Btz treatment at steady-state levels (revised Fig 1D). Therefore, we are confident that our CHX experiments has worked and shows a black-and-white effect of Btz on C9orf72 which we do not find necessary to quantify.

9. Fig 2A and 2B have the same issues as Fig 1.

>As stated above (in response to #4), we did not state in our manuscript that C9orf72 levels were fully recovered. Nevertheless, we quantified C9orf72 protein levels represented in Fig 2A showing that treatment with MLN7243 can recover C9orf72 more than 20%. This quantification is now shown as new Fig 2B.

10. Fig 2C, the expression level of HA-C9orf72 appears to be orders of magnitude higher than that of endogenous C9orf72. Studies of the degradation of such overexpressed proteins are unlikely to reflect the natural turnover process of the native protein. In addition, the overexpression of C9orf72 did not cause any restoration of SMCR8 protein levels, contradicting the author's claim that C9orf72 and SMCR8 are mutually dependent.

>The strong decrease of endogenous C9orf72 when SMCR8 is absence makes it challengeable to examine the interactome of endogenous C9orf72 in this setting. Therefore, we took advantage of an overexpression system. Treatment with Btz caused increased levels of exogenous C9orf72, implying that the protein is at least partially following a similar ubiquitin-proteasome system-dependent path. These findings are now included in the revised manuscript as new Fig 2E. Importantly, we did not expect to rescue SMCR8 protein levels when overexpressing C9orf72 as we used a CRISPR/Cas engineered cell line lacking SMCR8. Hence, this setting makes it impossible to restore SMCR8 protein levels.

11. Fig 2D, the apparent ubiquitination of overexpressed HA-C9orf72 in the HA-IP blot is not surprising, as it can be expected for most overexpressed proteins. Negative controls, such as HA-SMCR8 or K-null mutant of C9orf72 should be included to show the specificity of C9orf72.

>In the experiment shown in Fig 2D we ensured specificity by treating cell with Btz and by incubating HA-immune complexes with the promiscuous deubiquitinase USP2. As additional control, we performed denaturing IPs of GFP and GFP-C9orf72 in SMCR8 KO cells and did not observe any ubiquitination of GFP. This finding is now shown as new Fig. EV3. As suggested, we also generated a C9orf72 variant in which all four candidate ubiquitination sites were mutated to arginine (C9orf72 KR4). Denaturing IPs revealed that this mutant is even more ubiquitinated than wild-type C9orf72 (new Fig 4C). Since it is known in the field that ubiquitin can "jump" to other sites in K-to-R mutants, we mutated three adjacent lysines to arginine (C9orf72 KR7). However, this mutant likewise showed increased ubiquitination compared to wild-type (new Fig 4D). These findings are consistent with the observation that C9orf72 KR4 also bound more

BAG6 and UBR5 (new Fig 4B). As additional control, we again employed denaturing IPs to monitor the ubiquitination of C9orf72-HA in SMCR8 KO cells expressing GFP or GFP-UBR5 and observed increase ubiquitination in the latter condition (new Fig 5A).

12. Along the same line, the MS and following analysis are based on the heavily over-expressed HA-C9orf72 that does not mimic the behavior of endogenous C9orf72. The results need to be validated with the endogenous C9orf72. Importantly, the condition of the MS analysis does not exclude the possibility that the detected E3 ligases and ubiquitin-associated proteins are simply binding to ubiquitin or other unknown proteins but have no interaction with C9orf72. More rigorous analysis such as in vitro ubiquitination assays are necessary to support the authors' claims.

>First of all, a number of studies supports the view that overexpressed C9orf72 does indeed mimic the behavior of endogenous C9orf72 for example with regard to its interaction landscape (PMID: 27103069: Sellier et al. 2016) and with regard to its turnover (new Fig 1G and 2E) Second, we would like to point out that we actually validated the UBR5 interaction with endogenous C9orf72. While we formally did not exclude the possibility that a priming ubiquitin on C9orf72 is recruiting the BAG6-UBR5 machinery, we tested this scenario by differentially treating C9orf72 bound to HA agarose with the promiscuous deubiquitinase USP2 prior to washing, elution and immunoblot analysis. This experiment clearly shows that BAG6 and UBR5 bind C9orf72 independent of its ubiquitination status (new Fig 4A).

13. The claim that "these interactions were increased in the absence of SMCR8 when comparing immunoprecipitations from parental and SMCR8 KO cells" is very weak. There is no quantitation for any of the data. In the top panels, the level of HA-C9orf72 in the HA-IP precipitates from the SMCR KO sample is higher than from the parental sample, so the blots are consistent with the higher level of bait (HA-C9orf72) rather than any increased interaction as claimed. The data are self-conflicting as the bottom panel shows lower level of HA-C9orf72 in the IP samples. Finally, the depletion of SMCR does not show any impact on the level of HA-C9orf72, which completely contradicts with the main conclusion that SMCR8 controls C9orf72 degradation.

>The point of this experiment (revised Fig 3D) was to validate the MS result and confirm the binding of uncomplexed C9orf72 to a number of quality control machinery components in an independent setting. In this context, we do not feel that a quantification is required. Notably, while the inputs for C9orf72 in this IP (revised Fig

3D) are quite similar between SMCR8 KO and parental cells, ubiquitination of C9orf72 upon Btz treatment in SMCR8 KO cells blurs the C9orf72 band and leaves the impression of uneven C9orf72 levels. However, the levels of all tested interactors (except UBL4A) increase non-proportionally to the apparent uneven levels of C9orf72 in parental and SMCR8 KO cells. To increase consistency, we exchanged the lower part with a more representative experiment (revised Fig 3D). In addition, we toned down our conclusion that binding of C9orf72 to its partners was increased in the absence of SMCR8 (page 7, bottom). While carefully re-evaluation of overexpressed C9orf72 in parental and SMCR8 KO cells by CHX chase assays revealed a clear proteasome-dependent turnover as shown in the new Fig 2E, we suspect that the steady state C9orf72 levels in Fig 3D might not be the optimal setting to observe the turnover differences between parental and KO cells.

14. Fig 3E shows that C9orf72 was pulled down with GFP-URB5 exclusively after proteasomal inhibition. This suggests that URB5 may simply bind to ubiquitin but not C9orf72 at all. Similarly, in Fig 3D, all the putative interactors of HA-C9orf72 was pulled down exclusive after proteasomal inhibition, suggesting that they may simply bind to ubiquitin but not C9orf72.

>We indeed observed that C9orf72 interacts with UBR5 also in DMSO control (revised Fig 3E). While we do not formally exclude the possibility that C9orf72 carrying a priming ubiquitin recruits UBR5, we tested the ubiquitin dependence of the UBR5-C9orf72 interaction by performing IPs in which C9orf72 immune complexes bound to HA-agarose were differentially treated with USP2. In this setting, we did not observe any binding differences (new Fig 4A), indicating that UBR5 binds C9orf72 independently of its ubiquitination.

15. Fig 3F attempts to show a co-IP between GFP-URB5 and endogenous C9orf72. The co-IP experiment lacks an appropriate control for the over-expressed GFP-URB5, and the result could be a non-specific effect.

>Since we showed that GFP-UBR5 and not GFP alone specifically immunoprecipitates overexpressed C9orf72 (revised Fig 3E), we did not feel that Fig 3F needs another control beside MOCK transfection.

16. Fig 4A, the experiment to show positive signals of K11/K48 immunoreactivity has two major issues: 1) over-expression of eGFP-C9orf72, 2) the large moiety of GFP could have

ubiquitination of its own. The experiment needs to be done with endogenous C9orf72 or HA-C9orf72 expressed at the endogenous level.

>We thank the referee for pointing out that GFP might potentially be ubiquitinated itself. Accordingly, we performed denaturing IPs with GFP-C9orf72 and GFP only as control. In this setting, we did not observe any ubiquitination of GFP (new Fig EV3).

17. Fig 4B has the same issues as Fig 4A. In addition, the depletion of UBR4/5 has no impacts on the level of GFP-C9orf72, which contradicts with the main claim that UBR4/5 promotes C9orf72 degradation.

>In this experiment, we address GFP-C9orf72 ubiquitination but not the overall protein level of GFP-C9orf72. As siCTRL and siUBR4/5 conditions are treated with Btz to visualize ubiquitination in the first place, we did not expect GFP-C9orf72 levels to be reduced after knockdown of UBR4/5 but rather increased since proteasomal turnover is blocked. The specificity of GFP-C9orf72 has been addressed as explained above and shown in new Fig EV3.

18. Fig EV2A, the detection of K11, K48, and K63 could be due to non-specific effects. Negative controls, such as SMCR8, which authors claim is not ubiquitinated, should be added. The data also raises an important question whether the degradation of C9orf72 is mediated by traditional poly-Ub chains or K11/K48 hybrid chains. There is no good evidence that the latter plays an important role.

>The re-ordered EV2B (previous Fig EV2A) supports our MS data in which we find K11, K48 and K63 as signature peptides for C9orf72 under denaturing conditions. In this context, this denaturing IP is a validation experiment. Since these antibodies and the denaturing IP condition are broadly used in the field, it is not clear how a Btz-sensitive ubiquitin linkage signal would be due to non-specific effects. Moreover, our manuscript does not state at any point that SMCR8 is not ubiquitinated. Yet, there is no data on how SMCR8 is degraded in the cell and to what extend the protein gets ubiquitinated. Therefore, we do not consider SMCR8 as a negative control. Lastly, there is an increasing literature on the importance of heterotypic ubiquitin chains (e.g., Stolz & Dikic 2018 PMID: 29191367; Baur&Rape 2020 PMID: 31951536).

19. All the experiments seem to be done in 293T cells. Since it has been reported that the

degradation of C9orf72 is cell type-dependent (PMID 31658762), key experiments supporting the conclusions need to be validated in other types of cells such as neurons.

>We agree that it is of great interest to study the UBR5-mediated degradation of C9orf72 in other cell types. Therefore, we monitored C9orf72 abundance in response to Btz and MLN7243 in parental and SMCR8 KO HAP1 cells. These findings are now shown as new Fig EV1A and EV2A.

20. The model includes BAG6, but there is hardly any data except for one IP experiment. More evidence is needed. Similarly, the competition between SMCR8 and ubiquitination is mostly speculative and lacks any convincing data.

>We would like to point out that BAG6 was found by IP-MS (Fig 3B) and was validated as C9orf72 interactor in Fig 3D. Our model (now Fig 6) did not imply any competition between ubiquitination and SMCR8. Rather the model shows that C9orf72 is ubiquitinated when SMCR8 is not presence. In our Discussion (page 10, top) we have speculated that the presence of SMCR8 might prevent quality control units to bind and ubiquitinate C9orf72. We feel that the Discussion is the right place for such a hypothesis. However, we rephrased the respective sentence to make its speculative nature more apparent.

##

Referee #3

The paper adds to the understanding of this complex, which is pathophysiologically relevant in a number of neurodegenerative conditions including ALS. The next steps, ie understanding how repeat expansion variants affect complex formation and degradation behaviour, seem the logical progression from this work. The experiments are logically presented, the data is clear and the text is crisp. I only have minor comments and would support publication of the manuscript. Comments:

- In Figure 1, a band is marked on the C9orf72 western blots as non-specific band, however its behaviour sometimes closely matches that of C9orf72 (1C, 1G) but sometimes it does not (1D, 1F, 2A). Please identify this band.

>We perceive the concern of the referee about the asterisk marked unspecific band in our C9orf72 Western blots and refer to the manufacturer's information of GeneTex (https://www.genetex.com/Product/Detail/C9orf72-antibody-GT779/GTX632041#datasheet) where this band is also detected in other tissues types

such as mouse and rat brain extracts when immunoblotting with antibody GTX632041. Here, the unspecific band varies in intensities for mouse and rat even though C9orf72 levels are the same in both tissue extracts. We also refer to the C9orf72 antibody characterization study by Laflamme et al. 2019 (PMID: 31612854, Supplement Fig 1A and 1B) showing this unspecific protein band in HEK293 cells and mouse brain lysates when immunoblotting with GTX632041 for C9orf72. Here, the unspecific band remains constant independent from C9orf72 levels. In addition, Lui et al. 2021 (PMID: 33558503, Source Data Fig 5F) used the GTX632041 antibody for Western blot quantification also detecting a protein band slightly above the C9orf72 signal which appears to be inconsistent throughout different replicates of the same experiment setup. When comparing C9orf72 levels in parental 293T (Fig 1A, 1B, 1C, 1D, 1G, 2A, EV1A, EV1B, EV1C and EV1E) we also observe that the unspecific band seem to appear in a randomized manner without showing any direct correlation to the presence of C9orf72. Based on the above-mentioned arguments, we consider the asterisk protein band as non-specific.

- Please further annotate/explain the NRF2 control blots which also show 2 bands.

>We apologize for the confusion – the lower band in the NRF2 blot is an unspecific band detected by the NRF2 antibody. We indicated the height of the specific NRF2 band with an arrow on all relevant immunoblots (revised Fig 1D and 1G, new Fig 2E, revised Fig 3C and 3D). Considering the right protein band, NRF2 is rapidly turned over in untreated conditions whereas proteasomal inhibition leads to a strong stabilization of NRF2 levels (revised Fig 1G), confirming the effect of Btz treatment at steady-state levels (revised Fig 1D).

- Please explain source of the K11/K48 antibody in methods.

>We have added the source of the K11/K48 antibody (which is Genentech) in the method sections.

- Please add reference to the pdb code in Fig 2E legend

>We inserted the reference Su et al. 2021 along with PDB 6V4U in the figure legend which is now Fig 2G in the revised manuscript.

In the discussion (p9 bottom) the authors further discuss the idea that when in isolation, c9orf72 displays Lys residues not available when in complex with SMCR8, and I agree that the complex is stable as the Lys residues are not available. However, this does not address the more pertinent question, which is how the protein is recognised when in isolation. The authors refer to this elusive 'eat-me' signal in the main text, but maybe there is an opportunity to discuss this in more detail in the Discussion too.

>We apologized for the unprecise usage of the term “eat-me signal”. While we meant to write “degron”, we realized that such a motif is actually different from a ubiquitination site. Since we do not have any data on C9orf72 recognition by BAG6 and/or UBR5, we feel that it is too early to including this aspect in our discussion. Nevertheless, we added that this is an important question to address in the future (page 10 top).

1st Revision - Editorial Decision 3rd May 2023

Dear Christian,

Thank you once again for the submission of your revised manuscript and for your patience while we have reviewed it. As you know, referee 1 and 3 are very positive and support publication in EMBO reports, while referee 2 raised concerns in particular regarding the use of overexpressed proteins. You have supplied a preliminary point-by-point response to these concerns, which I have further discussed with referee 1, who considered your response satisfactory and sufficient to clarify important points. The referee also noted that "[...] several of the critiques that remain unaddressed reflect fundamental difficulties that are common to most if not all studies of ubiquitination". Taking your feedback, the feedback from referee 1 and the positive assessment from referee 1 and 3, both good experts in the field, into account, we have decided to proceed with the publication of your study, given that all conclusions are appropriately phrased and the limitations discussed, as also outlined in your rebuttal. Please also submit all source data for the Western blots, as you already indicated in your response. Regarding source data: We need the data sorted per figure, then the files are zipped and uploaded.

From the editorial side, there are also a few things that we need before we can proceed with the official acceptance of your study.

- Please update the 'Conflict of interest' paragraph to our new 'Disclosure and competing interests statement'. For more information see
https://www.embopress.org/page/journal/14693178/authorguide#conflictsofinterest

- Regarding the Author Contributions, we now use CRediT to specify the contributions of each author in the journal submission system. CRediT replaces the author contribution section, which therefore needs to be removed from the manuscript text. You can use the free text box in our system if you wish to provide more detailed descriptions. See also guide to authors https://www.embopress.org/page/journal/14693178/authorguide#authorshipguidelines.

- Please rename Table 2 to Dataset EV1 and add the name and legend to a new sheet.

- Table 1 should be a word or pdf file. The legend should be in the table file.

- Please describe your new data in the abstract in present tense.

- Please add the heading 'Expanded View Figure Legends' to that section.

- Finally, EMBO reports papers are accompanied online by A) a short (1-2 sentences) summary of the findings and their significance, B) 2-3 bullet points highlighting key results and C) a synopsis image that is 550x300-600 pixels large (width x height) in PNG for JPG format. You can either show a model or key data in the synopsis image. Please note that the size is rather small and that text needs to be readable at the final size. Please send us this information along with the revised manuscript.

We look forward to seeing a final version of your manuscript as soon as possible.

With kind regards,

Martina

Martina Rembold, PhD
Senior Editor
EMBO reports

****

Referee #1:

In the revised manuscript, the authors have dealt adequately with my concerns. Overall, the manuscript is improved relative to the previous version. I only have two minor points remaining:

1. The finding that uncomplexed SMCR8 interacts with both UBR5 and BAG6 needs to be presented as a supplementary figure. The authors may also offer their interpretation of the data while stating that it remains speculative and requires further analysis.

2. There is a typo in the abstract: "C9orf72 binds SMCR8 to from a robust complex
that regulates small GTPases". It should be form instead of from. Considering that figures were mislabeled in the previous version the authors should have been extra careful with the revision.

Referee #2:

The revised manuscript shows reasonable evidence that highly overexpressed C9orf72 binds E3 ligase UBR5 and the BAG6 chaperone and there is an unknown percentage of overexpressed C9orf72 that might have K11/K48 ubiquitin chains.

However, there are important issues insufficiently addressed that left major gaps between the data and the main conclusions.

1. Some new data has been added. But there are still cases of lacking appropriate controls or self-conflicting data or interpretations (see below). Most of the data rely on artificial systems of overexpressed proteins.

2. The central claim that URB5-dependnet K11/K48 ubiquitin chains mediate the degradation of C9orf72 is still insufficiently supported. The main new data using C9orf72 lysine mutants does not support but rather weakens the central claims (see below). The roles of traditional ubiquitin chains in the turnover of C9orf72 is not addressed or discussed.

3. Fig 1C, the asterisk marks an "unspecific" band recognized by the C9orf72 antibody that correlates strongly with the lower band interpreted as C9orf72. The literature has reported such unspecific bands, but this blot shows such a strong level of correlation that is unusual for an unspecify band. Did this "unspecific" band truly appear in a randomized manner as claimed when the experiments are repeated? Please provide an explanation.

Similarly, Fig 5D, there is an upper band that changes in correlation with the lower band interpreted as C9orf72. Does this mean that siUBR4/5 regulates these proteins in a manner that is unspecific to C9orf72?

4. Fig 1D, the blot does not seem to match the claim that "treatment with Btz can recover C9orf72 up to ~40%". A better plot needs to be shown to match the reported quantitation.

5. Response 10

The authors argue that "the strong decrease of endogenous C9orf72 when SMCR8 is absence makes it challengeable to examine the interactome of endogenous C9orf72 in this setting." Contrary to this statement, Fig 1G shows a highly detectable level of C9orf72 in SMCR8 KO cells before CHX treatment, suggesting that many key experiments could be done with the endogenous level of C9orf72 protein.

To avoids the artifacts especially when it pertains to the studies of ubiquitination, it is important to study the endogenous protein or a stably expressed protein near the endogenous level, such as the experiments to show the K11/K48 modification of endogenous C9orf72 and various protein-protein interactions. It remains unknown whether endogenous C9orf72 has K11/K48 chains or interacts with other endogenous proteins identified in the mass spec.

6. Response 11

The authors added new data that does not support the hypothesis but rather weakens the central claims. One of the major concerns is the reliance on the heavily overexpressed HA-C9orf72 (orders of magnitude higher than the endogenous level) to show the purported ubiquitination of this protein. It is well-known the overexpression leads to abnormal protein folding and artificial ubiquitination. The crucial K-to-R mutations did not reduce the ubiquitination but rather increase the ubiquitination (new Fig 4C, 4D), weakening the claim that the Btz-sensitive ubiquitination sites detected by mass spectrometry analysis indicate a ubiquitin-dependent regulation of C9orf72. The most straightforward explanation is that these are all unspecific ubiquitination modifications caused by the overexpression of the protein.

7. Response 12

The authors also argue in a new experiment that it "clearly shows that BAG6 and UBR5 bind C9orf72 independent of its ubiquitination status (new Fig 4A). This data is self-conflicting because the purported binding of BAG6/UBR5 to C9orf72 completely depends on the treatment with Btz, suggesting that ubiquitination is required for the biding. In fact, all the protein-protein interactions in Fig 3D are completely Btz-dependent. In their response, the authors suggest a "possibility that a priming ubiquitin on C9orf72 is recruiting the BAG6-UBR5 machinery". If this is how the authors interpret these conflicting results, they should clearly discuss it in the manuscript.

8. Response 13

Fig 3D, the depletion of SMCR does not change the level of HA-C9orf72, which contradicts with the main conclusion that SMCR8 controls C9orf72 degradation. In their response, the authors suggest that "the steady state C9orf72 levels in Fig 3D might not be the optimal setting to observe the turnover differences between parental and KO cells". But at the same time, the authors were trying to argue that the interactions between C9orf72 and BAG6/UBR5/others were increased in the absence SMCR8 (page 7 bottom, page 10 middle paragraph).

So all these increased protein-protein interactions have no impact on the turnover of C9orf72, contradicting with the main claim. The authors need to make consistent interpretations for these self-conflicting data.

9. Response 15

Previous comments: "Fig 3F attempts to show a co-IP between GFP-URB5 and endogenous C9orf72. The co-IP experiment lacks an appropriate control for the over-expressed GFP-URB5, and the result could be a non-specific effect."

In their response, the authors argue that, since a GFP negative control is shown in Fig. 3E, it is no longer needed in 3F. However, Fig. 3F shows an independent and different experiment (endogenous vs. overexpressed C9orf72). The appropriate negative control should be included here.

More importantly, to avoid the artificial effects of overexpressed proteins, a better co-IP experiment should be performed with both endogenous URB5 and C9orf72 or those expressed near the endogenous levels.

10. Response 17

Fig 5C (previous Fig 4B), previous comment: "the depletion of UBR4/5 has no impacts on the level of GFP-C9orf72, which contradicts with the main claim that UBR4/5 promotes C9orf72 degradation."

The comment is not addressed by the authors. GFP-C9orf72 levels were not increased after knockdown of UBR4/5, although expected by the authors, raising the major issue of lacking consistent evidence supporting the main claim.

11. Response 20

The authors speculate that "The fact that the identified ubiquitin acceptor sites are located within these two interfaces indicates that not only deficiency of the C9orf72-SMCR8 complex likely causes C9orf72 turnover but also that SMCR8 might physically block C9orf72 degradation." However, mutating these sites does not increase but rather decrease C9orf72 (Fig 4), arguing against this model. There is no evidence in the current manuscript to support the idea that the Btz-sensitive ubiquitin sites at the C9orf72-SMCR8 interface plays any role in K11/K48 mediated quality control of C9orf72. The authors need to be careful in their discussion and avoid making unsubstantiated speculations.

In the discussion, the authors claim that "the findings provided here could serve as a strategy to modulate C9orf72 quality control in order to revert C9orf72 loss during on-set or progression of disease." However, based on Fig 1D and 1G, C9orf72 in parental cells is completely insensitive to Btz, suggesting that ubiquitination is not the main factor for its stabilization in the cell. Although the specific information on C9orf72 turnover in the absence SMCR8 is worth learning, its relevance to human diseases where SMCR8 is presumably intact is unclear.

Referee #3:

I was happy with the authors addressing the points I raised and felt they did a reasonable job addressing the other Reviewers' comments. Hapy to support publication.

The authors have addressed all minor editorial requests.

2nd Revision - Editorial Decision 26th May 2023

Prof. Christian Behrends
Ludwig-Maximilians-University Munich
Munich Cluster for Systems Neurology
Feodor-Lynen Strasse 17
Munich 81377
Germany

Dear Christian,

I am very pleased to accept your manuscript for publication in the next available issue of EMBO reports. Thank you for your contribution to our journal.

At the end of this email I include important information about how to proceed. Please ensure that you take the time to read the information and complete and return the necessary forms to allow us to publish your manuscript as quickly as possible.

As part of the EMBO publication's Transparent Editorial Process, EMBO reports publishes online a Review Process File to accompany accepted manuscripts. As you are aware, this File will be published in conjunction with your paper and will include the referee reports, your point-by-point response and all pertinent correspondence relating to the manuscript.

If you do NOT want this File to be published, please inform the editorial office within 2 days, if you have not done so already, otherwise the File will be published by default [contact: emboreports@embo.org]. If you do opt out, the Review Process File link will point to the following statement: "No Review Process File is available with this article, as the authors have chosen not to make the review process public in this case."

Thank you again for your contribution to EMBO reports and congratulations on a successful publication. Please consider us again in the future for your most exciting work.

Kind regards,

Martina

Martina Rembold, PhD
Senior Editor
EMBO reports

****

THINGS TO DO NOW:

Please note that you will be contacted by Wiley Author Services to complete licensing and payment information. The required 'Page Charges Authorization Form' is available here: https://www.embopress.org/pb-assets/embo-site/er_apc.pdf - please download and complete the form and return to embopressproduction@wiley.com

EMBO Press participates in many Publish and Read agreements that allow authors to publish Open Access with reduced/no publication charges. Check your eligibility: https://authorservices.wiley.com/author-resources/Journal-Authors/open-access/affiliation-policies-payments/index.html

You will receive proofs by e-mail approximately 2-3 weeks after all relevant files have been sent to our Production Office; you should return your corrections within 2 days of receiving the proofs.

Please inform us if there is likely to be any difficulty in reaching you at the above address at that time. Failure to meet our deadlines may result in a delay of publication, or publication without your corrections.

All further communications concerning your paper should quote reference number EMBOR-2022-55895V3 and be addressed to emboreports@wiley.com.

Should you be planning a Press Release on your article, please get in contact with emboreports@wiley.com as early as possible, in order to coordinate publication and release dates.

EMBO Press Author Checklist

Corresponding Author Name: Christian Behrends	
Journal Submitted to: EMBO Report	
Manuscript Number: EMBOR-2022-55895-T	

USEFUL LINKS FOR COMPLETING THIS FORM
The EMBO Journal - Author Guidelines
EMBO Reports - Author Guidelines
Molecular Systems Biology - Author Guidelines
EMBO Molecular Medicine - Author Guidelines

Reporting Checklist for Life Science Articles (updated January 2022)
This checklist is adapted from Materials Design Analysis Reporting (MDAR) Checklist for Authors. MDAR establishes a minimum set of requirements in transparent reporting in the life sciences (see Statement of Task: 10.31222/osf.io/9sm4x). Please follow the journal's guidelines in preparing your manuscript.
Please note that a copy of this checklist will be published alongside your article.

Abridged guidelines for figures
1. Data
The data shown in figures should satisfy the following conditions:
- the data were obtained and processed according to the field's best practice and are presented to reflect the results of the experiments in an accurate and unbiased manner.
- ideally, figure panels should include only measurements that are directly comparable to each other and obtained with the same assay.
- plots include clearly labeled error bars for independent experiments and sample sizes. Unless justified, error bars should not be shown for technical replicates.
- if n<5, the individual data points from each experiment should be plotted. Any statistical test employed should be justified.
- Source Data should be included to report the data underlying figures according to the guidelines set out in the authorship guidelines on Data Presentation.

2. Captions
Each figure caption should contain the following information, for each panel where they are relevant:
- a specification of the experimental system investigated (eg cell line, species name).
- the assay(s) and method(s) used to carry out the reported observations and measurements.
- an explicit mention of the biological and chemical entity(ies) that are being measured.
- an explicit mention of the biological and chemical entity(ies) that are altered/varied/perturbed in a controlled manner.
- the exact sample size (n) for each experimental group/condition, given as a number, not a range;
- a description of the sample collection allowing the reader to understand whether the samples represent technical or biological replicates (including how many animals, litters, cultures, etc.).
- a statement of how many times the experiment shown was independently replicated in the laboratory.
- definitions of statistical methods and measures:
  - common tests, such as t-test (please specify whether paired vs. unpaired), simple χ2 tests, Wilcoxon and Mann-Whitney tests, can be unambiguously identified by name only, but more complex techniques should be described in the methods section;
  - are tests one-sided or two-sided?
  - are there adjustments for multiple comparisons?
  - exact statistical test results, e.g., P values = x but not P values < x;
  - definition of 'center values' as median or average;
  - definition of error bars as s.d. or s.e.m.

Please complete ALL of the questions below.
Select "Not Applicable" only when the requested information is not relevant for your study.

Materials

Newly Created Materials	Information included in the manuscript?	In which section is the information available? (Reagents and Tools Table, Materials and Methods, Figures, Data Availability Section)
New materials and reagents need to be available; do any restrictions apply?	Yes	Material and Methods

Antibodies	Information included in the manuscript?	In which section is the information available? (Reagents and Tools Table, Materials and Methods, Figures, Data Availability Section)
For **antibodies** provide the following information:<br>- Commercial antibodies: RRID (if possible) or supplier name, catalogue number and or/clone number<br>- Non-commercial: RRID or citation	Yes	Material and Methods

DNA and RNA sequences	Information included in the manuscript?	In which section is the information available? (Reagents and Tools Table, Materials and Methods, Figures, Data Availability Section)
**Short novel DNA or RNA including primers, probes**: provide the sequences.	Yes	Material and Methods

Cell materials	Information included in the manuscript?	In which section is the information available? (Reagents and Tools Table, Materials and Methods, Figures, Data Availability Section)
**Cell lines:** Provide species information, strain. Provide accession number in repository **OR** supplier name, catalog number, clone number, and/**OR** RRID.	Yes	Material and Methods
**Primary cultures:** Provide species, strain, sex of origin, genetic modification status.	Not Applicable	
Report if the cell lines were recently **authenticated** (e.g., by STR profiling) and tested for mycoplasma contamination.	Yes	Material and Methods

Experimental animals	Information included in the manuscript?	In which section is the information available? (Reagents and Tools Table, Materials and Methods, Figures, Data Availability Section)
**Laboratory animals or Model organisms:** Provide species, strain, sex, age, genetic modification status. Provide accession number in repository **OR** supplier name, catalog number, clone number, **OR** RRID.	Not Applicable	
**Animal observed in or captured from the field:** Provide species, sex, and age where possible.	Not Applicable	
Please detail **housing and husbandry conditions**.	Not Applicable	

Plants and microbes	Information included in the manuscript?	In which section is the information available? (Reagents and Tools Table, Materials and Methods, Figures, Data Availability Section)
**Plants:** provide species and strain, ecotype and cultivar where relevant, unique accession number if available, and source (including location for collected wild specimens).	Not Applicable	
**Microbes:** provide species and strain, unique accession number if available, and source.	Not Applicable	

Human research participants	Information included in the manuscript?	In which section is the information available? (Reagents and Tools Table, Materials and Methods, Figures, Data Availability Section)
If collected and within the bounds of privacy constraints report on age, sex and gender or ethnicity for all study participants.	Not Applicable	

Core facilities	Information included in the manuscript?	In which section is the information available? (Reagents and Tools Table, Materials and Methods, Figures, Data Availability Section)
If your work benefited from core facilities, was their service mentioned in the acknowledgments section?	Not Applicable	

Design

Study protocol	Information included in the manuscript?	In which section is the information available? (Reagents and Tools Table, Materials and Methods, Figures, Data Availability Section)
If study protocol has been **pre-registered, provide DOI in the manuscript**. For clinical trials, provide the trial registration number **OR** cite DOI.	Not Applicable	
Report the **clinical trial registration number** (at ClinicalTrials.gov or equivalent), where applicable.	Not Applicable	

Laboratory protocol	Information included in the manuscript?	In which section is the information available? (Reagents and Tools Table, Materials and Methods, Figures, Data Availability Section)
Provide DOI OR other citation details if **external detailed step-by-step protocols** are available.	Not Applicable	

Experimental study design and statistics	Information included in the manuscript?	In which section is the information available? (Reagents and Tools Table, Materials and Methods, Figures, Data Availability Section)
Include a statement about **sample size** estimate even if no statistical methods were used.	Not Applicable	
Were any steps taken to minimize the effects of subjective bias when allocating animals/samples to treatment (e.g. **randomization procedure**)? If yes, have they been described?	Not Applicable	
Include a statement about **blinding** even if no blinding was done.	Not Applicable	
Describe **inclusion/exclusion criteria** if samples or animals were excluded from the analysis. Were the criteria pre-established?<br><br>If sample or data points were omitted from analysis, report if this was due to attrition or intentional exclusion and provide justification.	Not Applicable	
For every figure, are **statistical tests** justified as appropriate? Do the data meet the assumptions of the tests (e.g., normal distribution)? Describe any methods used to assess it. Is there an estimate of variation within each group of data? Is the variance similar between the groups that are being statistically compared?	Yes	Figure Legend, Materials and Methods

Sample definition and in-laboratory replication	Information included in the manuscript?	In which section is the information available? (Reagents and Tools Table, Materials and Methods, Figures, Data Availability Section)
In the figure legends: state number of times the experiment was **replicated** in laboratory.	Yes	Figure Legend
In the figure legends: define whether data describe **technical or biological replicates**.	Yes	Figure Legend

Ethics

Ethics	Information included in the manuscript?	In which section is the information available? (Reagents and Tools Table, Materials and Methods, Figures, Data Availability Section)
Studies involving **human participants**: State details of **authority granting ethics approval** (IRB or equivalent committee(s), provide reference number for approval.	Not Applicable	
Studies involving **human participants**: Include a statement confirming that **informed consent** was obtained from all subjects and that the experiments conformed to the principles set out in the WMA Declaration of Helsinki and the Department of Health and Human Services Belmont Report.	Not Applicable	
Studies involving **human participants:** For publication of **patient photos**, include a statement confirming that consent to publish was obtained.	Not Applicable	
Studies involving experimental **animals**: State details of **authority granting ethics approva**l (IRB or equivalent committee(s), provide reference number for approval. Include a statement of compliance with ethical regulations.	Not Applicable	
Studies involving **specimen and field samples:** State if relevant **permits** obtained, provide details of authority approving study; if none were required, explain why.	Not Applicable	

Dual Use Research of Concern (DURC)	Information included in the manuscript?	In which section is the information available? (Reagents and Tools Table, Materials and Methods, Figures, Data Availability Section)
Could your study fall under dual use research restrictions? Please check biosecurity documents and list of **select agents and toxins** (CDC): https://www.selectagents.gov/sat/list.htm	Not Applicable	
If you used a select agent, is the security level of the lab appropriate and reported in the manuscript?	Not Applicable	
If a study is subject to dual use research of concern regulations, is the name of the **authority granting approval and reference number** for the regulatory approval provided in the manuscript?	Not Applicable	

Reporting

The MDAR framework recommends adoption of discipline-specific guidelines, established and endorsed through community initiatives. Journals have their own policy about requiring specific guidelines and recommendations to complement MDAR.

Adherence to community standards	Information included in the manuscript?	In which section is the information available? (Reagents and Tools Table, Materials and Methods, Figures, Data Availability Section)
State if relevant guidelines or checklists (e.g., **ICMJE, MIBBI, ARRIVE, PRISMA**) have been followed or provided.	Not Applicable	
For **tumor marker prognostic studies,** we recommend that you follow the **REMARK** reporting guidelines (see link list at top right). See author guidelines, under 'Reporting Guidelines'. Please confirm you have followed these guidelines.	Not Applicable	
For **phase II and III randomized controlled trials**, please refer to the **CONSORT** flow diagram (see link list at top right) and submit the CONSORT checklist (see link list at top right) with your submission. See author guidelines, under 'Reporting Guidelines'. Please confirm you have submitted this list.	Not Applicable	

Data Availability

Data availability	Information included in the manuscript?	In which section is the information available? (Reagents and Tools Table, Materials and Methods, Figures, Data Availability Section)
Have **primary datasets** been deposited according to the journal's guidelines (see 'Data Deposition' section) and the respective accession numbers provided in the Data Availability Section?	Yes	Data Availability Section
Were **human clinical and genomic datasets** deposited in a public access-controlled repository in accordance to ethical obligations to the patients and to the applicable consent agreement?	Not Applicable	
Are **computational models** that are central and integral to a study available without restrictions in a machine-readable form? Were the relevant accession numbers or links provided?	Not Applicable	
If publicly available data were reused, provide the respective **data citations in the reference list**.	Not Applicable	
