# Supplementary material for: C9orf72 protein quality control by UBR5‐mediated heterotypic ubiquitin chains
Source: EMBO Rep. 2023 Jun 15;24(8):e55895. doi: 10.15252/embr.202255895 (PMC10398660; doi:10.15252/embr.202255895)

Figure EV2A

Figure EV2B

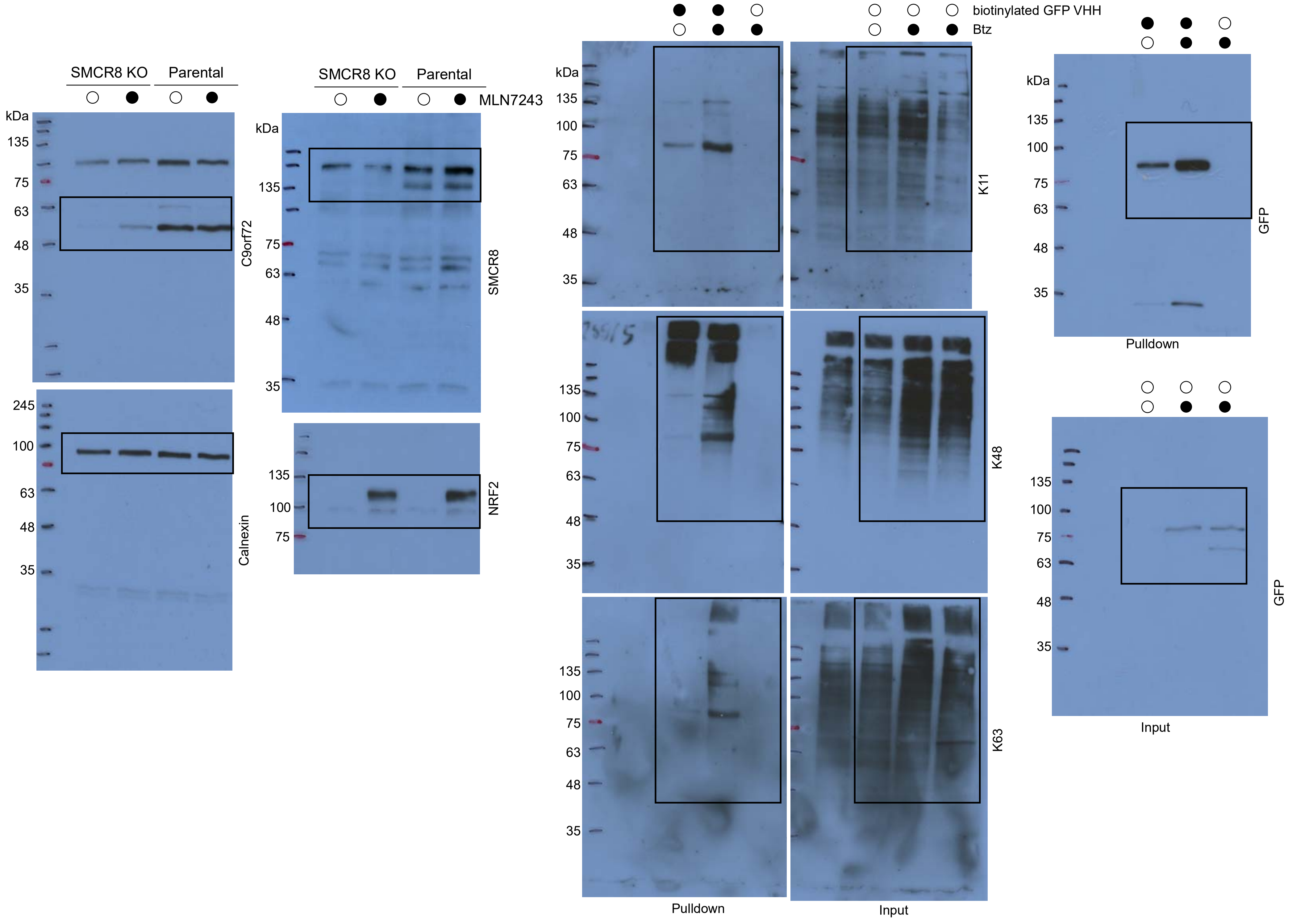

Supplement: Supplementary file 3 — Source Data for Expanded View [file EMBR-24-e55895-s010.zip › EV_Figure_Source_Data/Figure_EV_2.zip!/Figure EV 2/Figure EV2.pdf]
