# Supplementary material for: C9orf72 protein quality control by UBR5‐mediated heterotypic ubiquitin chains
Source: EMBO Rep. 2023 Jun 15;24(8):e55895. doi: 10.15252/embr.202255895 (PMC10398660; doi:10.15252/embr.202255895)
Supplement: Supplementary file 3 — Source Data for Expanded View [file EMBR-24-e55895-s010.zip › EV_Figure_Source_Data/Figure_EV_3.zip!/Figure EV 3/Figure EV3.pdf]

Figure EV3

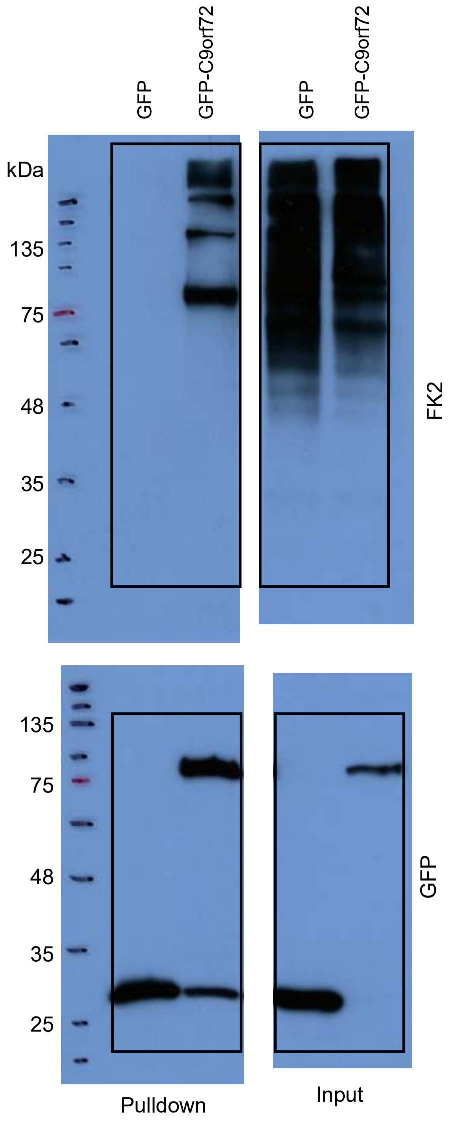
GFP
GFP-C9orf72
GFP
GFP-C9orf72
kDa
135
75
48
35
25
FK2
135
75
48
35
25
GFP
Pulldown
Input
