# Supplementary material for: C9orf72 protein quality control by UBR5‐mediated heterotypic ubiquitin chains
Source: EMBO Rep. 2023 Jun 15;24(8):e55895. doi: 10.15252/embr.202255895 (PMC10398660; doi:10.15252/embr.202255895)

Figure EV1A

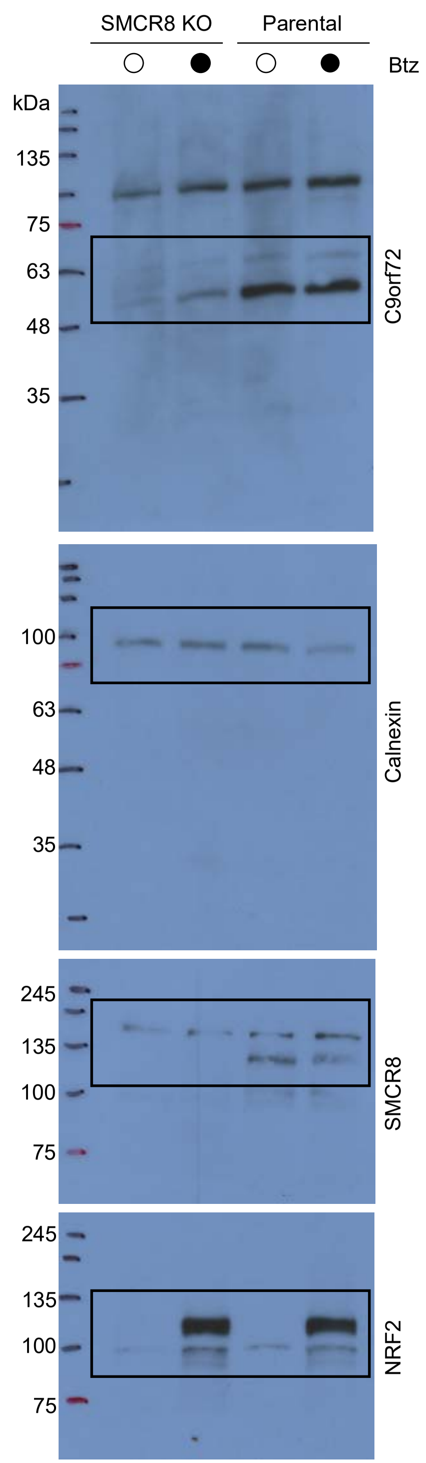

Figure EV1B

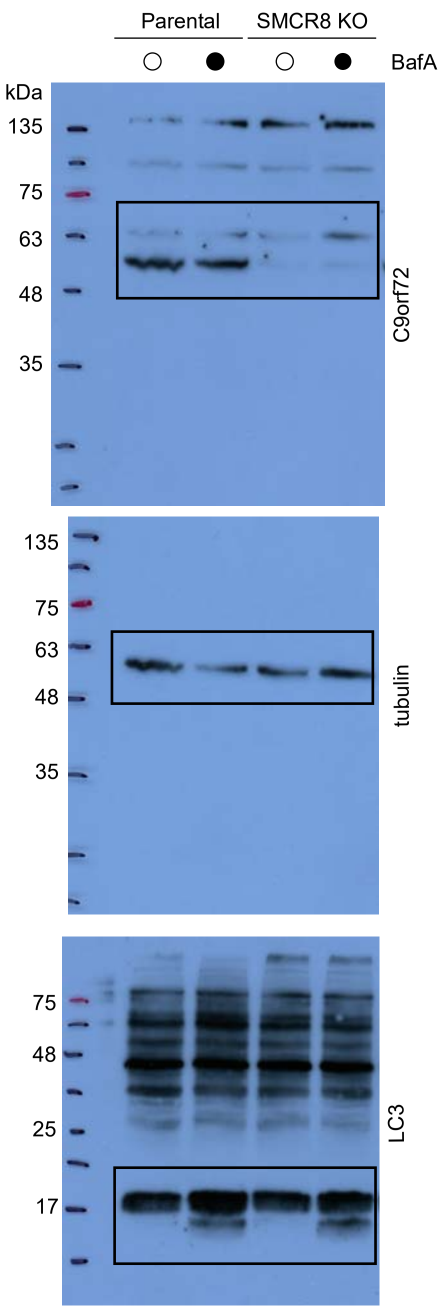

Figure EV1C

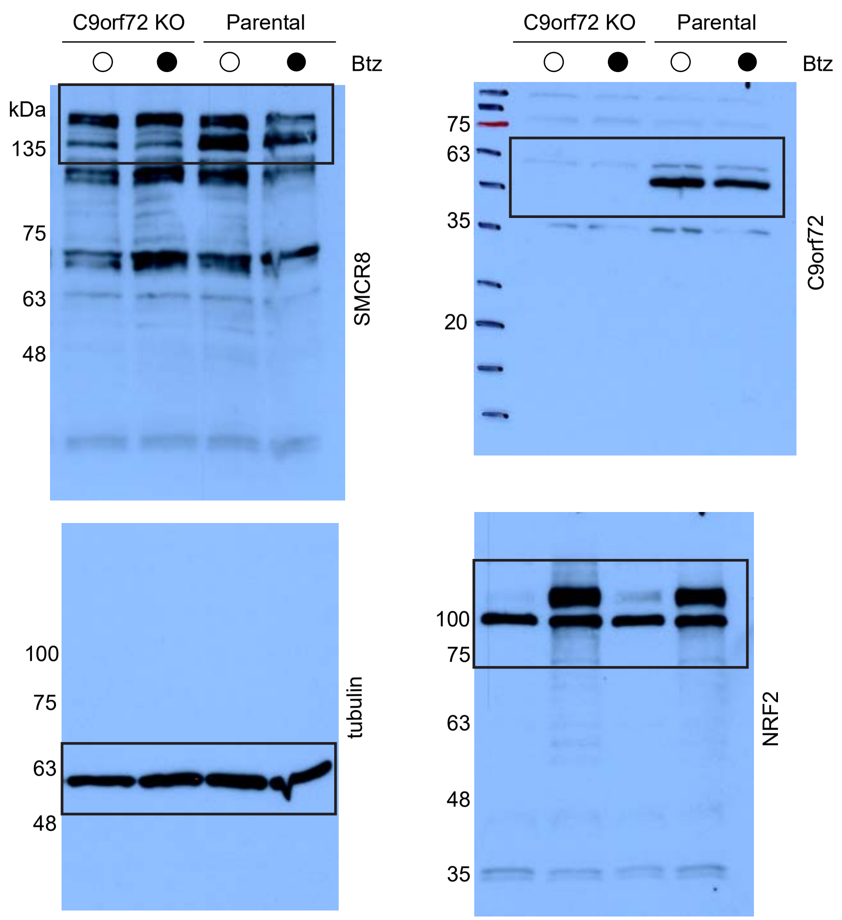

Figure EV1D

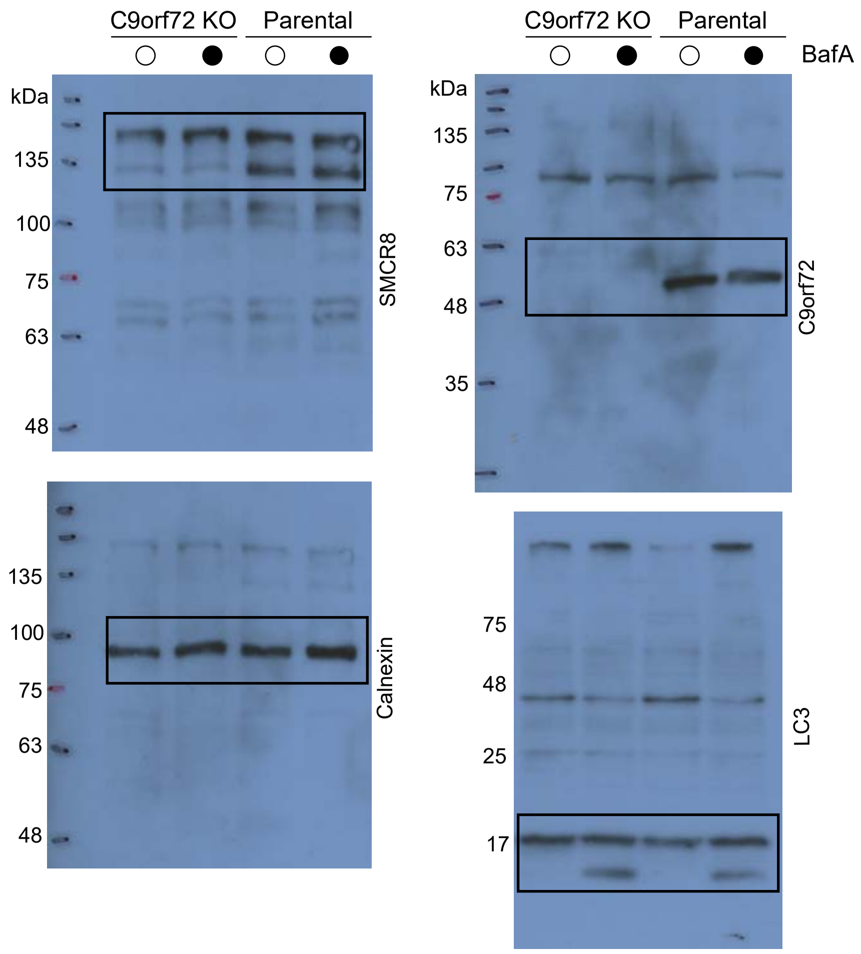

Figure EV1E

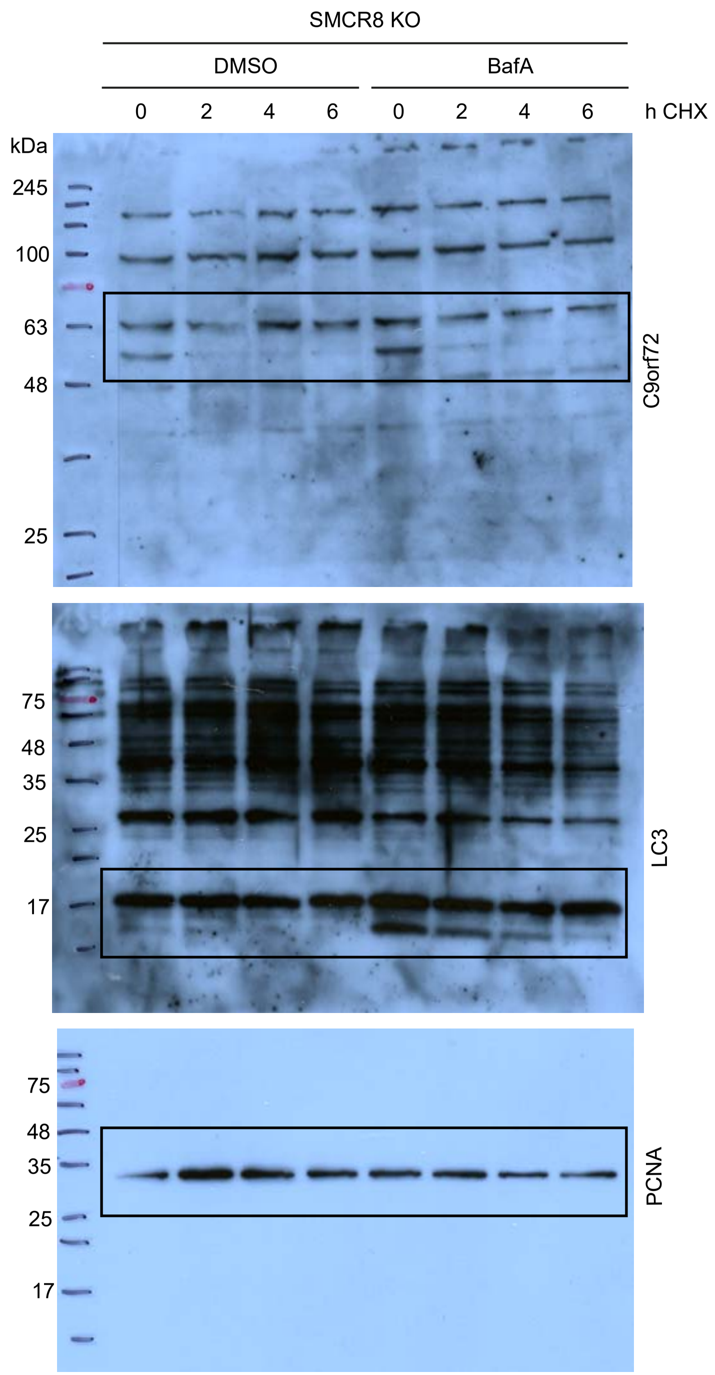

Supplement: Supplementary file 3 — Source Data for Expanded View [file EMBR-24-e55895-s010.zip › EV_Figure_Source_Data/Figure_EV_1.zip!/Figure EV 1/Figure EV1.pdf]
